# Synthesis of Substituted 1*H*-Phenalen-1-ones and Nitrogen-Containing Heterocyclic Analogues as Potential Anti-Plasmodial Agents

**DOI:** 10.3390/molecules30244667

**Published:** 2025-12-05

**Authors:** Teresa Abad-Grillo, Grant McNaughton-Smith, Mónica Blanco Freijo, David Gutiérrez, Ninoska Flores

**Affiliations:** 1 Departamento de Química Orgánica, Universidad de La Laguna, Avenida Astrofísico Francisco Sánchez, 2, 38206 La Laguna, Tenerife, Spain; 2 Centro Atlántico del Medicamento S.A. (CEAMED S.A.), Parque Científico y Tecnológico de Tenerife (PCTT), 38200 La Laguna, Tenerife, Spain; 3 Instituto de Investigaciones Fármaco Bioquímicas, Facultad de Ciencias Farmacéuticas y Bioquímicas, Universidad Mayor de San Andrés, Avenida Saavedra 2224, Miraflores, La Paz 00067, Bolivia

**Keywords:** substituted 1*H*-phenalen-1-ones, nitrogen-containing heterocyclic structural analogs, phenoxazine, acridin-7-one, anti-plasmodial activity, structure-activity relationship

## Abstract

Given the known biological activity of both natural and synthetic substituted 1*H*-phenalen-1-ones, the generation of a small chemically and structurally diverse 1*H*-phenalen-1-one-based library of compounds was warranted. Herein, we have synthesized several groups of compounds to broaden and improve the chemical diversity of our 1*H*-phenalen-1-one collection. Additionally, we have also introduced hydroxyl, amides or carboxylic groups onto the core, or nitrogen atoms into the core, to increase the chemical diversity while also lowering the ClogP values to aid their water solubility. Notably, we have also improved the synthetic routes to several compounds of interest and have observed the unexpected formation of phenoxazine and acridin-7-one systems during cross-coupling reactions with polyfunctional anilines. Combining the compounds generated in this work with previous ones has enabled us to create a library of chemically diverse 1*H*-phenalen-1-one available for screening assays. Evaluation of anti-plasmodial activity against the chloroquine-resistant *Plasmodium falciparum* strain FCR3 revealed four compounds with notable activity, three of which exhibited IC_50_ values below 1 µM, while none displayed significant cytotoxicity at 10 µM.

## 1. Introduction

1*H*-Phenalen-1-one-based compounds have been isolated from both plants and fungi, where they are believed to play a key role in the organisms’ defense mechanisms [1]. Interestingly, substituted 1*H*-phenalen-1-ones, whether natural or synthetic, have been shown to display a broad range of biological activities.

**Figure 1:**
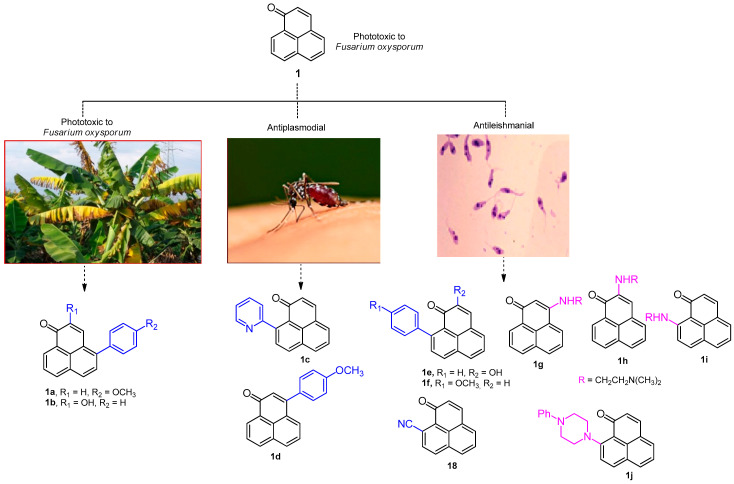
A representative collection of 1*H*-phenalen-1-one-based bioactive compounds.

Several phenalenones are known to be phototoxins [2,3]. For example, it has been shown that exposure to UV light (355 nm) increased the antifungal activity against *Fusarium oxysporum* of some natural and synthetic phenylphenalenones, and their increased activity correlated with their quantum yields (**1a**–**b**) (Figure 1) [4]. Likewise, Otálvaro and colleagues [5], demonstrated that a collection of phenalenones had improved antifungal activities against *Mycosphaerella fijiensis* when exposed to light. *M. fijiensis* and *F. oxysporum* are both known to reduce the production of banana plants by up to 20%.

Phenalenone-based compounds have also shown activity against Leishmania species, including compounds **1e**, **1f** and **18** (Figure 1) [6]. Of note, some amine-functionalized phenalenones, such as **1g**–**j** (Figure 1), have demonstrated 3- to 5-fold greater potency against intracellular amastigotes of *L. amazonensis* compared to miltefosine, a clinically approved anti-leishmanial drug [7].

Further research has shown that 9- and 3-phenylphenalenone derivatives, as well as 9-heteroaryl-phenalenones, also possess antiplasmodial effects against both chloroquine-sensitive and chloroquine-resistant strains of *Plasmodium falciparum* [8]. Malaria, which is caused by various *Plasmodium* species, remains the leading parasitic disease globally in terms of morbidity and mortality, particularly in developing countries, where it imposes substantial social and economic burdens. Although effective treatments exist, they face significant limitations such as resistance, high costs, and complex treatment regimens [9]. Compounds **1c** and **1d** (Figure 1) represent a new structural class of antiplasmodial agents, offering a new scaffold for antimalarial drug development [8].

In general, the chemical synthesis of substituted phenalenones can be achieved by functionalizing pre-formed phenalenones [6,7,10,11,12,13,14,15] or by constructing the phenalenone core from pre-designed building blocks such as functionalized naphthalenes [16,17,18].

**Figure 2:**
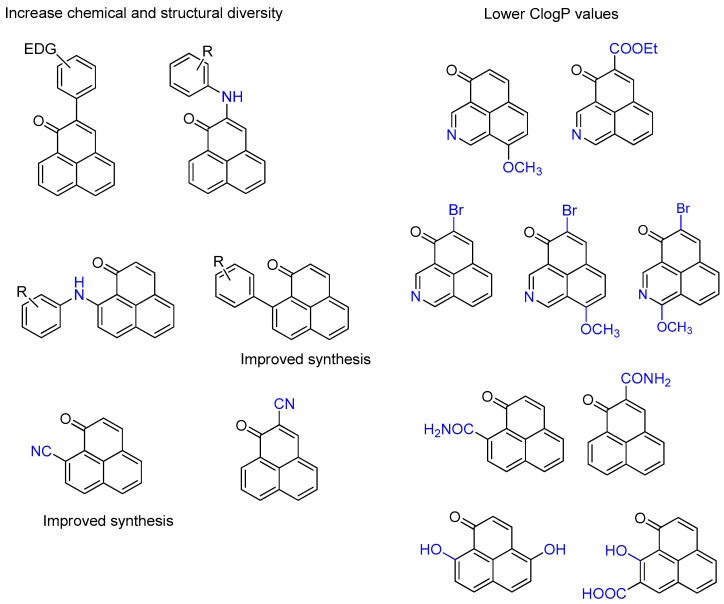
Compounds of interest described in this work.

Given their interesting range of biological activities, we wished to supplement and broaden the chemical diversity and novelty of our 1*H*-phenalen-1-one collection, with the goal of generating a small phenalenone-based library ready for biological screening (Figure 2). As the phenalenone core itself has a relatively high ClogP value of 2.84 and a low predicted water solubility, we also wanted to investigate whether we could generate analogs with lower ClogP values, either by adding polar groups (-OH, -COOH, -CONH_2_) to the core or by incorporating a nitrogen atom directly into the core. Additionally, where possible, we also wanted to improve the synthetic routes to these phenalenones.

## 2. Results and Discussion

**Preparation of 2-aryl/heteroaryl phenalenones**

**Scheme 1:**
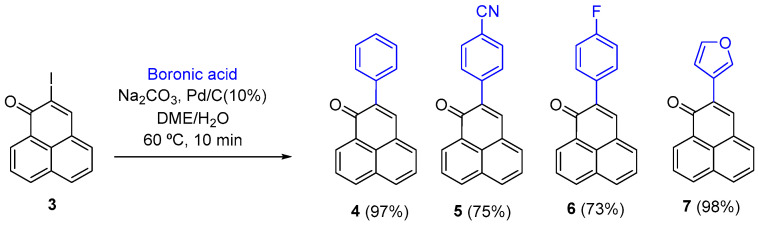
Synthesis of 2-substituted 1*H*-phenalen-1-ones (**4**–**7**).

Previously we had only prepared 2-aryl-phenalenones with electron-donating groups (-OMe) on the aryl entity [19], so to improve the diversity of this sub-series we also needed to incorporate electron-withdrawing groups. It has been demonstrated that the Suzuki–Miyaura coupling works with 2-iodocycloenones, Pd/C, and arylboronic acids. Interestingly, when the reaction was carried out with 2-bromocyclohex-2-en-1-one, no reaction occurred [20]. Based on these results, compounds (**4**–**7**) (Scheme 1) were rapidly and efficiently synthesized using a combination of 2-iodo-1*H*-phenalen-1-one **3** as the phenalenone source and ligand-free palladium on carbon (Pd/C) as the cross-coupling agent.

**Preparation of 9-aryl/heteroaryl phenalenones**.

**Scheme 2:**
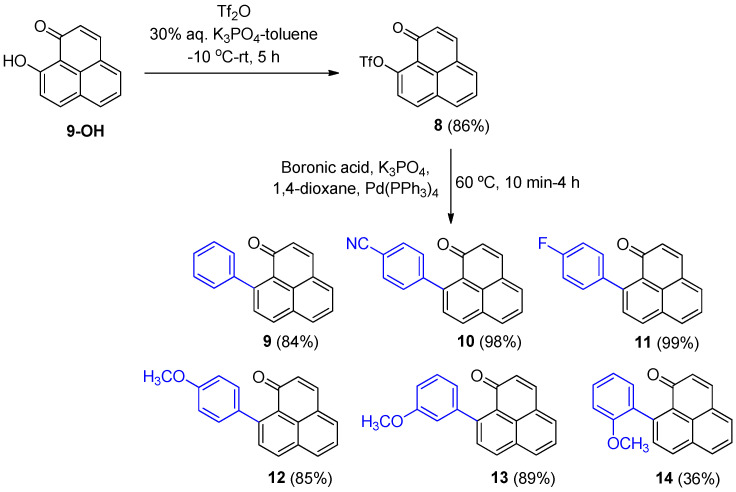
Synthesis of 9-substituted aryl 1*H*-phenalen-1-ones (**9**–**14**).

Compounds of this type have been prepared using a two-step process [6]. Firstly, a nucleophilic addition using a Grignard reagent followed by oxidation with DDQ. This process, although viable, had certain drawbacks, notably the limitations of functional groups in the Grignard reagent, and often the reagent added to the carbonyl group as well as the 9-position, even at low temperatures. Given the success of the Suzuki–Miyaura coupling observed for the 2-aryl derivatives (**4**–**7**), we investigated its use to generate derivatives at the 9-position. Iodides, bromides, and triflates are commonly used in these cross-coupling reactions. Because triflate **8** can be prepared directly from the 9-OH precursor (Scheme 2)—and the corresponding 9-iodide can be generated from **8** [21]—the 9-triflate phenalenone (**8**) was selected first, thereby avoiding the additional step required to convert **8** into the iodide. The conditions used in Scheme 1 were not compatible with the triflate (**8**) as it was rapidly hydrolyzed. Fortunately, more standard conditions (K_3_PO_4_, Pd(PPh_3_)_4,_ in 1,4-dioxane at 60 °C), provided the 9-aryl derivatives (**9**–**14**) in one-step and with yields typically 10–30% higher than the previous two-step process [12,13,14] (Scheme 2).

**Preparation of 9-amino-aryl-1*H*-phenalenones**.

**Scheme 3:**
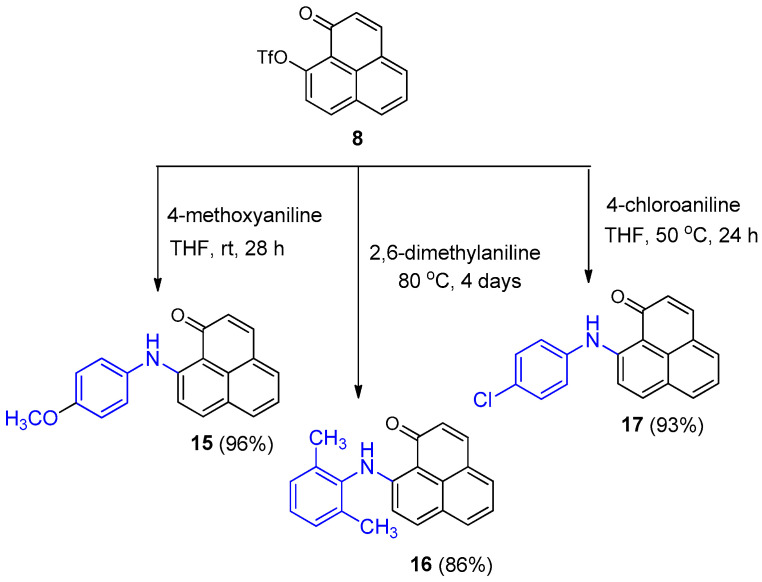
Synthesis of 9-arylamino 1*H*-phenalen-1-ones (**15**–**17**).

The novel 9-aminoaryl-phenalenone derivatives, compounds **15**–**17** (Scheme 3), were synthesized from compound **8** and an excess of the corresponding anilines via a nucleophilic substitution reaction. For the synthesis of **16**, neat 2,6-dimethylaniline was used, with no THF added as solvent Although they required long reaction times (up to 4 days in the case of **16**) the products were all obtained in high yields.

**Preparation of 2- and 9-cyano/amido 1*H*-phenalenone derivatives**

**Scheme 4:**
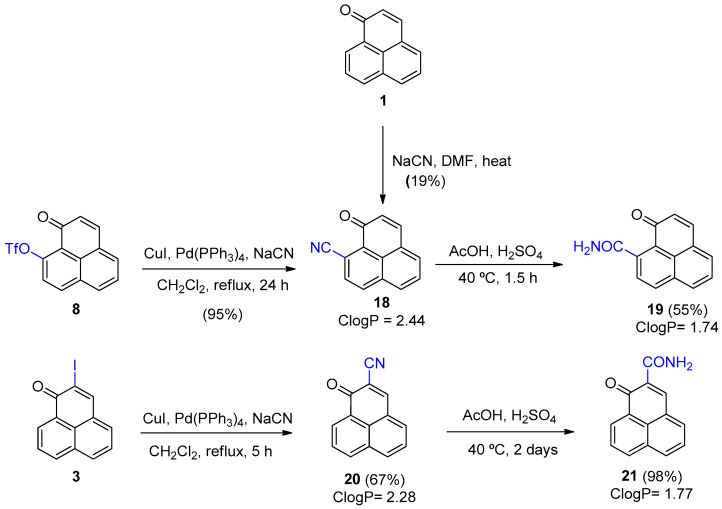
Synthesis of 9- and 2-cyano 1*H*-phenalen-1-ones (**18** and **20**) and amides (**19** and **21**).

Compound **18** possesses interesting antiprotozoal activities, especially against *L. amazonesis*, and *L. donovani*. Unfortunately, our previous synthesis of **18** from **1** using sodium cyanide in aqueous DMF followed by air oxidation only afforded a 19% yield [6]. We therefore investigated whether other synthetic routes to **18** were more viable (Scheme 4). An initial attempt using a palladium/copper-catalyzed cyanation of triflate **8** in acetonitrile under reflux afforded **18** in a 50% yield. The product was accompanied by a significant amount of 9-hydroxy-1*H*-phenalen-1-one revealing that triflate hydrolysis was an issue. Changing the solvent to dichloromethane eliminated this side reaction and increased the yield of **18** to 95% after 24 h at reflux. Under the same reaction conditions 2-iodo-1*H*-phenalenone **3** was transformed cleanly into the 2-cyano analog (**20**). The difference in yields between isomers **18** (95%) and **20** (67%) is difficult to ascertain as a direct comparison is difficult since the starting materials are different. Interestingly, no 2-cyano **20** was formed when 2-bromo-1*H*-phenalenone **2** was used as the phenalenone source.

As one of our goals was also to obtain 1*H*-phenalenone compounds with lower ClogP values, we converted the cyano compounds **18** and **20** into their respective amido analogs **19** and **21** using acidic conditions. A notable difference in the yields of **19** and **21** was observed. Both reactions proceeded to completion, although **21** took considerably longer. We observed that immediately upon addition of concentrated H_2_SO_4_ to a solution of **18** in AcOH, the solution turned dark. Moreover, after extraction of the organics and neutralization of the acid, a significant amount of a dark, insoluble material (char) remained suspended in the aqueous layer. In contrast, no darkening or formation of insoluble material was observed when using **20**, even after heating at 40 °C for two days.

**Preparation of 2-amino-aryl/heteroaryl 1*H*-phenalenone derivatives and the unexpected formation of phenoxarine and acridine derivatives**.

**Scheme 5:**
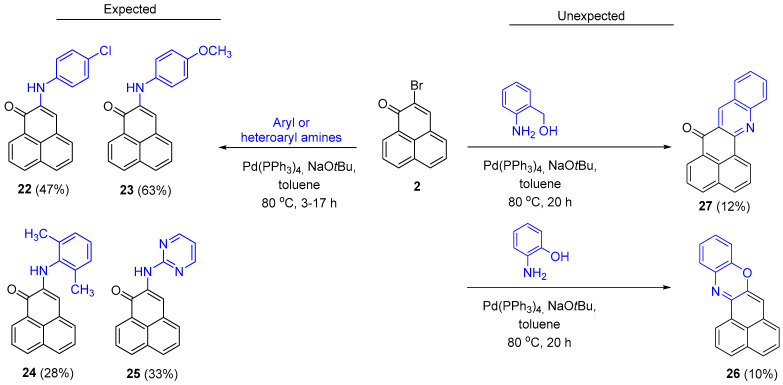
Synthesis of 2-substituted 1*H*-phenalen-1-ones (**22**–**25**), the phenoxazine **26** and the acridin-7-one **27**.

Cross-coupling of 2-bromo-1*H*-phenalenone **2** with weakly nucleophilic amines using Büchwald-Hartwig conditions, generally provides the desired 2-aryl/heteroaryl products as illustrated by the generation of the novel analogs (**22**–**25**, Scheme 5). Even the sterically hindered 2,6-dimethylaniline and the delocalized pyrimidin-2-amine afforded their expected products.

Interestingly, however, using either 2-aminophenol or (2-aminophenyl)methanol as the aniline did not lead to the generation of their predicted coupled products. Instead, the only products that could be isolated and characterized were the novel naphtho[1,8-*ab*]phenoxazine (**26**) and 7*H*-naphtho[1,8-*bc*]acridin-7-one (**27**), respectively, albeit in low yields (Scheme 5).

**Figure 3:**
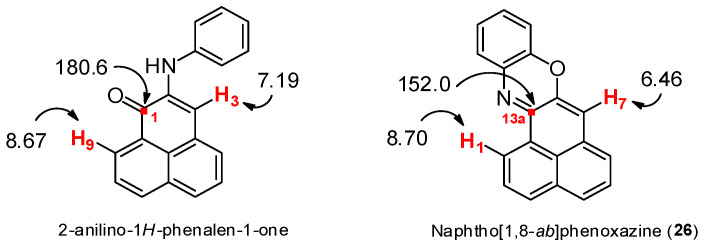
Comparative analysis of NMR chemical shifts between the anilino derivative and **26**.

To confirm the structures of product **26** and **27**, ^1^H NMR, ^13^C NMR, mass and IR spectra were performed, in addition to bi-dimensional COSY, HMBC, and HSQC NMR experiments. In the ^1^H NMR spectrum of **26**, we observed 11 signals corresponding to aromatic protons (Figure 3), one of which appeared as a singlet at 6.46 ppm, in contrast to the singlet at 7.19 ppm for 2-anilino-1*H*-phenalen-1-one, the product with the structure closest to what we expected in this reaction [7]. In the ^13^C NMR spectrum, the quaternary carbon signal corresponding to the enone carbonyl group around 180 ppm was absent. Instead, a quaternary carbon signal was present, shifted around 152 ppm (Figure 3), likely due to a C=N group.

**Figure 4:**
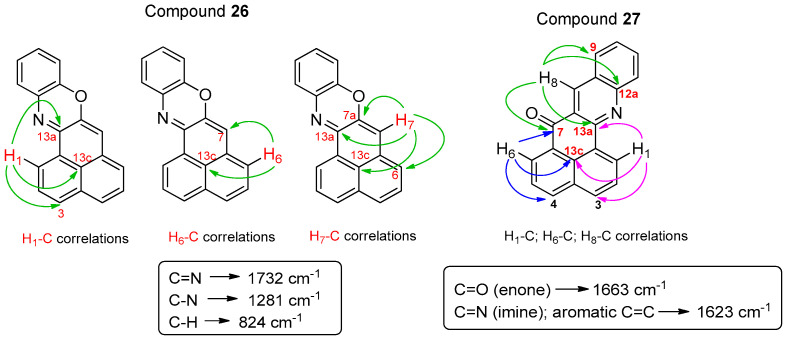
Key HMBC correlations at two or three bonds and IR bands of compounds **26** and **27**.

Based on the couplings observed in the COSY experiment, we assigned the protons H-1 to H-6 of the 1*H*-phenalen-1-one nucleus. The corresponding CH carbons were assigned using HSQC. To assign quaternary carbons we used HMBC. The IR bands of compound **26** were in agreement with its functional groups (Figure 4).

**Scheme 6:**
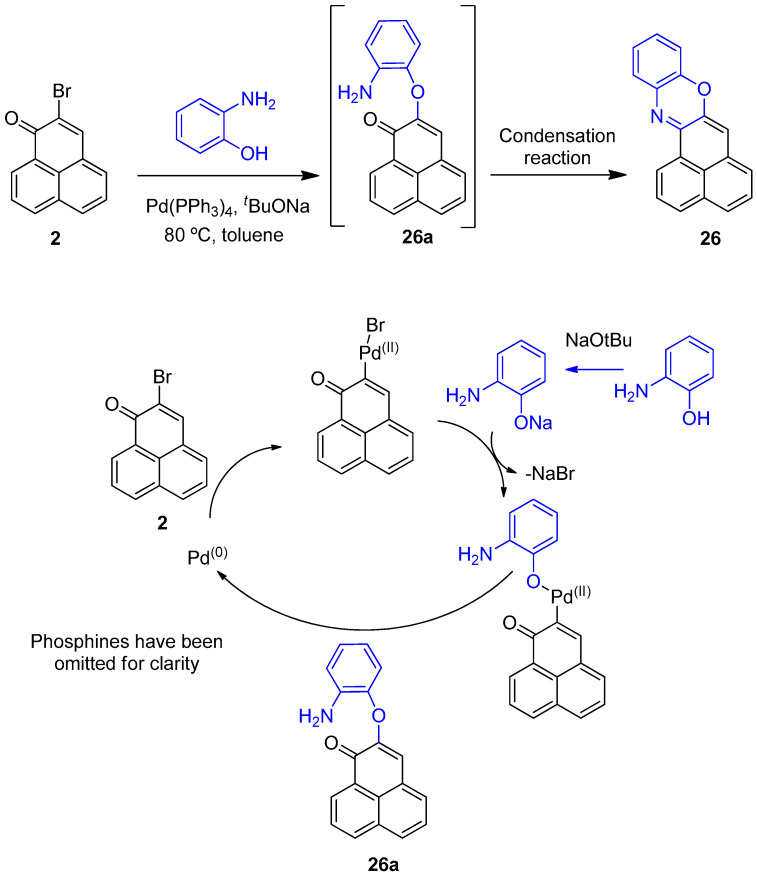
Proposed mechanism for the cross-coupling of 2-aminophenol with 2-bromo-1*H*-phenalen-1-one (**2**) to give **26a**.

Although less well known, Buchwald–Hartwig reaction conditions can also generate diaryl ethers, when phenolic groups are present [22,23]. Under the reaction conditions used the phenoxy group in 2-aminophenol can be deprotonated by the base forming the corresponding phenoxide ion. The phenoxide ion, being more nucleophilic than the aniline group, will be preferentially incorporated into the palladium catalytic cycle and will generate the corresponding diaryl ether intermediate **26a** (Scheme 6). The aniline group is now ideally positioned to undergo an intramolecular imine reaction resulting in compound **26**. The use of more electron-rich and bulkier ligands than triphenylphosphine has been reported to improve the yields of these types of diaryl ethers, and may explain the relatively low yield observed [24].

For compound **27**, all proton and CH carbon signals were assigned using two-dimensional NMR experiments. High-resolution mass spectrometry established its molecular formula as C_20_H_11_NO, consistent with a highly unsaturated structure, and the IR spectrum showed bands in agreement with the proposed functional groups (Figure 4).

Mechanistically, the formation of **27** under these reaction conditions is intriguing. An initial Buchwald-Hartwig coupling would attach the nitrogen to position 2, from where it would seem improbable to generate the characterized product **27**. Of note, the synthesis of compound **27** has been previously described by Kuroki and co-workers [25] albeit using relatively harsh conditions. Although they also used 2-(aminophenyl)methanol as the amine source, they used 3-hydroxyphenalenone as the phenalenone source. Apart from solvents, no other additives were employed. Boiling in DMSO only generated a trace amount of **27**, while heating in boiling HMPT (195 °C) increased the yield of **27** to 33%.

Further experimental studies will be needed to assess the role of each reagent (Pd(Ph_3_P)_4_, NaO*t*Bu) in our reaction conditions, and to try and elucidate a plausible mechanism for the formation of **27** under these milder reaction conditions.

**Preparation of hydroxyl and carboxylic acid containing 1*H*-phenalen-1-ones**

As several naturally occurring phenalenones with antifungal activities are hydroxylated, or even poly-hydroxylated [4], we wanted to have more hydroxylated examples in our collection.

**Scheme 7:**
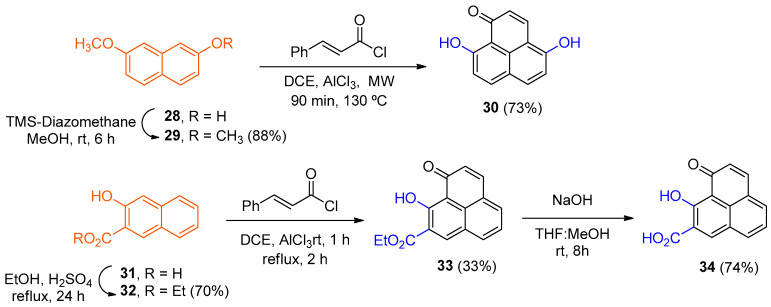
Synthesis of hydroxylated analogs (**30**, **33** and **34**).

The desired derivatives **30** and **33** were synthesized by constructing the phenalenone core using hydroxylated naphthalene’s as building blocks (Scheme 7). Using the same methodology reported for the synthesize 9-hydroxy-1*H*-phenalen-1-one [17,18,21], but with **29** as the starting material, a complex mixture of products was obtained from which **30** was isolated in yields < 30%. However, substitution of conventional heating for microwave irradiation in a sealed tube afforded the fully demethylated product **30** in 73% yield. Ethyl 3-hydroxynaphthalene-2-carboxylate (**32**) was converted into its corresponding 1*H*-phenalenone derivative **33** in a similar fashion; however, the microwave conditions were too harsh for this substrate and so the original conditions were preferred. Subsequent hydrolysis of **33** furnished compound **34**, which combines the 1*H*-phenalen-1-one core with a salicylic acid moiety.

**Preparation of nitrogen containing 1*H*-phenalen-1-ones**

**Scheme 8:**
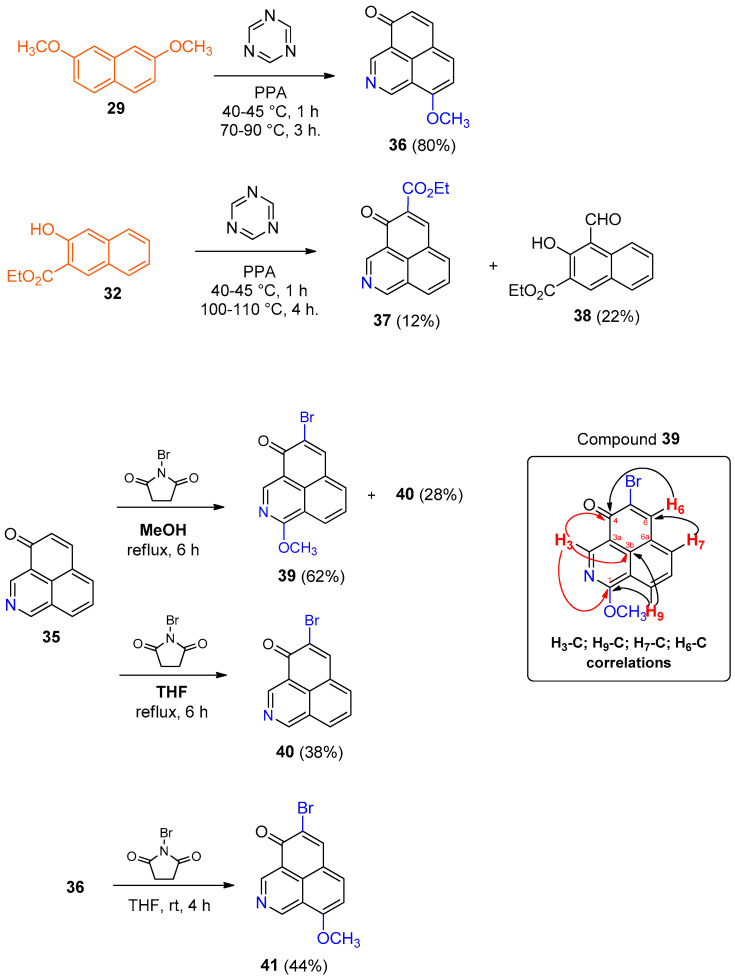
Synthesis of heterocyclic 1*H*-phenalen-1-one analogs (**36**–**37** and **39**–**41**) and key HMBC correlations at three bonds of compound **39**.

The 4*H*-benzo[*de*]isoquinolin-4-one **35** derivatives **36** and **37** were synthesized from naphthalenes **29** and **32** using 1,3,5-triazine and PPA (polyphosphoric acid) [26] (Scheme 8). The poorer solubility of **32** compared to **29** in PPA was probably accountable for the observed lower yield of **37** compared to **36**.

To enable further functionalization of these heterocycles in a manner similar to the phenalenone core, we explored the introduction of a suitable leaving group at the C-2 position. Bromination of compound **35** with NBS in MeOH afforded compound **39** (62% yield), along with compound **40** (28% yield). Compound **39** was likely formed via activation of the pyridine nitrogen by NBS, followed by regioselective addition of methanol (Scheme 7). Two-dimensional COSY, HMBC, and HSQC NMR experiments were performed to determine which of the positional isomers [either 5-bromo-3-methoxy- or 5-bromo-1-methoxy-4*H*-benzo[*de*]isoquinolin-4-one (**39**)], had been obtained. Based on the couplings observed in the COSY spectrum, we assigned protons H-6 and H-8. The corresponding CH carbons were identified using HSQC. For the assignment of quaternary carbons, we relied on HMBC correlations (Scheme 8), confirming **39** as the isomer formed.

When THF was used instead of MeOH, compounds **40** and **41** were obtained from compounds **35** and **36**, respectively (Scheme 8).

**In Vitro Anti-plasmodium Activity and Cytotoxicity**

To date, thirty phenalenone-based compounds of the total synthetized, including the 1*H*-phenalen-1-one (**1**) as a starting material and compounds **2**, **3**, 9-hydroxy-1*H*-phenalen-1-one (**9-OH**) and **35** as intermediates, have been investigated for their activity against the chloroquine-resistant *P. falciparum* strain FCR3, and the results are summarized in Table 1. The cytotoxicity of these compounds was also evaluated by measuring their ability to reduce the viability of MCF-7 cells at 10 µM.

Four compounds exhibited antiplasmodium activity, with three having IC_50_ values < 1 µM. The most potent compound was the 9-cyano derivative (**18**). Interestingly, neither the 2-cyano compound (**20**) nor the 9-amido compound (**19**) were active, even at 10 µM. Extending the cyano group by introducing a phenyl spacer at the 9-position, compound (**10**), was also shown to be detrimental to activity. The 9-hydroxy compound also possessed sub-micromolar activity which was unfortunately lost upon the addition of either an ethyl ester (**33**) or carboxylic acid (**34**) in the adjacent 8-position. Notably, the introduction of another hydroxyl group at position 4, compound (**30**), also diminished antiplasmodium activity. While the 2-bromo derivative (**2**) was active (IC_50_ = 0.7 µM), substituting it for the larger iodo (**3**), phenyl (**4**) or furan (**7**) groups again caused a significant loss in antiplasmodium activity. Finally, the introduction of a nitrogen atom into the 8-position (compound **35**) of phenaleonone (**1**) resulted in a complete loss of activity relative to the parent structure. On a more positive note, none of the tested compounds produced significant levels of cytotoxicity at 10 µM.

Overall, these active phenalenone derivatives exhibited improved antiplasmodial activity compared to previously tested compounds, such as **1c** (IC_50_ = 21.0 µM) and **1d** (IC_50_ = 2.1 µM) [8] (Figure 1). Importantly, the active compounds showed no evidence of cytotoxicity with IC_50_ > 10 μM and possess favorable predicted physicochemical properties (Table 2).

## 3. Materials and Methods

### 3.1. General Methods

All of the reagents and solvents were obtained from Aldrich Chemical Co. (Merck KGaA, Darmstadt, Germany) and used without further purification. Reactions with sensitive reagents were performed under an inert atmosphere (argon or nitrogen), and organic solvents were dried using standard methods. Unless otherwise stated, solvents were removed under reduced pressure using a rotary evaporator at 40–60 °C. All of the reactions were monitored via analytical thin layer chromatography (TLC) on POLIGRAM^®^ SIL G/UV_254_ silica gel-coated plates (0.20 mm) from MACHEREY-NAGEL (Düren, Germany). Column chromatography was performed on silica gel 60 (0.063–0.20 mm) from MERCK (Merck KGaA, Darmstadt, Germany). Preparative thin layer chromatography was carried out with GF silica gel plates (1 mm) with fluorescent indicator 254 nm (UNIPLATE) (Merck KGaA, Darmstadt, Germany). The compounds were visualized using ultraviolet light (254 nm). ^1^H and ^13^C NMR spectra were recorded at room temperature (rt) on a Bruker Avance 500 or 600 MHz NMR spectrometer in the solvent indicated (Bruker Española S.A., Madrid, Spain). Data for ^1^H NMR are reported as follows: chemical shift (δ ppm), integration, multiplicity and coupling constant (Hz). ^13^C NMR analyses are reported in terms of chemical shift. IR spectra were recorded as a thin film on NaCl plates with a BRUKER IFS 66 spectrophotometer. UV spectra were recorded on a JASCO modelo V-560 spectrophotometer (Jasco Analítica Spain, S.L., Madrid, Spain). Melting points were determined using a Stuart Scientific SMP11 instrument. Low-resolution (EIMS) and high-resolution mass spectrometry (HRMS) were performed on a Micromass VG AutoSpec magnetic tri-sector (EBE geometry) mass spectrometer (Manchester, UK) via electrospray ionization (ESI) or electron impact (EI) (Agilent Technologies Spain, S.L., Barcelona, Spain). Microwave reactions were performed using a Biotage^®^ Initiator (software version 2.5). Lyophilization was performed using a CHRIST ALPHA 2–4 lyophilizer (TELSTAR S.L.U., Terrassa, Spain).

### 3.2. Synthesis

Compounds **2** [28], **3 [29]**, **8** [21] and **35** [26] were prepared as described in the references.

#### 3.2.1. Synthesis of 2-Bromo-1*H*-phenalen-1-one (**2**)

1*H*-Phenalen-1-one (**1**) (3.0 g, 16.6 mmol), neutral alumina (22.6 g), and NBS (5.0 g, 28.3 mmol) were mixed in a mortar until a uniformly yellow mixture was obtained. This mixture was then heated at 45 °C for 22 h. The crude product was filtered, washing with CH_2_Cl_2_, and the solvent was evaporated to dryness under reduced pressure using a rotary evaporator. The resulting mixture was washed with hot water and filtered while hot to afford compound **2** (4.0 g, 93%) as a yellow solid. ^1^H NMR (400 MHz, CDCl_3_) δ: 8.64 (1H, dd, *J* = 0.9, 7.4 Hz, H-9), 8.19 (1H, d, *J* = 8.0 Hz, H-7), 8.14 (1H, s, H-3), 8.02 (1H, d, *J* = 8.4, H-6), 7.74 (1H, dd, *J* = 8.0, 7.6 Hz, H-8), 7.67 (1H, d, *J* = 7.0 Hz, H-4), 7.56 (1H, dd, *J* = 8.0, 7.2 Hz, H-5). ^13^C NMR (100 MHz, CDCl_3_) δ: 178.7 (C=O), 143.1 (CH, C-3), 135.6 (CH), 132.5 (CH), 132.2 (CH), 132.2 (C), 131.5 (CH), 128.6 (C), 127.9 (C), 127.5 (CH), 126.9 (CH), 126.6 (C), 125.9 (C). UV-Vis (EtOH) λ_max_: 403, 362, 328 nm. Mp: 150–152 °C.

#### 3.2.2. Synthesis of 2-Iodo-1*H*-phenalen-1-one (**3**)

A solution of iodine (6.0 g, 20 mmol) in CCl_4_:Py (20 mL, 1:1) was added dropwise to a solution of 1*H*-phenalen-1-one (**1**) (2.0 g, 11.1 mmol) in CCl_4_:Py (20 mL, 1:1) at 0 °C under an argon atmosphere. The mixture was stirred at room temperature for 48 h. It was then diluted with ether (100 mL) and washed successively with water (50 mL), 1 N HCl solution (2 × 50 mL), water (50 mL), and an aqueous 20% Na_2_S_2_O_3_ solution (50 mL). The organic layer was dried over Na_2_SO_4_, filtered, and the solvent was evaporated to dryness under reduced pressure. The crude product was purified by recrystallization from ether to afford compound **3** (1.56 g, 46%) as a yellow solid. ^1^H NMR (400 MHz, CDCl_3_) δ: 8.55 (1H, dd, *J* = 1.2, 7.6 Hz, H-9), 8.37 (1H, s, H-3), 8.12 (1H, d, *J* = 7.2 Hz, H-7), 7.96 (1H, d, *J* = 8.4 Hz, H-6), 7.66 (1H, dd, *J* = 7.6, 8.0 Hz, H-8), 7.57 (1H, d, *J* = 6.8 Hz, H-4), 7.50 (1H, dd, *J* = 8, 7.2 Hz, H-5). ^13^C NMR (100 MHz, CDCl_3_) δ: 179.5 (C=O), 150.5 (CH, C-3), 135.4 (CH), 132.6 (CH), 132.4 (CH), 132.0 (C), 131.2 (C), 128.7 (C), 127.4 (CH), 127.0 (C), 126.9 (C), 126.8 (CH), 106.0 (C, C-2). EIMS: m/z 306 (M^+^, 15), 305 (M-1, 100), 179 (M-I^+^, 22), 151 (47), 111 (26), 97 (37), 57 (59). HRMS (EI): calcd. for C_13_H_7_IO (M^+^) 305.9542, found 305.9540.

#### 3.2.3. Synthesis of 1-Oxo-1*H*-phenalen-9-trifluoromethanesulfonate (**8**)

To a solution of 9-hydroxy-1*H*-phenalen-1-one (**1**) (400 mg, 2.04 mmol) in toluene (20 mL) was added 20 mL of a 30% K_3_PO_4_ solution (8.6 g, 40.8 mmol). The mixture was cooled to −10 °C, then triflic anhydride (0.42 mL, 2.45 mmol) was slowly added, and the reaction was stirred at room temperature for 5 h. The aqueous phase was extracted with toluene (3 × 15 mL), the combined organic layers were dried (MgSO_4_), filtered, and the solvent was removed under reduced pressure. The crude product was purified by silica gel column chromatography (hexane/EtOAc, 10–30%) to afford compound **8** (575 mg, 86%) as a yellow solid. ^1^H NMR (400 MHz, CDCl_3_) δ: 8.29 (1H, d, *J* = 8.8 Hz), 8.07 (1H, d, *J* = 8.2 Hz), 7.87 (1H, d, *J* = 7 Hz), 7.76 (1H, d, *J* = 9.8 Hz), 7.70 (1H, dd, *J* = 7.8, 7.5 Hz), 7.57 (1H, d, *J* = 8.8, Hz), 6.74 (1H, d, *J* = 9.8 Hz). EIMS: *m*/*z* 139 (100), 166 (31), 195 (35), 196 (16). 236 (32) 327 (73), 328 (M^+^, 14). HRMS (EI): calcd. for C_14_H_7_F_3_O_4_S (M^+^) 328.0017, found 327.0019. UV-Vis (EtOH) λ_max_: 307, 340, 355, 382 nm.

#### 3.2.4. Synthesis of 2-Aryl-1*H*-phenalen-1-one Derivatives: General Procedure

To a solution of 2-iodo-1*H*-phenalen-1-one (**3**) in a 1:1 DME/H_2_O mixture, Na_2_CO_3_, boronic acid, and 10% Pd/C were added. The reaction mixture was heated at 60 °C. After this time, EtOAc (10 mL) was added and the mixture was filtered over Celite^®^. H_2_O (20 mL) was then added, the mixture was extracted with EtOAc (3 × 8 mL), and dried (MgSO_4_). After filtration and concentration under vacuum, the crude product was purified by column chromatography or on a preparative silica gel plate.

**Synthesis of 2-phenyl-1*H*-phenalen-1-one (4)**.

From 2-iodo-1H-phenalen-1-one (**3**) (51.8 mg, 0.169 mmol), DME/H_2_O (1 mL), Na_2_CO_3_ (41.6 mg, 0.392 mmol), phenylboronic acid (46.1 mg, 0.378 mmol), and 10% Pd/C (15.6 mg), the mixture was heated for 10 min; column chromatography (CH_2_Cl_2_); product **4** (42.3 mg, 97%) as a yellow solid. ^1^H NMR (400 MHz, CDCl_3_) δ: 8.71 (1H, d, *J* = 7.4 Hz, H-9), 8.20 (1H, d, *J* = 8.0 Hz), 8.01 (1H, d, *J* = 8.3 Hz), 7.85 (1H, s, H-3), 7.85–7.78 (2H, m), 7.69 (2H, d, *J* = 8.0 Hz), 7.61 (1H, t, *J* = 7.6), 7.49–7.45 (2H, m), 7.42–7.38 (1H, m). ^13^C NMR (100 MHz, CDCl_3_) δ: 184.1 (C=O), 139.7 (CH), 139.4 (C), 136.7 (C), 134.7 (CH), 132.1 (C), 131.5 (CH), 131.1 (CH), 130.0 (C), 129.2 (2 × CH), 128.3 (2 × CH), 128.3 (C), 128.2 (CH), 127.4 (CH), 127.3 (C), 126.9 (CH, C-3). EIMS: *m*/*z* 301 (40), 280 (23), 279 (M^+^ + Na, 100). HRMS (ESI): calcd. for C_19_H_12_ONa (M^+^ + Na) 279.0787, found 279.0787. Mp: 147–149 °C.

**Synthesis of 4-(1-oxo-1*H*-phenalen-2-yl)benzonitrile (5)**.

Starting with 2-iodo-1*H*-phenalen-1-one (**3**) (70.0 mg, 0.228 mmol), DME/H_2_O (1.4 mL), Na_2_CO_3_ (56.0 mg, 0.524 mmol), (4-cyanophenyl)boronic acid (77.0 mg, 0.524 mmol), and 10% Pd/C (15.6 mg), the mixture was heated for 1 h and 20 min; column chromatography (hexane/EtOAc, 4:1 to 3:2) yielded product **5** (48.0 mg, 75%) as a yellow solid. ^1^H NMR (500 MHz, CDCl_3_) δ: 8.73 (1H, dd, *J* = 1.1, 7.4 Hz, H-9), 8.27 (1H, dd, *J* = 0.9, 8.0 Hz, H-7), 8.09 (1H, d, *J* = 8.2 Hz), 7.91 (1H, s, H-3), 7.88–7.85 (2H, m), 7.81 (2H, dd, *J* = 1.9, 6.0 Hz, H-2’, H-6’,), 7.74 (2H, dd, *J* = 2.0, 6.6 Hz, H-3’, H-5’), 7.66 (1H, dd, *J* = 7.6, 8.2 Hz, H-5). ^13^C NMR (125 MHz, CDCl_3_) δ: 183.4 (C=O), 141.4 (C, C-2), 140.9 (CH), 137.6 (C), 135.3 (CH), 132.5 (CH), 132.4 (CH), 132.2 (C), 132.1 (2 × CH), 131.5 (CH), 129.9 (2 × CH), 129.7 (C), 127.7 (CH), 127.6 (C), 127.4 (C), 127.1 (CH), 119.1 (CN), 111.7 (C, C-1’). EIMS: *m*/*z* 281 (M^+^, 56), 280 (10), 251 (11), 140 (11). HRMS (EI): calcd. for C_20_H_11_NO (M^+^) 281.0841, found 281.0834. UV-Vis (EtOH) λ_max_: 400, 363, 282, 249 nm. IR (film) ν_max_: 2852, 1731, 1634, 1115, 837 cm^−1^. Mp: 232–234 °C.

**Synthesis of 2-(4-fluorophenyl)-1*H*-phenalen-1-one (6)**.

Starting with 2-iodo-1*H*-phenalen-1-one (**3**) (70.0 mg, 0.228 mmol), DME/H_2_O (1.4 mL), Na_2_CO_3_ (56.0 mg, 0.524 mmol), (4-fluorophenyl)boronic acid (87.0 mg, 0.524 mmol), and 10% Pd/C (15.6 mg), the mixture was heated for 10 min; column chromatography (hexane/AcOEt, 4:1) gave product **6** (46.0 mg, 73%) as a yellow solid. ^1^H NMR (500 MHz, CDCl_3_) δ: 8.70 (1H, dd, *J* = 1.1, 7.3 Hz, H-9), 8.23 (1H, dd, *J* = 0.8, 8.0 Hz, H-7), 8.04 (1H, d, *J* = 8.1 Hz), 7.83 (1H, s, H-3), 7.81 (2H, t, *J* = 7.6 Hz), 7.66 (1H, dd, *J* = 2.1, 5.5 Hz), 7.62 (1H, dd, *J* = 7.1, 8.2 Hz, H-8), 7.14 (1H, dd, *J* = 2.1, 5.5 Hz). ^13^C NMR (125 MHz, CDCl_3_) δ: 184.1 (C=O), 162.9 (d, *J_C-F_* = 245.7 Hz), 139.6 (CH), 138.4 (C), 134.9 (CH), 132.6 (d, *J_C-F_* = 3.5 Hz), 132.2 (C), 131.7 (CH), 131.6 (CH), 131.2 (CH), 131.0 (d, *J_CH-F_* = 8.0 Hz, 2 × CHF), 129.9 (C), 128.1 (C), 127.5 (CH), 127.3 (C), 127.0 (CH), 115.3 (d, *J_CH-F_* = 21.2 Hz, 2 × CHF). ^19^F NMR (470 MHz, CDCl_3_) δ: −113.8. EIMS: *m*/*z* 274 (M^+^, 68), 273 (100), 244 (17), 136 (11). HRMS (EI): calcd. for C_19_H_11_FO (M^+^) 274.0794, found 274.0786. UV-Vis (EtOH) λ_max_: 405, 361, 254 nm. IR (film) ν_max_: 2850, 1631, 1577, 1288, 1220, 833cm^−1^. Mp: 200–202 °C.

**Synthesis of 2-(3-furyl)-1*H*-phenalen-1-one (7)**.

From 2-iodo-1*H*-phenalen-1-one (**3**) (50.0 mg, 0.163 mmol), DME/H_2_O (1 mL), Na_2_CO_3_ (34.6 mg, 0.326 mmol), 3-furylboronic acid (35.4 mg, 0.316 mmol), and 10% Pd/C (15.6 mg), the mixture was heated for 10 min; column chromatography (CH_2_Cl_2_) gave product **7** (39.5 mg, 98%) as a yellow solid. ^1^H NMR (400 MHz, CDCl_3_) δ: 8.68 (1H, d, *J* = 7.3 Hz, H-9), 8.56 (1H, s, H-2’), 8.18 (1H, d, *J* = 7.9 Hz, H-7), 7.97 (1H, d, *J* = 8.2 Hz), 7.87 (1H, s, H-3), 7.80–7.76 (2H, m), 7.58 (1H, t, *J* = 7.6 Hz, H-5), 7.50 (1H, s, H-5’), 6.84 (1H, s, H-4’). ^13^C NMR (100 MHz, CDCl_3_) δ: 183.8 (C=O), 143.8 (CH), 142.6 (CH), 135.9 (CH), 134.9 (CH), 132.0 (C), 131.3 (CH), 131.2 (CH), 131.0 (CH), 130.8 (C), 129.7 (C), 128.1 (C), 127.3 (CH), 126.9 (CH), 126.5 (s), 120.3 (C), 108.2 (CH). EIMS: *m*/*z* 270 (17), 269 (M^+^ + Na^+^,100). HRMS (ESI): calcd. for C_17_H_10_O_2_Na (M^+^ + Na^+^) 269.0578, found 269.0572. UV-Vis (EtOH) λ_max_: 407, 362, 246 nm. IR (film) ν_max_: 3393, 1754, 1636, 1573, 936 cm^−1^. Mp: 133–135 °C.

#### 3.2.5. Synthesis of 9-Phenyl-1*H*-phenalen-1-one (**9**)

To a solution of 1-oxo-1*H*-phenalen-9-trifluoromethylsulfonate (**8**) (46.0 mg, 0.140 mmol), K_3_PO_4_ (44.0 mg, 0.210 mmol), and Pd(PPh_3_)_4_ (10.5 mg, 0.009 mmol) in 1,4-dioxane (1.2 mL), 95% phenylboronic acid (28 mg, 0.229 mmol) was added, and the mixture was heated for 3 h at 60 °C. The reaction crude was concentrated on a rotary evaporator, H_2_O (10 mL) was added, extracted with EtOAc (3 × 8 mL), dried (Na_2_SO_4_), filtered and the solvent was removed on a rotary evaporator. The crude was purified by preparative silica gel plate chromatography (hexane/EtOAc, 3:2) to obtain product **9** (30 mg, 84%) as a red oil. ^1^H NMR (500 MHz, CDCl_3_) δ: 8.16 (1H, d, *J* = 8.3 Hz, H-7), 8.03 (1H, dd, *J* = 0.8, 8.2 Hz), 7.77 (1H, dd, *J* = 0.7, 7.0 Hz), 7.69 (1H, d, *J* = 9.7 Hz), 7.61 (1H, dd, *J* = 7.1, 8.2 Hz), 7.59 (1H, d, *J* = 8.2 Hz), 7.49–7.37 (5H, m), 6.60 (1H, d, *J* = 9.7 Hz).

**Synthesis of 4-(1-oxo-1*H*-phenalen-9-yl)benzonitrile (10)**.

To a solution of 1-oxo-1*H*-phenalen-9-trifluoromethylsulfonate (**8**) (50.0 mg, 0.152 mmol), K_3_PO_4_ (48 mg, 0.228 mmol), and Pd(PPh_3_)_4_ (10 mg, 0.009 mmol) in 1,4-dioxane (1.3 mL), (4-cyanophenyl)boronic acid (36 mg, 0.243 mmol) was added, and the mixture was heated for 2 h and 30 min at 60 °C. The reaction crude was concentrated on a rotary evaporator, H_2_O (10 mL) was added, the mixture was extracted with EtOAc (3 × 5 mL), dried (Na_2_SO_4_), filtered, and the solvent was removed on a rotary evaporator. The crude was purified by silica gel column chromatography (hexane/EtOAc, 4:1 to 3:2) to give product **10** (42 mg, 98%) as an orange solid. ^1^H NMR (500 MHz, CDCl_3_) δ: 8.22 (1H, d, *J* = 8.2 Hz, H-7), 8.07 (1H, dd, *J* = 0.7, 8.2 Hz), 7.82 (1H, d, *J* = 6.5 Hz), 7.74–7.72 (3H, m), 7.56 (1H, dd, *J* = 7.1, 8.2 Hz, H-5), 7.51 (1H, d, *J* = 8.2 Hz), 7.45 (2H, dd, *J* = 8.4, 1.9 Hz, H-2’, H-6’), 6.58 (1H, d, *J* = 9.7 Hz, H-2). ^13^C NMR (125 MHz, CDCl_3_) δ: 185.6 (C=O), 148.2 (C, C-9), 145.3 (C), 141.1 (CH), 134.3 (CH), 132.3 (C), 132.2 (2 × CH, C-2’, C-6’), 132.1 (CH), 132.0 (CH), 130.6 (CH), 130.2 (CH), 128.8 (2 × CH, C-3’, C-5’), 128.6 (C), 128.3 (C), 127.1 (CH), 126.1 (C), 119.2 (C, CN), 111.0 (C, C-1’). EIMS: *m*/*z* 281 (M^+^, 40), 280 (100), 251 (7), 140 (8) HRMS (EI): calcd. for C_20_H_11_NO (M^+^) 281.0841, found 281.0833. UV-Vis (EtOH) λ_max_: 361, 257 nm. IR (film) ν_max_: 2222, 1623, 1546, 832 cm^−1^. Mp: 191–193 °C.

**Synthesis of 9-(4-fluorophenyl)-1*H*-phenalen-1-one (11)**.

To a solution of 1-oxo-1*H*-phenalene-9-trifluoromethylsulfonate (**8**) (50.0 mg, 0.152 mmol), K_3_PO_4_ (48 mg, 0.228 mmol), and Pd(PPh_3_)_4_ (10 mg, 0.009 mmol) in 1,4-dioxane (1.3 mL), 95% (4-fluorophenyl)boronic acid (42 mg, 0.253 mmol) was added, and the mixture was heated for 1 h and 30 min at 60 °C. The reaction crude was concentrated on a rotary evaporator, H_2_O (8 mL) was added, the mixture was extracted with EtOAc (3 × 5 mL), dried (Na_2_SO_4_), filtered, and the solvent was removed on a rotary evaporator. The crude was purified by silica gel column chromatography (hexane/EtOAc, 4:1 to 3:2) to give product **11** (41.5 mg, 99%) as an orange solid. ^1^H NMR (500 MHz, CDCl_3_) δ: 8.16 (1H, d, *J* = 8.3 Hz, H-7), 8.03 (1H, dd, *J* = 0.95, 8.2 Hz), 7.76 (1H, dd, *J* = 0.8, 7.0 Hz), 7.68 (1H, d, *J* = 9.7 Hz, H-3), 7.61 (1H, dd, *J* = 7.1, 8.1 Hz, H-5), 7.55 (1H, d, *J* = 8.3, H-8), 7.35 (1H, d, *J* = 8.7 Hz, H-3’, H-5’), 7.34 (1H, d, *J* = 8.8 Hz), 7.16 (1H, d, *J* = 8.7, H-2’, H-6’), 7.14 (1H, d, *J* = 8.8 Hz), 6.59 (1H, d, *J* = 9.7 Hz, H-2). ^13^C NMR (125 MHz, CDCl_3_) δ: 185.9 (C=O), 163.3 (d, *J_C-F_* = 244.5 Hz), 146.6 (C), 140.6 (CH), 138.8 (d, *J_C-F_* = 3.6 Hz), 133.9 (CH), 131.9 (C), 131.9 (CH), 131.7 (2 × CH), 130.6 (CH), 129.8 (d, *J_CH-F_* = 8.0 Hz, 2 × CHF), 128.5 (C), 128.4 (C), 126.6 (CH), 126.2 (C), 115.4 (d, *J_CH-F_* = 21.7 Hz, 2 × CHF). ^19^F NMR (470 MHz, CDCl_3_) δ: −115.4. EIMS: *m*/*z* 274 (M^+^, 33), 273 (100), 136 (7), 113 (4). HRMS (EI): calcd. for C_19_H_11_FO (M^+^) 274.0794, found 274.0802. UV-Vis (EtOH) λ_max_: 398, 362, 252 nm. IR (film) ν_max_: 1624, 1552, 1350, 1211, 829 cm^−1^. Mp: 136–138 °C.

**Synthesis of 9-(4-methoxyphenyl)-1*H*-phenalen-1-one (12)**.

To a solution of 1-oxo-1*H*-phenalene-9-trifluoromethylsulfonate (**8**) (28.0 mg, 0.085 mmol), K_3_PO_4_ (27 mg, 0.128 mmol), and Pd(PPh_3_)_4_ (6 mg, 0.005 mmol) in 1,4-dioxane (0.7 mL) was added 95% (4-methoxyphenyl)boronic acid (33 mg, 0.217 mmol) and heated for 1 h and 30 min at 60 °C under an inert atmosphere. The reaction crude was concentrated on a rotary evaporator, H_2_O (10 mL) was added, the mixture was extracted with EtOAc (3 × 5 mL), dried (Na_2_SO_4_), filtered, and the solvent was removed on a rotary evaporator. The crude was purified by silica gel column chromatography (hexane/EtOAc, 10–30%) to give product **12** (20.7 mg, 85%) as an orange solid. ^1^H NMR (400 MHz, CDCl_3_) δ: 8.14 (1H, d, *J* = 8.3 Hz, H-7), 8.07 (1H, d, *J* = 8.2 Hz, H-6), 7.77 (1H, d, *J* = 7 Hz, H-4), 7.68 (1H, d, *J* = 9.7 Hz, H-3), 7.68 (2H, m, H-5, H-8), 7.34 (2H, d, *J* = 8.6, Hz, H-2’, H-6’), 7.0 (2H, d, *J* = 8.6 Hz, H-3’, H-5’), 6.60 (1H, d, *J* = 9.7 Hz, H-2), 3.88 (3H, s, OCH_3_). Mp: 175–178 °C.

**Synthesis of 9-(3-methoxyphenyl)-1*H*-phenalen-1-one (13)**.

To a solution of 1-oxo-1*H*-phenalene-9-trifluoromethylsulfonate (**8**) (80.0 mg, 0.244 mmol), K_3_PO_4_ (76 mg, 0.366 mmol), and Pd(PPh_3_)_4_ (17 mg, 0.015 mmol) in 1,4-dioxane (2 mL) was added (3-methoxyphenyl)boronic acid (59 mg, 0.390 mmol) and heated for 4 h at 60 °C. The reaction crude was concentrated on a rotary evaporator, H_2_O (8 mL) was added, the mixture was extracted with EtOAc (3 × 5 mL), dried (Na_2_SO_4_), filtered, and the solvent was removed on a rotary evaporator. The crude oil was purified by silica gel column chromatography (hexane/EtOAc, 19:1 to 17:3) to give product **13** (62 m, 89%) as an orange oil. ^1^H NMR (500 MHz, CDCl_3_) δ: 8.16 (1H, d, *J* = 8.3 Hz, H-7), 8.04 (1H, dd, *J* = 0.9, 8.2 Hz), 7.78 (1H, d, *J* = 6.7 Hz), 7.68 (1H, d, *J* = 9.7 Hz, H-3), 7.61 (1H, dd, *J* = 7.1, 8.2 Hz, H-5), 7.60 (1H, d, *J* = 8.3, H-8), 7.37 (1H, t, *J* = 7.8 Hz, H-5), 6.95 (2H, dd, *J* = 2.1, 7.9 Hz, H-4’, H-6’), 6.90 (1H, m, H-2’), 6.59 (1H, d, *J* = 9.7 Hz, H-2), 3.83 (3H, s, CH_3_). ^13^C NMR (125 MHz, CDCl_3_) δ: 185.8 (C=O), 159.8 (C, C-3’), 147.5 (C), 144.5 (C), 140.5 (CH), 138.8 (CH), 131.9 (C), 131.8 (CH), 131.6 (CH), 131.5 (CH), 130.6 (CH), 129.4 (CH), 128.7 (C), 128.5 (C), 126.5 (CH), 126.3 (C), 120.4 (CH), 113.7 (CH), 112.7 (CH), 56.4 (CH_3_, OCH_3_). EIMS: *m*/*z* 286 (M^+^, 74), 285 (100), 271 (28), 256 (19), 255 (95), 242 (30), 213 (19). HRMS (EI): calcd. for C_20_H_14_O_2_ (M^+^) 286.0994, found 286.1003. UV-Vis (EtOH) λ_max_: 362, 253 nm. IR (film) ν_max_: 1640, 1558, 1240, 850 cm^−1^.

**Synthesis of 9-(2-methoxyphenyl)-1*H*-phenalen-1-one (14)**.

To a solution of 1-oxo-1*H*-phenalene-9-trifluoromethylsulfonate (**8**) (20.0 mg, 0.058 mmol), K_3_PO_4_ (18 mg, 0.086 mmol), and Pd(PPh_3_)_4_ (4 mg, 0.003 mmol) in 1,4-dioxane (0.5 mL) was added (2-methoxyphenyl)boronic acid (14 mg, 0.093 mmol) and heated for 4 h at 60 °C. The reaction crude was concentrated on a rotary evaporator, H_2_O (8 mL) was added, the mixture was extracted with EtOAc (3 × 5 mL), dried (Na_2_SO_4_), filtered, and the solvent was removed on a rotary evaporator. The crude oil was purified by preparative silica gel plate chromatography (CH_2_Cl_2_) to give product **14** (6 mg, 36%) as a yellow solid. ^1^H NMR (500 MHz, CDCl_3_) δ: 8.18 (1H, d, *J* = 8.2 Hz, H-7), 8.03 (1H, dd, *J* = 0.9, 8.2 Hz), 7.75 (1H, dd, *J* = 0.9, 7.0 Hz), 7.67 (1H, d, *J* = 9.7 Hz, H-3), 7.60 (1H, dd, *J* = 7.1, 8.2 Hz, H-5), 7.58 (1H, d, *J* = 8.2 Hz, H-8), 7.39 (1H, ddd, *J* = 1.7, 7.4, 8.2 Hz), 7.16 (1H, dd, *J* = 1.8, 7.5 Hz), 7.07 (1H, ddd, *J* = 0.9, 7.3, 8.3 Hz), 6.99 (1H, dd, *J* = 0.6, 8.2 Hz), 6.57 (1H, d, *J* = 9.7 Hz, H-2), 3.70 (3H, s, CH_3_). ^13^C NMR (125 MHz, CDCl_3_) δ: 185.8 (C=O), 156.6 (C, C-2’), 143.9 (C), 140.4 (CH), 133.9 (CH), 132.4 (C), 132.0 (C), 131.8 (CH), 131.7 (CH), 131.2 (CH), 130.4 (CH), 128.9 (CH), 128.8 (CH), 128.7 (C), 128.4 (C), 127.1 (C), 126.3 (CH), 121.1 (CH), 110.9 (CH), 56.2 (CH_3_, OCH_3_). Mp: 123–125 °C.

#### 3.2.6. Synthesis of 9-Arylamino-1*H*-phenalen-1-ones Derivatives

**Synthesis of 9-[(4-methoxyphenyl)amino]-1*H*-phenalen-1-one (15)**.

To a solution of 1-oxo-1*H*-phenalene-9-trifluromethylsulfonate (**8**) (25 mg, 0.076 mmol) in THF (0.65 mL) was added (4-methoxyphenyl)amine (21 mg, 0.173 mmol) and stirred at room temperature for 28 h. After that time, the THF was removed under high vacuum. The crude product was purified by silica gel column chromatography (hexane/EtOAc, 4:1, 7:3) to give product **15** (22 mg, 96%) as a red solid. ^1^H NMR (500 MHz, CDCl_3_) δ: 13.64 (1H, br s, NH), 7.88–7.85 (4H, m), 7.44 (1H, t, *J* = 7.6 Hz, H-5), 7.35 (1H, d, *J* = 9.3 Hz, H-8), 7.30 (2H, d, *J* = 8.5 Hz, H-3’, H-5’), 7.03 (1H, d, *J* = 9.4 Hz, H-2), 6.98 (2H, d, *J* = 8.6 Hz, H-2’, H-6’), 3.86 (3H, s, OCH_3_). Mp: 128–130 °C.

**Synthesis of 9-[(2,6-dimethylphenyl)amino]-1*H*-phenalen-1-one (16)**.

A solution of 1-oxo-1*H*-phenalene-9-trifluromethylsulfonate (**8**) (50 mg, 0.152 mmol) and 2,6-dimethylaniline (0.6 mL) was heated at 80 °C for 4 days. After that time, the amine was removed under high vacuum. The crude product was purified by silica gel column chromatography (hexane/EtOAc, 1:1 to 1:9) to obtain product **16** (39 mg, 86%) as a yellow solid. ^1^H NMR (500 MHz, CDCl_3_) δ: 13.38 (1H, br s, NH), 7.94 (4H, m), 7.51 (1H, t, *J* = 7.5 Hz, H-5), 7.26 (3H, m), 7.13 (1H, d, *J* = 9.4 Hz), 6.77 (1H, d, *J* = 9.2 Hz, H-2), 2.29 (6H, s, 2 × CH_3_). ^13^C NMR (125 MHz, CDCl_3_) δ: 185.1 (C=O), 155.5 (C, C-9), 138.9 (CH), 138.4 (CH), 136.2 (CH), 135.9 (C), 131.9 (CH), 131.5 (CH), 128.9 (CH), 128.7 (2 × CH), 128.5 (C), 127.6 (2 × C), 125.4 (C), 125.0 (C), 122.2 (CH), 115.7 (CH), 108.2 (C), 18.4 (2 × CH_3_). EIMS: *m*/*z* 299 (M^+^, 80), 298 (100), 141 (11). HRMS (EI): calcd. for C_21_H_17_NO (M^+^) 299.1310, found 299.1315. UV-Vis (EtOH) λ_max_: 468, 441, 355, 250 nm. IR (film) ν_max_: 3397, 1632, 1509, 1242, 852 cm^−1^. Mp: 205–207 °C.

**Synthesis of 9-[(4-chlorophenyl)amino]-1*H*-phenalen-1-one (17)**.

To a solution of 1-oxo-1*H*-phenalene-9-trifluoromethylsulfonate (**8**) (50 mg, 0.152 mmol) in THF (1 mL) was added (4-chlorophenyl)amino (54.5 mg, 0.427 mmol) and heated at 50 °C for 24 h. After that time, THF was removed under high vacuum. The crude product was purified by silica gel column chromatography (hexane/EtOAc, 9:1 to 4:1) to give product **17** (43 mg, 93%) as an orange solid. ^1^H NMR (500 MHz, CDCl_3_) δ: 13.73 (1H, br s, NH), 7.92 (1H, d, *J* = 9.3 Hz, H-7), 7.91–7.86 (3H, m), 7.46 (1H, t, *J* = 7.6 Hz, H-5), 7.42 (1H, d, *J* = 9.2, H-3), 7.40 (2H, d, *J* = 8.6 Hz, H-2’, H-6’), 7.31 (2H, d, *J* = 8.6 Hz, H-3’, H-5’), 7.00 (1H, d, *J* = 9.4 Hz, H-2). ^13^C NMR (125 MHz, CDCl_3_) δ: 185.1 (C=O), 153.6 (C, C-9), 139.3 (CH), 138.2 (CH), 137.2 (C), 132.2 (CH), 131.6 (CH), 131.5 (CH), 131.8 (C), 129.8 (2 × CH, C-2’, C-6’), 128.8 (CH), 128.3 (C), 126.2 (2 × CH, C-3’, C-5’), 125.6 (C), 125.4 (C), 122.7 (CH), 115.5 (CH), 109.1 (C). EIMS: *m*/*z* 307 (34), 305 (M^+^, 100), 270 (18), 241 (12), 120 (23), HRMS (EI): calcd. for C_19_H_12_ClNO (M^+^) 305.0607, found 305.0611. UV-Vis (EtOH) λ_max_: 469, 358, 283, 244 nm. IR (film) ν_max_: 3092, 1630, 1581, 1510, 1139, 830cm^−1^. Mp: 133–135 °C.

#### 3.2.7. Synthesis of 1-Oxo-1*H*-phenalene-9-carbonitrile (**18**) and Its Derivative (**19**)

**Synthesis of 1-oxo-1*H*-phenalene-9-carbonitrile (18)**.

To a solution of 1-oxo-1*H*-phenalene-9-trifluoromethylsulfonate (**8**) (200 mg, 0.609 mmol), CuI (60 mg, 0.318 mmol), and Pd(PPh_3_)_4_ (106 mg, 0.092 mmol) in DCM (4 mL), NaCN (60 mg, 1.223 mmol) was added, and the mixture was heated under reflux for 24 h. After this time, the mixture was filtered over Celite^®^, a saturated solution of NH_4_Cl (10 mL) was added, the mixture was extracted with CH_2_Cl_2_ (3 × 5 mL), dried (Na_2_SO_4_), filtered, and the solvent was removed using a rotary evaporator. The crude product was purified by column chromatography on silica gel (hexane/CH_2_Cl_2_, 9:1 to 2:3) to give product **18** (118 mg, 95%) as an orange solid. ^1^H NMR (500 MHz, CDCl_3_) δ: 8.28 (1H, d, *J* = 8.3 Hz, H-7), 8.06 (1H, d, *J* = 8.4 Hz, H-6), 8.02 (1H, d, *J* = 8.03 Hz, H-3), 7.84 (1H, d, *J* = 6.6 Hz, H-4), 7.75 (1H, dd, *J* = 8.7, 10, H-5), 7.73 (1H, d, *J* = 8.3 Hz, H-8), 6.79 (1H, d, *J* = 9.8 Hz, H-2). Mp: 172–175 °C.

**Synthesis of 1-oxo-1*H*-phenalene-9-carboxamide (19)**.

Concentrated H_2_SO_4_ (2 mL) was added to a solution of 1-oxo-1*H*-phenalene-9-carbonitrile (**18**) (30.0 mg, 0.146 mmol) in AcOH (2 mL) and heated for 1 h and 30 min at 40 °C. H_2_O (10 mL) was added to the reaction mixture, which was extracted with EtOAc (3 × 8 mL), washed with 1N NaOH solution until pH = 7–8, dried (Na_2_SO_4_), filtered, and the solvent was removed on a rotary evaporator. The crude oil was purified by preparative silica gel plate chromatography (hexane/CH_2_Cl_2_, 1:4) to give product **19** (18 mg, 55%) as a yellow solid. ^1^H NMR (500 MHz, CDCl_3_) δ: 8.25 (1H, d, *J* = 8.2 Hz, H-7), 8.04 (1H, d, *J* = 8.2 Hz, H-6), 7.81 (1H, d, *J* = 7.0 Hz, H-4), 7.74 (2H, d, *J* = 9.1 Hz, H-8, H-3), 7.65 (1H, dd, *J* = 7.1, 8.1 Hz, H-5), 6.71 (1H, d, *J* = 9.7, H-2), 5.84 (2H, s, NH_2_). ^13^C NMR (125 MHz, CDCl_3_) δ: 185.1 (C=O), 141.9 (C, CONH_2_), 139.9 (C, C-9), 135.2 (CH), 132.6 (CH), 132.5 (CH), 132.0 (2 × C), 129.0 (CH), 128.3 (C), 127.5 (2 × C), 126.5 (CH), 125.2 (C). EIMS: *m*/*z* 223 (M^+^, 100), 207 (80), 271 (28), 195 (35), 180 (30), 152 (49), 151 (68). HRMS (EI): calcd. for C_14_H_9_NO_2_ (M^+^) 223.0633, found 223.0638. UV-Vis (EtOH) λ_max_: 391, 359, 247 nm. IR (film) ν_max_: 3378, 3262, 1610, 1390, 1264, 847 cm^−1^. Mp: 242–244 °C.

#### 3.2.8. Synthesis of 1-Oxo-1*H*-phenalene-2-carbonitrile (**20**) and Its Derivative (**21**)

**Synthesis of 1-oxo-1*H*-phenalene-2-carbonitrile (20)**.

To a solution of 2-iodo-1*H*-phenalen-1-one (**3**) (100.0 mg, 0.327), anhydrous CuI (30 mg, 0.163 mmol), and Pd(PPh_3_)_4_ (57.0 mg, 0.049 mmol) in anhydrous DCM (2 mL), finely ground NaCN (32 mg, 0.654 mmol) was added and the mixture was refluxed for 5 h under an inert atmosphere. After this time, the mixture was filtered over Celite^®^, a saturated NH_4_Cl solution (10 mL) was added, the mixture was extracted with CH_2_Cl_2_ (3 × 5 mL), dried (Na_2_SO_4_), filtered, and the solvent was removed on a rotary evaporator. The crude oil was purified by silica gel column chromatography (hexane/CH_2_Cl_2_, 9:1 to 7:3) to obtain product **20** (45.0 mg, 67%) as a yellow solid. ^1^H NMR (500 MHz, CDCl_3_) δ: 8.73 (1H, dd, *J* = 1.2, 7.4 Hz, H-9), 8.32 (1H, dd, *J* = 2.1, 7.0 Hz, H-7), 8.32 (1H, s, H-3), 8.27 (1H, dd, *J* = 0.8, 8.3 Hz, H-6), 7.96 (1H, dd, *J* = 0.8, 7.1 Hz, H-4), 7.87 (1H, t, *J* = 7.2 Hz, H-8), 7.71 (1H, dd, *J* = 7.1, 8.2 Hz, H-5). ^13^C NMR (125 MHz, CDCl_3_) δ: 179.8 (C=O), 150.1 (CH), 136.4 (CH), 135.6 (CH), 134.7 (CH), 132.4 (CH), 132.3 (C), 128.4 (C), 128.1 (CH), 127.8 (C), 127.4 (C), 125.7 (C), 115.4 (C), 114.9 (C). EIMS: *m*/*z* 206 (15), 205 (M^+^, 100), 177 (50), 88 (9). HRMS (EI): calcd. for C_14_H_7_NO (M^+^) 205.0528, found 205.0530. UV-Vis (EtOH) λ_max_: 397, 365, 253 nm. IR (film) ν_max_: 2226, 1640, 1582, 1357, 1257, 845 cm^−1^. Mp: 225–227 °C.

**Synthesis of *N*-(1-oxo-1*H*-phenalene-2-yl)carboxamide (21)**.

To a solution of 1-oxo-1*H*-phenalene-2-carbonitrile (**20**) (34.0 mg, 0.165 mmol) in AcOH (2 mL) was added H_2_SO_4_ (2 mL) and heated for 2 days at 40 °C. The crude product was poured onto an ice-water mixture, and the precipitate was filtered, washed with water and 5% Na_2_CO_3_ solution until the filtrate reached a pH = 6–7, to obtain product **21** (36.0 mg, 98%) as a yellow solid. ^1^H NMR (500 MHz, CDCl_3_) δ: 9.24 (1H, br s, NH), 8.97 (1H, s, H-3), 8.71 (1H, d, *J* = 7.4 Hz, H-9), 8.26 (1H, d, *J* = 7.9 Hz, H-7), 8.16 (1H, d, *J* = 8.3 Hz), 8.02 (1H, d, *J* = 7.0 Hz), 7.83 (1H, dd, *J* = 7.6, 7.8 Hz, H-5), 7.67 (1H, dd, *J* = 7.5, 7.8 Hz), 5.98 (1H, br s, NH). ^13^C NMR (125 MHz, CDCl_3_) δ: 184.7 (C=O), 165.7 (C), 148.6 (CH), 135.9 (CH), 135.5 (CH), 134.6 (CH), 132.1 (CH), 130.0 (C), 129.9 (C), 128.3 (C), 127.8 (C), 127.7 (CH), 127.3 (CH), 126.4 (C). EIMS: *m*/*z* 223 (M^+^, 100), 207 (52), 180 (61), 151 (50). HRMS (EI): calcd. for C_14_H_9_NO_2_ (M^+^) 223.0633, found 223.0633. UV-Vis (EtOH) λ_max_: 390, 252 nm. IR (film) ν_max_: 3347, 1695, 1574, 697cm^−1^. Mp: 186–188 °C.

#### 3.2.9. Synthesis of 2-Arylamino-1*H*-phenalen-1-one Derivatives: General Procedure

Arylamine was added to a solution of 2-bromo-1*H*-phenalen-1-one (**2**), NaO^t^Bu, Pd(PPh_3_)_4_ in anhydrous toluene. The reaction mixture was heated to 80 °C with stirring for 2 h–4 days in the dark under an argon atmosphere. After this time, CH_2_Cl_2_ (10 mL) was added and the mixture was filtered over Celite^®^. The mixture was then concentrated under vacuum to dryness on a rotary evaporator, and the crude product was purified by column chromatography or preparative plate chromatography on silica gel.

**Synthesis of 2-[(4-chloroanilino]-1*H*-phenalen-1-one (22)**.

From 2-bromo-1H-phenalen-1-one (**2**) (101.2 mg, 0.391 mmol), NaO^t^Bu (53.8 mg, 0.560 mmol), Pd(PPh_3_)_4_ (46 mg, 0.04 mmol), anhydrous toluene (1.5 mL) and 4-chloroaniline (63.7 mg, 0.5 mmol); it was heated at 80 °C for 17 h; column chromatography (hexane/EtOAc, 95:5); product **22** (56.4 mg, 47%) was obtained as a dark red solid. ^1^H NMR (400 MHz, CDCl_3_) δ: 8.75 (1H, dd, *J* = 0.8, 7.2 Hz, H-9), 8.24 (1H, d, *J* = 8 Hz, H-7), 7.85 (1H, d, *J* = 7.7 Hz), 7.80 (1H, dd, *J* = 7.6, 8.0 Hz), 7.60–7.53 (2H, m), 7.36 (2H, d, *J* =8.8 Hz, H-3’, H-5’), 7.28–7.23 (4H, m, H-3, H-2’, H-6’, NH). ^13^C HMR (100 MHz, CDCl_3_) δ: 180.28 (C=O), 139.56 (C, C-1’), 137.51 (C, C-2), 136.21 (CH), 132.06 (C), 131.11 (CH), 129.64 (2 × CH, C-3’, C-5’), 129.08 (C), 128.85 (CH), 128.45 (CH), 127.92 (C), 127.46 (C), 127.37 (CH), 126.87 (CH), 124.16 (C), 121.64 (2 × CH, C-2’, C-6’), 109.80 (C, C-3). EIMS: *m*/*z* 307 (34), 306 (23), 305 (M^+^, 100), 135 (25), 68 (20). HRMS (EI): calcd. for C_19_H_12_ClNO (M^+^) 305.0607, found 305.0601. Mp: 182–183 °C.

**Synthesis of 2-(4-methoxyanilino)-1*H*-phenalen-1-one (23)**.

From 2-bromo-1*H*-phenalen-1-one (**2**) (105.8 mg, 0.408 mmol), NaO^t^Bu (51.9 mg, 0.540 mmol), Pd(PPh_3_)_4_ (45.8 mg, 0.039 mmol), anhydrous toluene (1.5 mL) and 4-methoxyaniline (61.3 mg, 0.498 mmol); it was heated at 80 °C for 17 h; column chromatography (toluene/EOAc, 95:5); product **23** (77.0 mg, 63%) was obtained as a dark red solid. ^1^H NMR (400 MHz, CDCl_3_) δ: 8.72 (1H, dd, *J* = 1.0, 7.4 Hz, H-9), 8.19 (1H, d, *J* = 8 Hz, H-7), 7.76–7.73 (2H, m), 7.49–7.48 (2H, m), 7.24 (2H, d, *J* = 8.8 Hz, H-3’, H-5’), 7.05 (1H, s, H-3), 7.0 (1H, br s, NH), 6.97 (2H, dd, *J* =1.9, 8.8 Hz, H-2’, H-6’), 3.83 (3H, s, OCH_3_). ^13^C NMR (100 MHz, CDCl_3_) δ: 180.6 (C=O), 155.9 (C, C-4’), 139.2 (C), 135.9 (CH), 133.7 (C), 132.0 (C), 130.8 (CH), 129.6 (C), 128.1 (CH), 128.0 (C), 127.7 (CH), 127.3 (CH), 126.7 (CH), 123.9 (C), 123.6 (2 × CH, C-3’, C-5’), 114.8 (2 × CH, C-2’, C-6’), 107.7 (CH, C-3), 55.69 (CH_3_, OCH_3_). EIMS: *m*/*z* 302 (22), 301 (M^+^, 100), 286 (44). HRMS (EI): calcd. for C_20_H_15_NO_2_ (M^+^) 301.1103, found 301.1092. Mp: 137–139 °C.

**Synthesis of 2-((2,6-dimethylphenyl)amino)-2,3-dihydro-1*H*-phenalen-1-one (24)**.

From 2-bromo-1*H*-phenalen-1-one (**2**) (104.9 mg, 0.405 mmol), NaO^t^Bu (53.3 mg, 0.555 mmol), Pd(PPh_3_)_4_ (47.5 mg, 0.041 mmol), anhydrous toluene (1.5 mL) and 2,6-dimethylaniline (58 μL, 0.463 mmol); it was heated at 80 °C for 4 days; column chromatography (toluene/EOAc, 95:5); product **24** (32.3 mg, 28%) was obtained as a red oil. ^1^H NMR (400 MHz, CDCl_3_) δ: 8.78 (1H, dd, *J* = 0.7, 7.2 Hz, H-9), 8.21 (1H, d, *J* = 8 Hz, H-7), 7.79 (1H, t, *J* = 7.6 Hz, H-5), 7.76 (1H, d, *J* = 7.5 Hz, H-6), 7.47 (1H, dd, *J* = 7.3, 8.0 Hz, H-8), 7.41 (1H, d, *J* = 7.0 Hz, H-4), 7.19 (3H, br s, H-3’, H-4’, H-5’), 6.61 (1H, br s, NH), 6.12 (1H, s, H-3), 2.28 (6H, s, CH_3_). ^13^C NMR (100 MHz, CDCl_3_) δ: 180.5 (C=O), 139.8 (C, C-1’), 136.9 (C, C-2), 136.4 (CH), 135.9 (CH), 132.1 (C), 130.7 (CH), 129.9 (C), 128.7 (CH, 2 × C, C-3’, C-5’), 128.3 (C), 127.9 (CH), 127.5 (CH), 127.3 (CH), 126.7 (C, CH, 2 × C), 123.9 (C), 107.5 (CH, C-3), 18.4 (CH_3_, 2 × C). EIMS: *m*/*z* 299 (M^+^, 100), 282 (44), 141 (29). HRMS (EI): calcd. for C_21_H_17_NO (M^+^) 299.1310, found 299.1310.

**Synthesis of 2-(pyrimidin-2-ylamino)-1*H*-phenalen-1-one (25)**.

From 2-bromo-1*H*-phenalen-1-one (**2**) (50.7 mg, 0.196 mmol), NaO^t^Bu (28.1 mg, 0.292 mmol), Pd(PPh_3_)_4_ (26.2 mg, 0.023 mmol), anhydrous toluene (0.75 mL) and pyrimidin-2-amine (25.6 mg, 0.269 mmol); it was heated at 80 °C for 3 h; column chromatography (hexane/EtOAc, 3:2); product **25** (17.2 mg, 33%) was obtained as a red solid. ^1^H NMR (400 MHz, CDCl_3_) δ: 8.86 (1H, s, H-3), 8.73 (1H, dd, *J* = 1.0, 7.4 Hz, H-9), 8.58 (1H, br s, NH), 8.53 (2H, d, *J* = 4.8 Hz, H-4’, H-6’), 8.21 (1H, dd, *J* = 0.6, 8.0 Hz, H-7), 7.88 (1H, d, *J* = 8.2 Hz), 7.79–7.74 (2H, m), 7.57 (1H, dd, *J* = 7.3, 8.0 Hz), 6.79 (1H, t, *J* = 4.8 Hz, H-5’). ^13^C NMR (100 MHz, CDCl_3_) δ: 179.7 (C=O), 159.9 (CH, C-2’), 158.0 (2 × CH, C-4’, C-6’), 135.9 (CH), 134.4 (C, C-2’), 132.0 (C), 131.1 (CH), 130.7 (CH), 129.4 (CH), 128.9 (C), 127.8 (C), 127.3 (CH), 126.8 (CH), 124.7 (C), 119.1 (CH), 113.3 (CH, C-3). EIMS: *m*/*z* 274 (18), 273 (M^+^, 100), 272 (54), 244 (14). HRMS (EI): calcd. for C_17_H_11_N_3_O (M^+^) 273.0902, found 273.0893. UV-Vis (EtOH) λ_max_: 457, 365, 282 nm. IR (film) ν_max_: 3349, 1622, 1564, 1519, 1395, 842 cm^−1^. Mp: 181–183 °C.

**Synthesis of naphtho[1,8-*ab*]phenoxazine (26)**.

From 2-bromo-1*H*-phenalen-1-one (**2**) (105.2 mg, 0.406 mmol), NaO^t^Bu (107.6 mg, 1.12 mmol), Pd(PPh_3_)_4_ (88 mg, 0.078 mmol), anhydrous toluene (1.5 mL) and 2-aminophenol (56.1 mg, 0.514 mmol); it was heated at 80 °C for 4 days; column chromatography (toluene/EtOAc, 9.5:0.5); product **26** (11.3 mg, 10%) was obtained as a red solid. ^1^H NMR (500 MHz, CDCl_3_) δ: 8.71 (1H, d, *J* = 7.5 Hz, H-1), 7.92 (1H, d, *J* = 8.0 Hz, H-3), 7.59–7.62 (2H, m, H-2, H-4), 7.38 (1H, dd, *J* = 1.2, 7.7 Hz), 7.35 (1H, t, *J* = 7.3 Hz, H-5), 7.24 (1H, m, H-6), 7.12 (1H, m), 7.03 (1H, m), 6.87 (1H, dd, *J* = 0.9, 7.9 Hz), 6.46 (1H, s, H-7). ^13^C NMR (125 MHz, CDCl_3_) δ: 152.7 (C, C-13a), 146.1 (C), 145.1 (C, C-7a), 135.0 (C), 133.3 (C, C-3a), 132.3 (CH, C-3), 131.4 (C, C-6a), 130.4 (C, C-13b), 128.8 (CH), 128.2 (CH), 127.1 (2 × CH), 127.0 (C, C-13c), 126.9 (CH), 125.5 (CH, C-1), 125.2 (CH, C-6), 124.1 (CH), 114.8 (CH), 108.5 (CH, C-7). EIMS: *m*/*z* 270 (M^+^+1, 21) 269 (M^+^, 100), 240 (13), 134 (18). HRMS (EI): calcd. for C_19_H_11_NO (M^+^) 269.0841, found 269.0842. UV-Vis (EtOH) λ_max_: 524, 491, 269, 237 nm. IR (film) ν_max_: 1732, 1281, 824 cm^−1^. Mp: 168–170 °C.

**Synthesis of 7*H*-naphtho[1,8-*bc*]acridin-7-one (27)**.

From 2-bromo-1*H*-phenalen-1-one (**2**) (51.5 mg, 0.168 mmol), NaO^t^Bu (23.0 mg, 0.239 mmol), Pd(PPh_3_)_4_ (19.6 mg, 0.017 mmol), anhydrous toluene (0.75 mL) and (2-aminophenyl)methanol (24.2 mg, 0.196 mmol); it was heated at 80 °C for 20 h; preparative plate chromatography (toluene/EtOAc, 9.8:0.2); product **27** (6.0 mg, 12%) was obtained as a yellow solid. ^1^H NMR (500 MHz, CDCl_3_) δ: 9.36 (1H, dd, *J* = 1.1, 7.3 Hz, H-1), 9.28 (1H, s, H-8), 8.79 (1H, d, *J* = 1.3, 7.3 Hz, H-6), 8.29 (1H, dd, *J* = 1.1, 8.1 Hz, H-4), 8.25 (1H, dd, *J* = 0.7, 8.5 Hz, H-12), 8.14 (1H, dd, *J* = 0.9, 8.1 Hz, H-3), 8.06 (1H, m, *J* = 7.0 Hz, H-9), 7.87 (1H, ddd, *J* = 1.5, 6.8, 8.4 Hz, H-11), 7.83 (1H, dd, *J* = 7.6, 7.9 Hz, H-2), 7.81 (1H, dd, *J* = 7.4, 7.9 Hz, H-5), 7.61 (1H, ddd, *J* = 1.1, 6.8, 8.0 Hz, H-10). ^13^C NMR (125 MHz, CDCl_3_) δ: 184.2 (C, C-7), 152.4 (C, C-13a), 150.4 (C, C-12a), 137.8 (CH, C-8), 135.6 (CH, C-4), 133.2 (C, C-6a), 132.4 (CH, C-11), 131.7 (CH, C-3), 129.9 (CH), 129.8 (CH), 129.7 (CH, C-9), 129.6 (C, C-13c), 128.2 (2 × C, C-3a, C-13b), 127.6 (C, 8a), 127.3 (CH, C-1), 127.2 (CH, C-2), 127.2 (CH, C-10), 126.5 (CH, C-5), 125.1 (C, C-7a). EIMS: *m*/*z* 311 (15), 305 (30), 304 (M^+^ + Na, 100), 301 (50). HRMS (ESI): calcd. for C_20_H_11_NO (M^+^ + Na) 304.0738, found 304.0743. Mp: 221–224 °C.

#### 3.2.10. Synthesis of 9-Hydroxy-1*H*-phenalen-1-one Derivatives

**Synthesis of 2,7-dimethoxynaphthalene (29)**.

Diazomethyl(trimethyl)silane (2.03 mL, 4.018 mmol) was slowly added to a solution of 7-methoxynaphthalene-2-ol (**28**) (100.0 mg, 0.574 mmol) in MeOH (2 mL) and stirred for 6 h at room temperature. The resulting precipitate was gravity filtered to give product **29** (95.2 mg, 88%) as a white solid. ^1^H NMR (400 MHz, CDCl_3_) δ: 7.68 (2H, d, *J* = 8.8 Hz, H-4, H-5), 7.09 (2H, s, H-1, H-8), 7.03 (2H, d, *J* = 2.0, 8.8 Hz, H-3, H-6), 3.92 (6H, s, OCH_3_). EIMS: *m*/*z* 189 (21), 145 (51), 188 (M^+^, 100). HRMS (EI): calcd. for C_12_H_12_O_2_ (M^+^) 188.0737, found 188.0754. Mp: 135–137 °C.

**Synthesis of 4,9-dihydroxy-1*H*-phenalen-1-one (30)**.

To a solution of 2,7-dimethoxynaphthalene (**29**) (30.0 mg, 0.159 mmol) and (2*E*)-3-phenylacryloyl chloride (27.0 g, 159 mmol) in DCE (1 mL) was added AlCl_3_ (48 mg, 0.318 mmol) portionwise, and the mixture was irradiated with MW for 60 min at 130 °C. Additional DCE (1 mL) and AlCl_3_ (25 mg, 0.159 mmol) were then added, and the mixture was irradiated with MW for 30 min at 130 °C. The reaction crude was concentrated in a rotary evaporator, cooled in an ice bath, and a cold 1N HCl solution (1 mL) was added. The aqueous phase was extracted with EtOAc (3 × 15 mL). The organic phase was washed with a 1% NaHCO_3_ solution (3 × 15 mL) and with H_2_O (10 mL). On the other hand, the aqueous phase was acidified to pH = 2–3 and extracted with EtOAc (3 × 15 mL). The organic phase was dried (MgSO_4_), filtered, and the solvent was removed in a rotary evaporator. The crude was purified by silica gel column chromatography (hexane/EtOAc, 50–70%, EtOAc, acetone) to obtain product **30** (24.5 mg, 73%) as a yellow solid. ^1^H NMR (400 MHz, (CD_3_)_2_CO) δ: 10.57 (1H, br s, OH), 8.62 (1H, d, *J* = 9.6 Hz, H-3), 8.19 (1H, d, *J* = 9.2 Hz), 8.09 (1H, d, *J* = 8.4 Hz), 7.25 (1H, d, *J* = 8.4 Hz), 7.08 (1H, d, *J* = 9.6 Hz, H-2), 6.99 (1H, d, *J* = 9.2 Hz), 3.20 (br s, OH). EIMS: *m*/*z* 212 (M^+^, 100), 105 (89), 51 (15). HRMS (EI): calcd. for C_13_H_8_O_3_ (M^+^) 212.0473, found 212.0468. Mp: 240 °C.

**Synthesis of ethyl 3-hydroxynaphthalene-2-carboxylate (32)**.

Concentrated H_2_SO_4_ (0.1 mL) was added to a solution of 3-hydroxy-2-naphthoic acid (**31**) (1 g, 5.3 mmol) in EtOH (10 mL). The reaction mixture was heated under reflux for 24 h. The solvent was then removed, dissolved in a hexane/EtOAc mixture (10 mL, 2:1), and washed with H_2_O, saturated NaHCO_3_ solution, and saturated NaCl solution. The mixture was then dried (MgSO_4_), filtered, and concentrated under vacuum to give **32** (800 mg, 70%) as a white solid. ^1^H NMR (400 MHz, CDCl_3_) δ: 11.92 (1H, br s, OH), 10.87(1H, s, H-1), 9.09 (1H, d, *J* = 8.6 Hz), 8.58 (1H, s, H-4), 7.76 (2H, dt, *J* = 0.4, 6.5 Hz), 7.67–7.64 (1H, cd, *J* = 1.1, 5.5 Hz), 7.41–7.38 (1H, cd, *J* = 0.8, 5.5 Hz), 4.49 (2H, c, *J* = 7.1 Hz, CH_2_), 1.48 (3H, t, *J* = 7.1 Hz, CH_3_). EIMS: *m*/*z* 216 (M^+^, 45), 171 (20), 169 (100), 142 (45), 114 (19). HRMS (EI): calcd. for C_13_H_12_O_3_ (M^+^) 216.0786, found 216. 0782. Mp: 85–88 °C.

**Synthesis of ethyl 9-hydroxy-1-oxo-1*H*-phenalene-8-carboxylate (33)**.

To a solution of ethyl 3-hydroxynaphthalene-2-carboxylate (**32**) (101 mg, 0.463 mmol) and (2*E*)-3-phenylacryloyl chloride (77.0 g, 463 mmol) in DCE (2 mL) was added AlCl_3_ (123 mg, 0.926 mmol) slowly, and the mixture was stirred at room temperature for 1 h. After this time, the mixture was heated under reflux for 2 h. The reaction mixture was then cooled and DCE (1 mL) and AlCl_3_ (62 mg, 0.463 mmol) were added, followed by reflux for 4 h. The crude product was concentrated on a rotary evaporator, cooled in an ice bath, and cold 1N HCl (1 mL) was added. The mixture was then lyophilized, the solid dissolved, and filtered with hot EtOAc. The crude product was purified by silica gel column chromatography (hexane/EtOAc, 10–80%) to yield 3 products: ethyl 4-hydroxy-3-oxo-1-phenyl-2,3-dihydro-1*H*-phenalene-5-carboxylate (7.1 mg, 4%), Ethyl 9-hydroxy-1-oxo-3-phenyl-1*H*-phenalene-8-carboxylate (2.1 mg, 1.3%), and product **33** (33 mg, 33%) as orange oils. Ethyl 4-hydroxy-3-oxo-1-phenyl-2,3-dihydro-1*H*-phenalene-5-carboxylate: ^1^H NMR (500 MHz, CDCl_3_) δ: 13.98 (1H, s, OH), 8.65 (1H, s, H-6), 7.77 (1H, d, *J* = 7.9 Hz), 7.36–7.27 (5H, m), 7.16 (2H, d, *J* = 7.2 Hz), 4.66 (1H, t, *J* = 7.2 Hz, H-1), 4.48 (2H, c, *J* = 7.1, Hz, H-1’), 3.28 (2H, d, *J* = 6.8 Hz, H-2), 1.46 (3H, t, *J* = 7.1 Hz, H-2’). ^13^C NMR (125 MHz, CDCl_3_) δ: 202.8 (C), 165.1 (C), 161.6 (C), 142.5 (C), 141.6 (CH), 134.11 (C), 133.3 (C), 130.0 (CH), 129.1 (2 × CH), 128.5 (CH), 127.9 (2 × CH), 127.5 (CH), 126.1 (C), 124.9 (CH), 121.4 (C), 111.2 (C), 61.7 (CH_2_), 44.91 (CH), 44.5 (CH_2_), 14.5 (CH_3_). EIMS: *m*/*z* 346 (M^+^, 100), 301 (27), 300 (43), 224 (25), 215 (19), 196 (14), 139 (14). HRMS (EI): calcd. for C_22_H_18_O_4_ (M^+^) 346.1205, found 346.1203. UV-Vis (EtOH) λ_max_: 378, 328, 234 nm. IR (film) ν_max_: 2983, 1729, 1631, 1207, cm^−1^. Ethyl 9-hydroxy-1-oxo-3-phenyl-1*H*-phenalene-8-carboxylate: ^1^H NMR (400 MHz, CDCl_3_) δ: 8.82 (1H, s, H-7), 8.15 (2H, d, *J* = 7.9 Hz, H-6, H-4), 7.60 (1H, t, *J* = 7.8 Hz, H-5), 7.56–7.51 (5H, m, *J* = 7.24 Hz, phenyl), 4.51 (2H, c, *J* = 7.12 Hz, H-1’), 1.48 (3H, t, *J* = 7.12 Hz, H-2’). ^13^C NMR (125 MHz, CDCl_3_) δ: 179 0, 176.8, 164.9, 153.9, 145.7, 138.1, 135.0, 134.4, 129.8 (2 × C), 129.0, 128.7 (3 × C), 125.6, 125.2, 124.5, 124.4, 123.7, 111.6, 61.7 (C-1’), 14.6 (C-2’). EIMS: *m*/*z* 344 (M^+^, 60), 299 (33), 272 (100), 270 (20), 215 (20), 213 (21). HRMS (EI): calcd. for C_22_H_16_O_4_ (M^+^) 344.1049, found 344.1047. UV-Vis (EtOH) λ_max_: 450, 425, 356, 256 nm. IR (film) ν_max_: 2921, 1719, 1625, 1200, 1110, 1025 cm^−1^. Compound **33**: ^1^H NMR (500 MHz, CDCl_3_) δ: 8.72 (1H, s, H-7), 8.09 (1H, d, *J* = 9.2 Hz, H-3), 8.09–8.08 (2H, m), 7.62 (1H, t, *J* = 7.6 Hz, H-5), 7.20 (1H, d, *J* = 9.2 Hz, H-2), 4.48 (2H, c, *J* = 7.2 Hz, H-1’), 1.46 (3H, t, *J* = 7.1 Hz, H-2’). ^13^C NMR (125 MHz, CDCl_3_) δ: 178.8 (C), 177.7 (C), 164.8 (C), 145.5 (CH), 140.9 (CH), 135.0 (CH), 134 8 (H), 127.9 (C), 125.5 (2 × C), 124.6 (CH), 124.0 (C), 123.5 (CH), 111.7 (C), 61.6 (CH_2_), 14.5 (CH_3_). EIMS: *m*/*z* 268 (M^+^, 64), 223 (52), 222 (71), 196 (100), 194 (29), 139 (36), 138 (15). HRMS (EI): calcd. for C_16_H_12_O_4_ (M^+^) 268.0736, found 268.0742. UV-Vis (EtOH) λ_max_: 446, 421, 354, 252 nm. IR (film) ν_max_: 3333, 1630, 1001, 845 cm^−1^.

**Synthesis of 9-hydroxy-1-oxo-1*H*-phenalene-8-carboxylic acid (34)**.

To a solution of the **33** (2.31 g, 7.77 mmol) in MeOH (1mL) and H_2_O (0.2 mL) was added NaOH (4 mg, 0.1 mmol). The mixture was stirred at rt for 8 h. The mixture was concentrated and the crude was diluted with H_2_O (5 mL) and acidified with an aq solution of 2M HCl (1 mL). This aqueous mixture was extracted with EtOAc (2 × 5 mL) and the combined organic extracts were washed with brine (10 mL), dried with MgSO_4_, and concentrated under reduce pressure to provide the product **34** (13 mg, 74%) as a yellow solid. ^1^H NMR (500 MHz, CDCl_3_) δ: 14.87 (1H, s, OH), 13.5 (1H, br s, OH), 9.24 (1H, d, *J* = 1.8 Hz, H-7), 8.37 (1H, d, *J* = 9.1 Hz, H-3), 8.32 (2H, d, *J* = 8.0 Hz, H-6, H-4), 7.79 (1H, t, *J* = 7.7 Hz, H-5), 7.47 (1H, d, *J* = 9.1 Hz, H-2). ^13^C NMR (125 MHz, CDCl_3_) δ: 185.7 (C, COOH), 172.8 (C, C-1), 165.3 (C, C-9), 149.9 (CH), 142.5 (CH), 137.7 (CH), 137.1 (CH), 127.8 (C), 125.9 (C), 125.5 (CH), 124.1 (C), 122.8 (C), 121.2 (CH), 111.1 (C). EIMS: *m*/*z* 240 (M^+^, 49), 222 (22), 196 (100), 194 (25), 168 (25), 83 (13). HRMS (EI): calcd. for C_14_H_8_O_4_ (M^+^) 240.0423, found 240.0417. UV-Vis (EtOH) λ_max_: 443, 351, 236 nm. IR (film) ν_max_: 3452, 2401, 1738, 1628, 1575, 1405 cm^−1^. Mp: 242–244 °C.

#### 3.2.11. Synthesis of 4*H*-Benzo[*de*]isoquinolin-4-ones

**Synthesis of 4*H*-benzo[*de*]isoquinolin-4-one (35)**.

A mixture of 2-methoxynaphthalene (316 mg, 1.98 mmol) and triazine (180 mg, 2.22 mmol) in PPA (6–8 g) was stirred at 40–45 °C for 1 h and then at 100–110 °C for 3 h. The reaction mixture was poured into cold H_2_O (60 mL) with vigorous stirring. The resulting mixture was extracted with EtOAc (3 × 50 mL), dried (MgSO_4_), filtered, and the solvent was removed under vacuum. The residue was purified by column chromatography on silica gel (CH_2_Cl_2_) to give 2-methoxy-1,6-naphthalenedicarbaldehyde (38 mg, 9%) as a pale yellow solid. The aqueous phase was treated with ammonia until pH = 8–9 and, after cooling, the oil was extracted with EtOAc (3 × 50 mL). The organic phase was dried (MgSO_4_), filtered and the solvent was removed under vacuum and the residue was purified by column chromatography on silica gel (CH_2_Cl_2_) to give product **35** (41 mg, 11%) as a yellow solid. Compound **35**: ^1^H NMR (600 MHz, CDCl_3_) δ: 9.53 (1H, s), 9.52 (1H, s), 8.15 (1H, d, *J* = 8.4 Hz), 7.93 (1H, d, *J* = 7.2 Hz), 7.78 (1H, d, *J* = 9.6 Hz, H-5), 7.72 (1H, dd, *J* = 7.6, 7.7 Hz, H-8), 6.74 (1H, d, *J* = 10.2 Hz, H-6). ^13^C NMR (150 MHz, CDCl_3_) δ: 185.6 (C), 157.1 (CH), 147.4 (CH), 141.0 (CH), 134.0 (CH), 130.9 (CH), 130.6 (C), 130.4 (CH), 128.4 (CH), 127.4 (C), 127.0 (C), 122.6 (C). EIMS: *m*/*z* 181 (M^+^, 100), 153 (58), 126 (15), 76 (15), 636 (17). HRMS (EI): calcd. for C_12_H_7_NO (M^+^) 181.0528, found 181.0520. UV-Vis (EtOH) λ_max_: 383, 340, 324, 252, 245, 232 nm. IR (film) ν_max_: 1638, 1574, 515, 495, 470 cm^−1^. Mp: 170–172 °C. 2-Methoxy-1,6-naphthalenedicarbaldehyde: ^1^H NMR (400 MHz, CDCl_3_) δ: 10.81 (1H, s), 10.05 (1H, s), 9.30 (1H, d, *J* = 8.9 Hz, H-8), 8.20 (1H, d, *J* = 1.2 Hz, H-5), 8.14 (1H, d, *J* = 9.2 Hz), 8.0 (1H, dd, *J* = 1.6, 8.9 Hz, H-7), 7.35 (1H, d, *J* = 9.8 Hz), 4.04 (3H, s). EIMS: *m*/*z* 214 (M^+^, 100), 213 (47), 185 (16), 154 (18). HRMS (EI): calcd. for C_13_H_10_O_3_ (M^+^) 214.0630, found 214.0620. IR (film) ν_max_: 2873, 1660, 1179, 1057, 506, 490 cm^−1^. Mp: 156–159 °C.

**Synthesis of 9-methoxy-4*H*-benzo[*de*]isoquinolin-4-one (36)**.

A mixture of 2,7-dimethoxynaphthalene (**29**) (100 mg, 0.53 mmol) and triazine (47 mg, 0.58 mmol) in APP (3–4 g) was stirred at 40–45 °C for 1 h and then at 70–90 °C for 3 h. The reaction mixture was poured into cold H_2_O (20 mL) with vigorous stirring. The resulting mixture was extracted with EtOAc (3 × 25 mL), dried (MgSO_4_), filtered, and the solvent was removed under vacuum. The aqueous phase was treated with ammonia until pH = 8–9 and, after cooling, the oil was extracted with EtOAc (3 × 25 mL). The organic phase was dried (MgSO_4_), filtered and the solvent was removed under vacuum and the residue was purified by silica gel column chromatography (CH_2_Cl_2_), to give product **36** (90 mg, 80%) as a yellow solid. ^1^H NMR (500 MHz, CDCl_3_) δ: 9.75 (1H, s), 9.48 (1H, s), 7.75 (1H, d, *J* = 8.1 Hz), 7.62 (1H, d, *J* = 9.7 Hz, H-5), 6.87 (1H, d, *J* = 8.1 Hz), 6.56 (1H, d, *J* = 9.7 Hz, H-6), 4.09 (3H, s, CH_3_). ^13^C NMR (125 MHz, CDCl_3_) δ: 185.5 (C, C-4), 160.5 (C), 152.1 (CH), 148.1 (CH), 141.1 (CH), 136.2 (CH), 131.4 (C), 127.5 (CH), 122.3 (C), 122.3 (C), 119.8 (C), 119.7 (C), 106.1 (CH), 56.4 (CH_3_, OCH_3_). EIMS: *m*/*z* 212 (14), 211 (M^+^, 100), 168 (17), 140 (27). HRMS (EI): calcd. for C_13_H_9_NO_2_ (M^+^) 211.0633, found 211.0626. UV-Vis (EtOH) λ_max_: 431, 278, 256, 230 nm. IR (film) ν_max_: 3336, 1644, 1581, 1236, 837, 489 cm^−1^. Mp: 202–204 °C.

**Synthesis of ethyl 4-oxo-4*H*-benzo[*de*]isoquinoline-5-carboxylate (37)**.

A mixture of ethyl 3-hydroxy-2-naphthoate (**32**) (800 mg, 3.7 mmol) and triazine (329 mg, 4.1 mmol) in APP (8–9 g) was stirred at 40–45 °C for 1 h and then at 100–110 °C for 4 h. The reaction mixture was poured into cold H_2_O (80 mL) with vigorous stirring. The resulting mixture was extracted with EtOAc (3 × 60 mL), dried (MgSO_4_), filtered, and the solvent was removed under vacuum. The residue was purified by silica gel column chromatography (hexane/EtOAc, 1:2) to give ethyl 4-formyl-3-methoxy-2-naphthoate (**38**) (196 mg, 22%) as a pale yellow solid. The aqueous phase was treated with ammonia until pH = 8–9 and, after cooling, the oil was extracted with EtOAc (3 × 60 mL). The organic phase was dried (MgSO_4_), filtered and the solvent was removed under vacuum. The residue was purified by silica gel column chromatography (hexane/EtOAc, 1:2) to give product **37** (100 mg, 12%) as an orange oil. Compound **37**: ^1^H NMR (500 MHz, CDCl_3_) δ: 9.58 (1H, s), 9.57 (1H, s), 8.45 (1H, s, H-6), 8.26 (1H, d, *J* = 8.3 Hz), 8.10 (1H, d, *J* = 7.0 Hz), 7.79 (1H, d, *J* = 7.6, 7.8 Hz, H-8), 4.45 (1H, c, *J* = 7.1 Hz), 1.44 (3H, t, *J* = 7.1 Hz). ^13^C NMR (125 MHz, CDCl_3_) δ: 181.2 (C, C-4), 164.9 (C), 157.0 (CH), 148.2 (CH), 144.9 (CH), 136.2 (CH), 132.2 (CH), 132.5 (CH), 131.4 (C), 130.7 (CH), 128.4 (CH), 126.7 (C), 125.7 (C), 123.0 (C), 61.7 (CH_2_), 14.3 (CH_3_). EIMS: *m*/*z* 209 (42), 208 (54), 181 (M^+^, 100), 180 (24), 152 (32), 148 (76), 57 (30). HRMS (EI): calcd. for C_15_H_11_NO_3_ (M^+^) 253.0739, found 253.0729. UV-Vis (EtOH) λ_max_: 389, 341, 326, 244, 223 nm. IR (film) ν_max_: 3024, 1218, 748, 672, 476 cm^−1^. Compound **38**: ^1^H NMR (500 MHz, CDCl_3_) δ: 11.91 (1H, br s, OH), 10.87 (1H, s, CHO), 9.10 (1H, d, *J* = 8.6 Hz), 8.58 (1H, s, H-1), 7.76 (1H, d, *J* = 8.6 Hz), 7.66 (1H, ddd, *J* = 1.4, 6.9, 8.5 Hz), 7.40 (1H, ddd, *J* = 1.0, 6.9, 8.0 Hz), 4.49 (2H, c, *J* = 7.1 Hz), 1.49 (3H, t, *J* = 7.1Hz). EIMS: *m*/*z* 244 (M^+^, 35), 216 (28), 170 (100), 142 (34). HRMS (EI): calcd. for C_14_H_12_O_4_ (M^+^) 244.0736, found 244.0726. Mp: 140–142 °C.

**Synthesis of 5-bromo-1-methoxy-4*H*-benzo[*de*]isoquinolin-4-one (39)**.

To a solution of **35** (5 mg, 0.028 mmol) in MeOH (1 mL) was added NBS (5 mg, 0.028 mmol), and the reaction mixture was heated under reflux for 6 h. The solvent was removed under vacuum, and the residue was purified by preparative silica gel plate chromatography (hexane/EtOAc, 3:2) to give the products **40** (2 mg, 28%) as an orange oil and **39** (5 mg, 62%) as an orange solid. ^1^H NMR (500 MHz, CDCl_3_) δ: 9.24 (1H, s, H-3), 8.39 (1H, dd, J = 0.7, 8.7 Hz, H-9), 8.21 (1H, s, H-6), 7.15 (1H, d, J = 7.0 Hz, H-7), 7.64 (1H, dd, J = 8.1 Hz, H-8), 4.27 (3H, s, CH_3_). ^13^C NMR (125 MHz, CDCl_3_) δ: 177.8 (C, C-4), 165.1 (C, C-1), 150.1 (CH, C-3), 142.1 (CH, C-6), 133.0 (CH, C-7), 131.8 (C, C-9b), 127.8 (CH, C-9), 127.4 (CH, C-8), 127.3 (C, C-5), 127.0 (C, C-6a), 118.2 (C, C-3a), 117.1 (C, C-9a), 55.0 (CH_3_). EIMS: *m*/*z* 290 (M^+^, 100), 287 (98), 285 (35). HRMS (EI): calcd. for C_13_H_8_NO_2_Br (M^+^) 290.9716, found 290.9713. Mp: 220–223 °C.

**Synthesis of 5-bromo-4*H*-benzo[de]isoquinolin-4-one (40)**.

To a solution of **35** (6 mg, 0.033 mmol) in THF (0.7 mL) was added NBS (5 mg, 0.028 mmol) and the reaction mixture was heated under reflux for 6 h. The solvent was removed under vacuum and the residue was purified by preparative silica gel plate chromatography (hexane/EtOAc, 3:2) to give product **40** (3.3 mg, 38%) as an orange oil. ^1^H NMR (500 MHz, CDCl_3_) δ: 9.62 (1H, s), 9.58 (1H, s), 8.28 (1H, s, H-6), 8.21 (1H, dd, J = 0.7, 8.2 Hz), 7.94 (1H, d, J = 7.1 Hz), 7.75 (1H, dd, J = 8.3, 7.3 Hz, H-8). ^13^C NMR (125 MHz, CDCl_3_) δ: 178.7 (s, C-4), 157.5 (d), 149.0 (d), 142.4 (d), 133.9 (d), 131.3 (d), 129.8 (s), 128.7 (d), 127.5 (s), 127.3 (s), 127.0 (s), 122.0 (s). EIMS: *m*/*z* 260 (M^+^, 100), 258 (99), 180 (32), 152 (46), 125 (20). HRMS (EI): calcd. for C_12_H_6_NOBr (M^+^) 258.9633, found 260.9641. UV-Vis (EtOH) λ_max_: 399, 344, 328, 252, 237 nm. IR (film) ν_max_: 3019, 1214, 647, 466 cm^−1^.

**Synthesis of 5-bromo-9-methoxy-4*H*-benzo[de]isoquinolin-4-one (41)**.

To a solution of **36** (37 mg, 0.175 mmol) in THF (2 mL) was added NBS (31 mg, 0.175 mmol), and the reaction mixture was stirred at room temperature for 4 h. The solvent was removed under vacuum, and the residue was purified by silica gel column chromatography (CH_2_Cl_2_) to give product **41** (22 mg, 44%) as an orange solid. ^1^H NMR (500 MHz, CDCl_3_) δ: 9.83 (1H, s), 9.60 (1H, s), 8.12 (1H, s, H-6), 7.79 (1H, d, *J* = 8.1 Hz), 6.94 (1H, d, *J* = 8.1 Hz), 4.15 (3H, s, CH_3_). ^13^C NMR (125 MHz, CDCl_3_) δ: 178.5 (s, C-4), 161.0 (s), 152.5 (d), 149.6 (d), 142.4 (d), 136.4 (d), 130.6 (s), 123.6 (s), 121.6 (s), 119.9 (s), 119.6 (s), 106.5 (d), 56.5 (c, CH_3_). EIMS: *m*/*z* 290 (M^+^, 100), 289 (49), 288 (99), 287 (37), 261 (30), 259 (30). HRMS (EI): calcd. for C_13_H_8_NO_2_Br (M^+^) 290.9718, found 290.9715. UV-Vis (EtOH) λ_max_: 397, 351, 257, 224 nm. IR (film) ν_max_: 3016, 1214, 744, 668, 462 cm^−1^. Mp: 215–217 °C.

### 3.3. Biological Assays

**In vitro antimalarial assay**.

FCR-3 chloroquine-resistant strain of *P. falciparum* (kindly provided by Dr. Fandeur, Pasteur Institute, Cayenne, France) was cultured [30] on glucose-enriched RPMI 1640 medium, supplemented with 10% human serum at 37 °C. After 24 h of incubation at 37 °C, the medium was replaced by fresh medium supplemented with the compound to be evaluated (initial a screening used a concentration of 10 μg/mL), and incubation was continued for a further 48 h. On the third day of the test, a blood smear was taken from each well, and parasitemia was calculated for each concentration of sample compared to the control [31]. Chloroquine (0.1 μg/mL) was used as a positive control. IC_50_ values were determined graphically by plotting concentrations (0.01–10 μg/mL) versus percent inhibition. All tests were performed in triplicate.

**Cytotoxicity study by MTT proliferation assay**.

MCF-7 cells were purchased from ATCC (Manassas, VA, USA) and cultured in DMEN containing 10% FBS. For 3-(4,5-dimethylthiazol-2-yl)-2,5-diphenyltetrazolium (MTT) (Sigma–Aldrich, St. Louis, MO, USA) analysis, cells were plated in 96-well plates at 10,000 cells/well. Twenty-four hours after plating, vehicle (0.1% DMSO, final concentration) or compound was added to cells at indicated doses. Seventy-two hours following compound addition, MTT was added to each well (0.5 mg/mL, final concentration) and plates were incubated for an additional 3 h at 37 °C. Medium was then aspirated and the formazan product was solubilized in SDS–HCl (20% SDS; HCl 0.02 M). The absorbance of each well was measured at 570 nm using a microplate reader (Sigma–Aldrich, St. Louis, MO, USA).

## 4. Conclusions

A diverse set of 1*H*-phenalen-1-one analogs was successfully synthesized using several complementary synthetic strategies. These compounds were designed to expand the chemical diversity of our 1*H*-phenalen-1-one collection and to assemble a focused library centered on this core scaffold. This library enables evaluation of how electronic and steric modifications influence both the chemical reactivity and biological activity of 1*H*-phenalen1-one derivatives. The inclusion of benzo[*de*]isoquinolin-4-one nitrogen analogs further enriches the structural scope. Additionally, the unexpected formation of phenoxazine and acrid-7-one systems was observed when 2-bromo-1*H*-phenalen-1-one was treated with selected anilines under cross-coupling conditions. Most compounds were obtained in moderate to good yields, with 23 identified as novel. Biological screening against the chloroquine-resistant *Plasmodium falciparum* strain FCR3 revealed three new antiplasmodial agents (compounds **2**, **9-OH**, and **18**) with IC_50_ values below 1 µM, representing a significant improvement over previously reported phenalenone derivatives (compounds **1c** and **1d**). None of the active compounds displayed detectable cytotoxicity. Together with their favorable predicted physicochemical properties, these results support further exploration of structure–activity relationships (SAR) within this promising class and investigation into their mechanism of action. We also highlight that, to date, these constitute the only reports describing antiplasmodial activity for this structural family, offering a new scaffold for antimalarial drug discovery and one of particular interest to medicinal chemists working with phenalenone derivatives.

## Figures and Tables

**Table 1:** In vitro antiplasmodial activity and cytotoxicity of 1*H*-phenalen-1-one derivatives.

Compound	Chloroquine–Resistant P. falciparum FCR3 IC50 ± SD (μM) ^a^	MCF-7 % Inh ± DS (10 μM) ^b^
**1**	2.46 ± 0.08	16 ± 0.4
**2**	0.70 ± 0.04	2 ± 0.7
**3**	>10	4 ± 0.4
**4**	>10	14 ± 0.8
**5**	>10	12 ± 0.3
**6**	>10	10 ± 2
**7**	>10	22 ± 0.2
**9**	>10	6 ± 0.1
**10**	>10	10 ± 1
**11**	>10	8 ± 0.2
**12**	>10	15 ± 0.3
**13**	>10	12 ± 0.1
**14**	>10	15 ± 0.2
**15**	>10	1 ± 0.4
**16**	>10	0
**17**	>10	12 ± 0.3
**18**	0.38 ± 0.06	32 ± 0.5
**19**	>10	0
**20**	>10	11 ± 0.2
**21**	>10	8 ± 0.3
**22**	>10	42 ± 0.1
**23**	>10	12 ± 0.6
**24**	>10	7 ± 0.5
**25**	>10	16 ± 0.9
**9-OH**	0.72 ± 0.06	6 ± 0.7
**30**	>10	16 ± 0.4
**33**	>10	0
**34**	>10	0
**35**	>10	7 ± 0.1
**36**	>10	5 ± 0.9
**Chloroquine ^c^**	0.16 ± 0.02	
**Adriamycin ^c^**		63 ± 4.7

^a^ Data are expressed as mean standard deviation of three determinations. ^b^ Cytotoxicity in MCF7 cells (10 μM). Inhibition of cell growth. Data are expressed as % inhibition = % activity in the presence of vehicle − % activity in the presence of inhibitor. ^c^ Positive control.

**Table 2:** Calculated physicochemical properties for the most active compounds ^a,b^.

Comp.	MW (g/mol)	LogP	# of Lipinski Violations	Oral Bioavailability (Veber Rule)
**1**	180.20	2.91	0	Good
**2**	259.10	3.46	0	Good
**9-OH**	196.20	3.25	0	Good
**18**	205.21	2.76	0	Good

^a^ Parameters measured using FAF-Drug3 web service [27]; ^b^ optimal range MW < 500, LogP < 5.

## Data Availability

The data are contained within the article and Supplementary Materials.

## Supplementary Materials

The following supporting information can be downloaded at: https://www.mdpi.com/article/10.3390/molecules30244667/s1, Supplementary Materials-S1: ^1^H and ^13^C NMR spectra; ^19^F NMR spectra of compound **6**; ^19^F NMR spectra of compound **11**; ^1^H NMR, ^13^C NMR, COSY, HSQC and HMBC spectra of compound **26**; ^1^H NMR, ^13^C NMR, COSY, HSQC and HMBC spectra of compound **27;** ^1^H NMR, ^13^C NMR, COSY, HSQC and HMBC spectra of compound **39**; Scheme S1. Proposed mechanism for the formation of compounds **36** and **37**; Supplementary Materials-S2: High-resolution mass spectra; Supplementary Materials-S3: UV-VIS spectra.
