# Supplementary material for: Synthesis of Substituted 1H-Phenalen-1-ones and Nitrogen-Containing Heterocyclic Analogues as Potential Anti-Plasmodial Agents
Source: Molecules. 2025 Dec 5;30(24):4667. doi: 10.3390/molecules30244667 (PMC12735704; doi:10.3390/molecules30244667)
Supplement: Supplementary file 1 [file molecules-30-04667-s001.zip › Supplementary Material-S1.pdf]

Supplementary Materials

Synthesis of Substituted 1*H*-Phenalen-1-ones and Nitrogen-Containing Heterocyclic Analogues as Potential Anti-plasmodial Agents

Teresa Abad-Grillo, [1,*] Grant McNaughton-Smith, [2,*] Mónica Blanco Freijó, [1] David Gutiérrez[3] and Ninoska Flores[3]

[1] Departamento de Química Orgánica, Universidad de La Laguna, Avenida Astrofísico Francisco Sánchez, 2, 38206 La Laguna, Tenerife, Spain; tereabad@ull.edu.es

[2] Centro Atlántico del Medicamento S.A (CEAMED S.A.), PCTT, 38200 La Laguna, Tenerife, Spain; gmcsmith@ceamedsa.com

[3] Instituto de Investigaciones Fármaco Bioquímicas, Facultad de Ciencias Farmacéuticas y Bioquímicas, Universidad Mayor de San Andrés, Avenida Saavedra 2224, Miraflores, La Paz, Bolivia

* Correspondence: tereabad@ull.edu.es; gmcsmith@ceamedsa.com

Contents: Page

1. ^{1}H and ^{13}C spectra 2
2. ^{19}F NMR spectra of compound **6** 13
3. ^{19}F NMR spectra of compound **11** 28
4. ^{1}H NMR, ^{13}C NMR, COSY, HSQC and HMBC spectra of compound **26** 87
5. ^{1}H NMR, ^{13}C NMR, COSY, HSQC and HMBC spectra of compound **27** 97
6. ^{1}H NMR, ^{13}C NMR, COSY, HSQC and HMBC spectra of compound **39** 104
7. Scheme S1. Proposed mechanism for the formation of compounds **36** and **37** 114

1. ^{1}H and ^{13}C NMR spectra of products

^{1}H NMR spectrum (400 MHz, $CDCl_3$) of compound **4**:

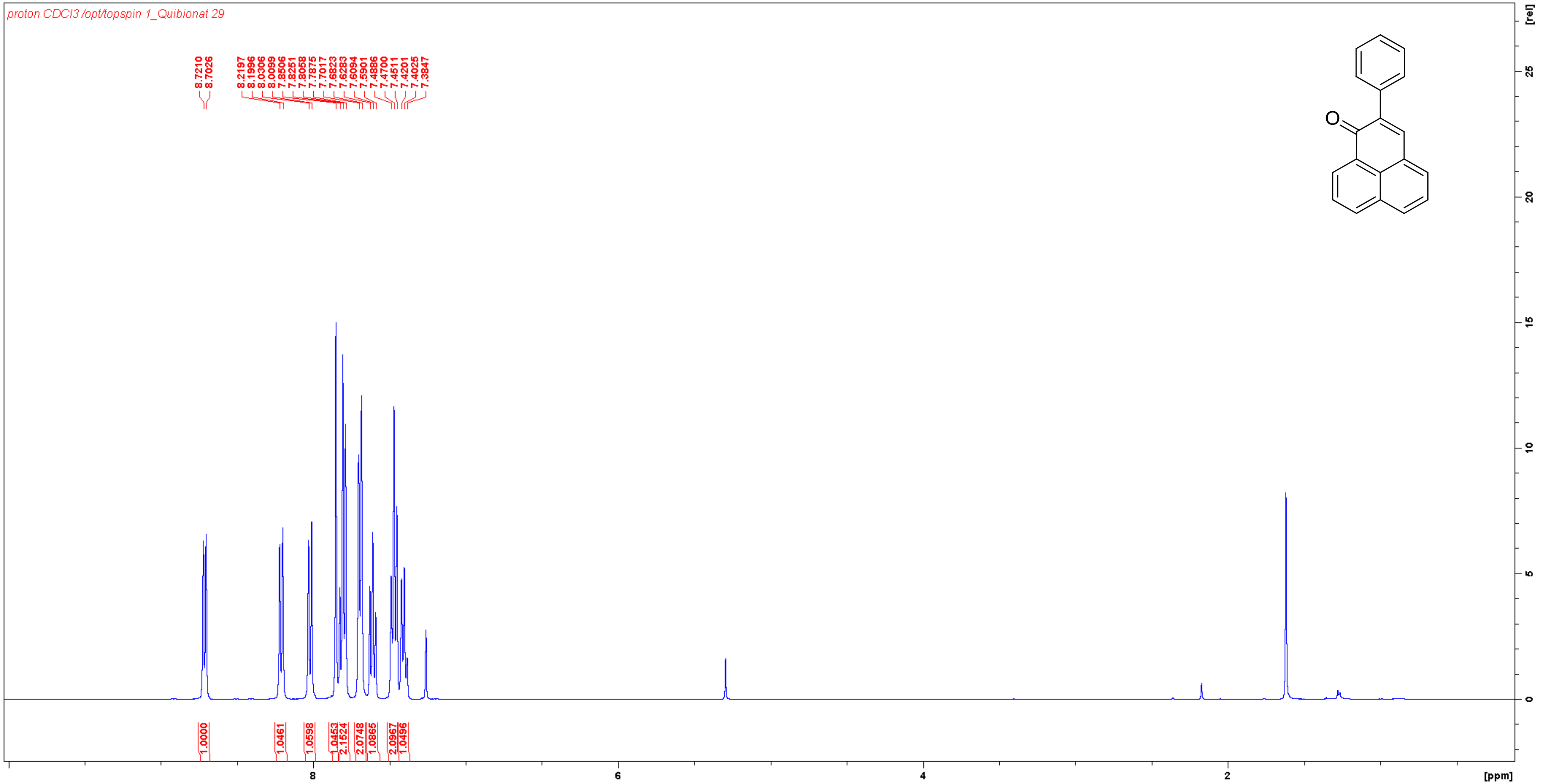

^{13}C NMR spectrum (100 MHz, $CDCl_3$) of compound **4**:

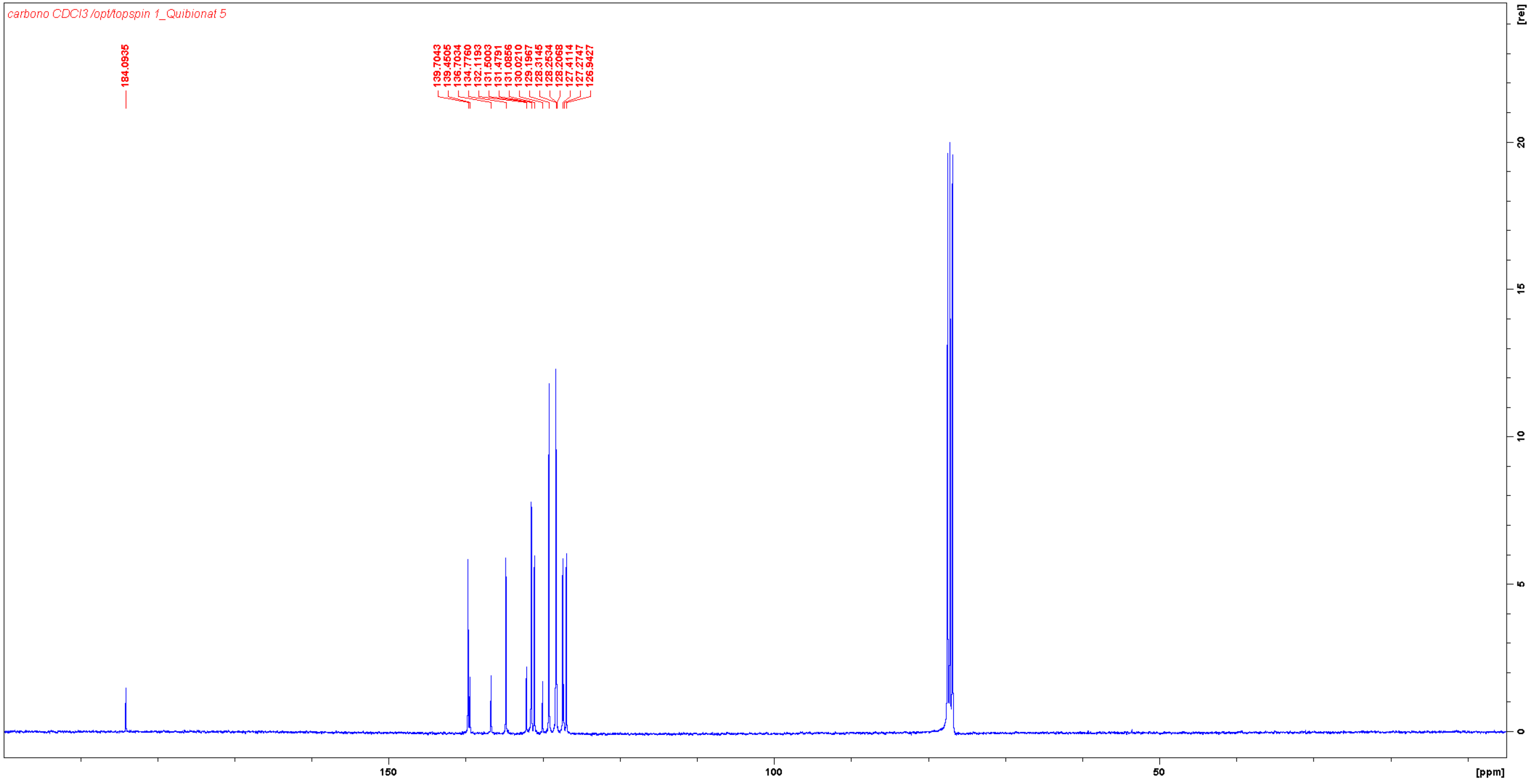

Expanded ^{13}C NMR spectrum (100 MHz, $CDCl_3$) of compound **4**:

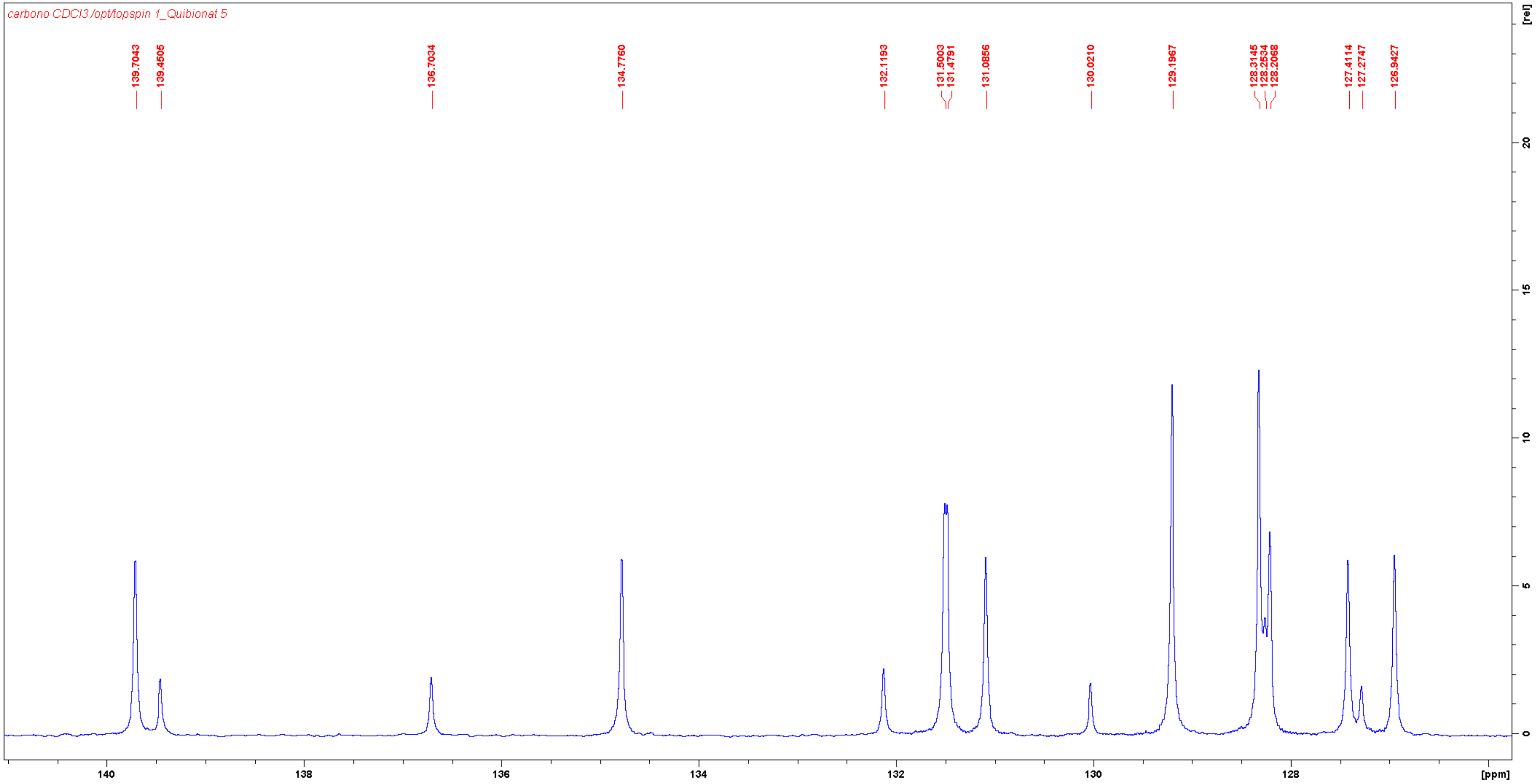

^{1}H NMR spectrum (500 MHz, $CDCl_3$) of compound **5**:

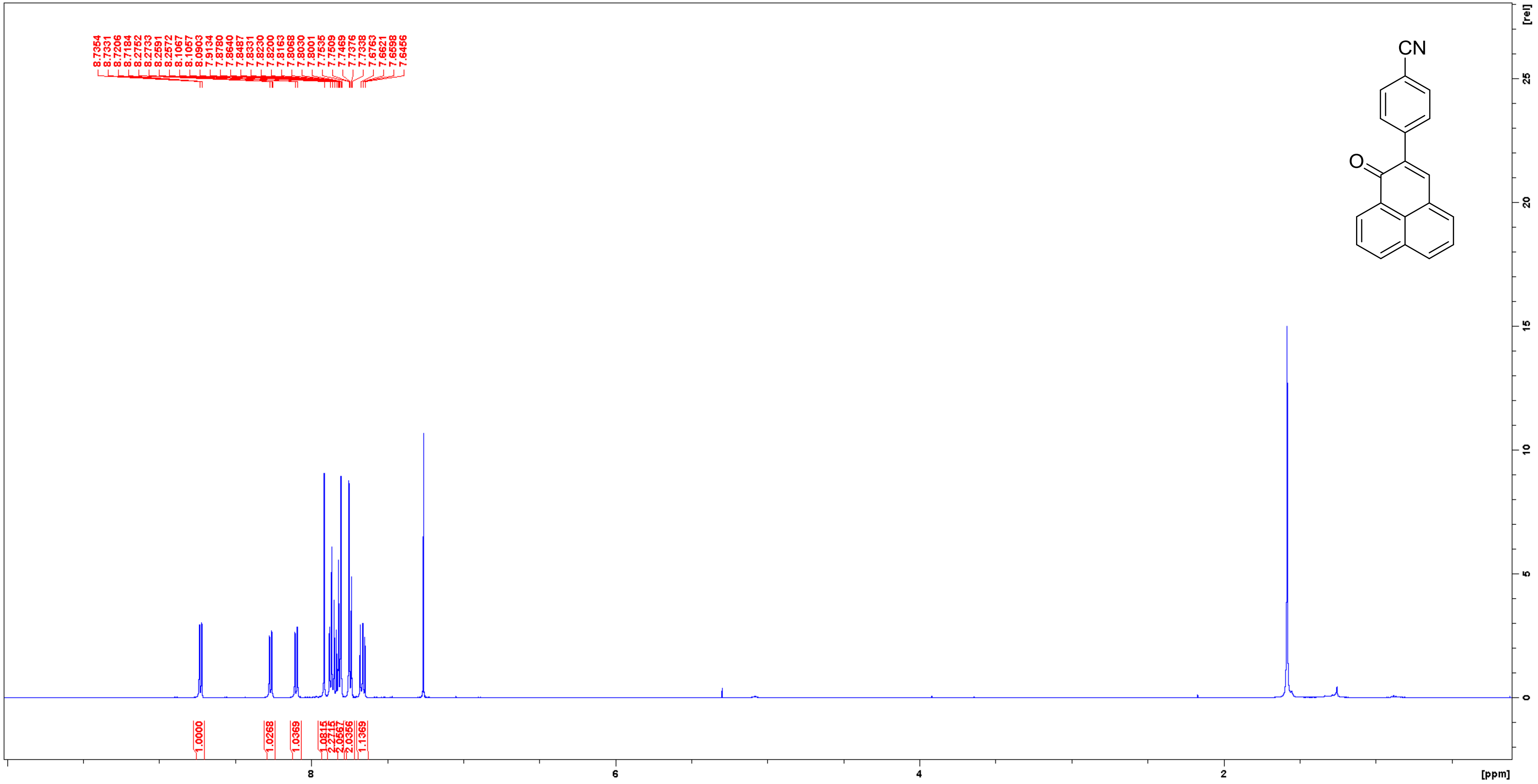

Expanded ^{1}H NMR spectrum (500 MHz, $CDCl_3$) of compound **5**:

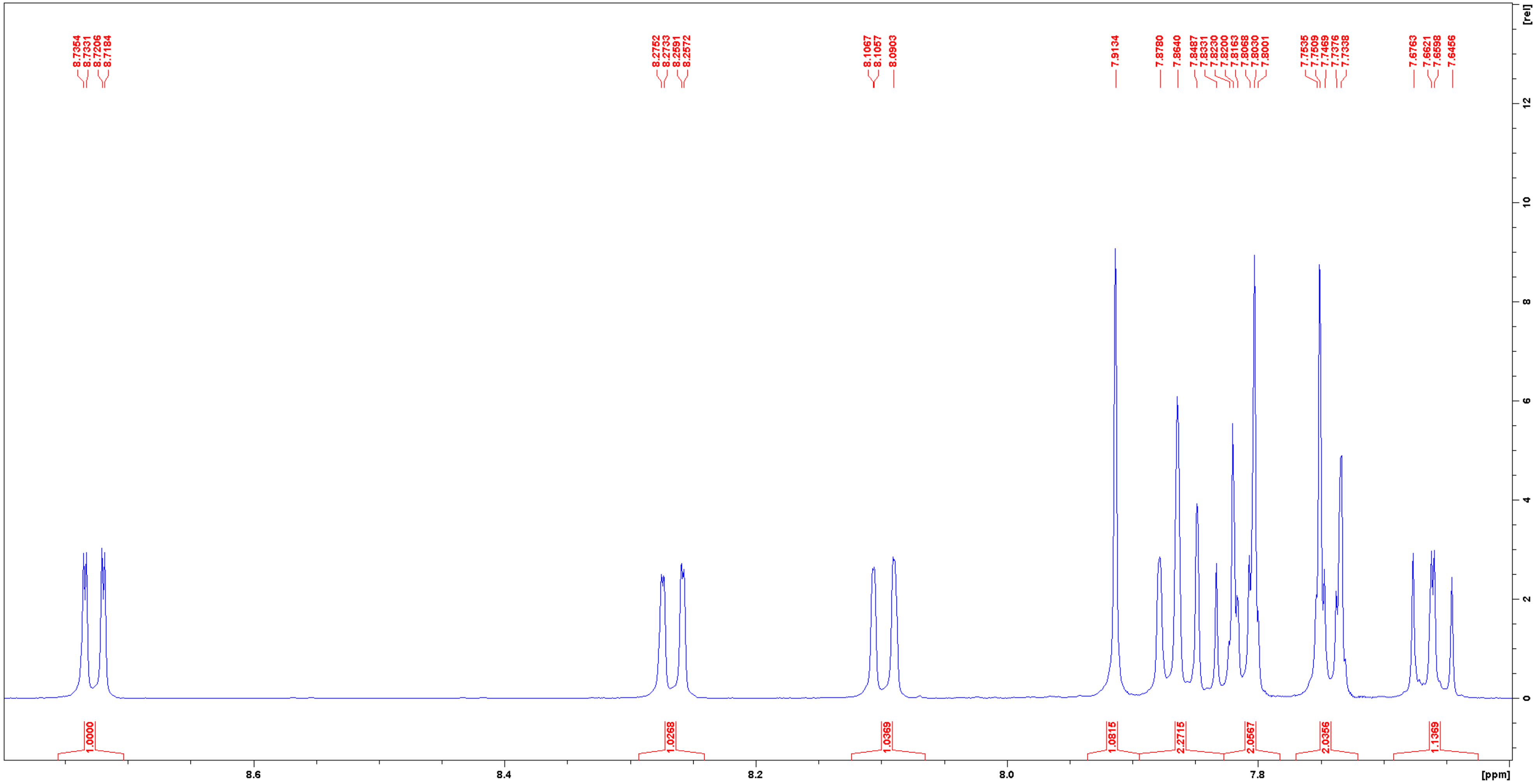

^{13}C NMR spectrum (125 MHz, $CDCl_3$) of compound **5**:

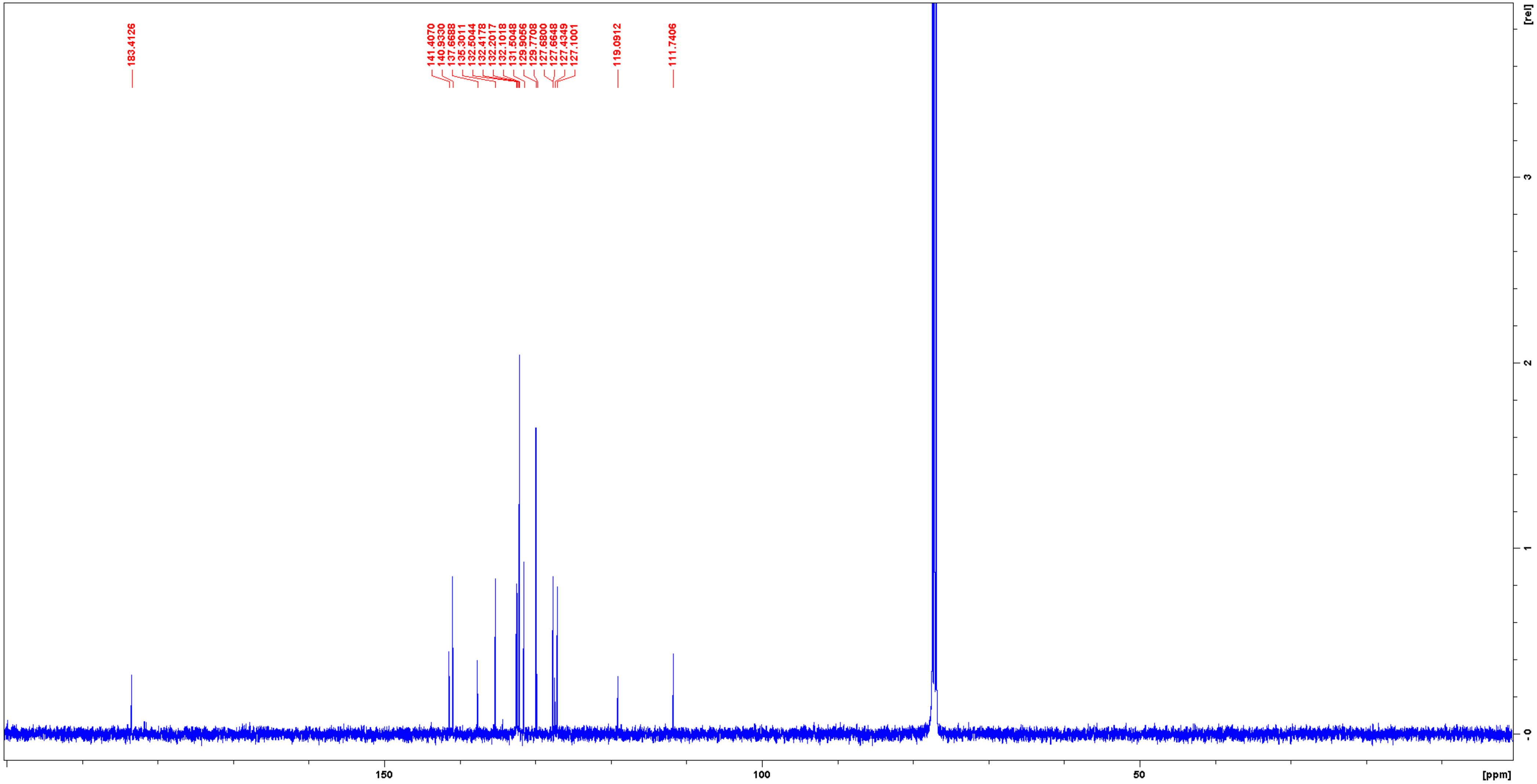

^{1}H NMR spectrum (500 MHz, $CDCl_3$) of compound **6**:

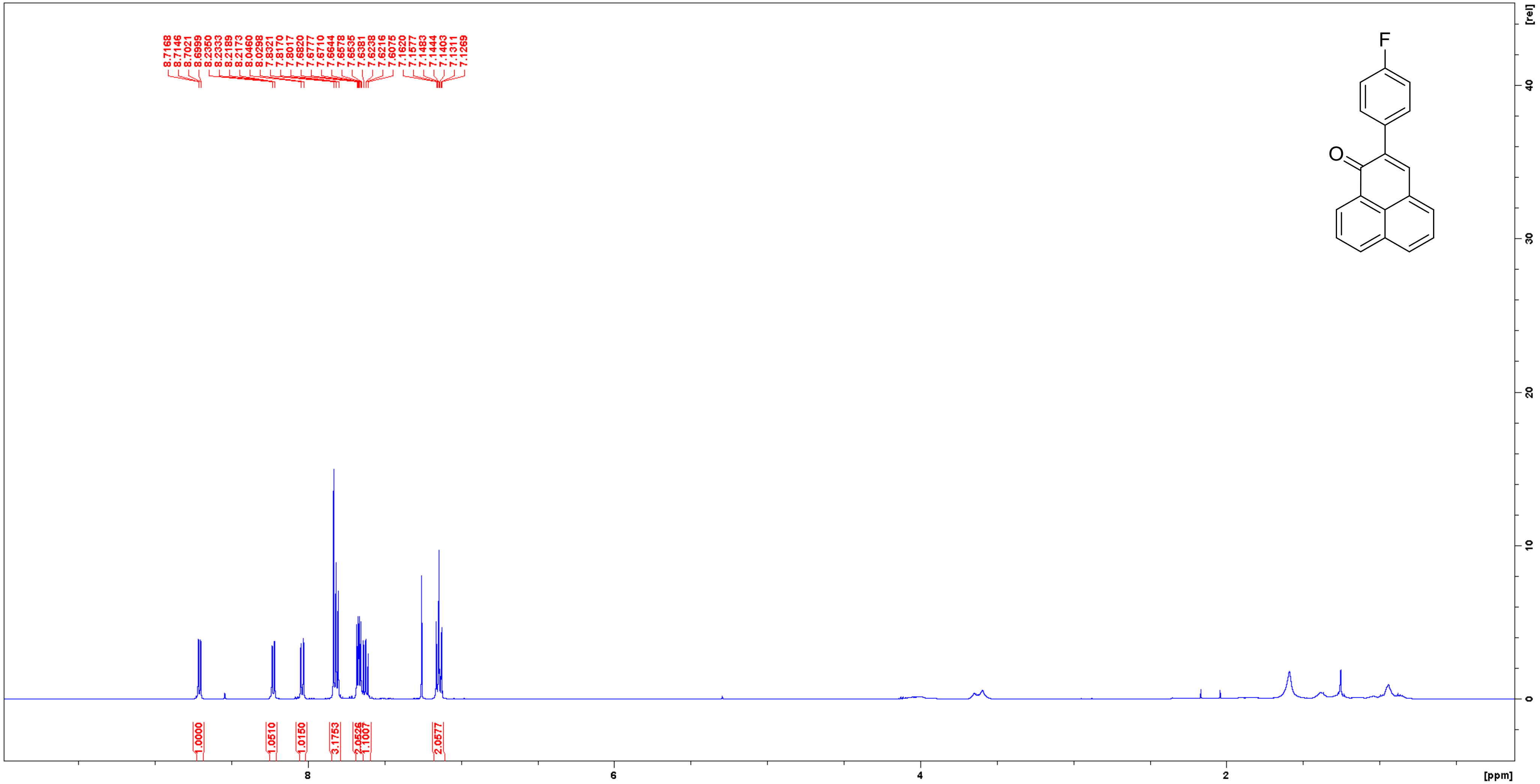

Expanded ^{1}H NMR spectrum (500 MHz, $CDCl_3$) of compound **6**:

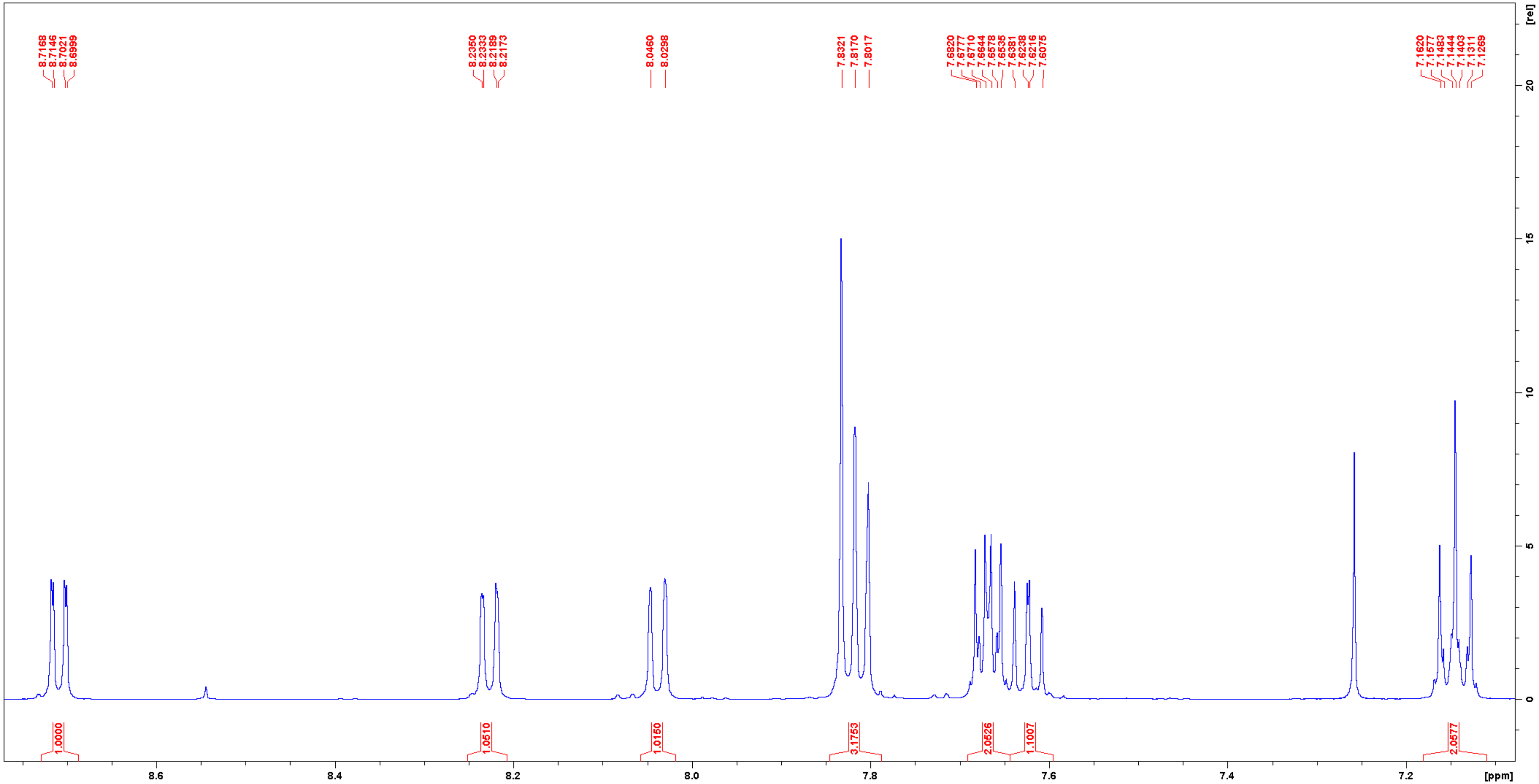

^{13}C NMR spectrum (125 MHz, $CDCl_3$) of compound **6**:

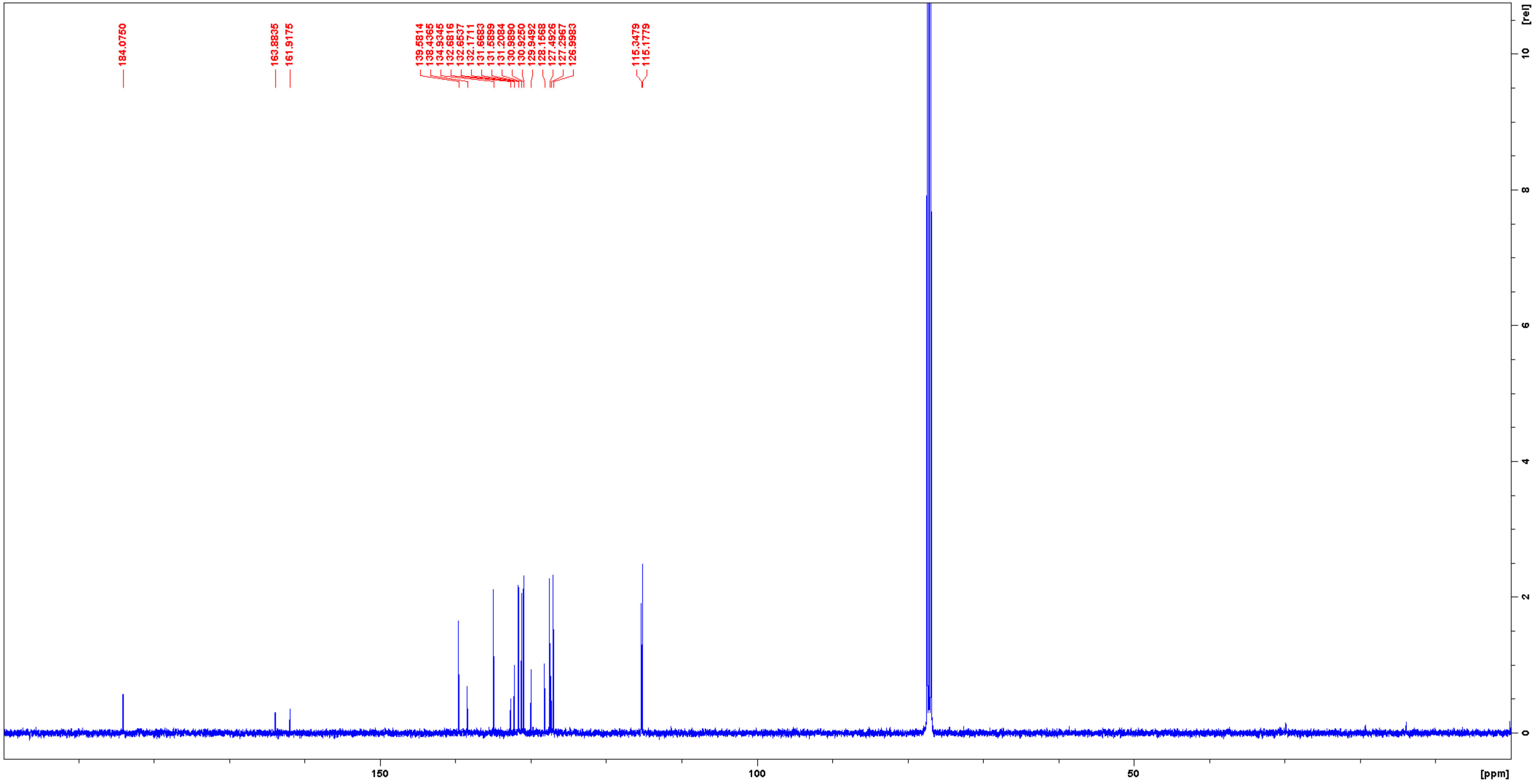

Expanded ^{13}C NMR spectrum (125 MHz, $CDCl_3$) of compound **6**:

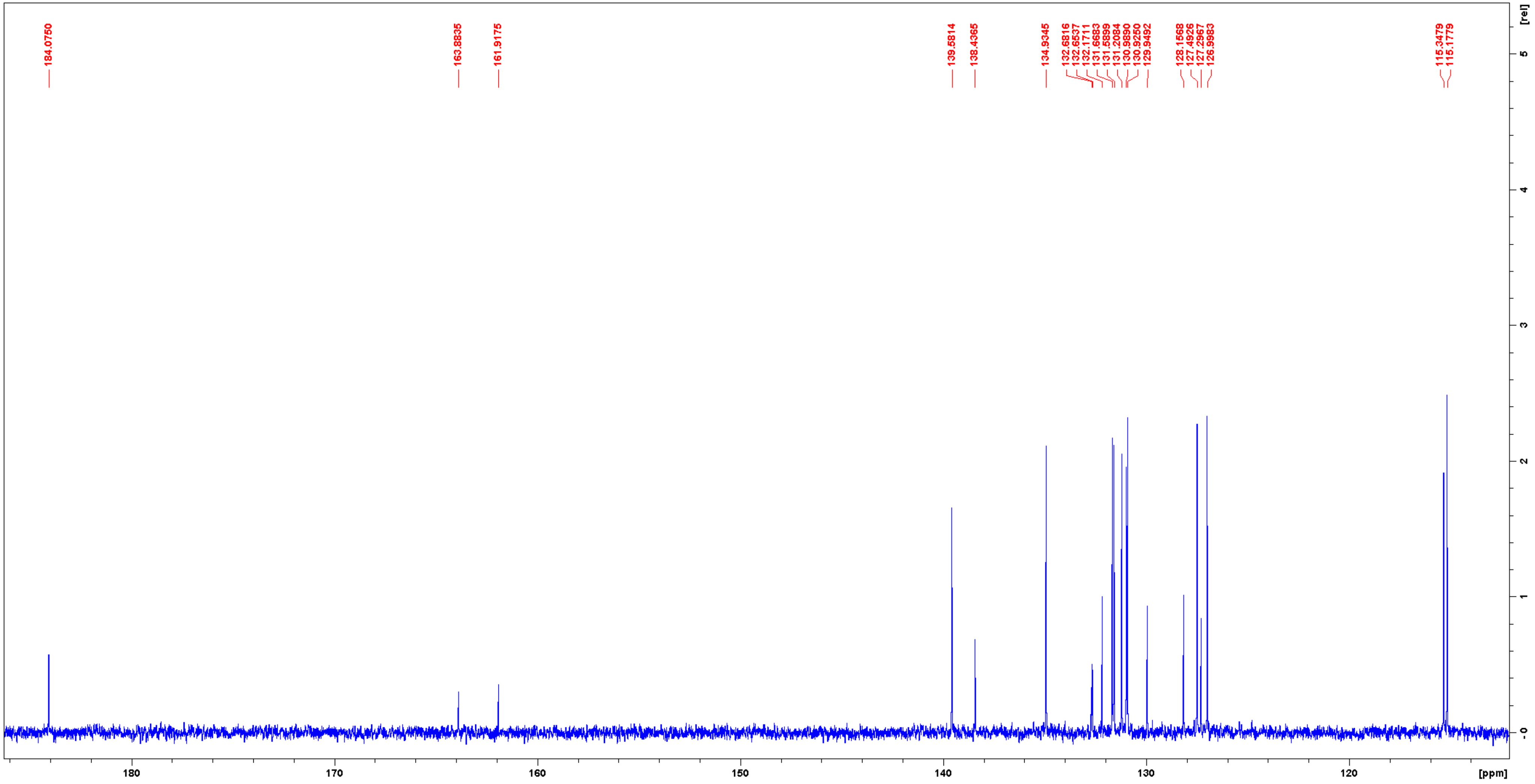

Expanded ^{13}C NMR spectrum (125 MHz, $CDCl_3$) of compound **6**:

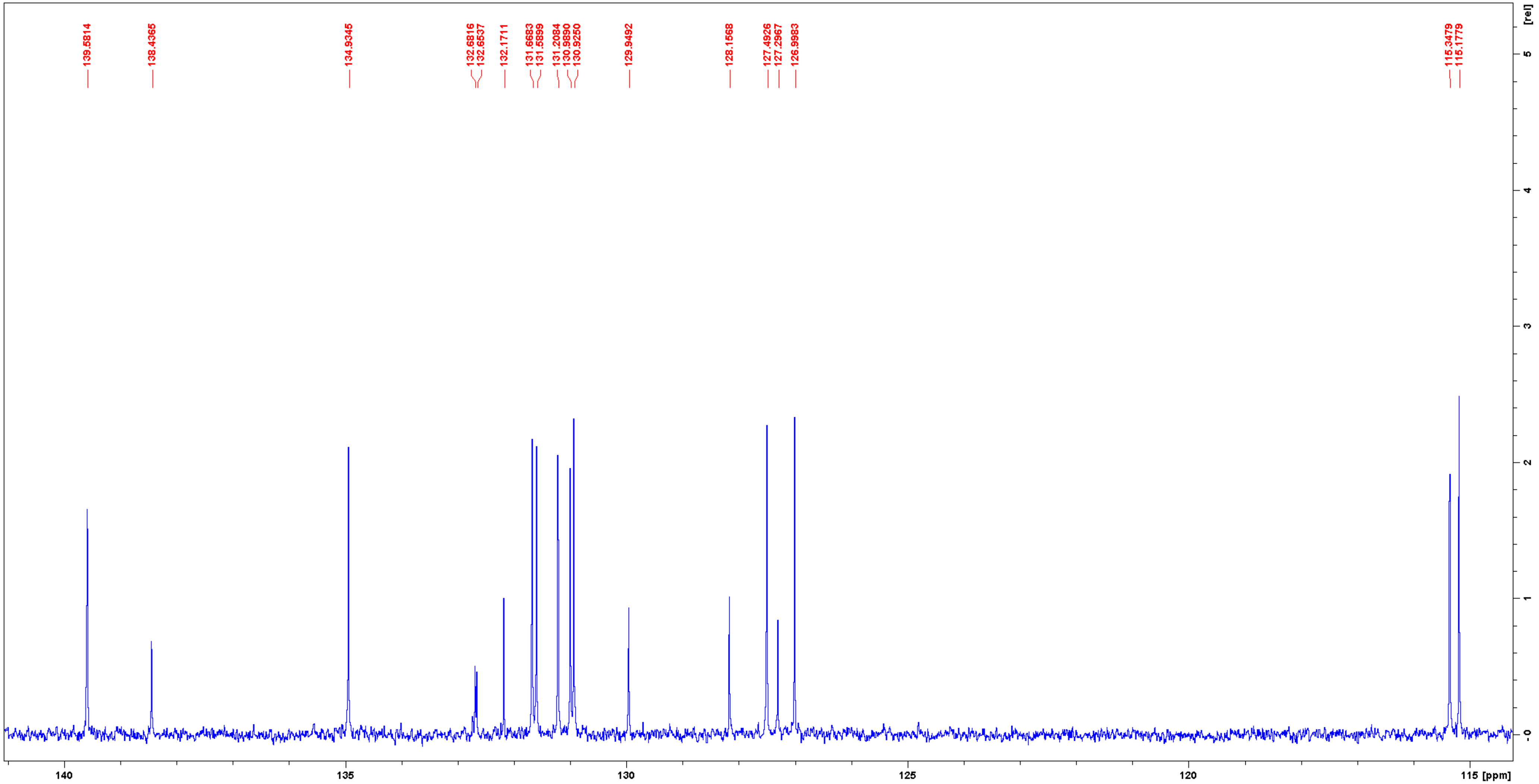

2. ^{19}F NMR spectrum (470 MHz, $CDCl_3$) of 6

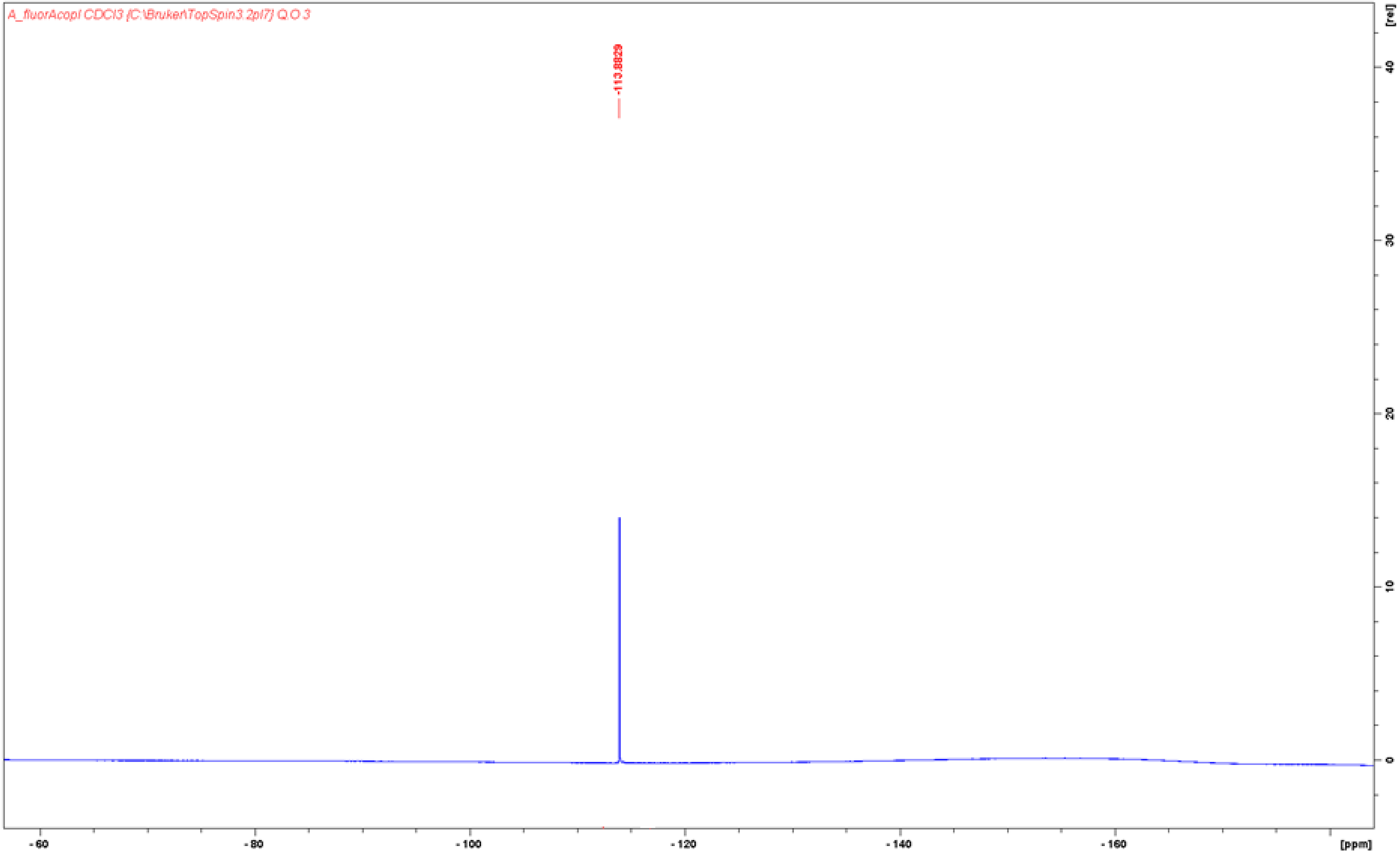

^{1}H NMR spectrum (400 MHz, $CDCl_3$) of compound **7**:

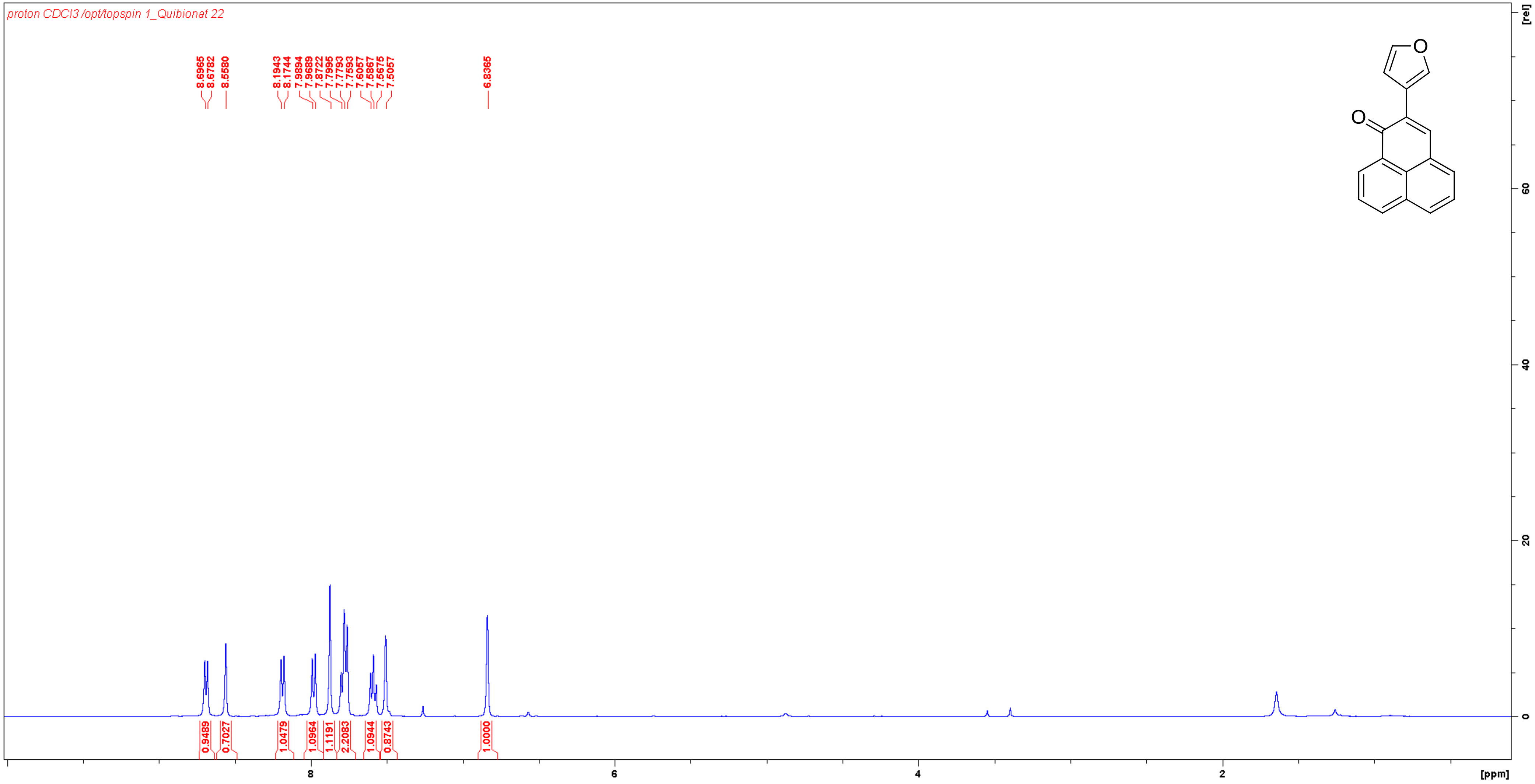

^{13}C NMR spectrum (100 MHz, $CDCl_3$) of compound **7**:

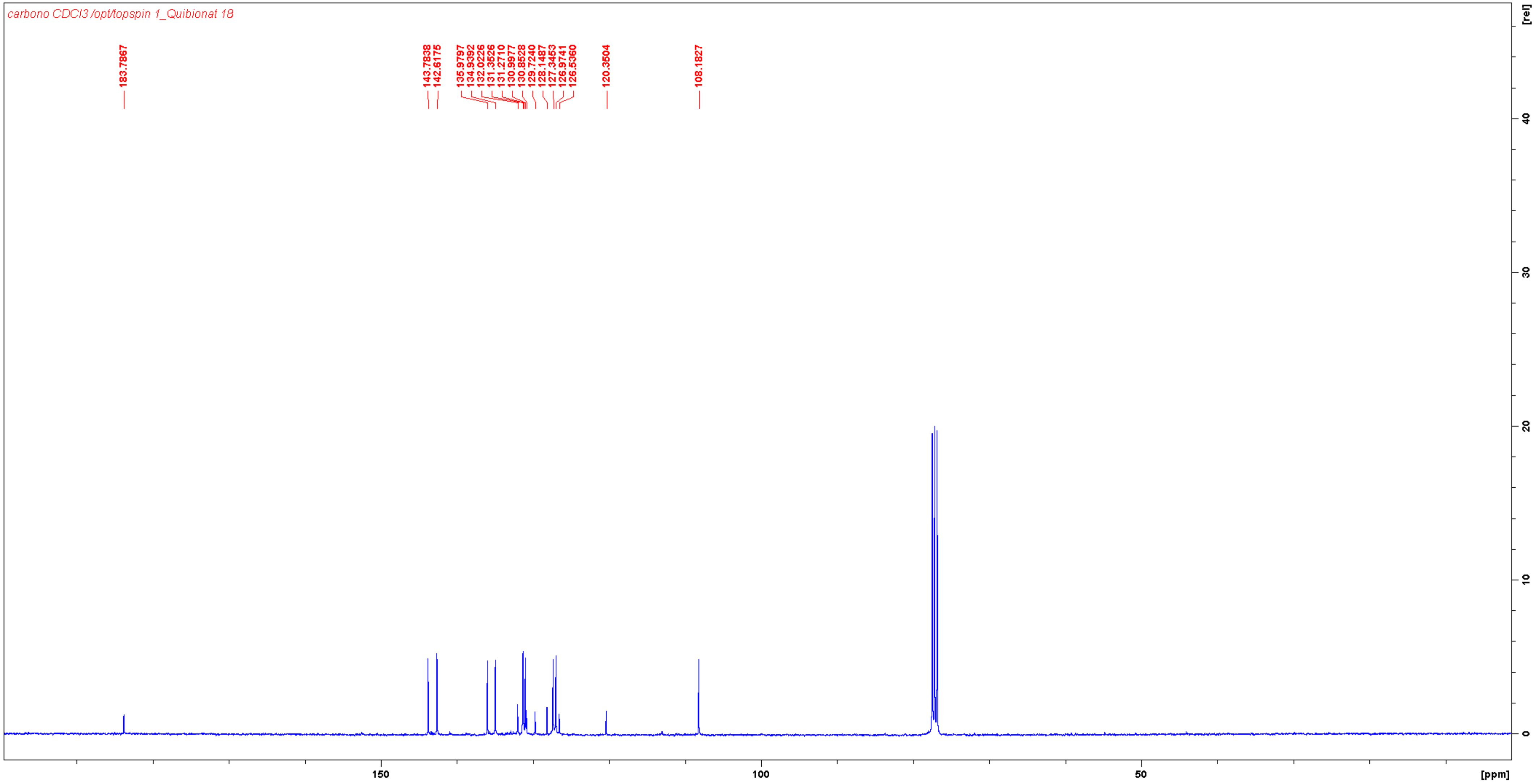

Expanded ^{13}C NMR spectrum (100 MHz, $CDCl_3$) of compound **7**:

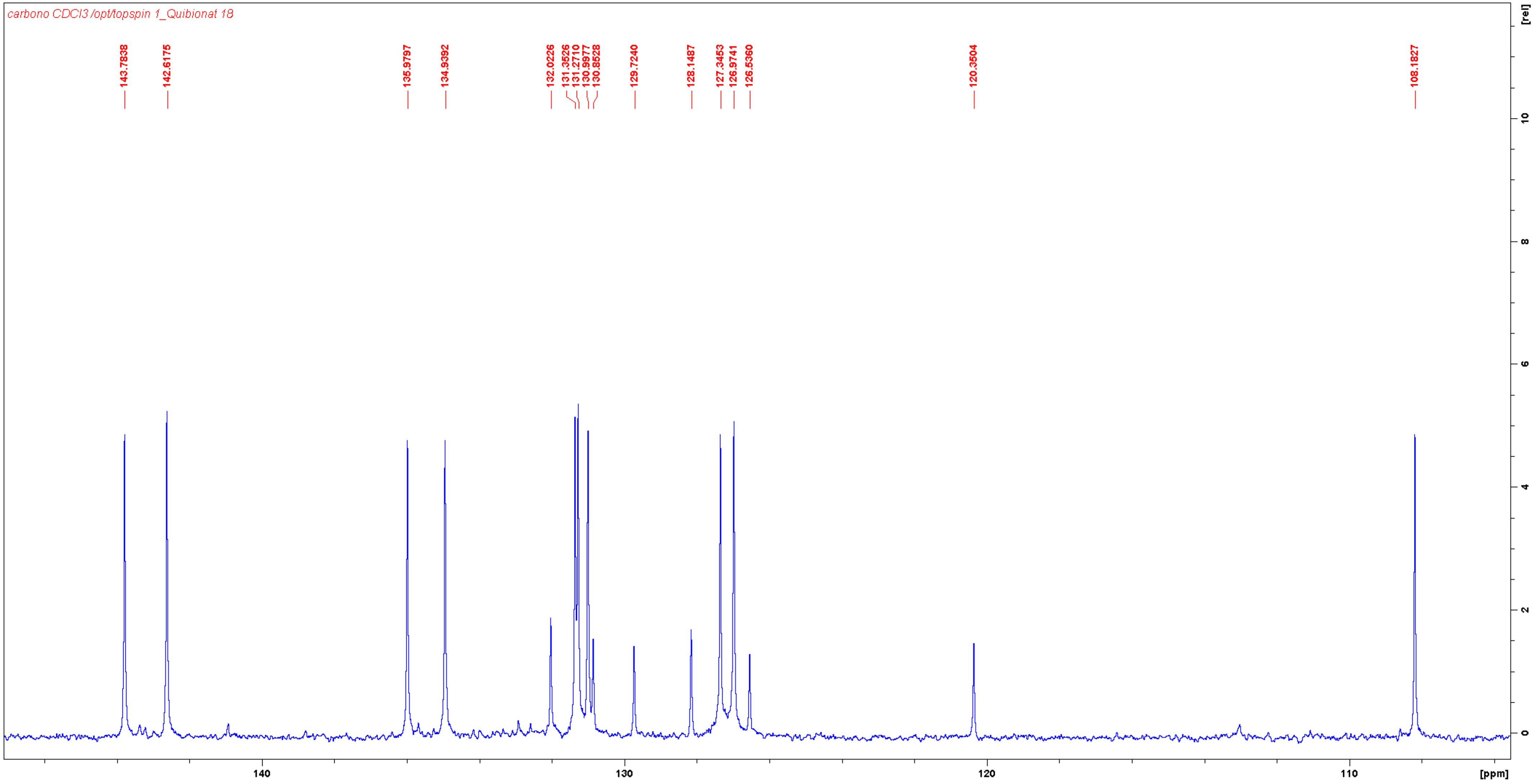

^{1}H NMR spectrum (400 MHz, $CDCl_3$) of compound **8**:

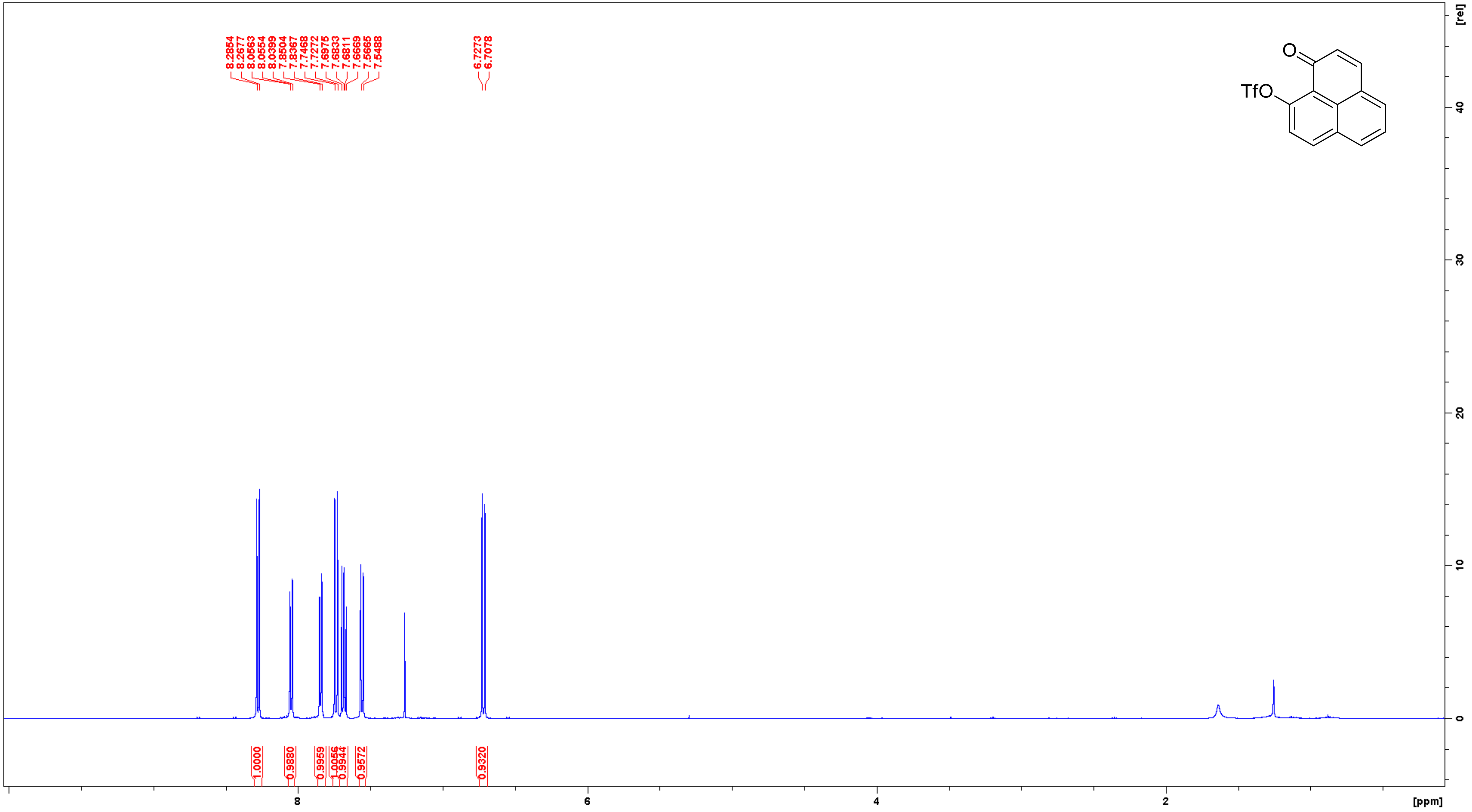

Expanded ^{1}H NMR spectrum (400 MHz, $CDCl_3$) of compound **8**:

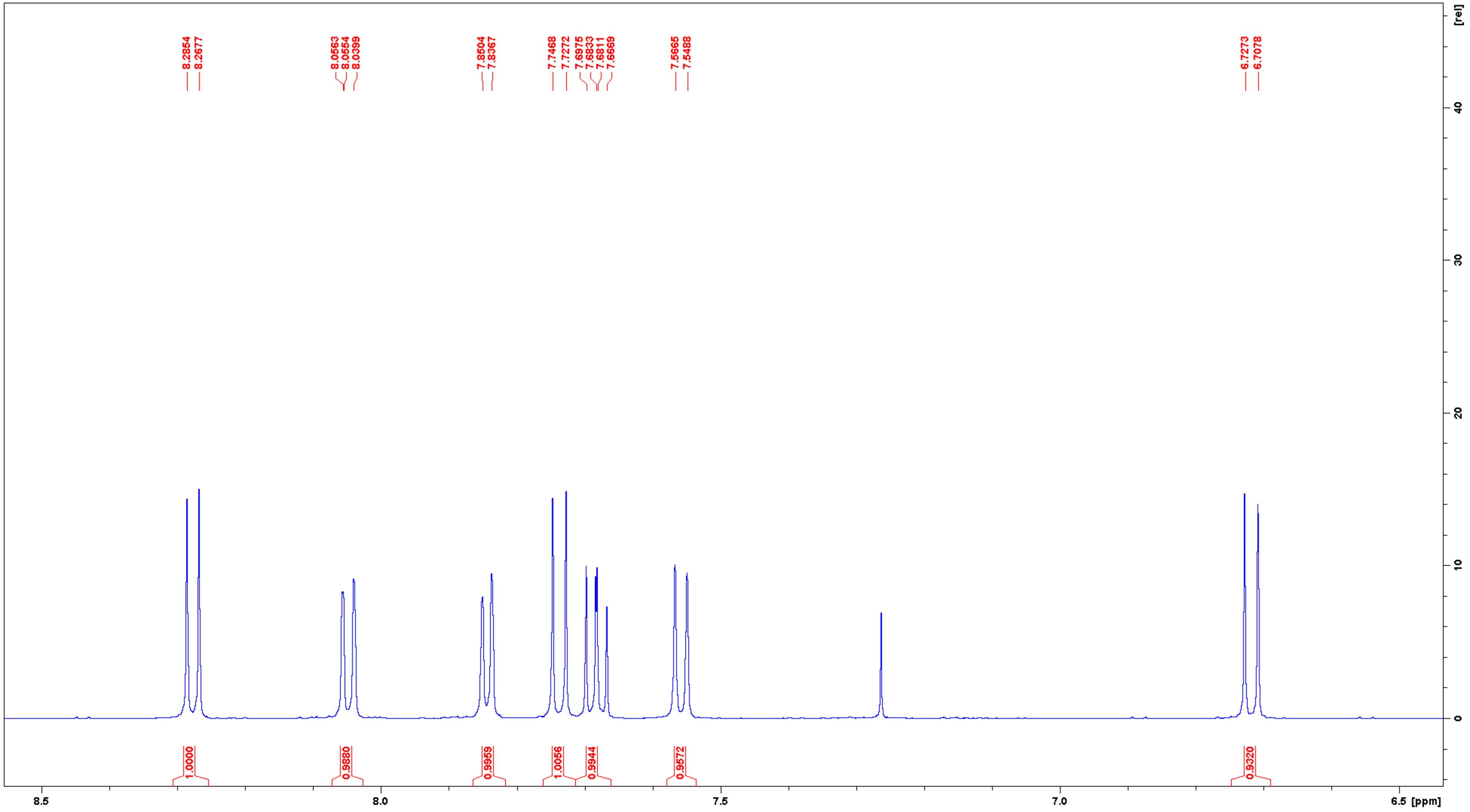

^{1}H NMR spectrum (500 MHz, $CDCl_3$) of compound **9**:

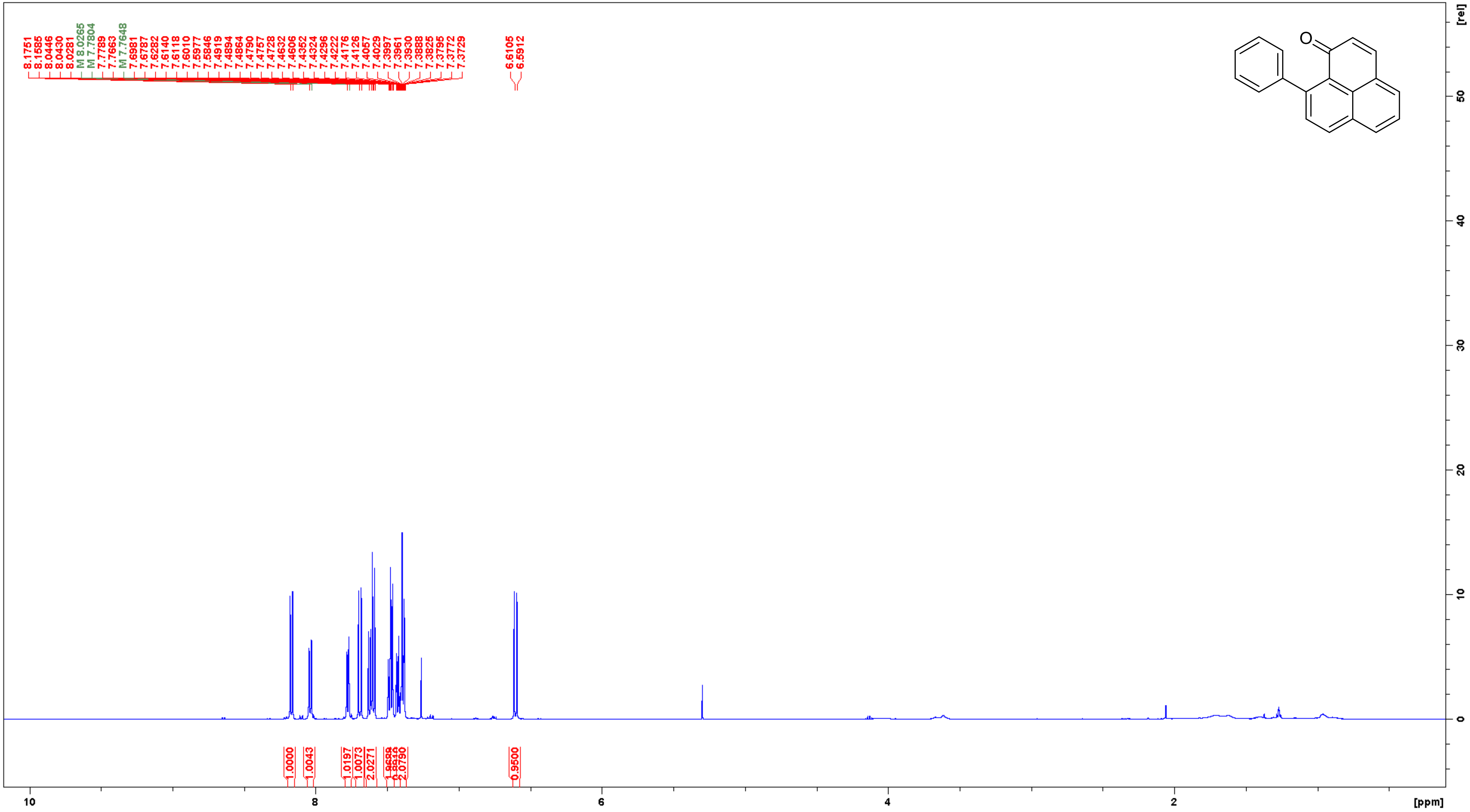

^{1}H NMR spectrum (500 MHz, $CDCl_3$) of compound **10**:

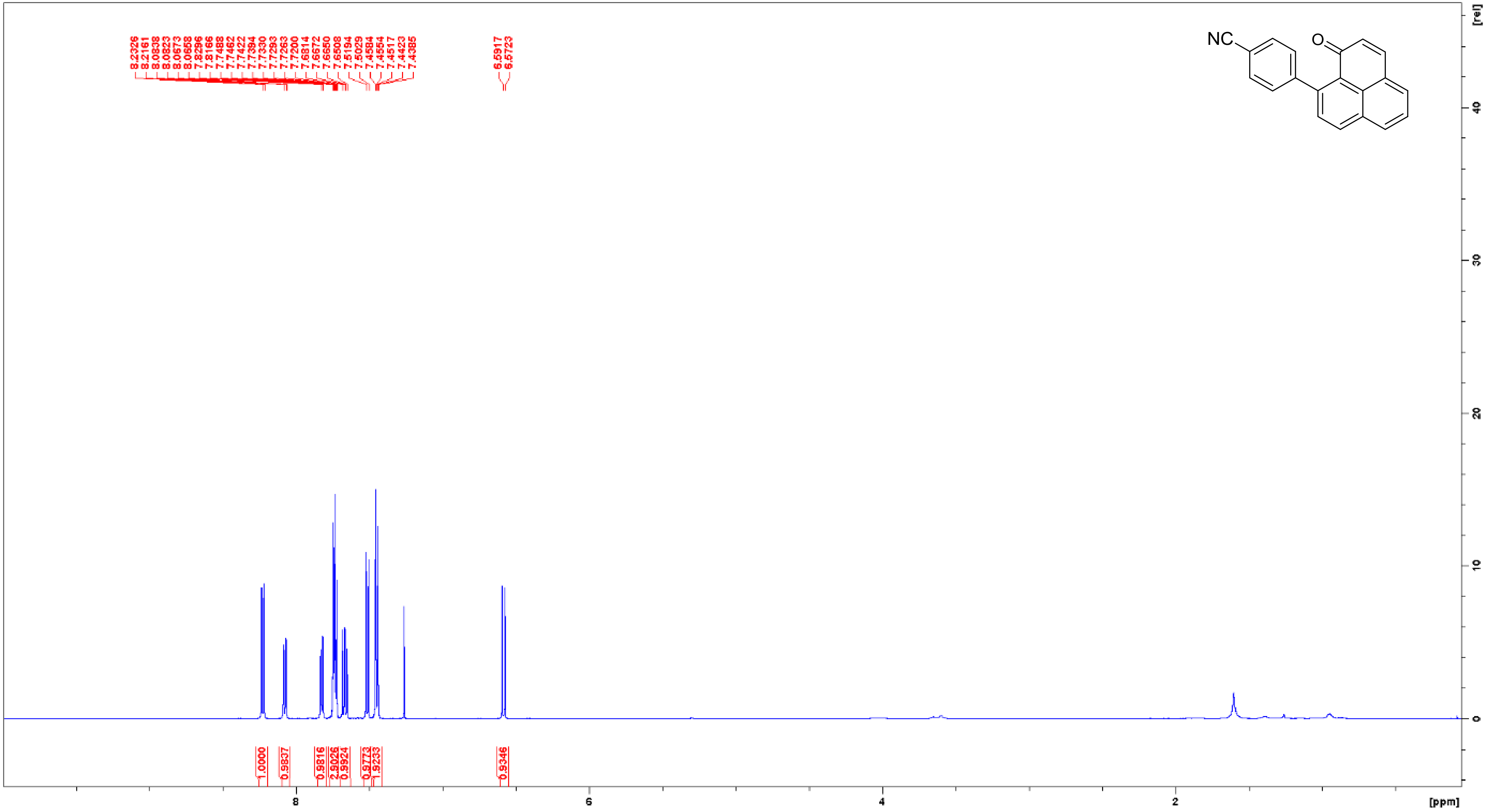

Expanded ^{1}H NMR spectrum (500 MHz, $CDCl_3$) of compound **10**:

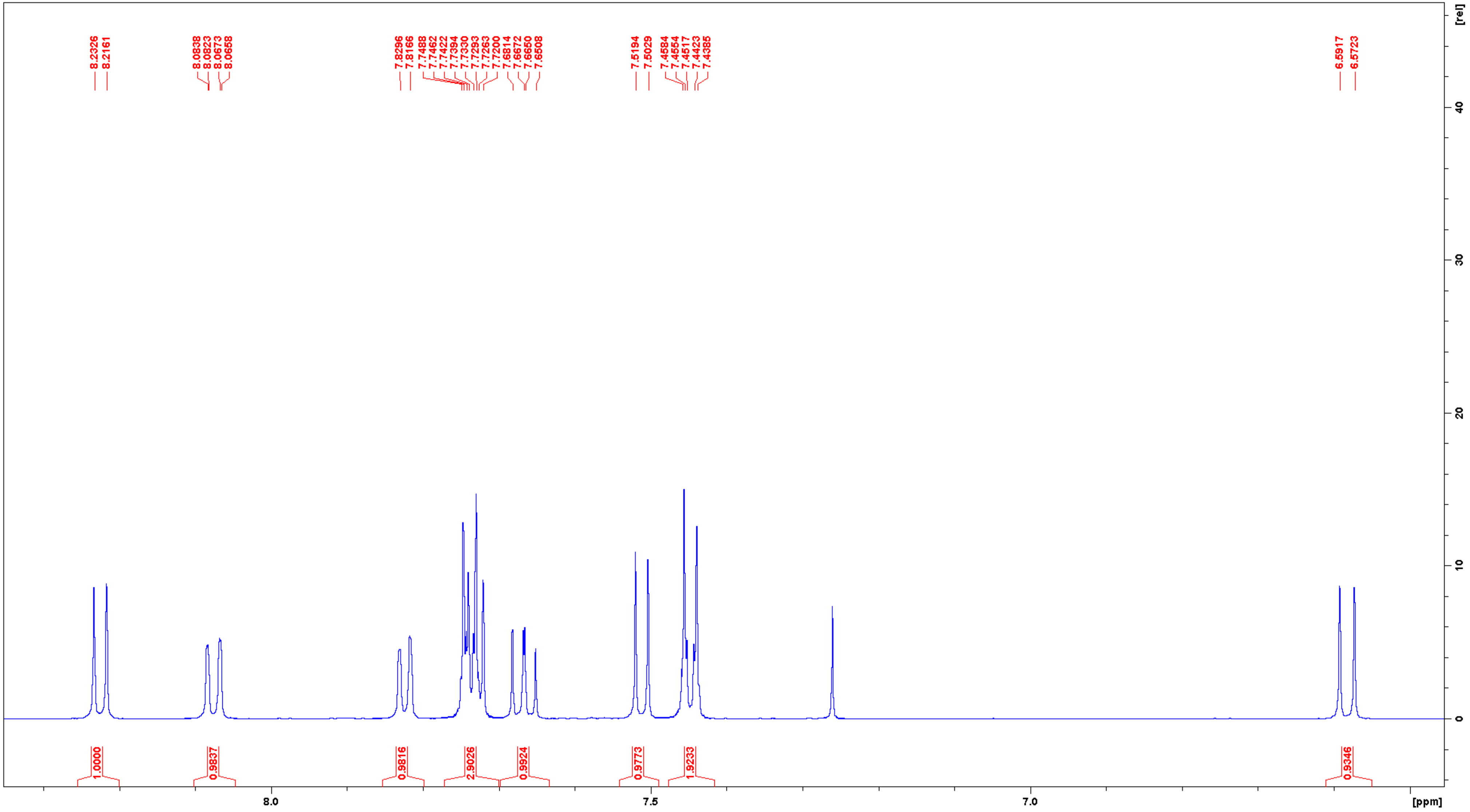

^{13}C NMR spectrum (125 MHz, $CDCl_3$) of compound **10**:

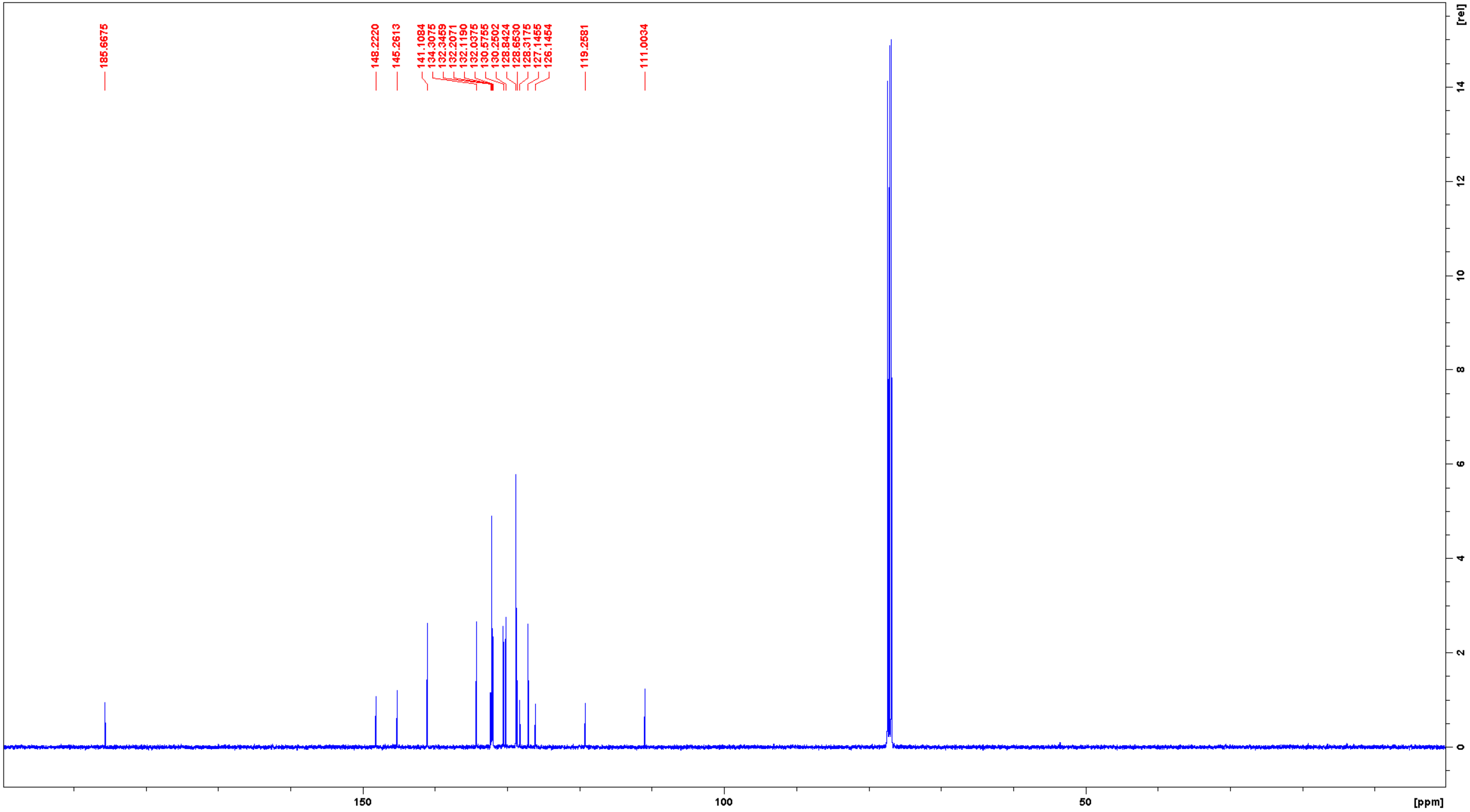

Expanded ^{13}C NMR spectrum (125 MHz, $CDCl_3$) of compound **10**:

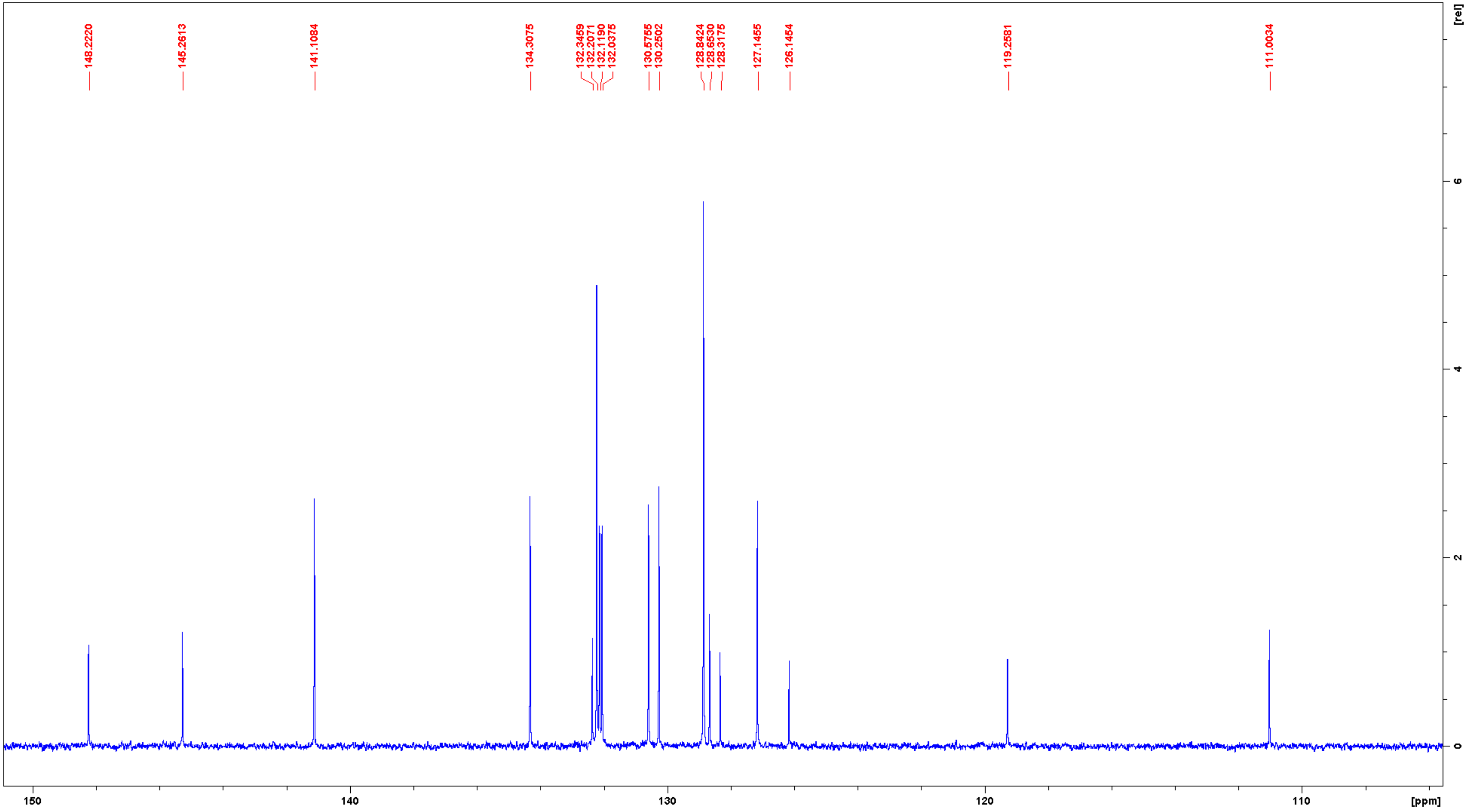

^{1}H NMR spectrum (500 MHz, $CDCl_3$) of compound **11**:

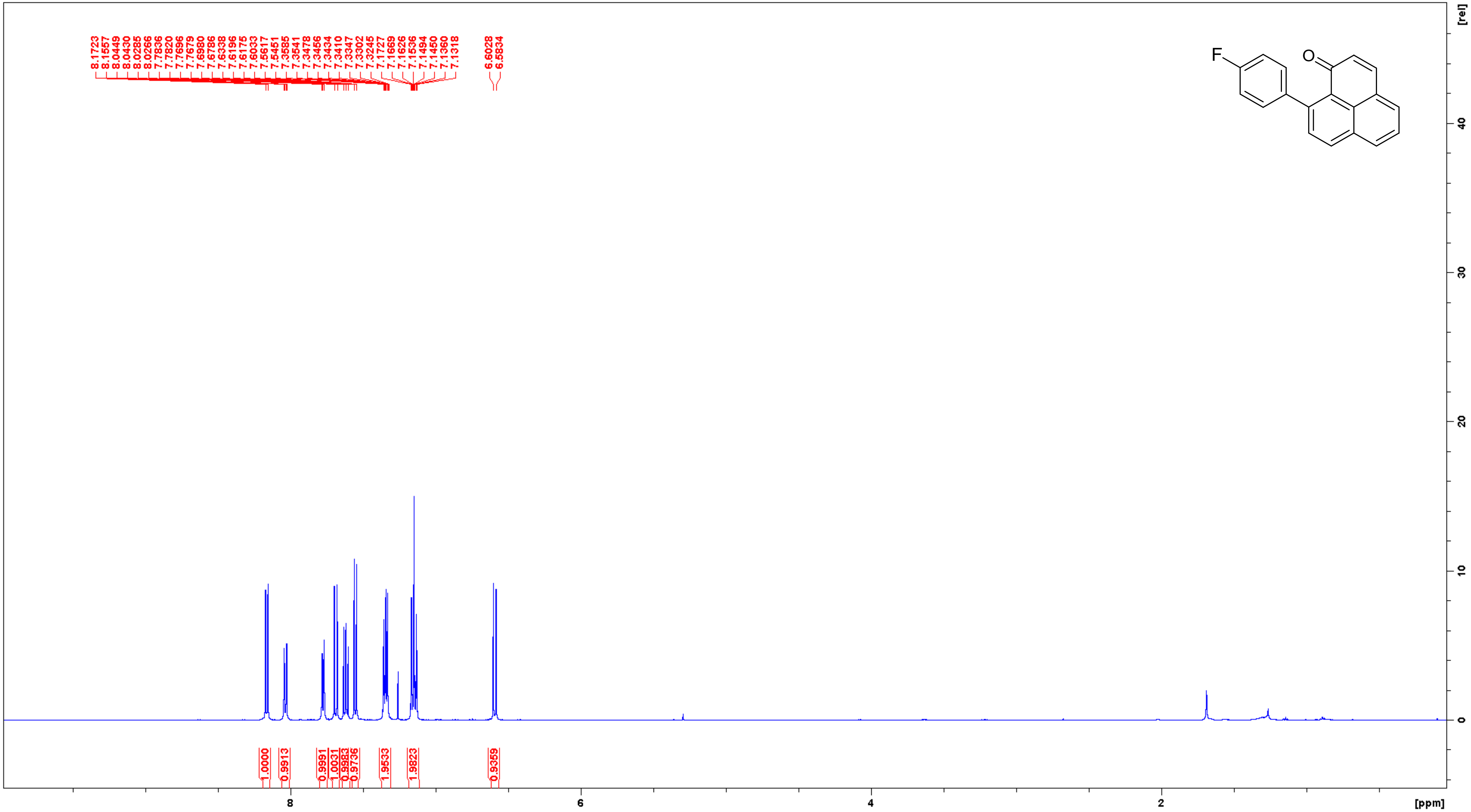

Expanded ^{1}H NMR spectrum (500 MHz, $CDCl_3$) of compound **11**:

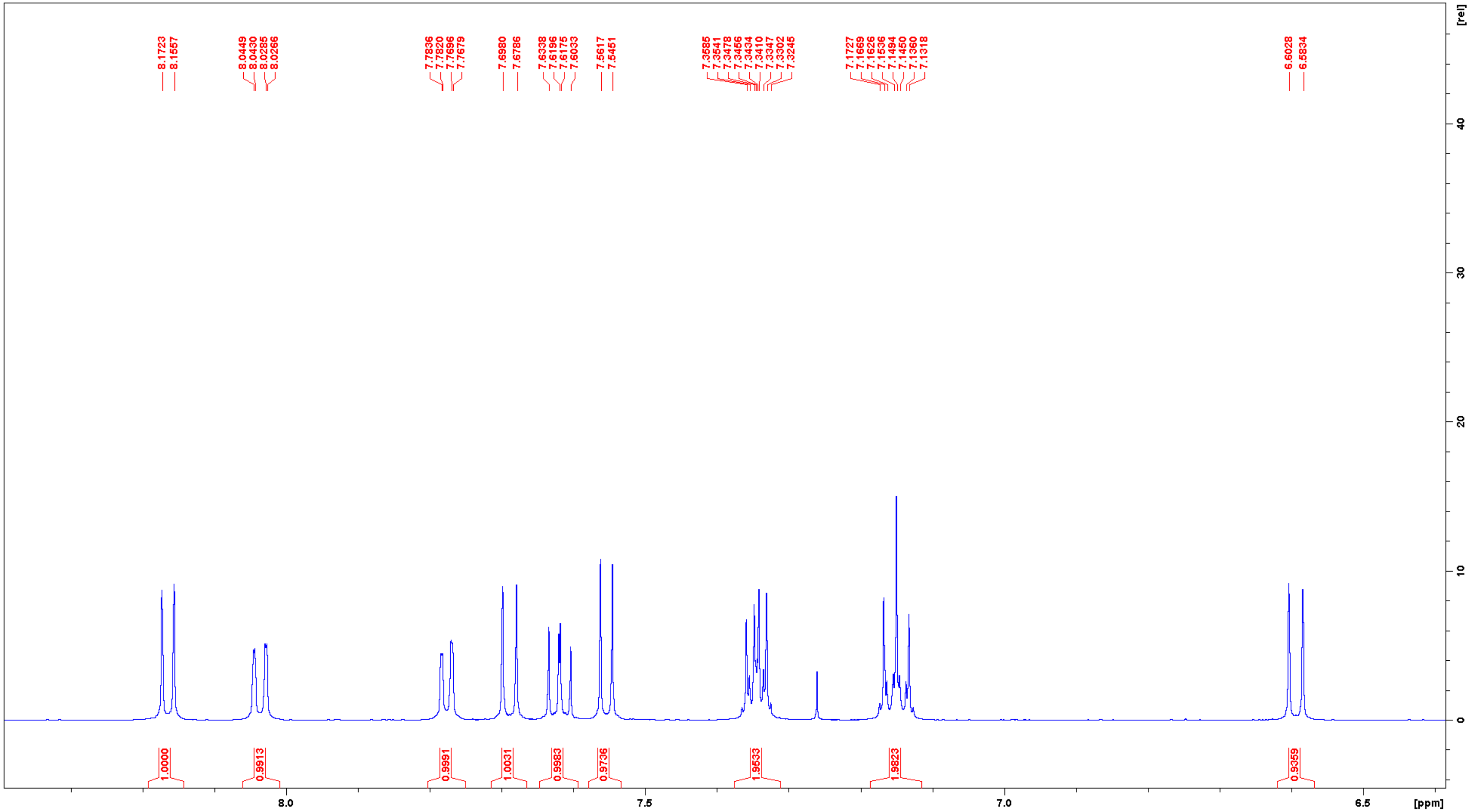

^{13}C NMR spectrum (125 MHz, $CDCl_3$) of compound **11**:

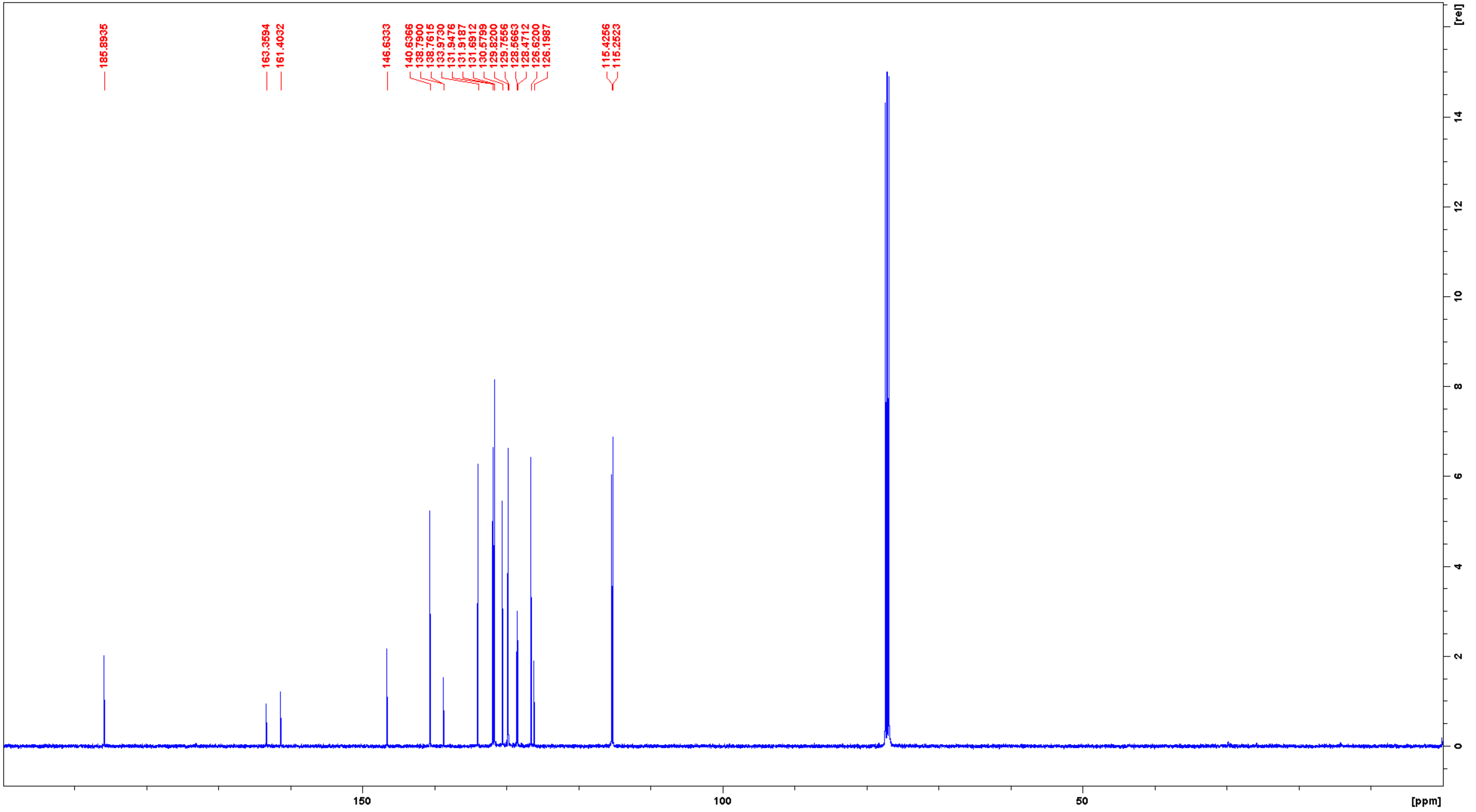

Expanded ^{13}C NMR spectrum (125 MHz, $CDCl_3$) of compound **11**:

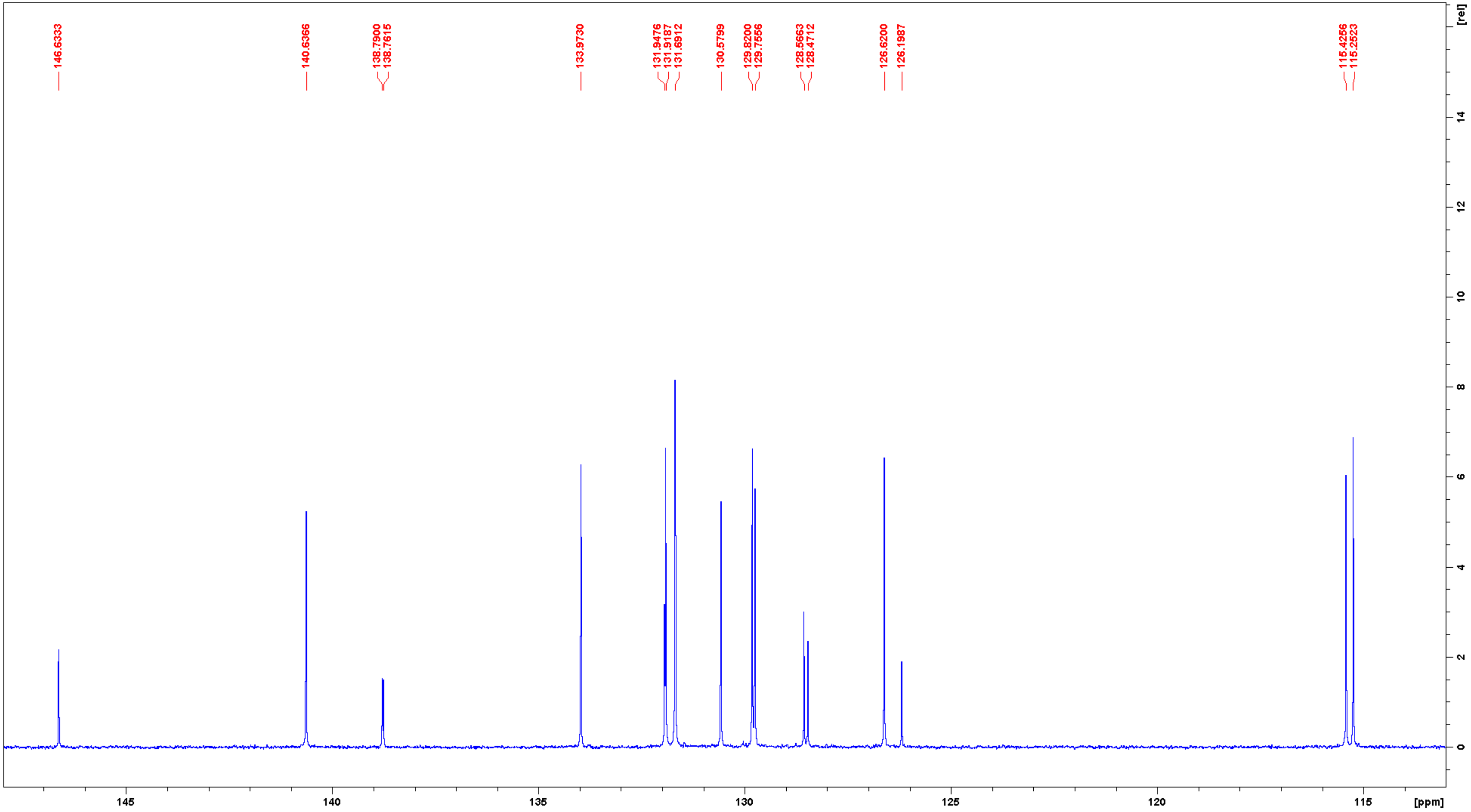

3. ^{19}F NMR spectrum (470 MHz, $CDCl_3$) of 11:

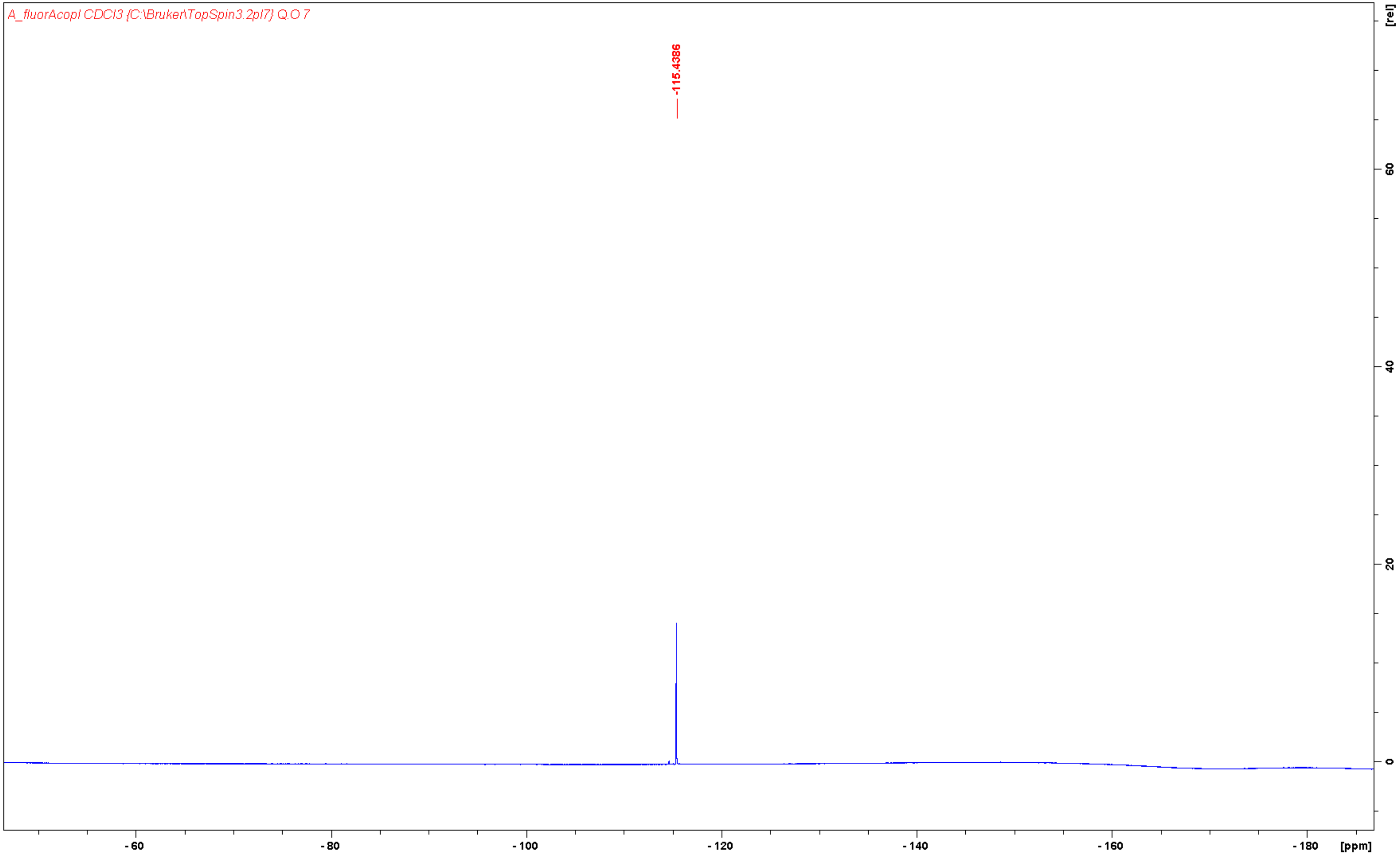

^{1}H NMR spectrum (400 MHz, $CDCl_3$) of compound **12**:

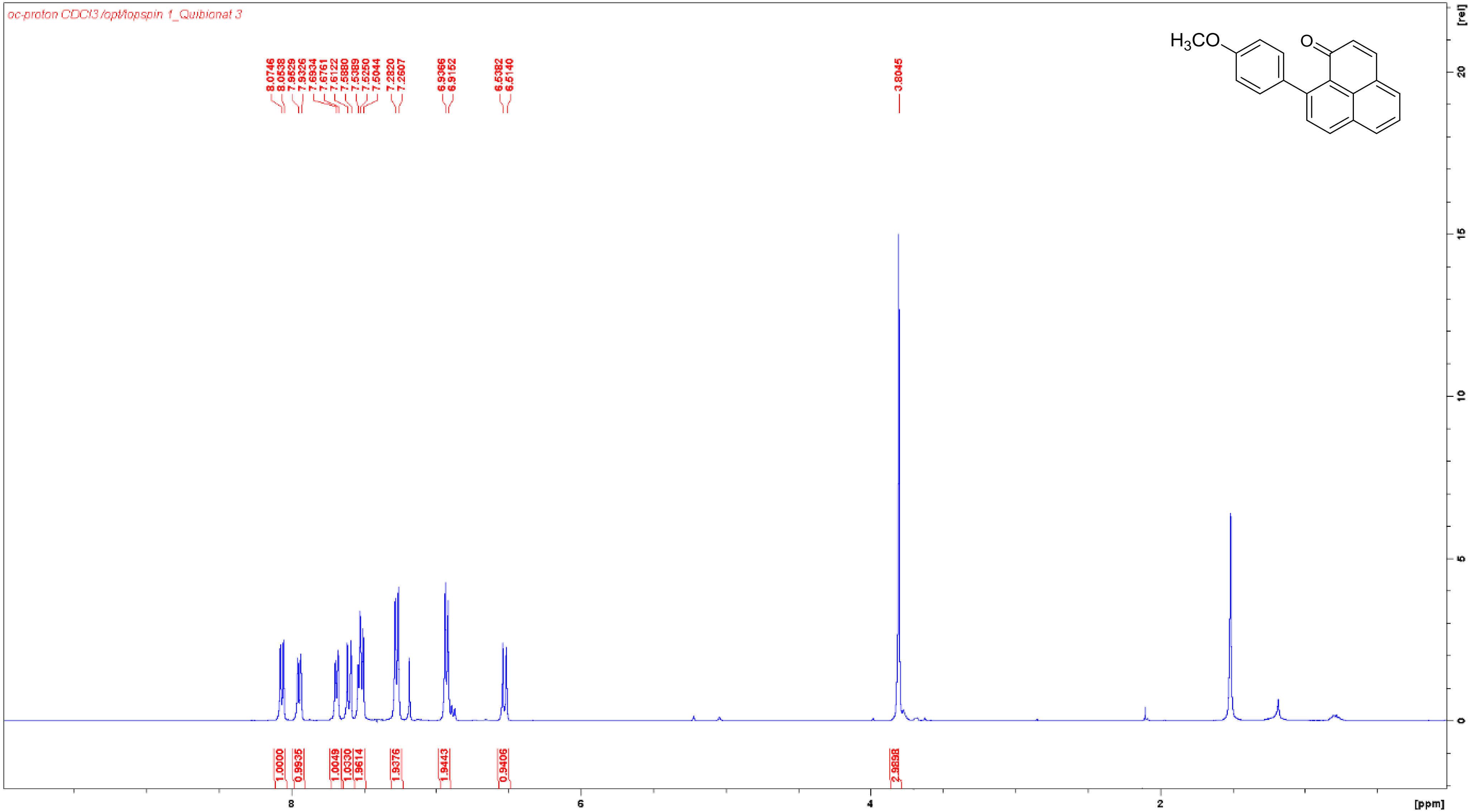

^{13}C NMR spectrum (100 MHz, $CDCl_3$) of compound **12**:

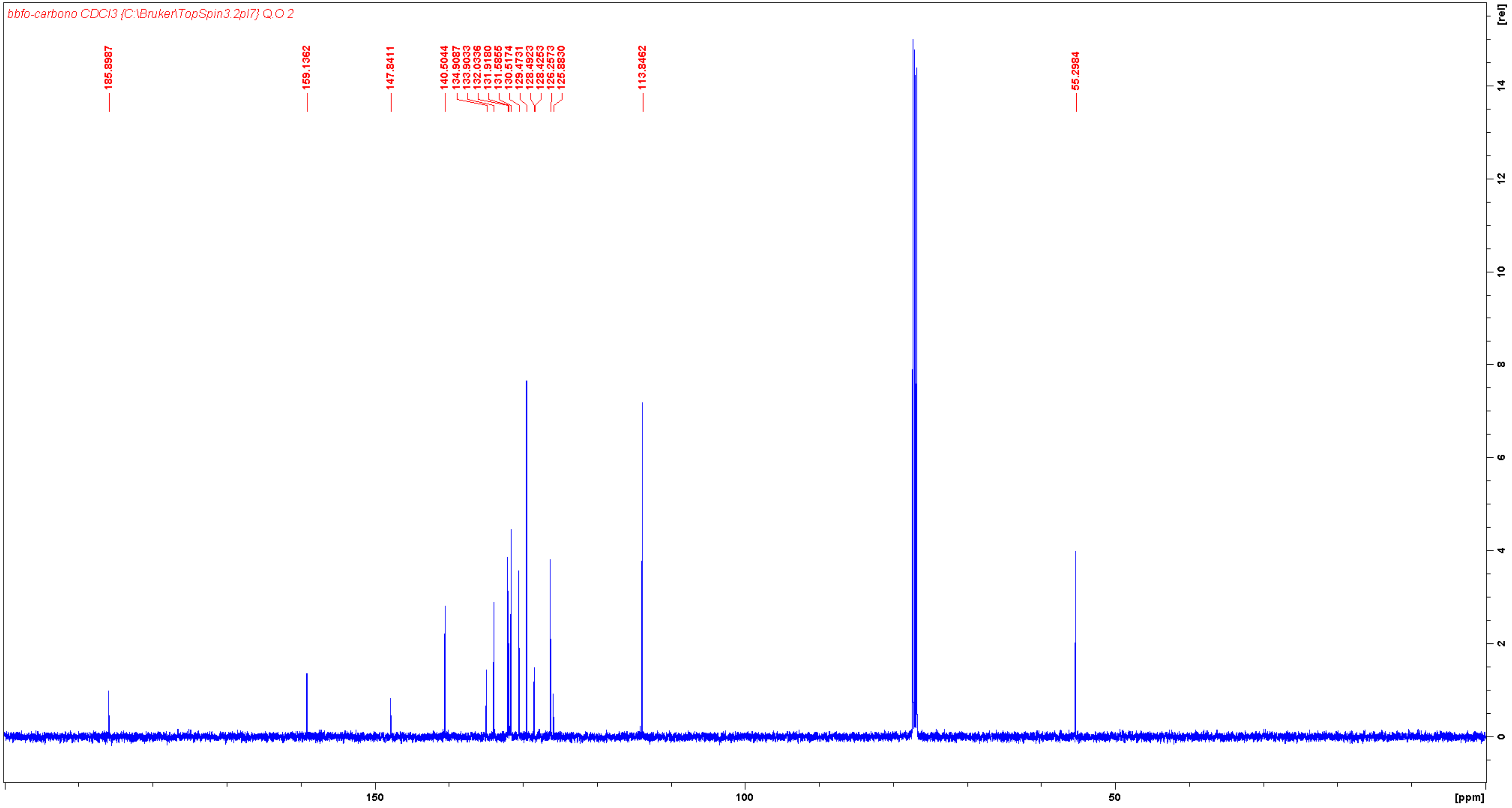

^1H NMR spectrum (500 MHz, $CDCl_3$) of compound **13**:

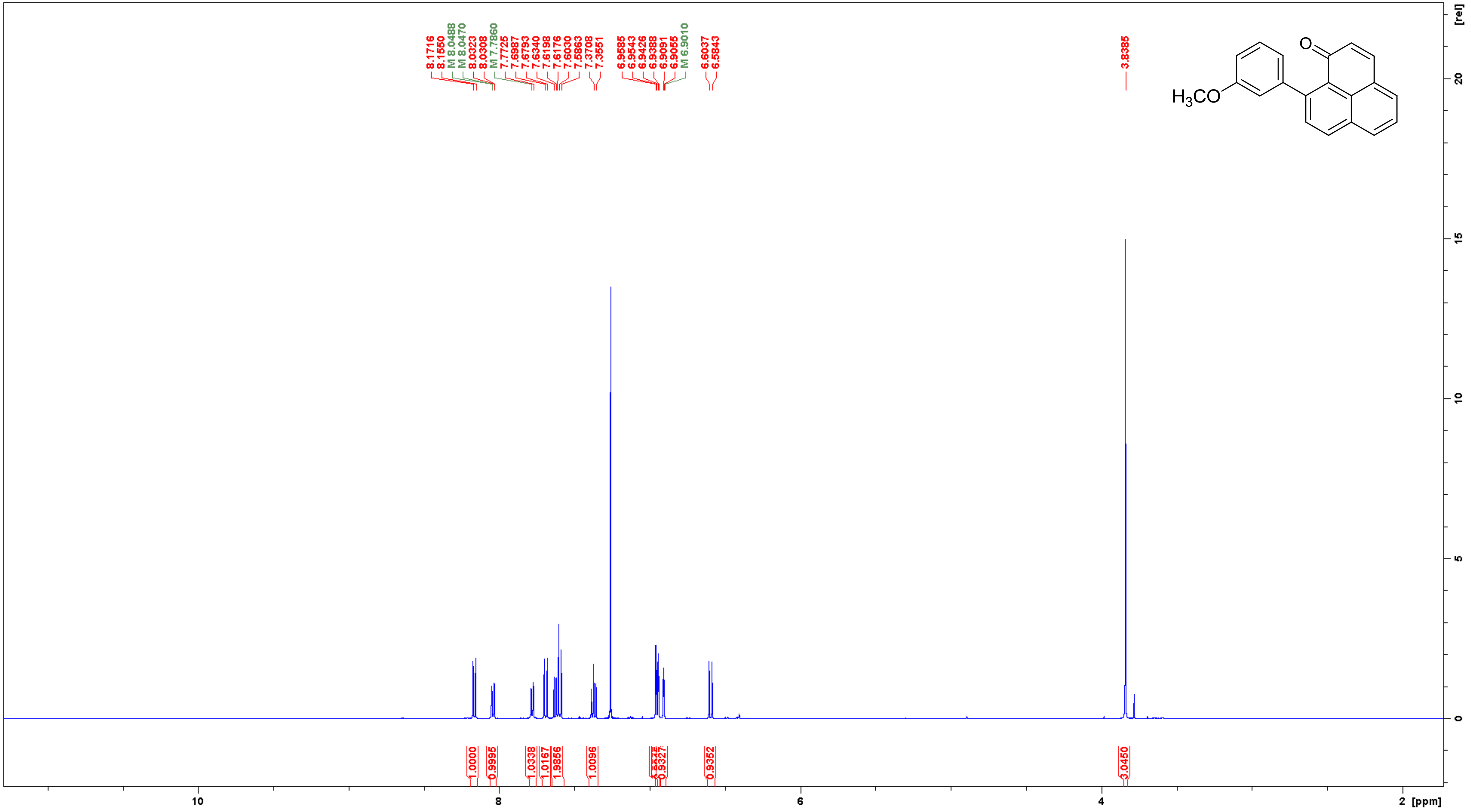

Expanded ^{1}H NMR spectrum (500 MHz, $CDCl_3$) of compound **13**:

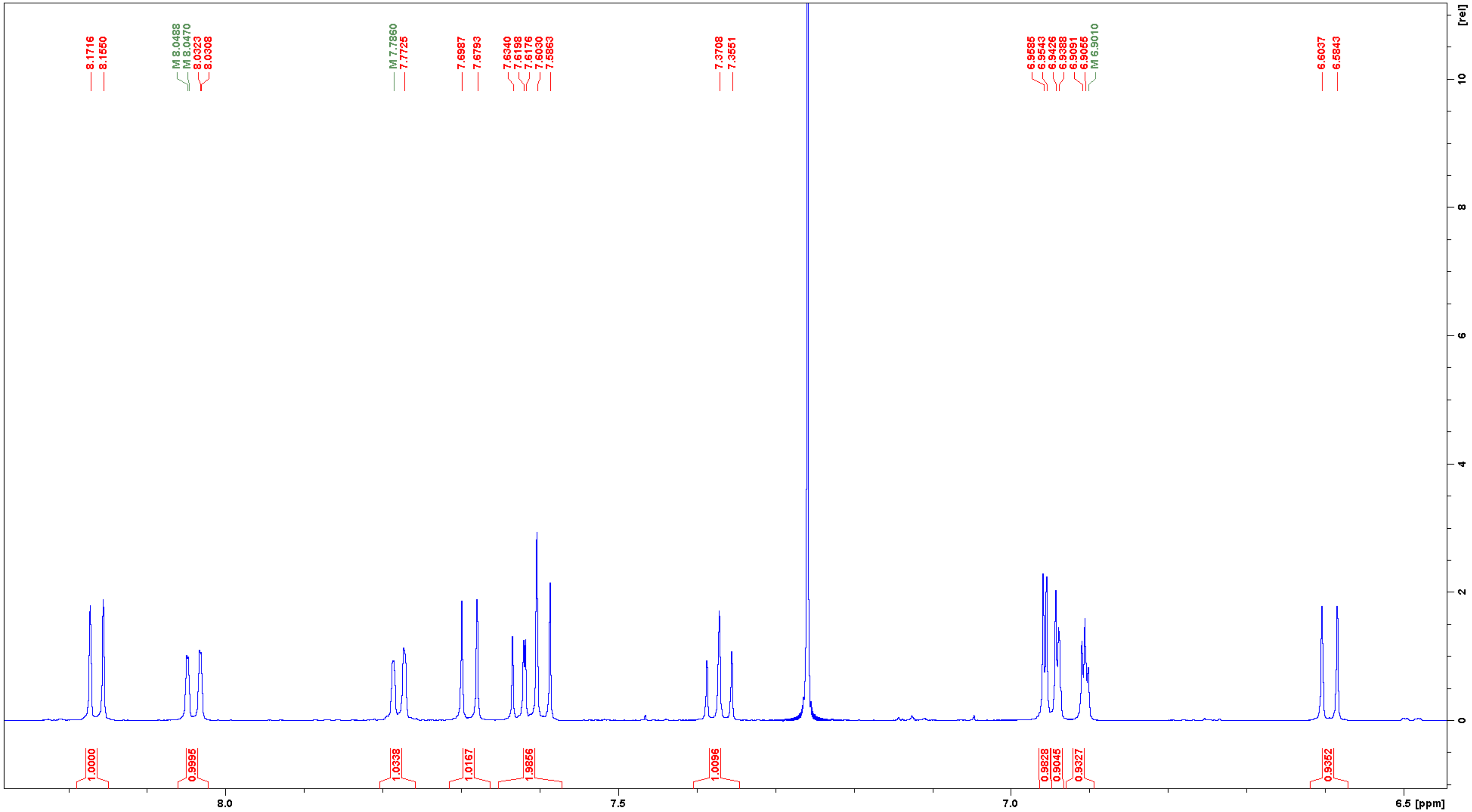

^{13}C NMR spectrum (125 MHz, $CDCl_3$) of compound **13**:

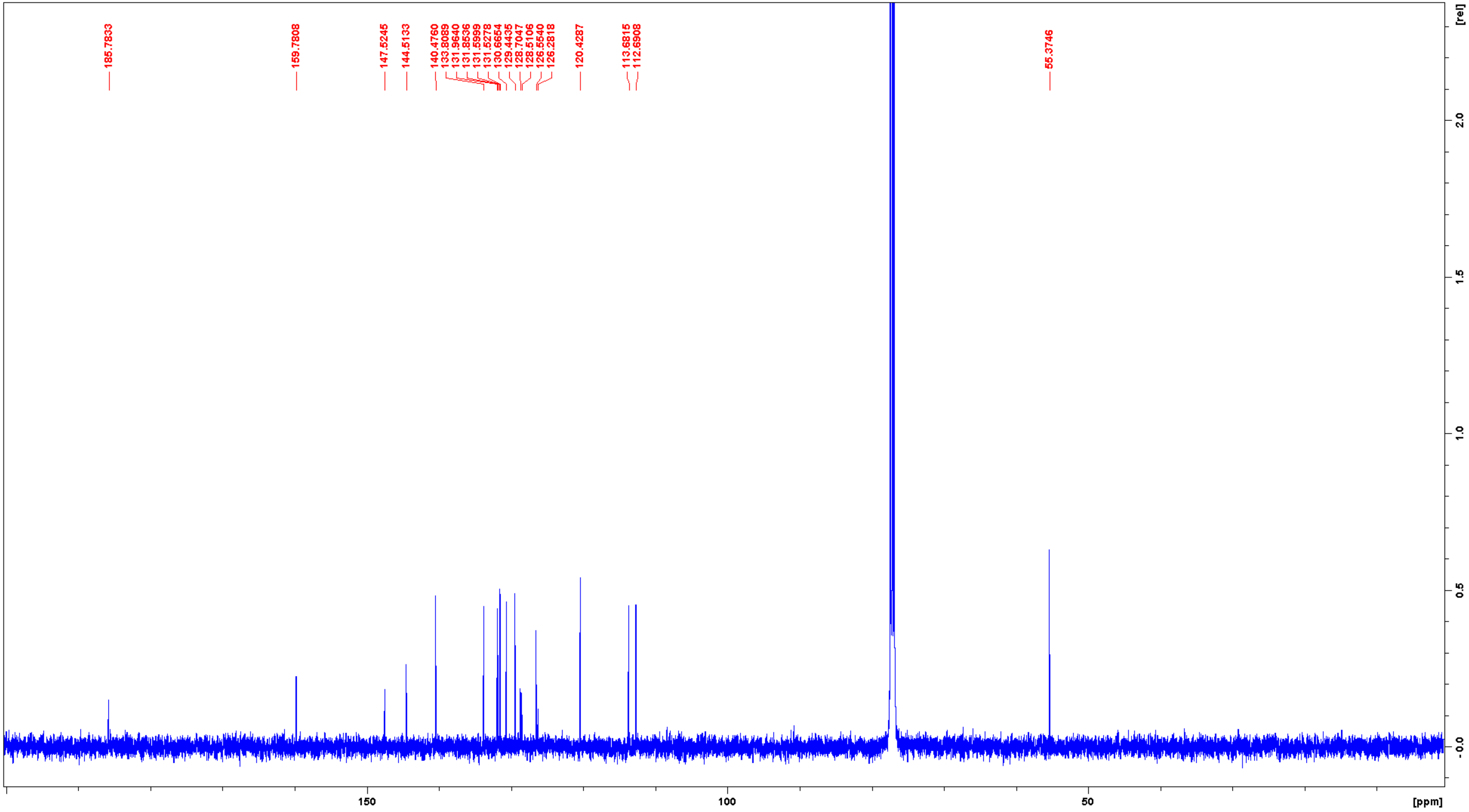

^{1}H NMR spectrum (500 MHz, $CDCl_3$) of compound **14**:

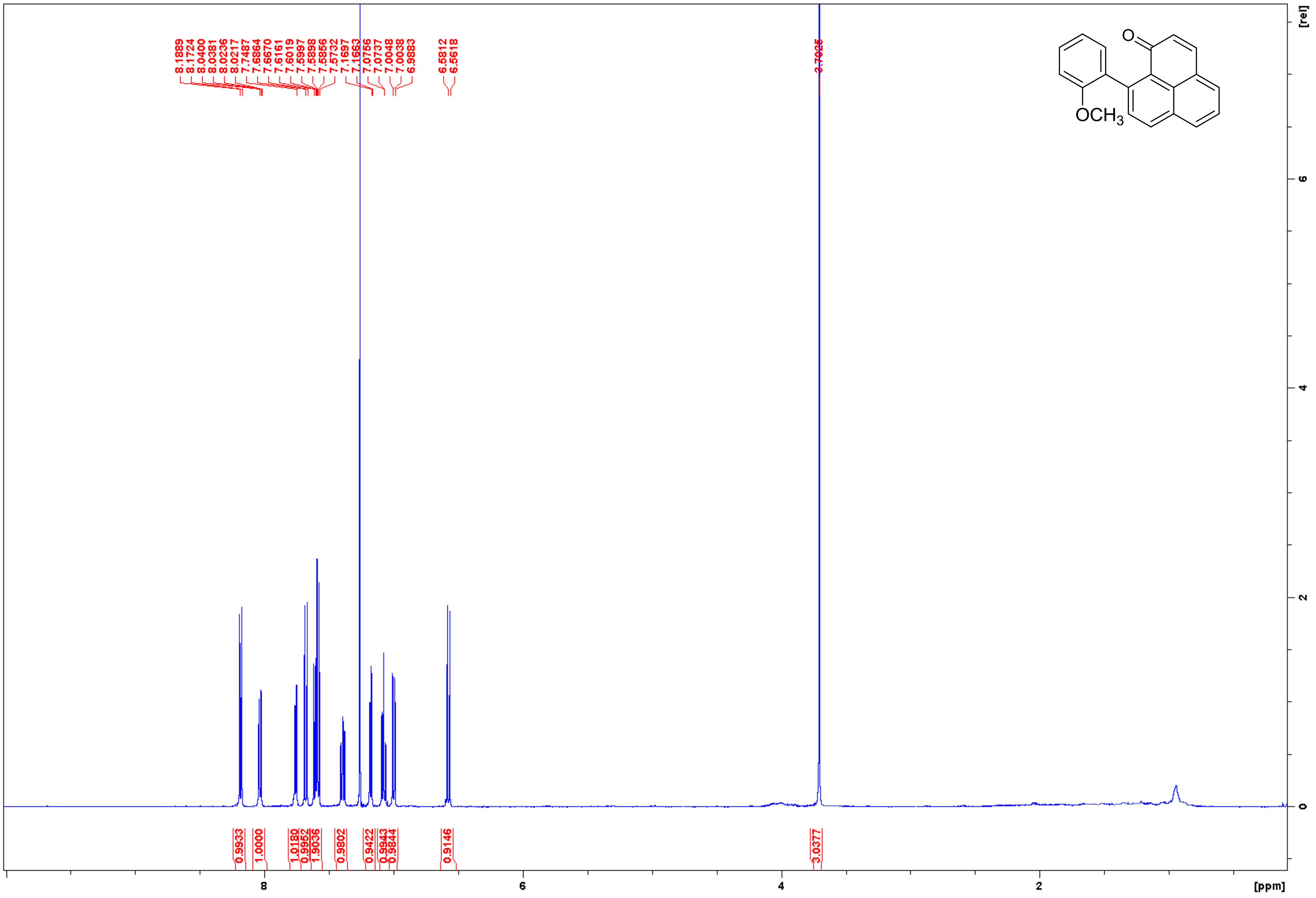

Expanded ^{1}H NMR spectrum (500 MHz, $CDCl_3$) of compound **14**:

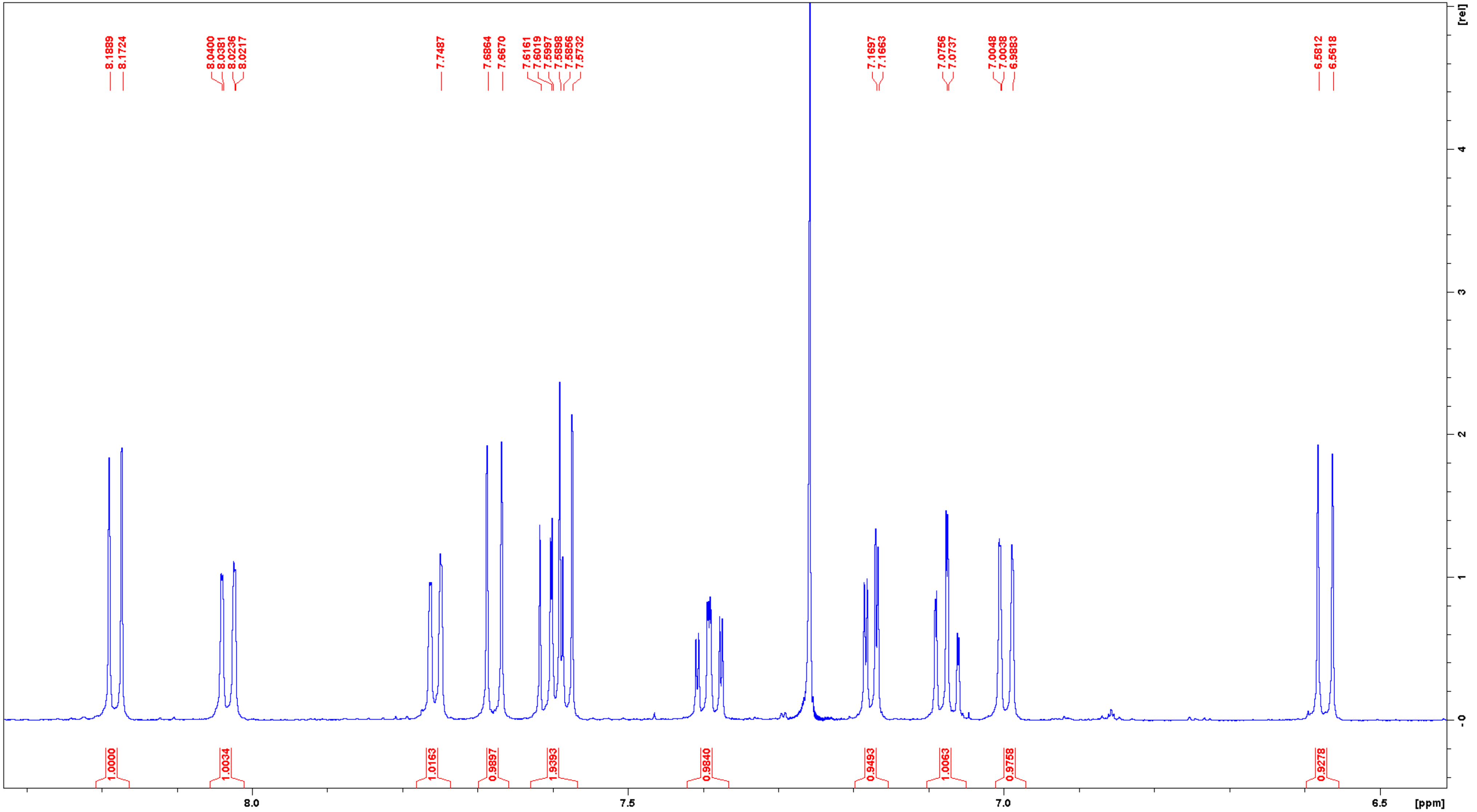

^{13}C NMR spectrum (125 MHz, $CDCl_3$) of compound **14**:

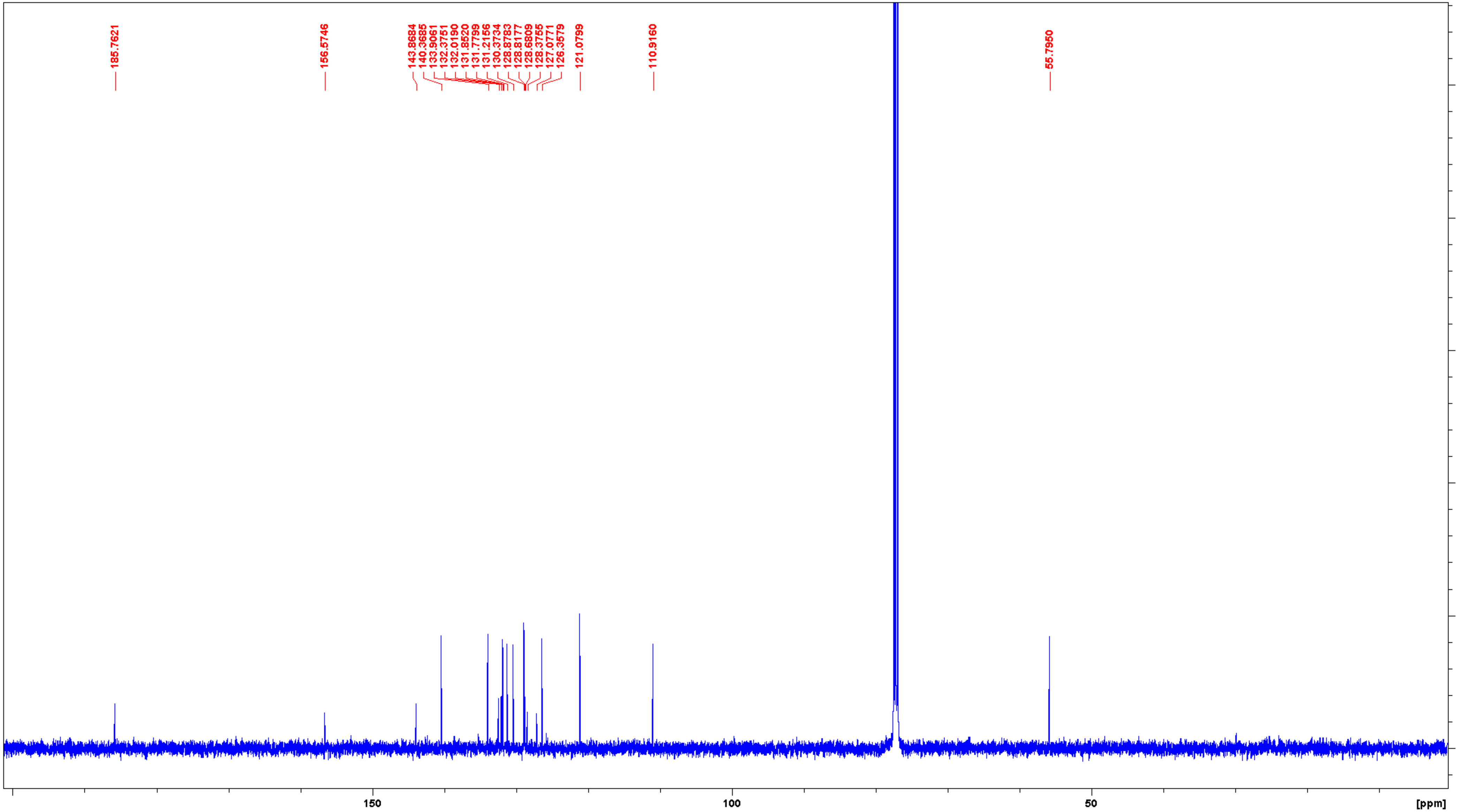

Expanded ^{13}C NMR spectrum (125 MHz, $CDCl_3$) of compound **14**:

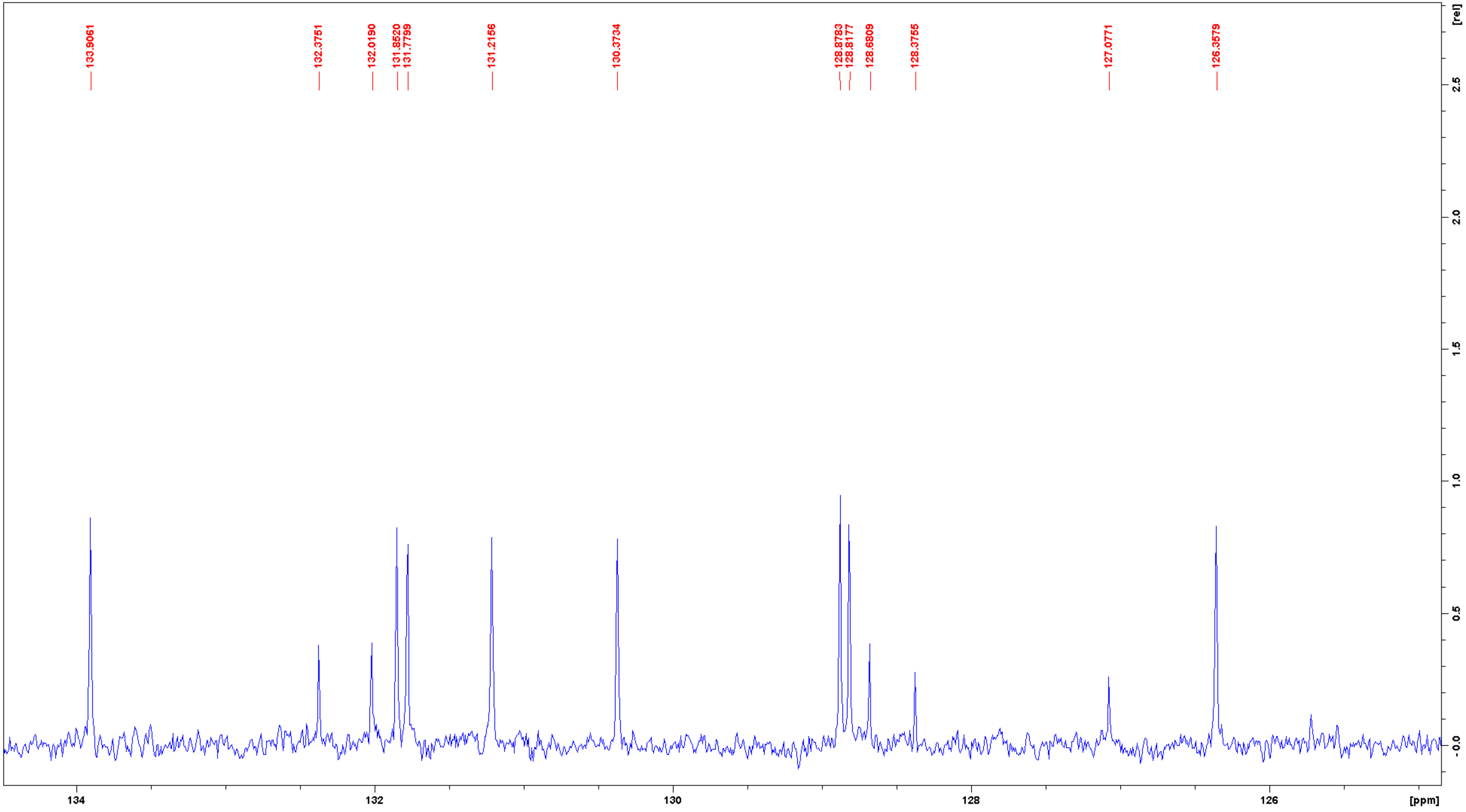

^{1}H NMR spectrum (500 MHz, $CDCl_3$) of compound **15**:

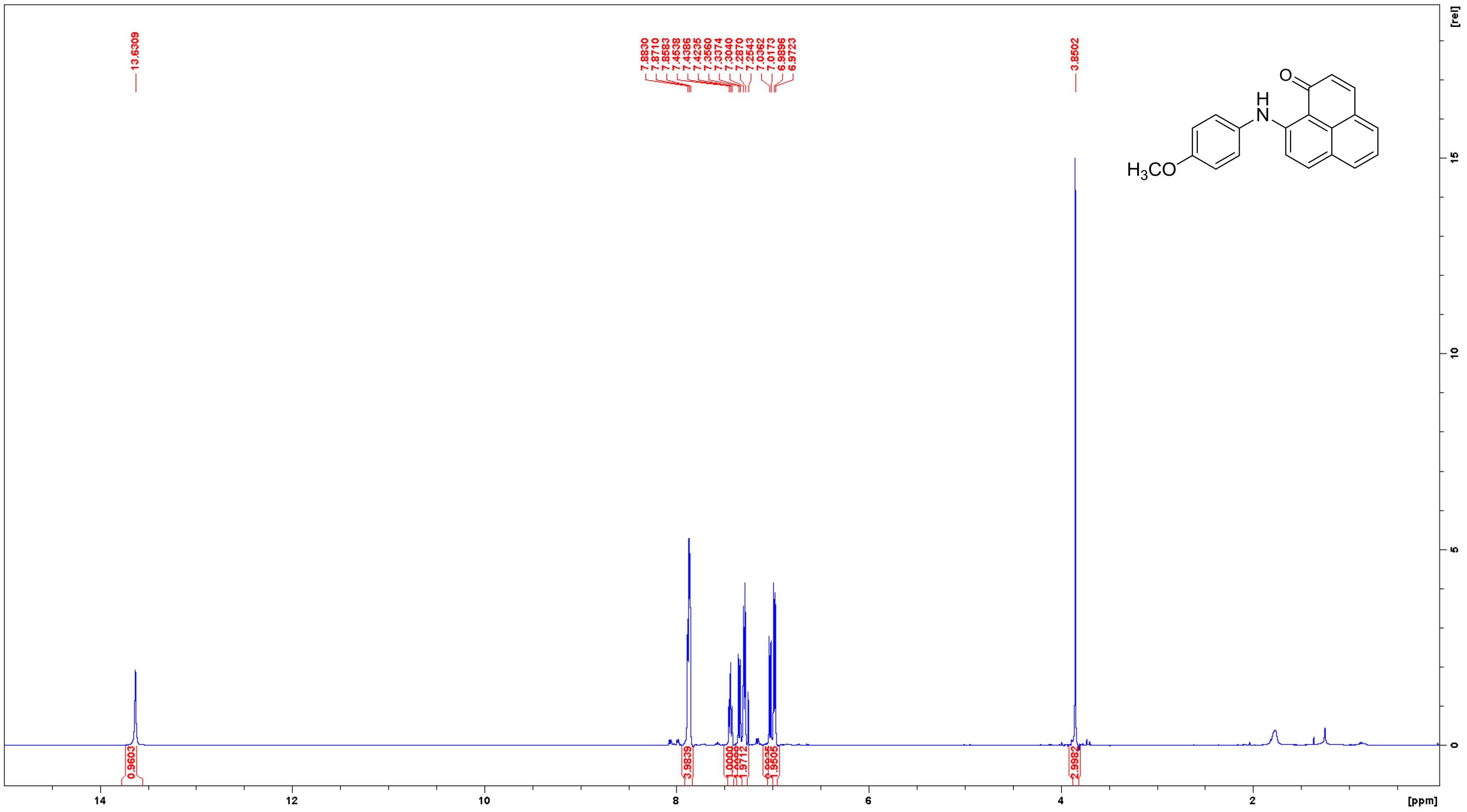

^{1}H NMR spectrum (500 MHz, $CDCl_3$) of compound **16**:

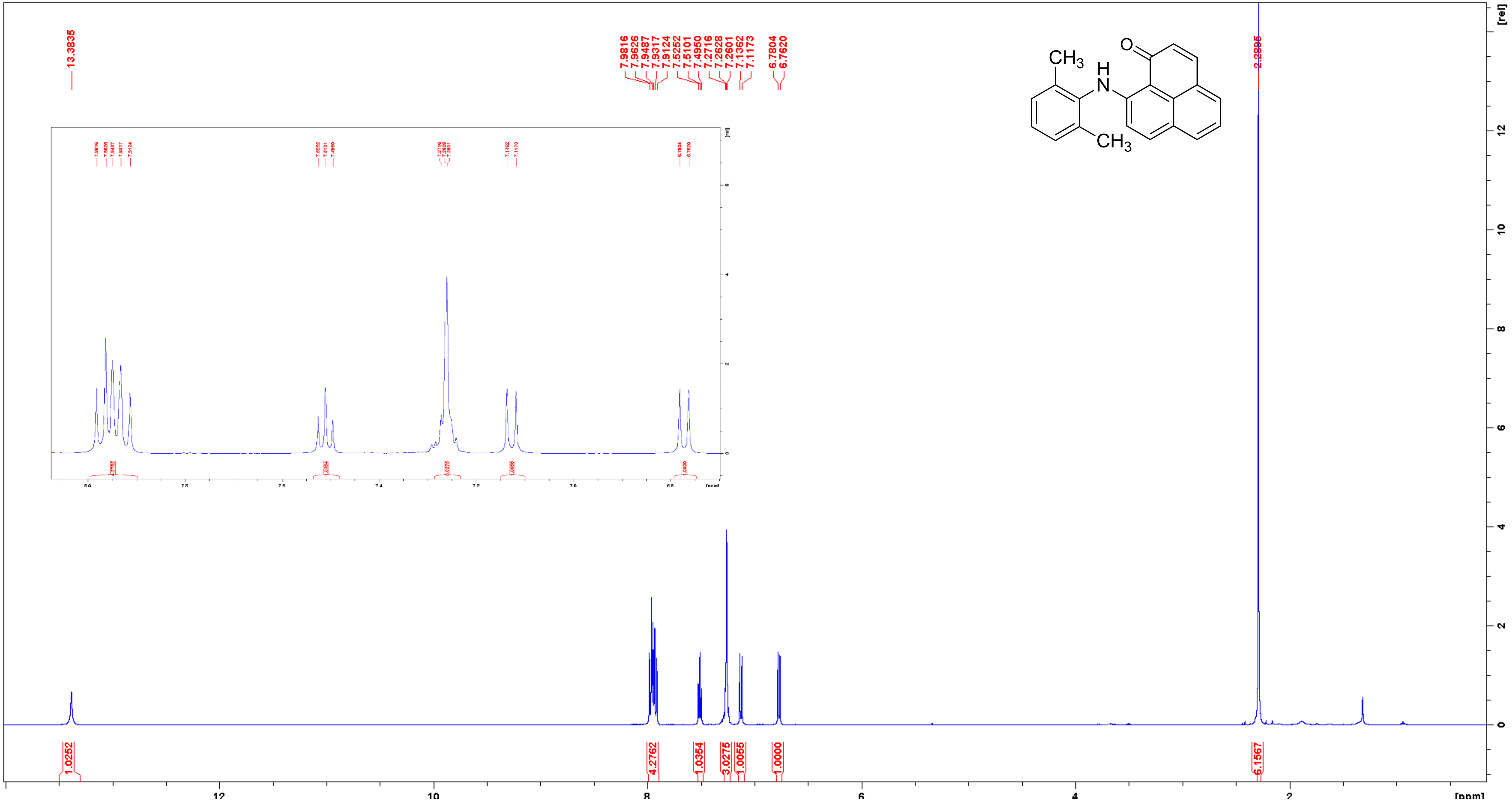

^{13}C NMR spectrum (125 MHz, $CDCl_3$) of compound **16**:

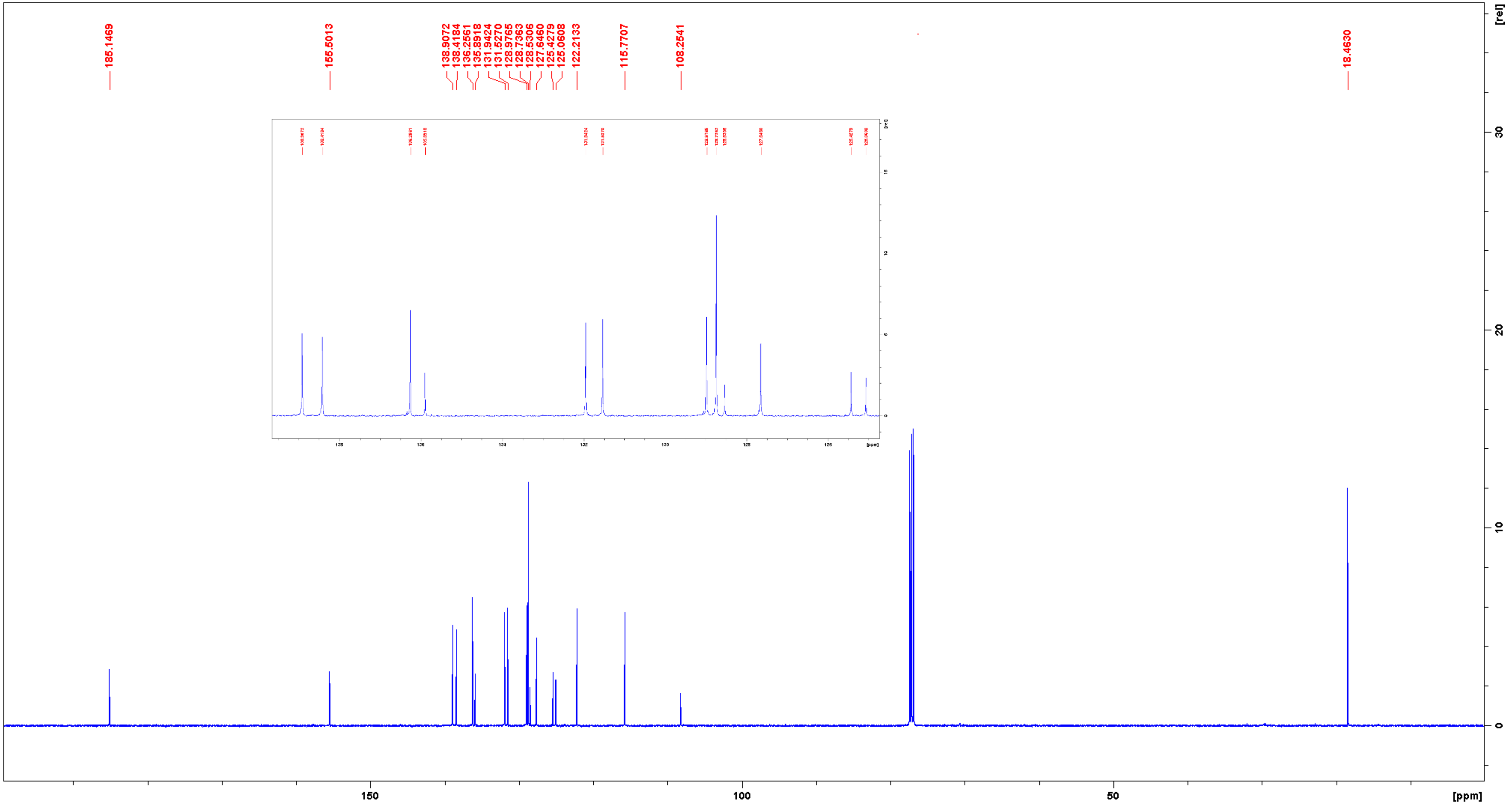

^{1}H NMR spectrum (500 MHz, $CDCl_3$) of compound **17**:

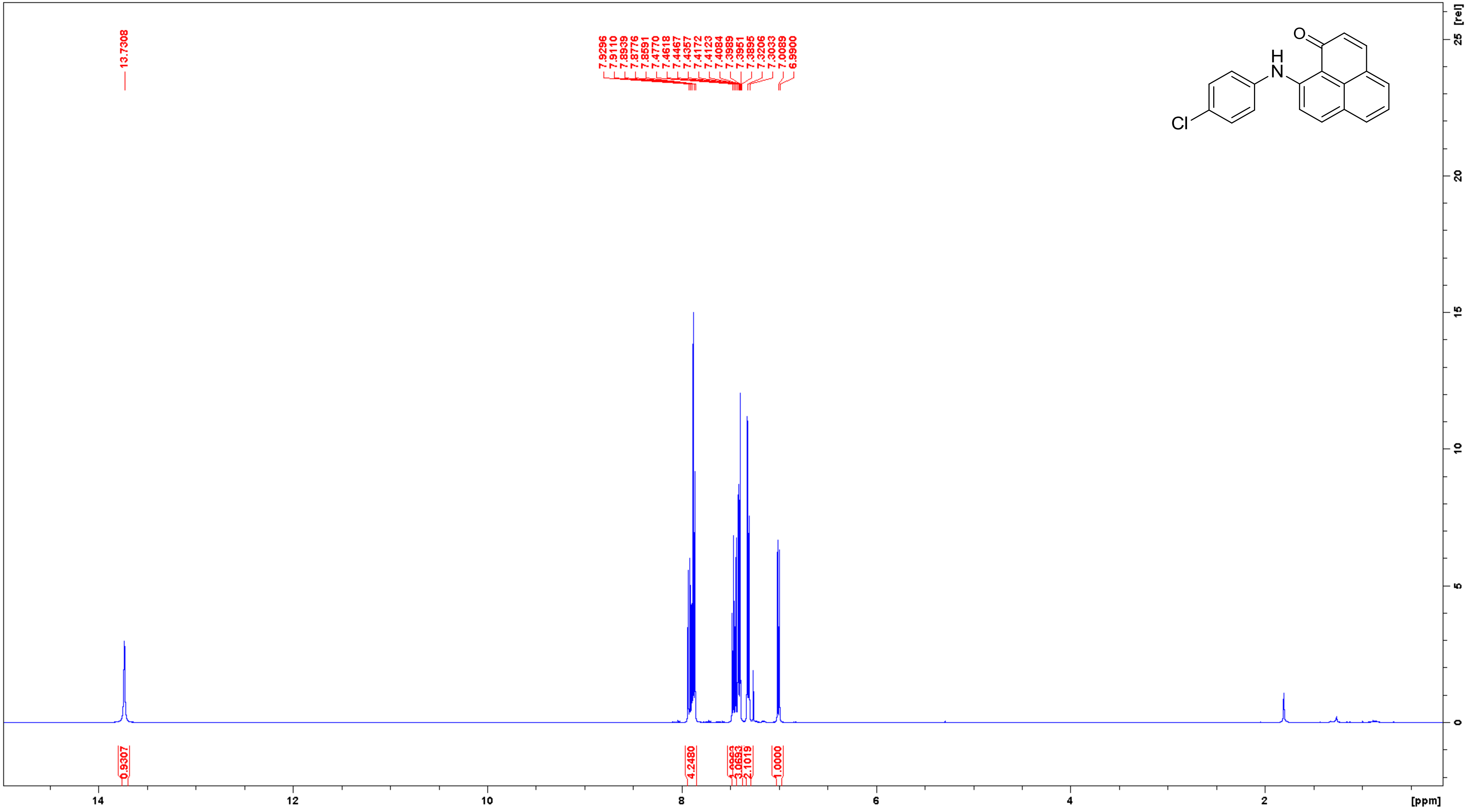

^{13}C NMR spectrum (125 MHz, $CDCl_3$) of compound **17**:

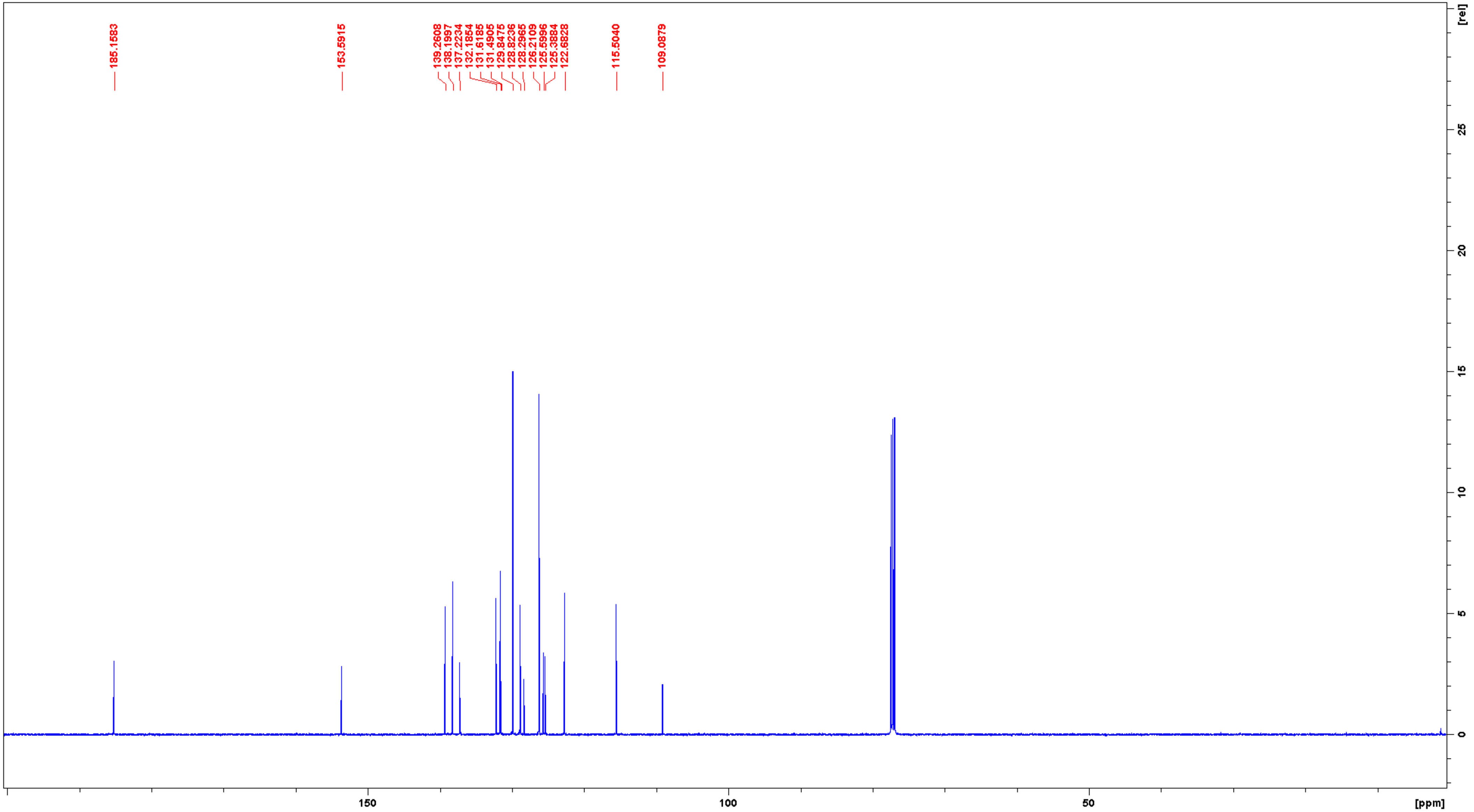

^{1}H NMR spectrum (500 MHz, $CDCl_3$) of compound **18**:

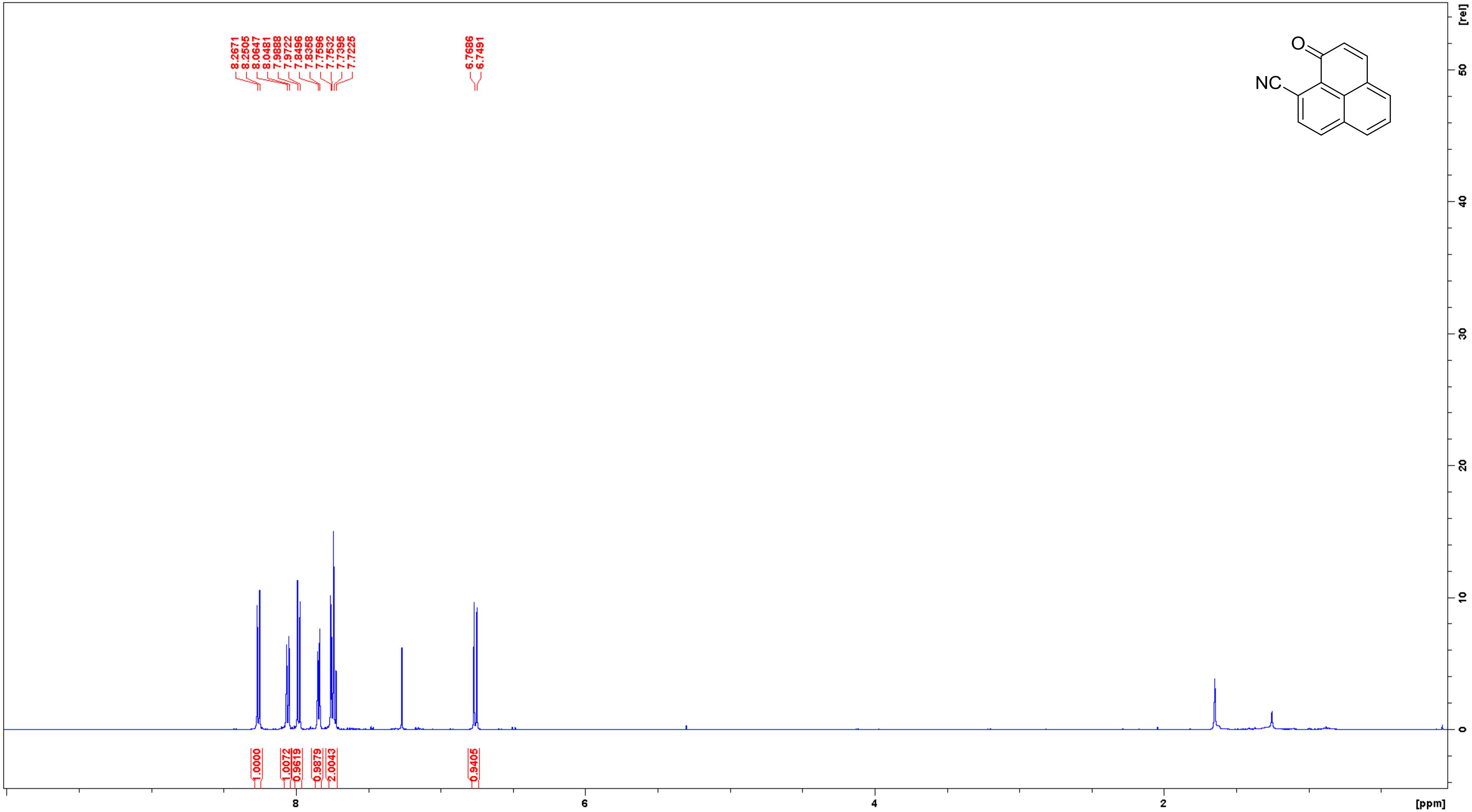

^{1}H NMR spectrum (500 MHz, $CDCl_3$) of compound **19**:

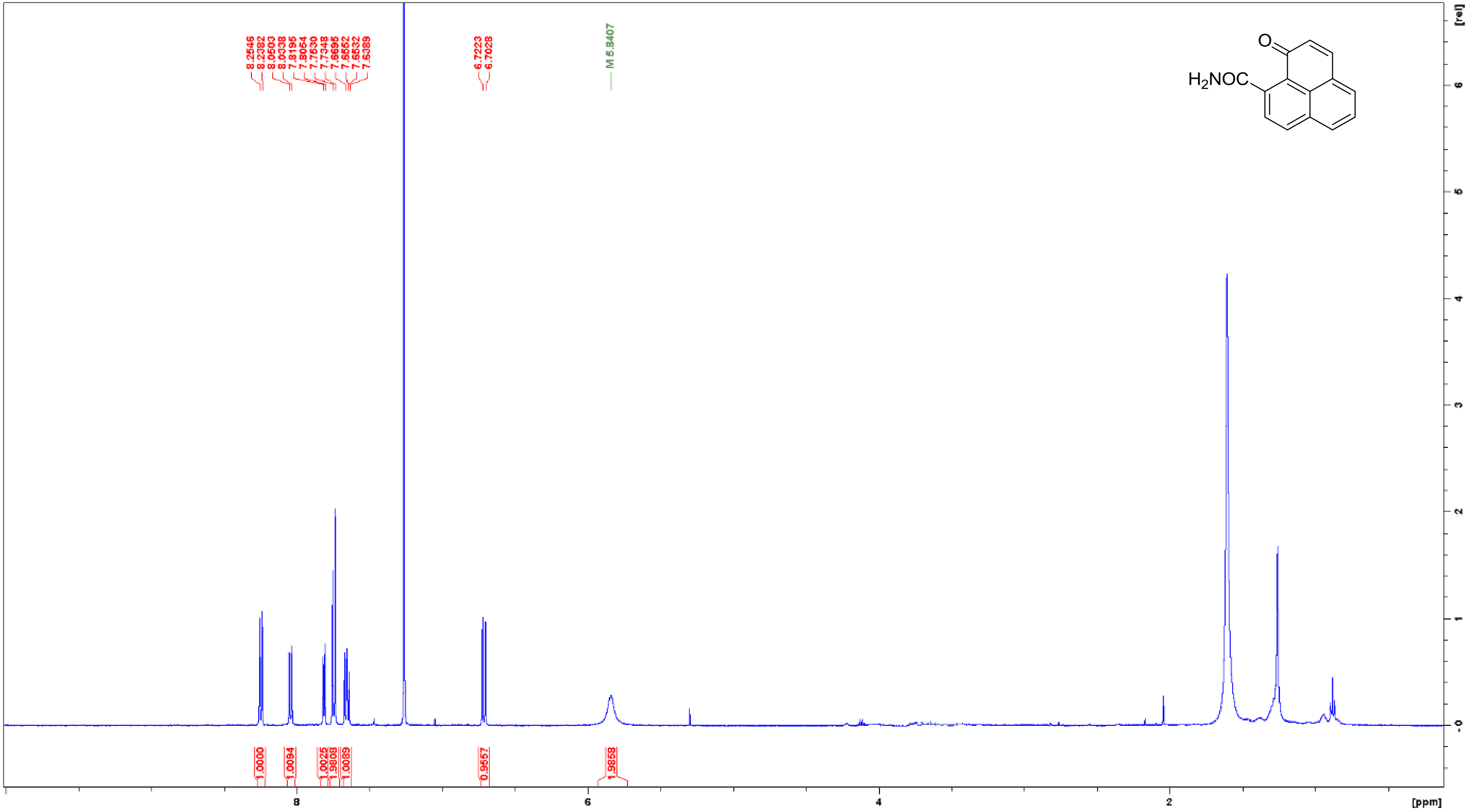

Expanded ^{1}H NMR spectrum (500 MHz, $CDCl_3$) of compound **19**:

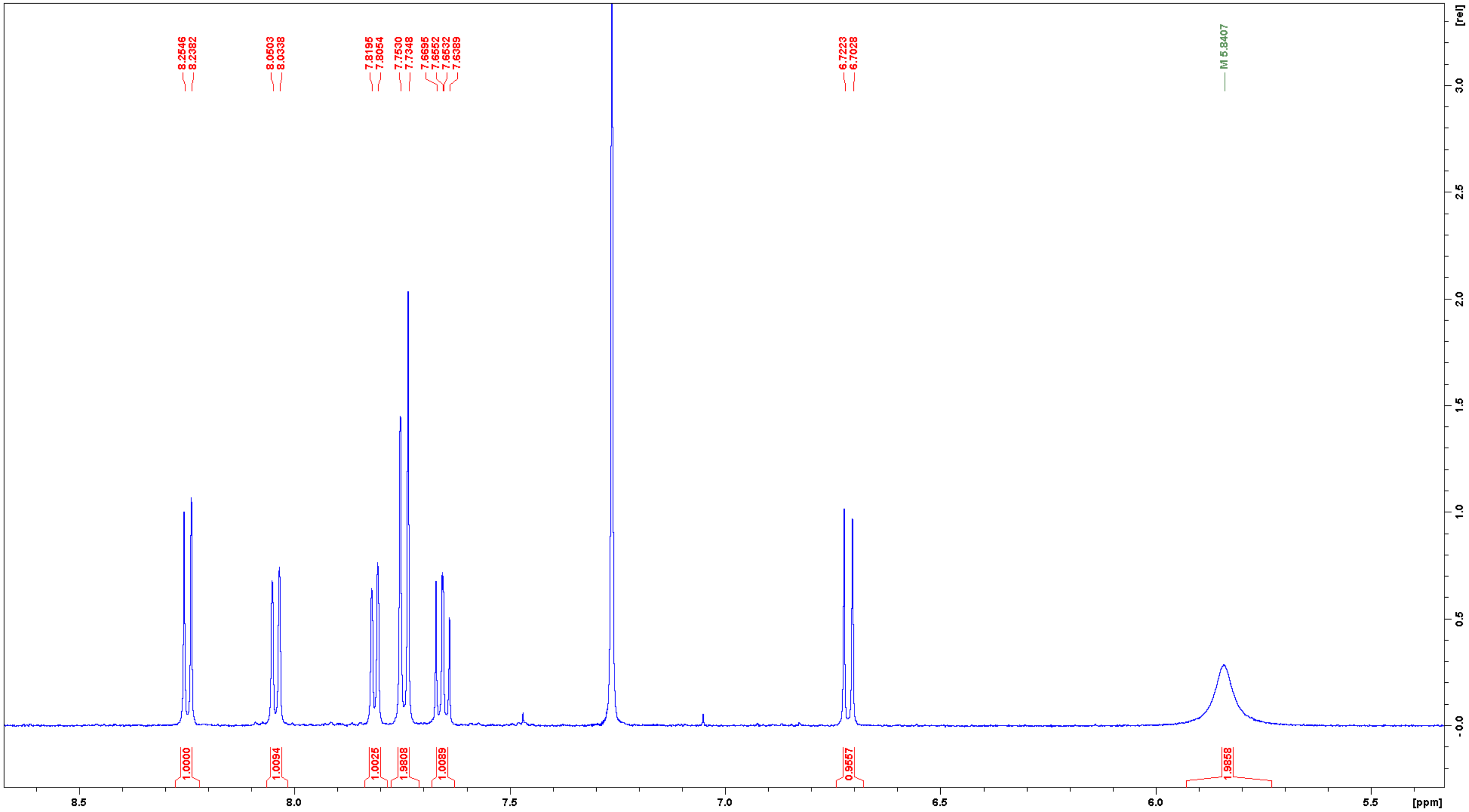

^{13}C NMR spectrum (125 MHz, $CDCl_3$) of compound **19**:

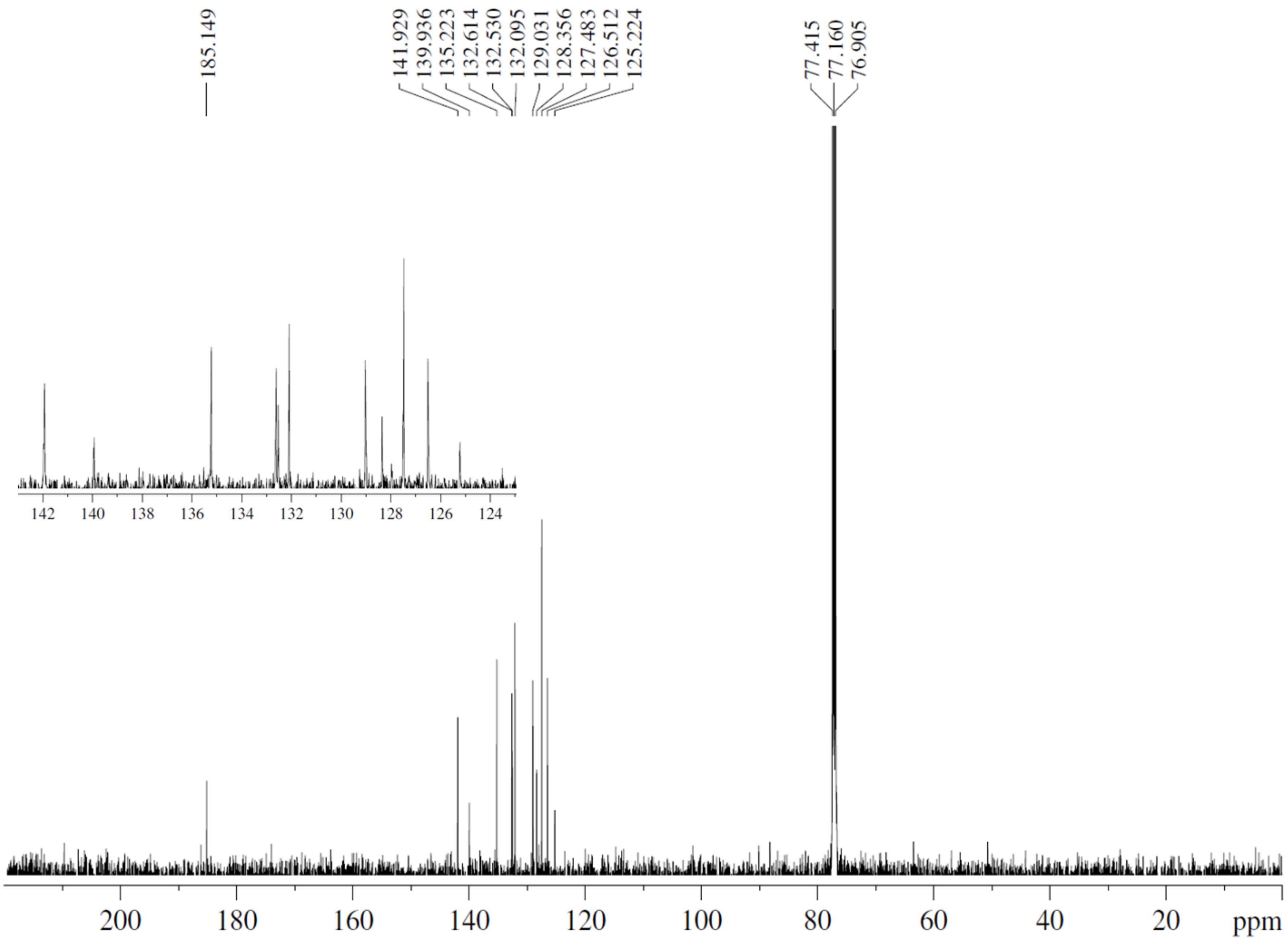

¹H NMR spectrum (500 MHz, $CDCl_3$) of compound **20**:

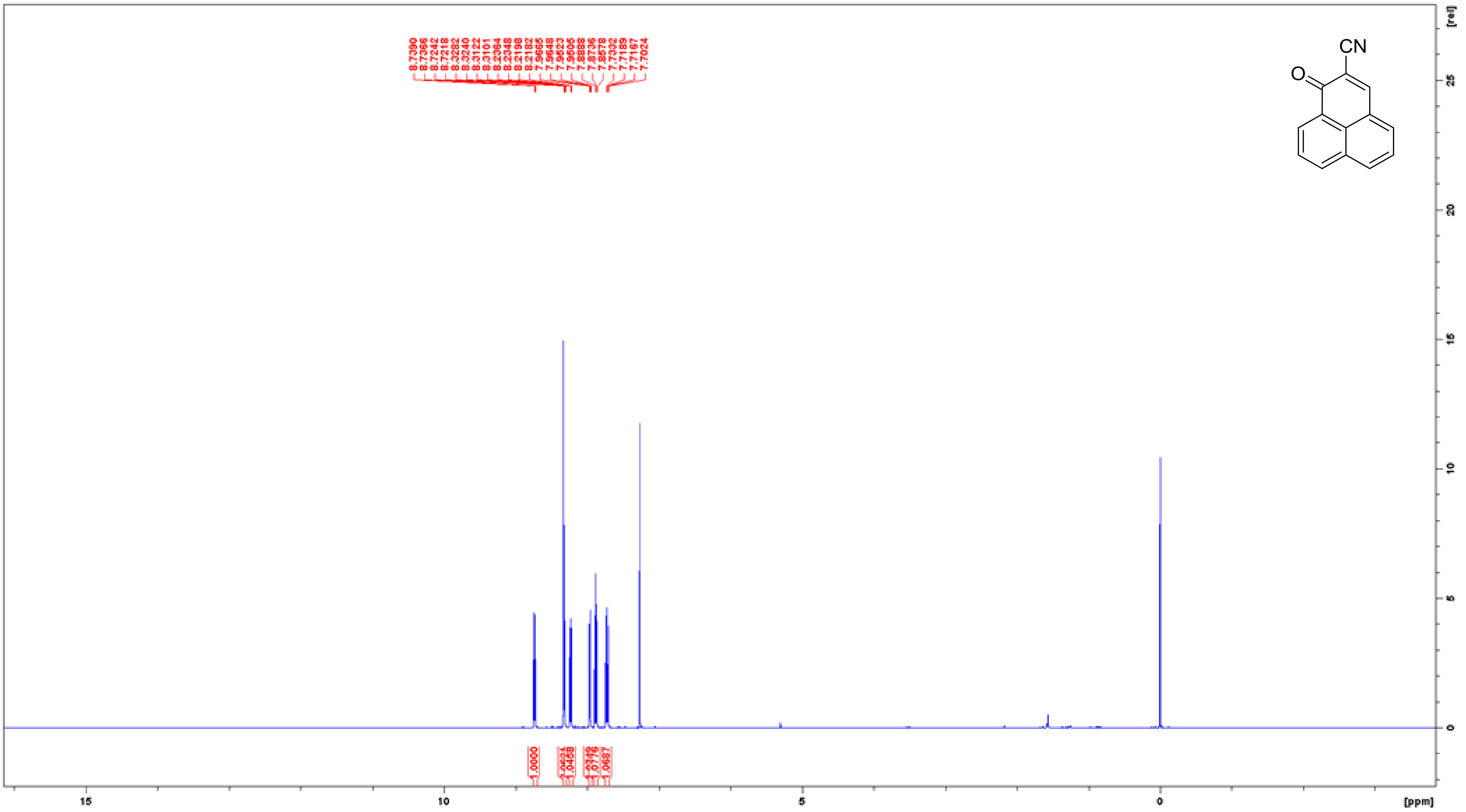

Expanded NMR spectrum (500 MHz, $CDCl_3$) of compound **20**:

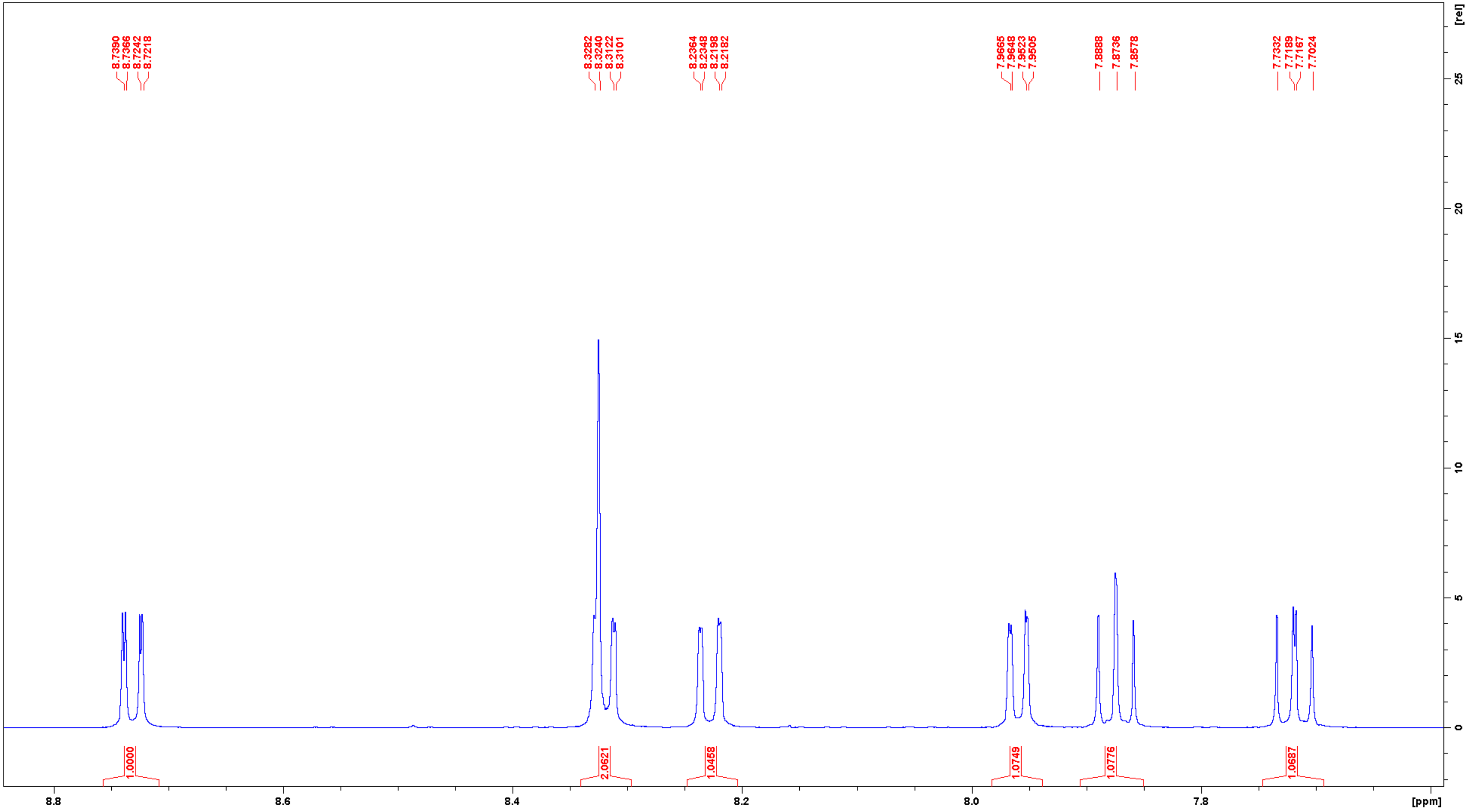

^{13}C NMR spectrum (125 MHz, $CDCl_3$) of compound **20**:

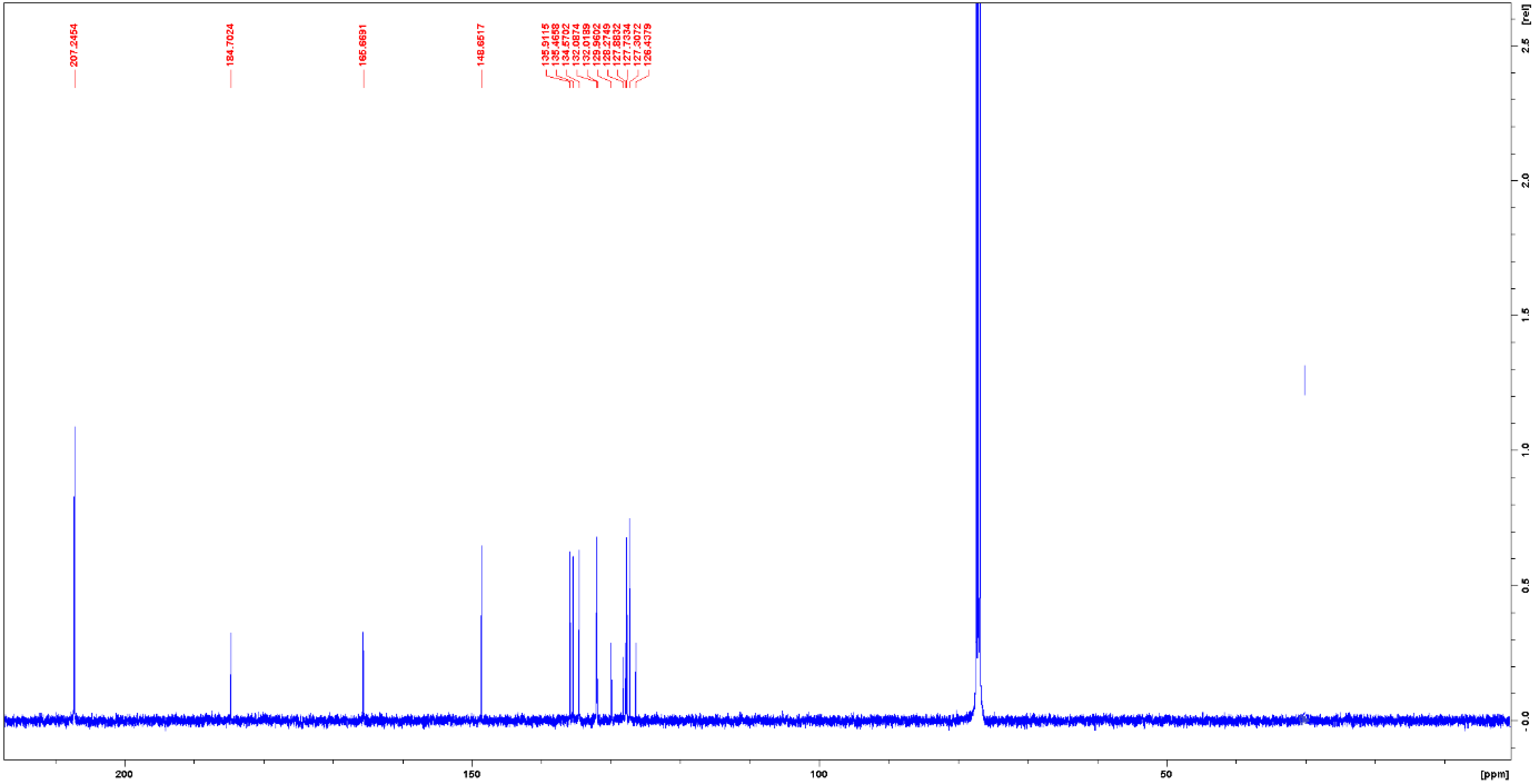

^{1}H NMR spectrum (500 MHz, $CDCl_3$) of compound **21**:

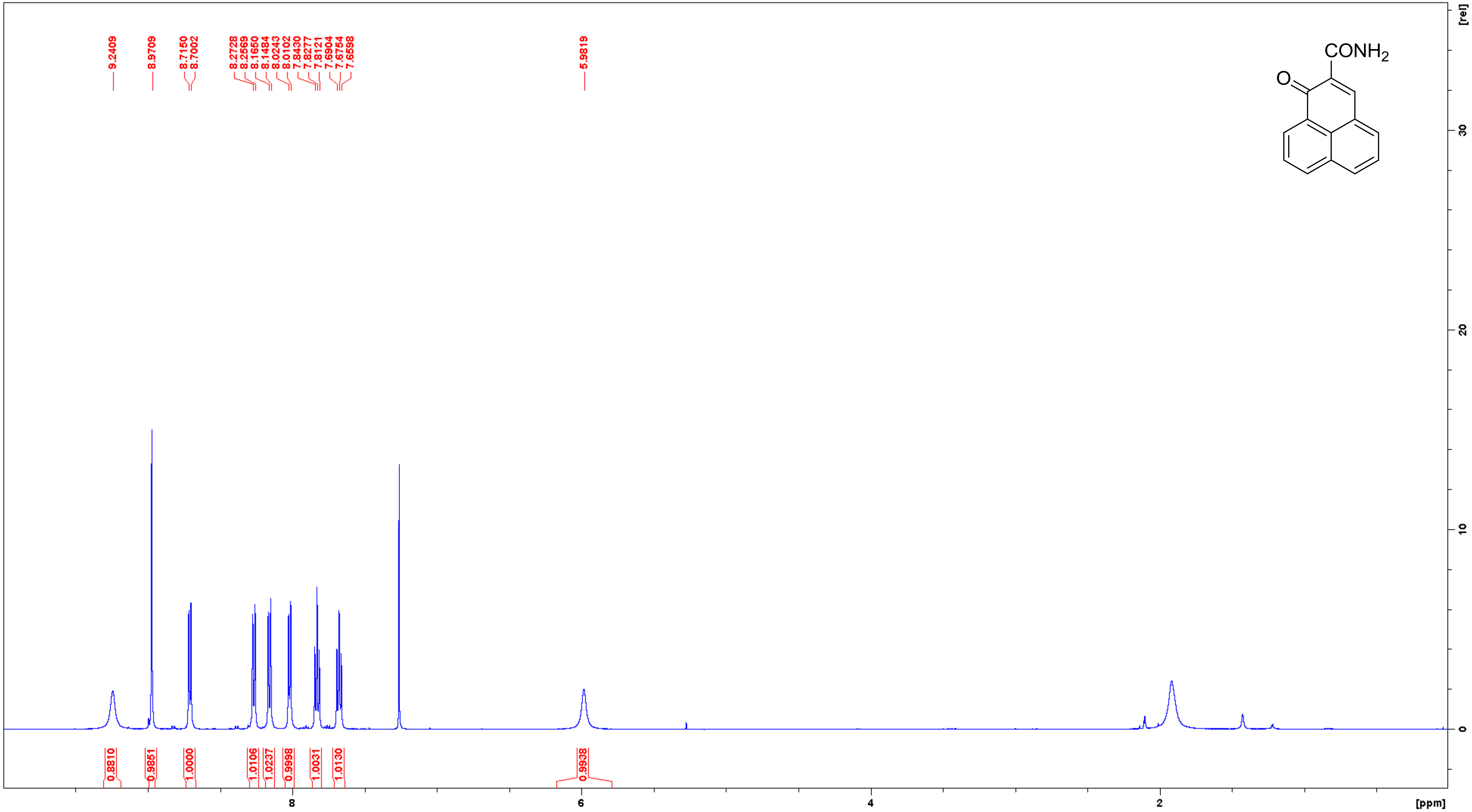

Expanded ^{1}H NMR spectrum (500 MHz, $CDCl_3$) of compound **21**:

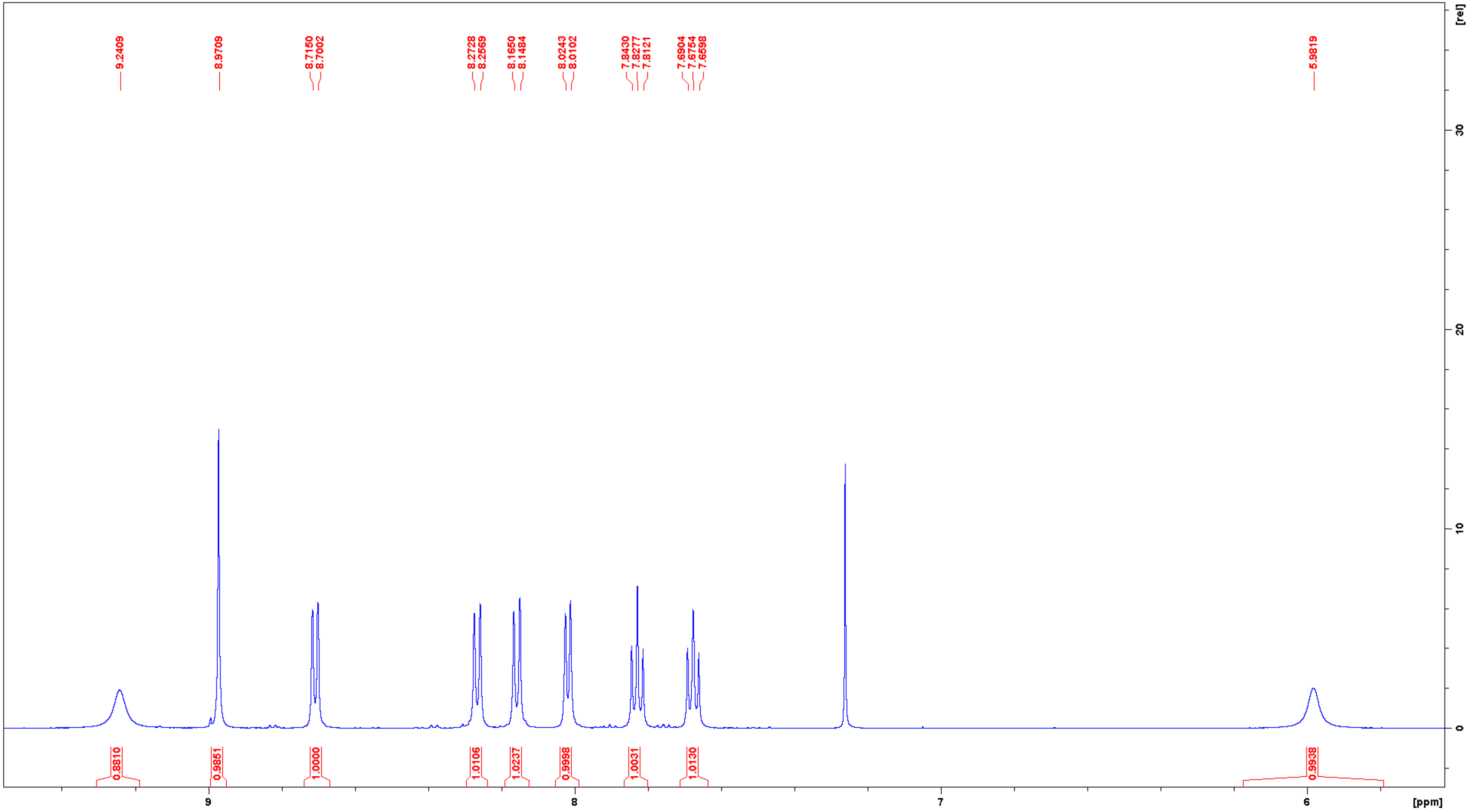

^{13}C NMR spectrum (125 MHz, $CDCl_3$) of compound **21**:

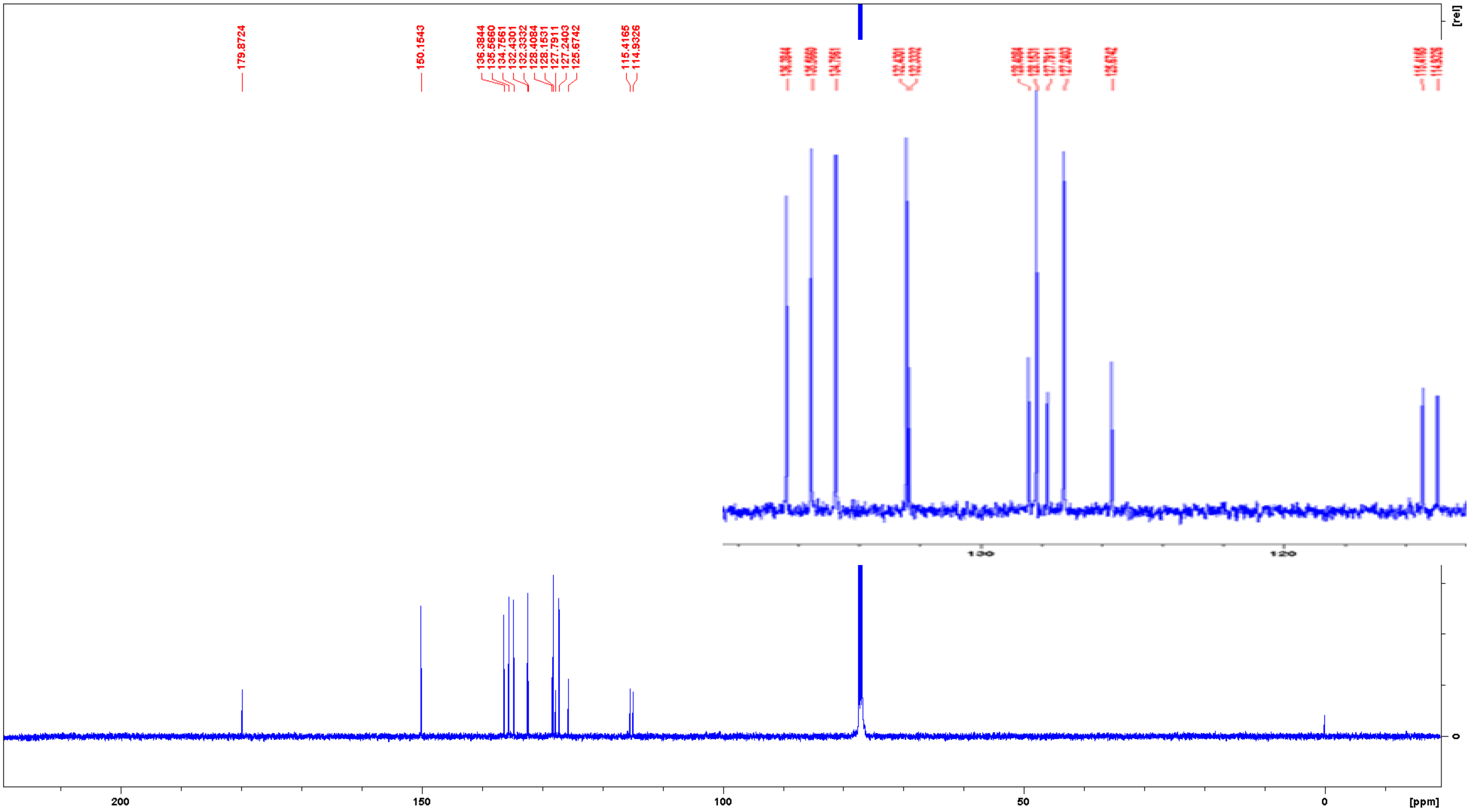

^{1}H NMR spectrum (400 MHz, $CDCl_3$) of compound **22**:

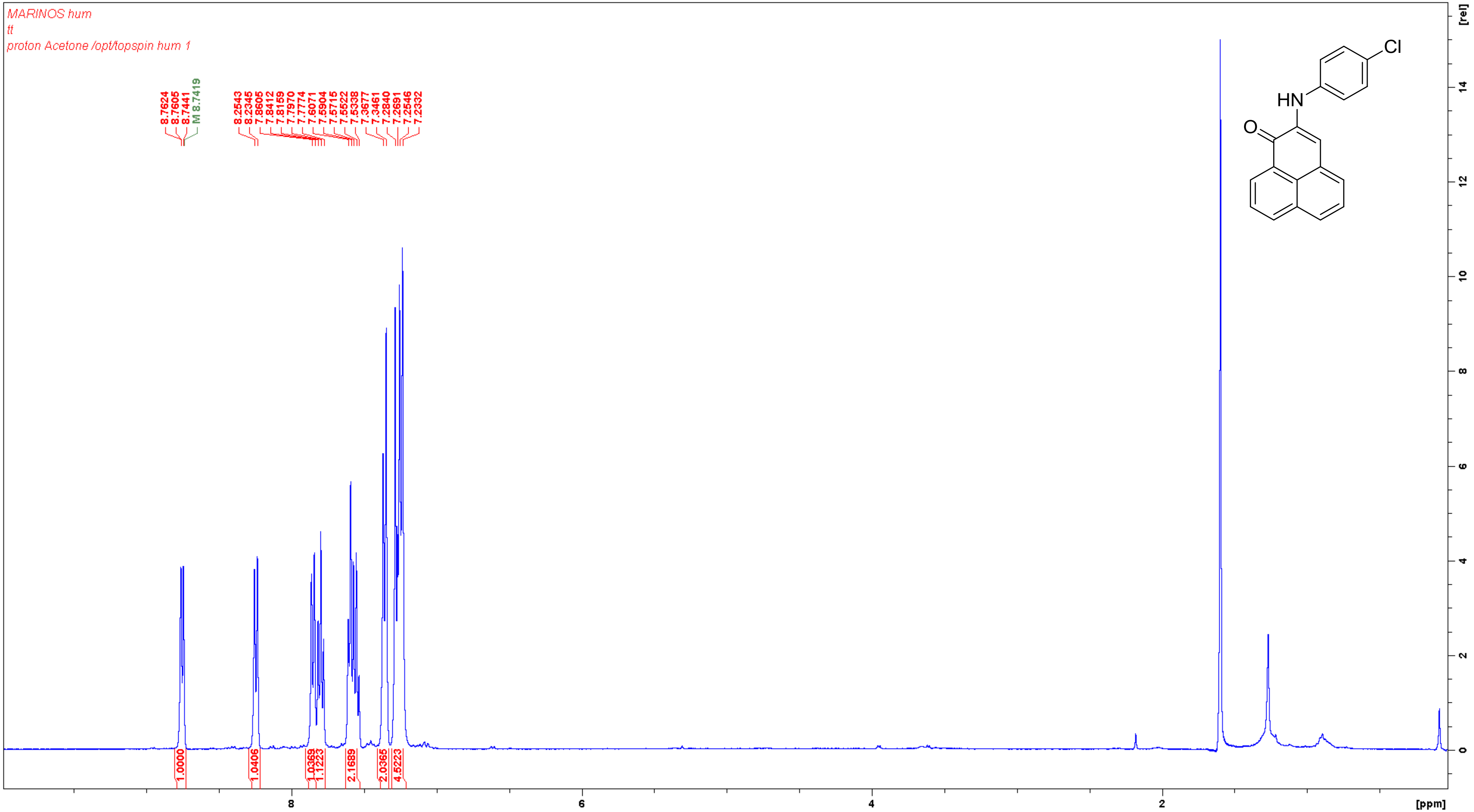

^{13}C NMR spectrum (100 MHz, $CDCl_3$) of compound **22**:

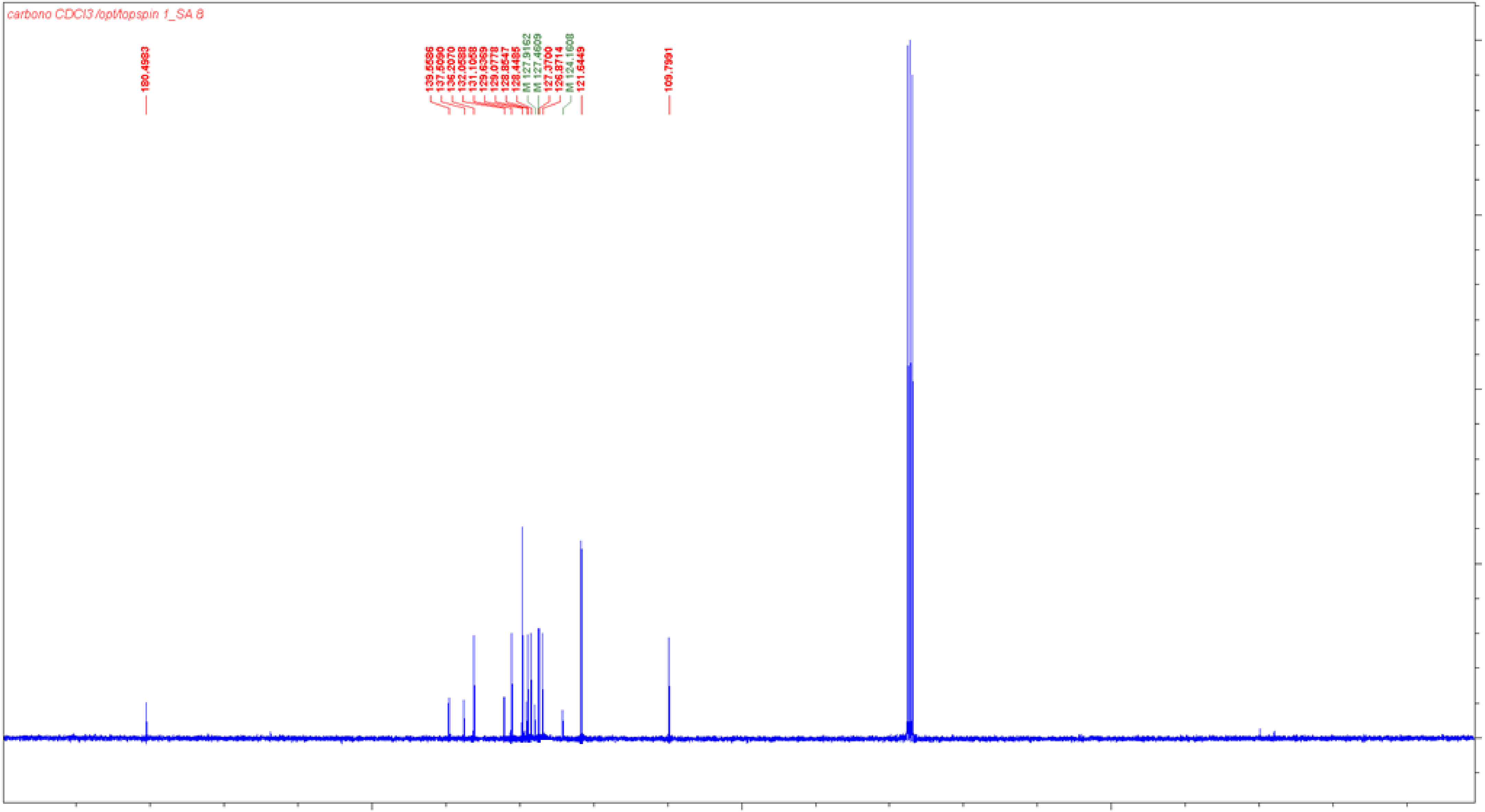

Dept 135 spectrum (100 MHz, $CDCl_3$) of compound **22**:

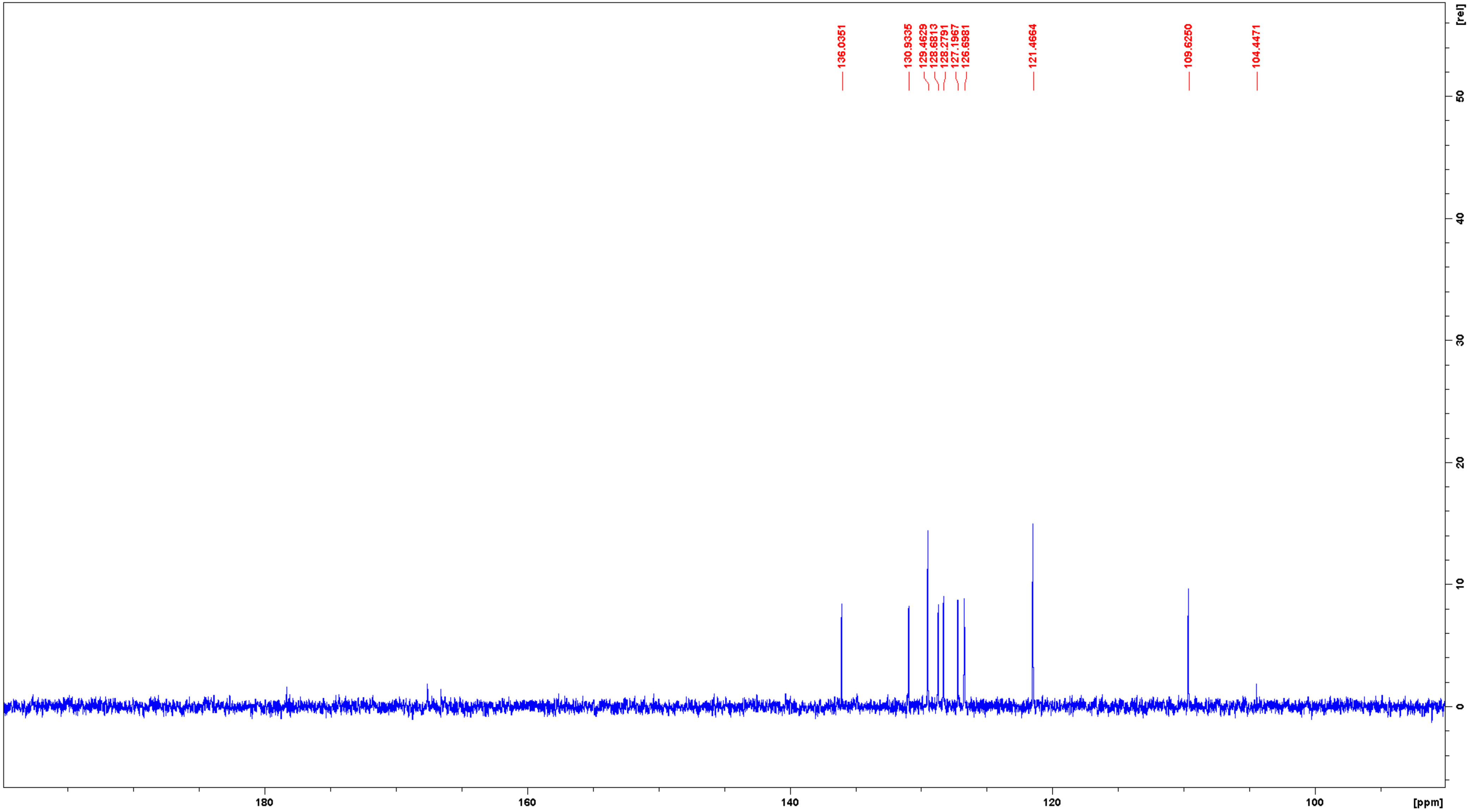

^{1}H NMR spectrum (400 MHz, $CDCl_3$) of compound **23**:

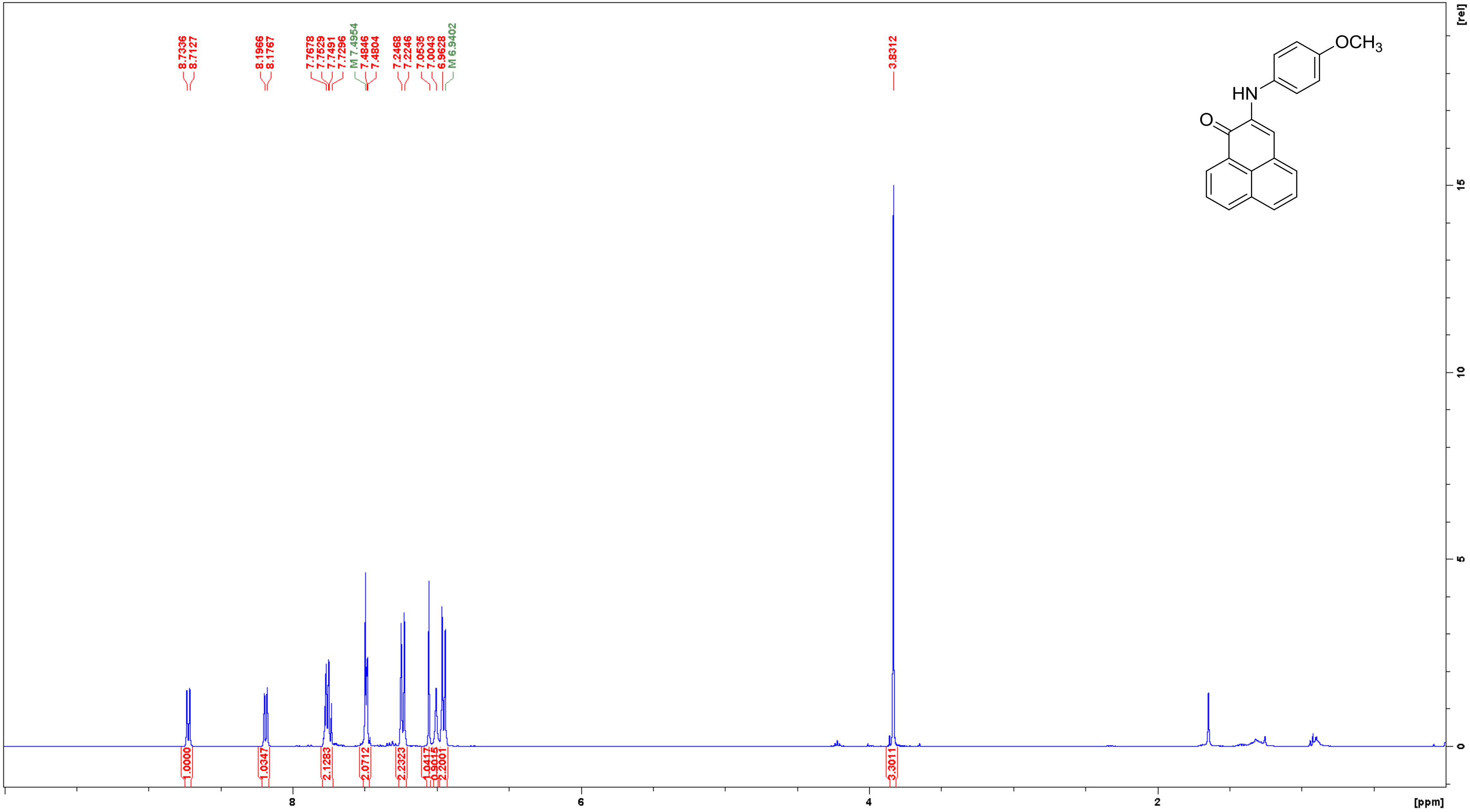

^{13}C NMR spectrum (100 MHz, $CDCl_3$) of compound **23**:

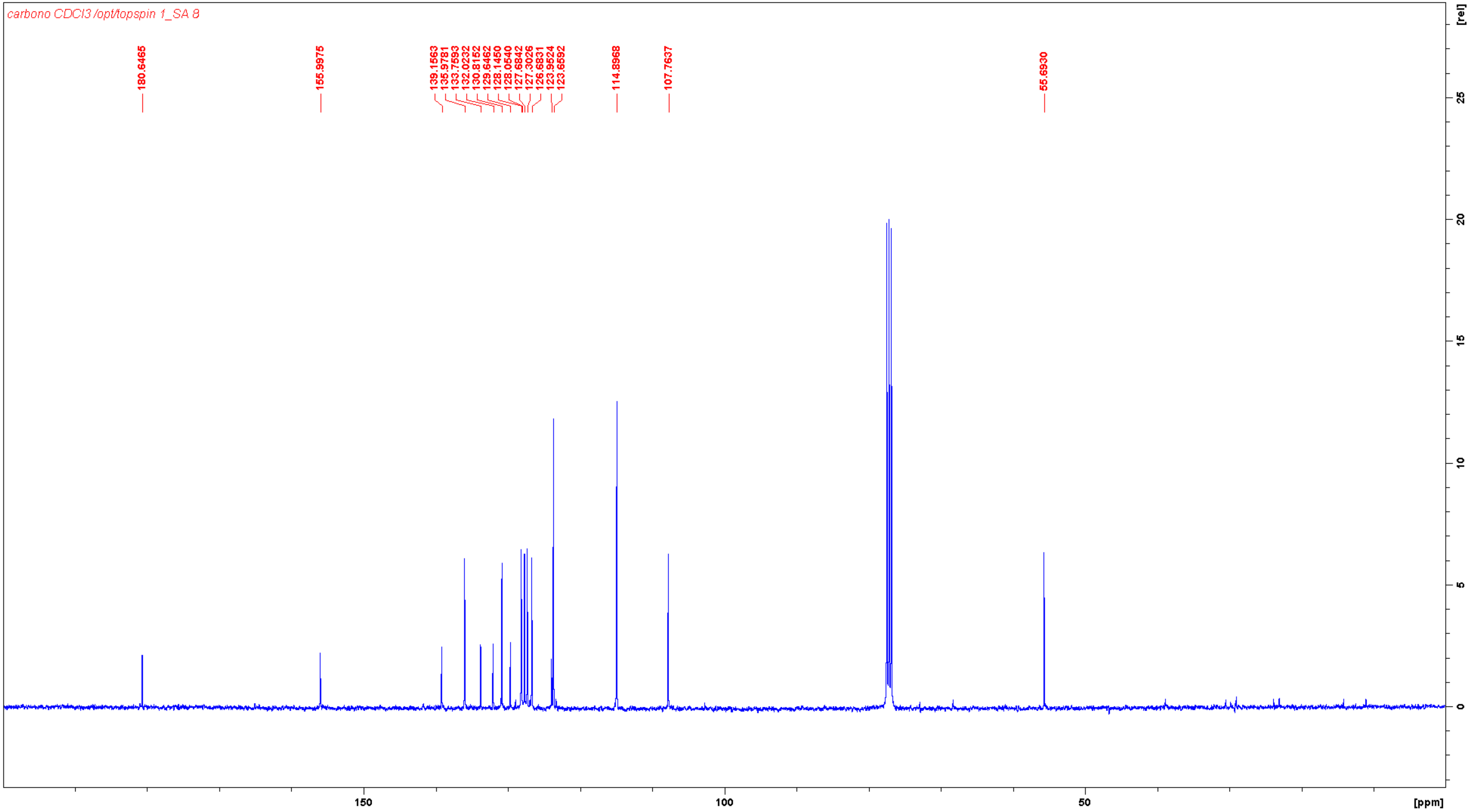

^{1}H NMR spectrum (400 MHz, $CDCl_3$) of compound **24**:

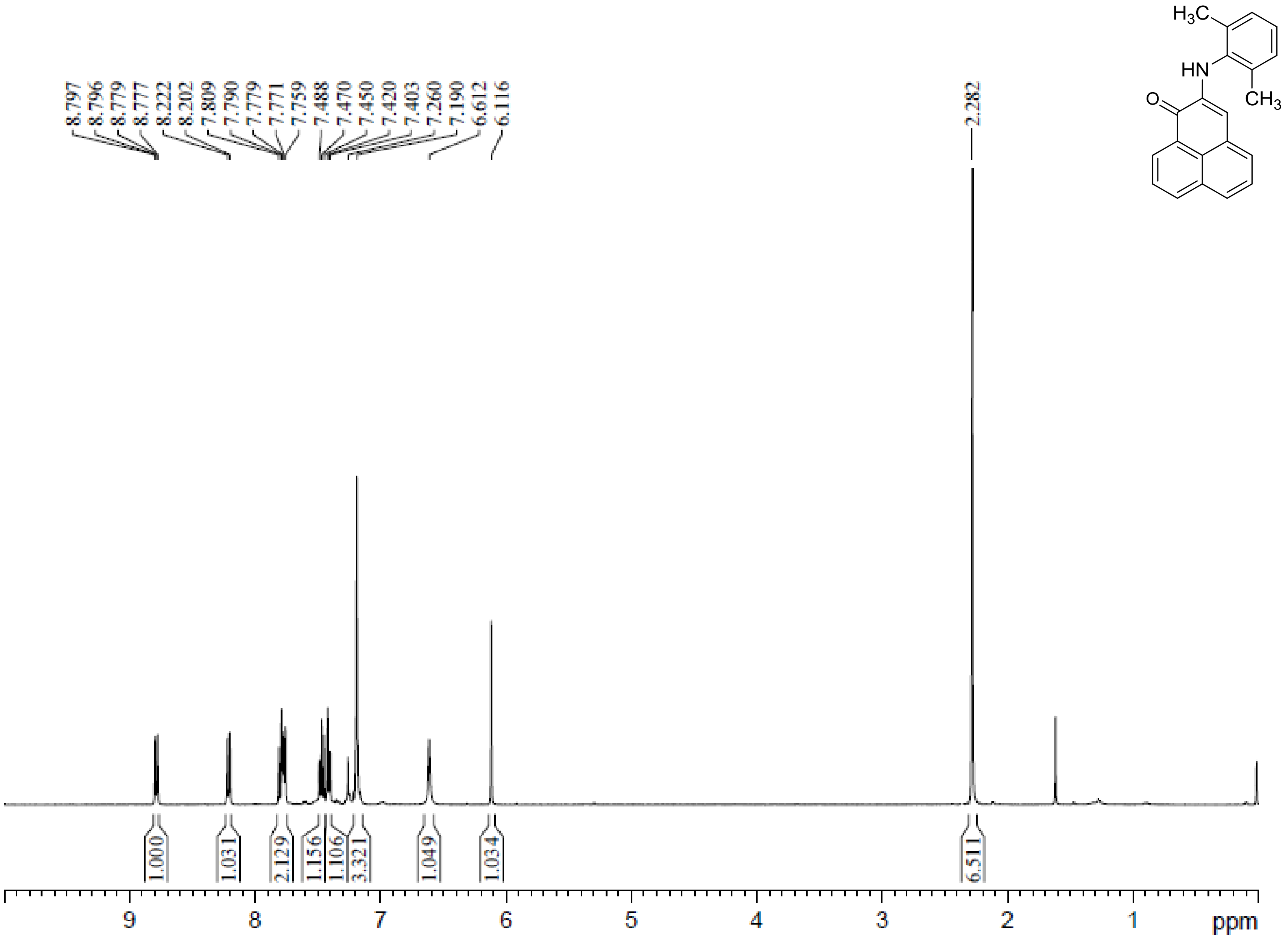

^{13}C NMR spectrum (100 MHz, $CDCl_3$) of compound **24**:

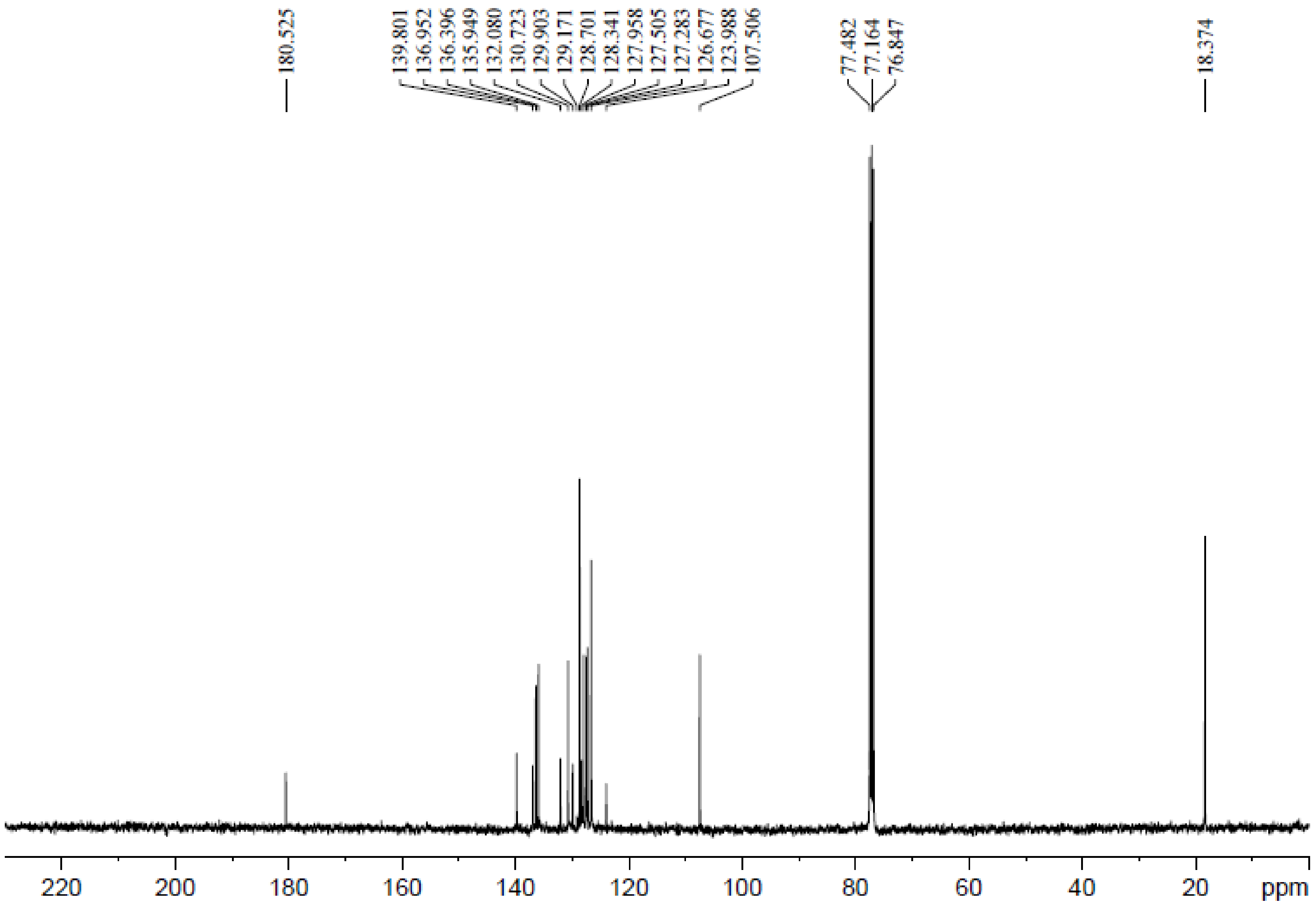

Expanded ^{13}C NMR spectrum (100 MHz, $CDCl_3$) of compound **24**:

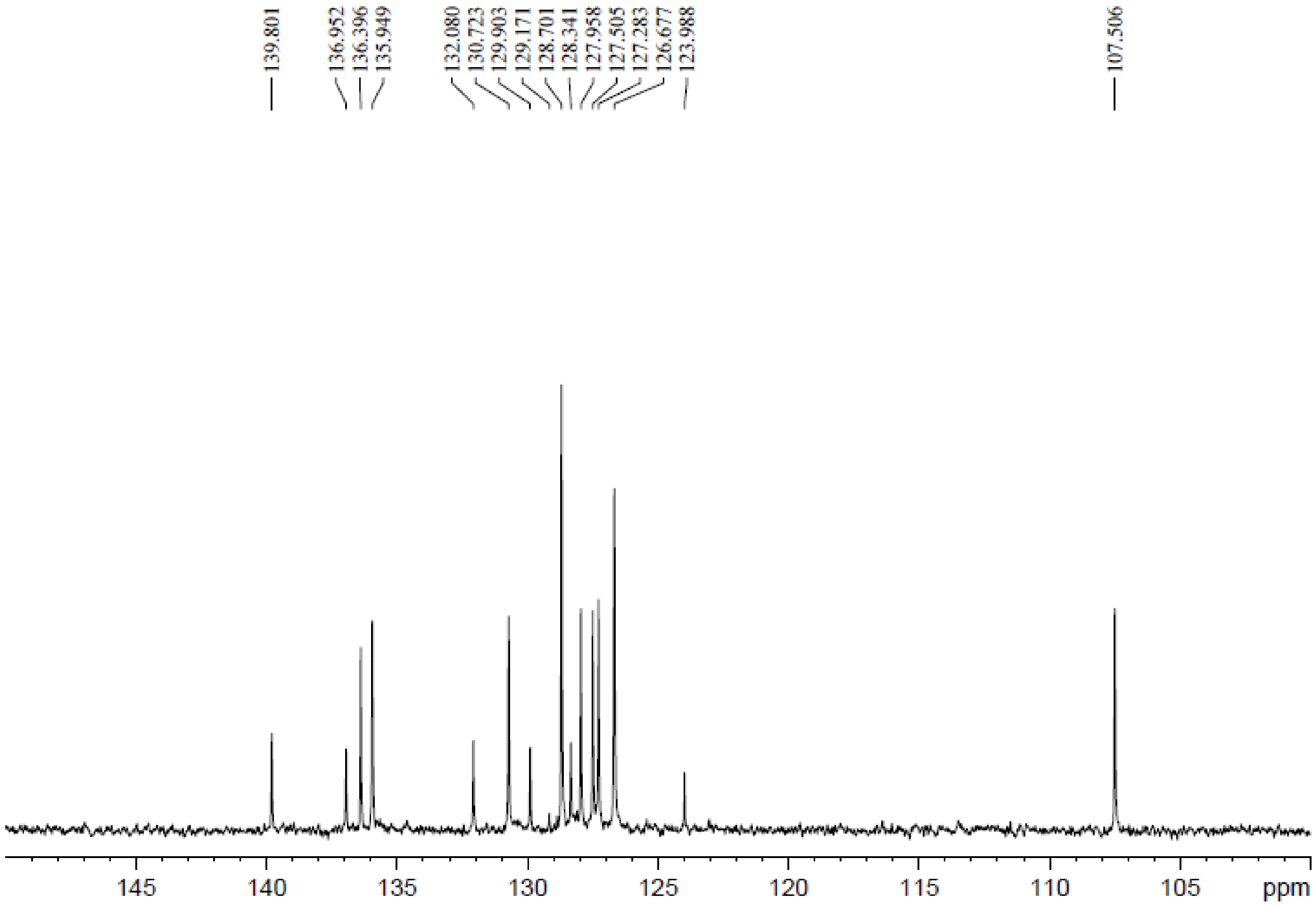

^{1}H NMR spectrum (400 MHz, $CDCl_3$) of compound **25**:

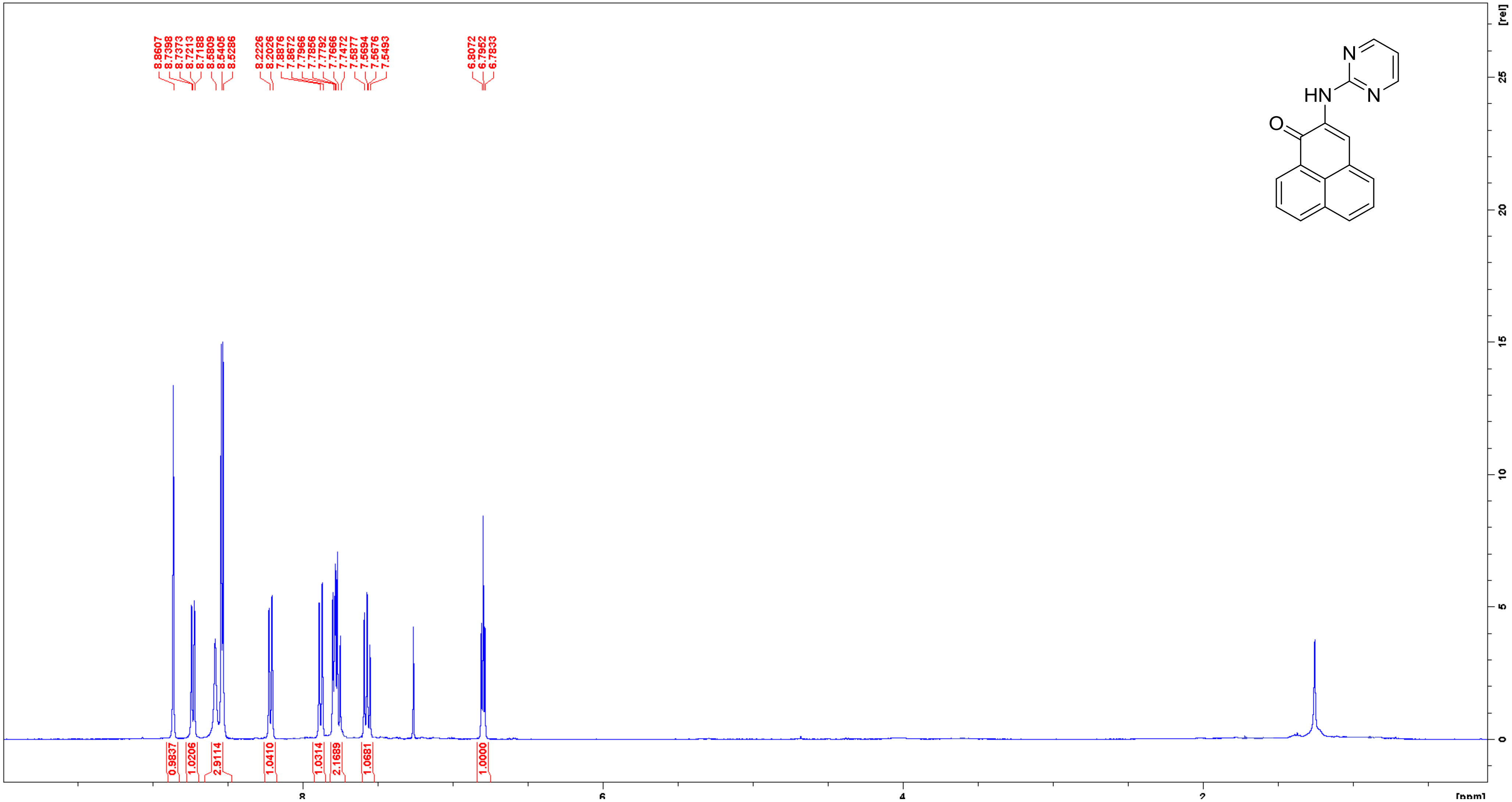

^{13}C NMR spectrum (100 MHz, $CDCl_3$) of compound **25**:

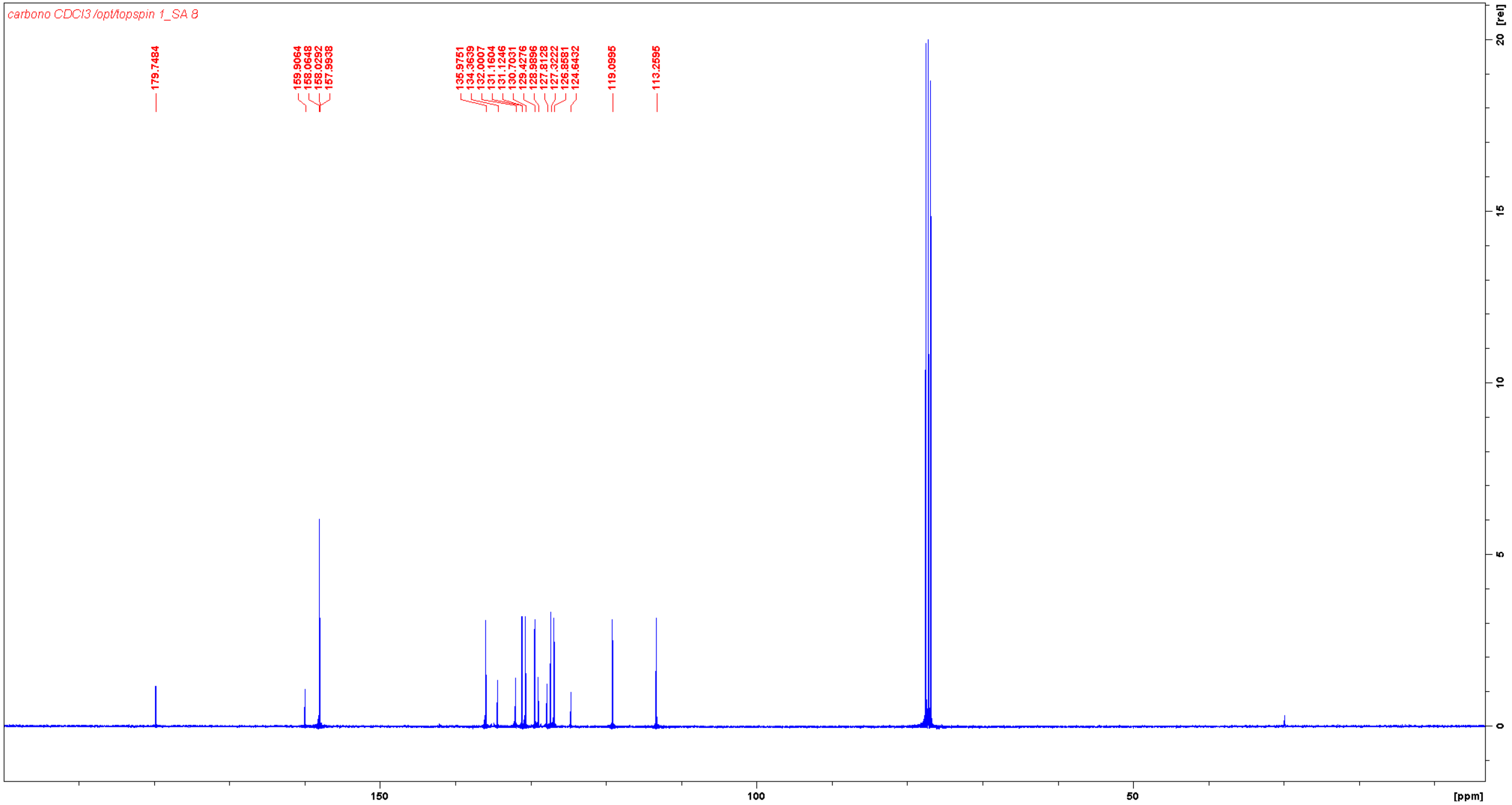

^{1}H NMR spectrum (500 MHz, $(CD_3)_2CO$) of 9-hydroxy-phenalen-1-one:

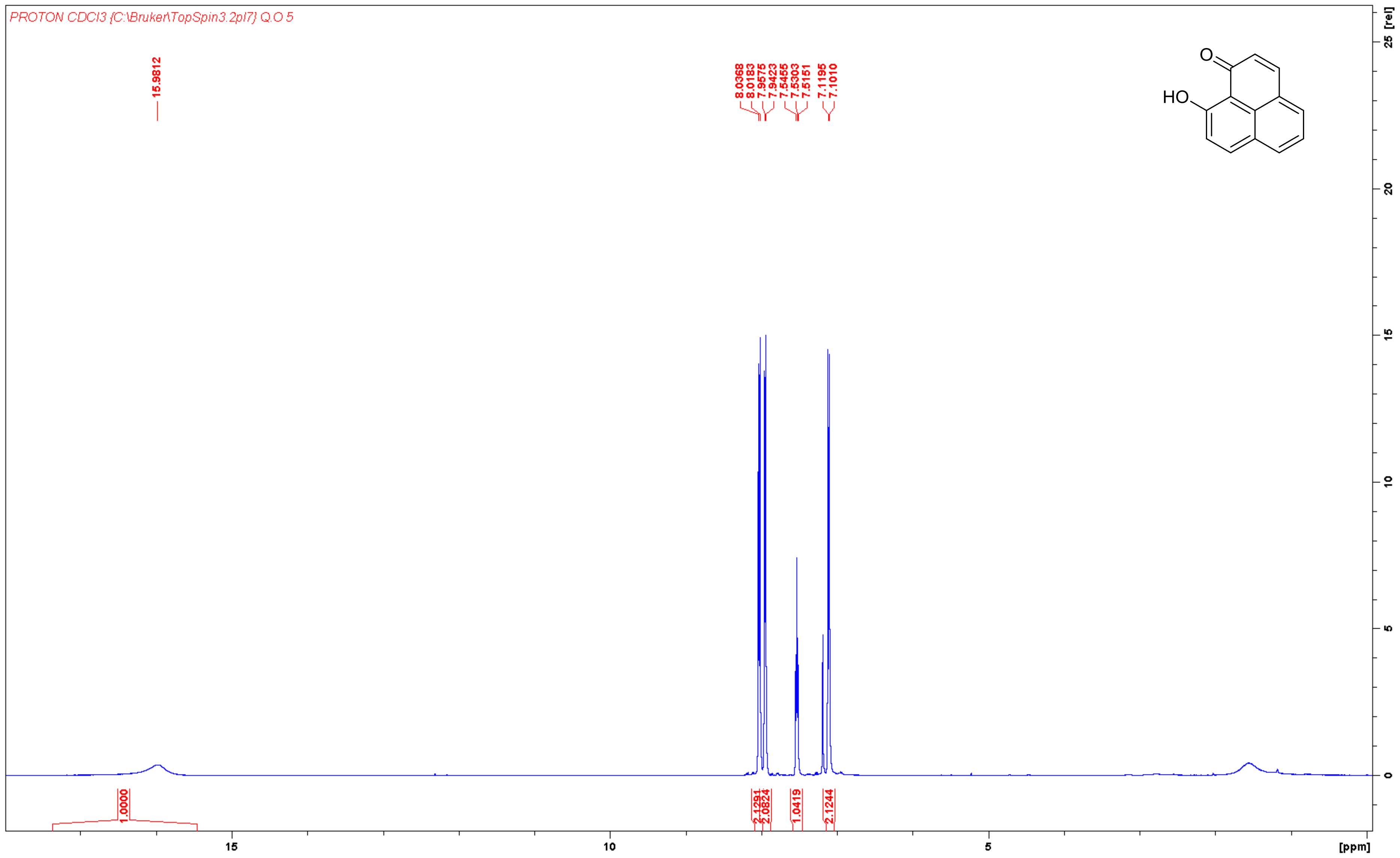

^{13}C NMR spectrum (125 MHz, $CDCl_3$) of 9-hydroxy-phenalen-1-one:

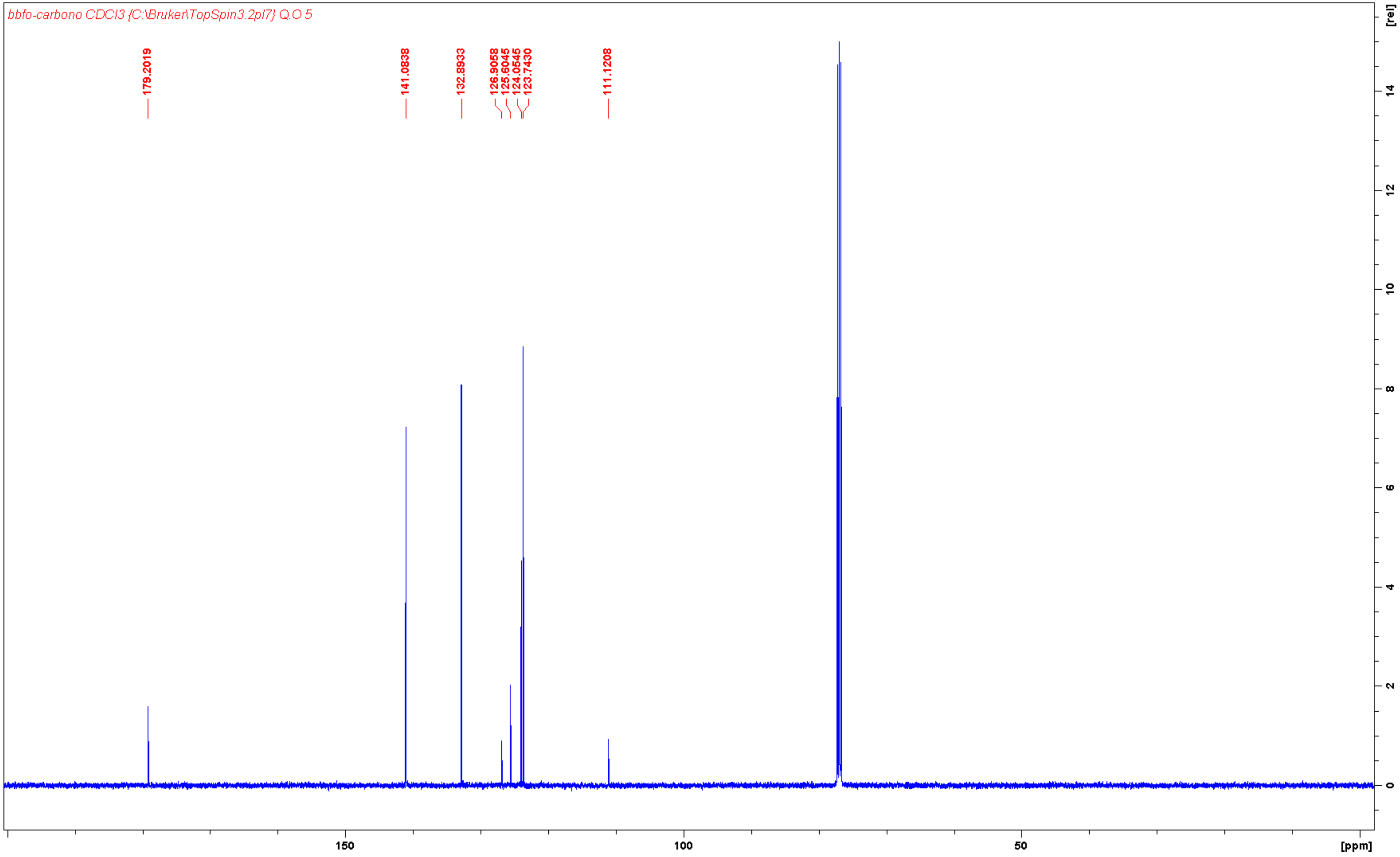

^{1}H NMR spectrum (400 MHz, $(CD_3)_2CO$) of compound **30**:

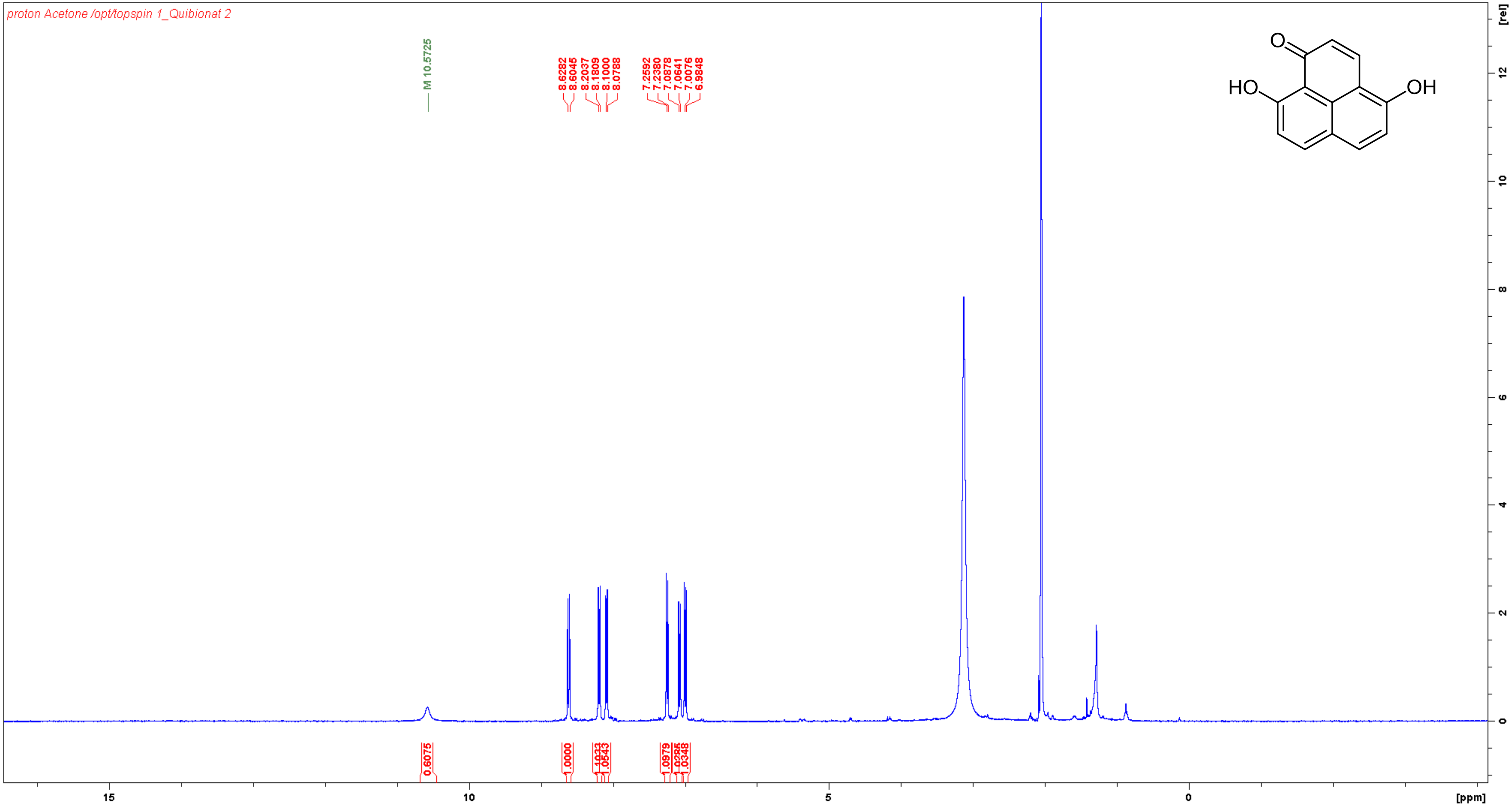

^{1}H NMR spectrum (500 MHz, $CDCl_3$) of compound **33**:

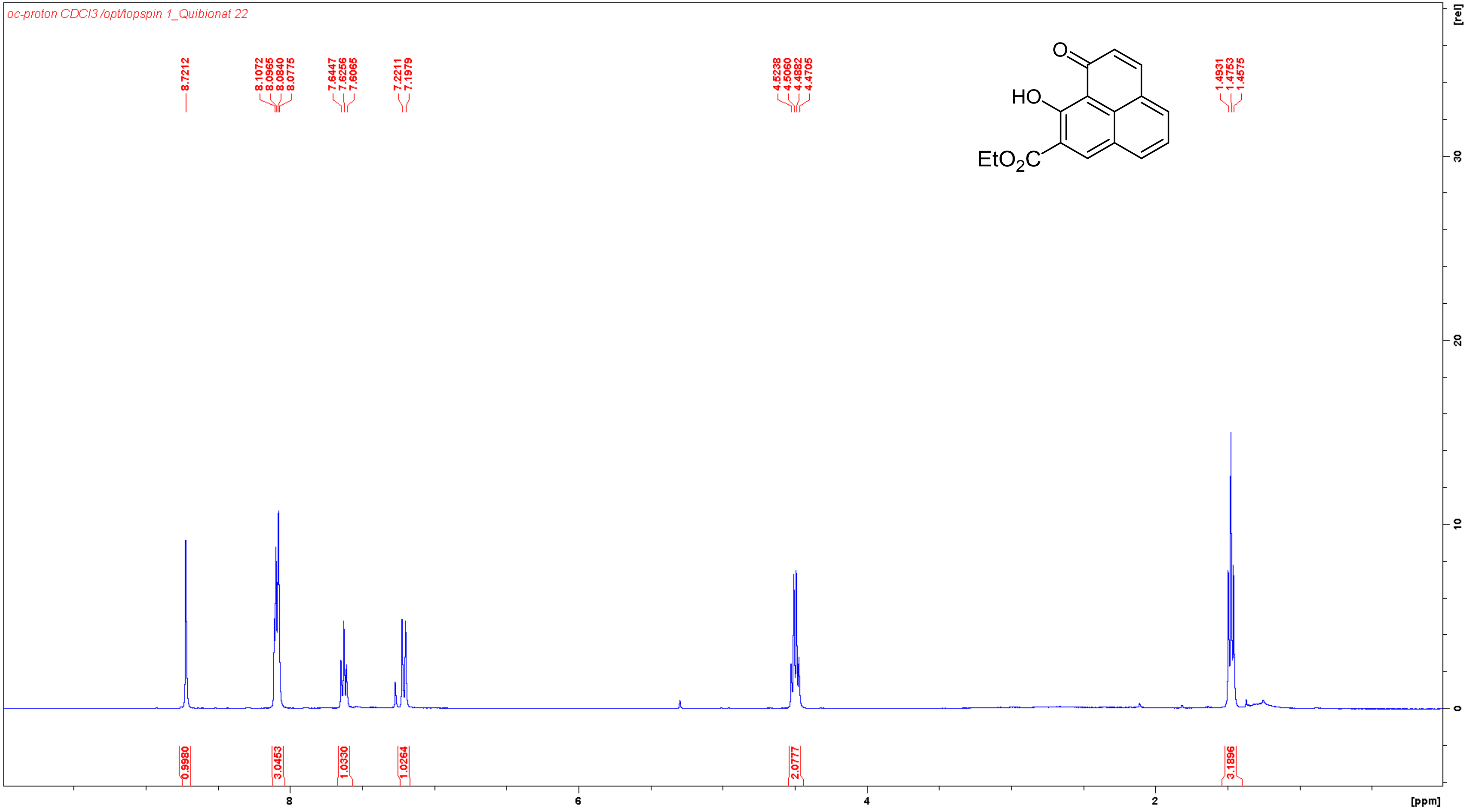

^{13}C NMR spectrum (125 MHz, $CDCl_3$) of compound **33**:

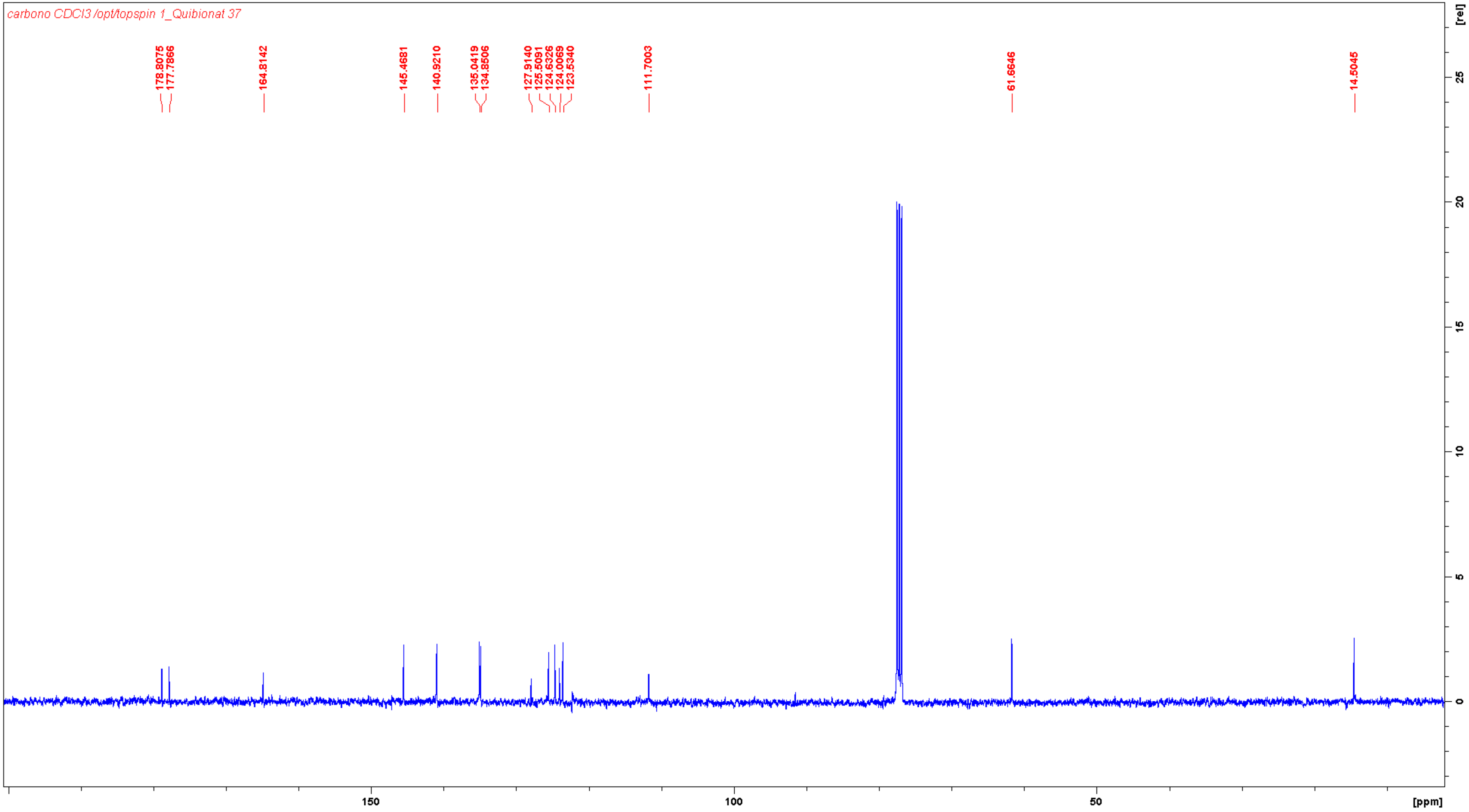

1H NMR spectrum (500 MHz, $CDCl_3$) of compound **34**:

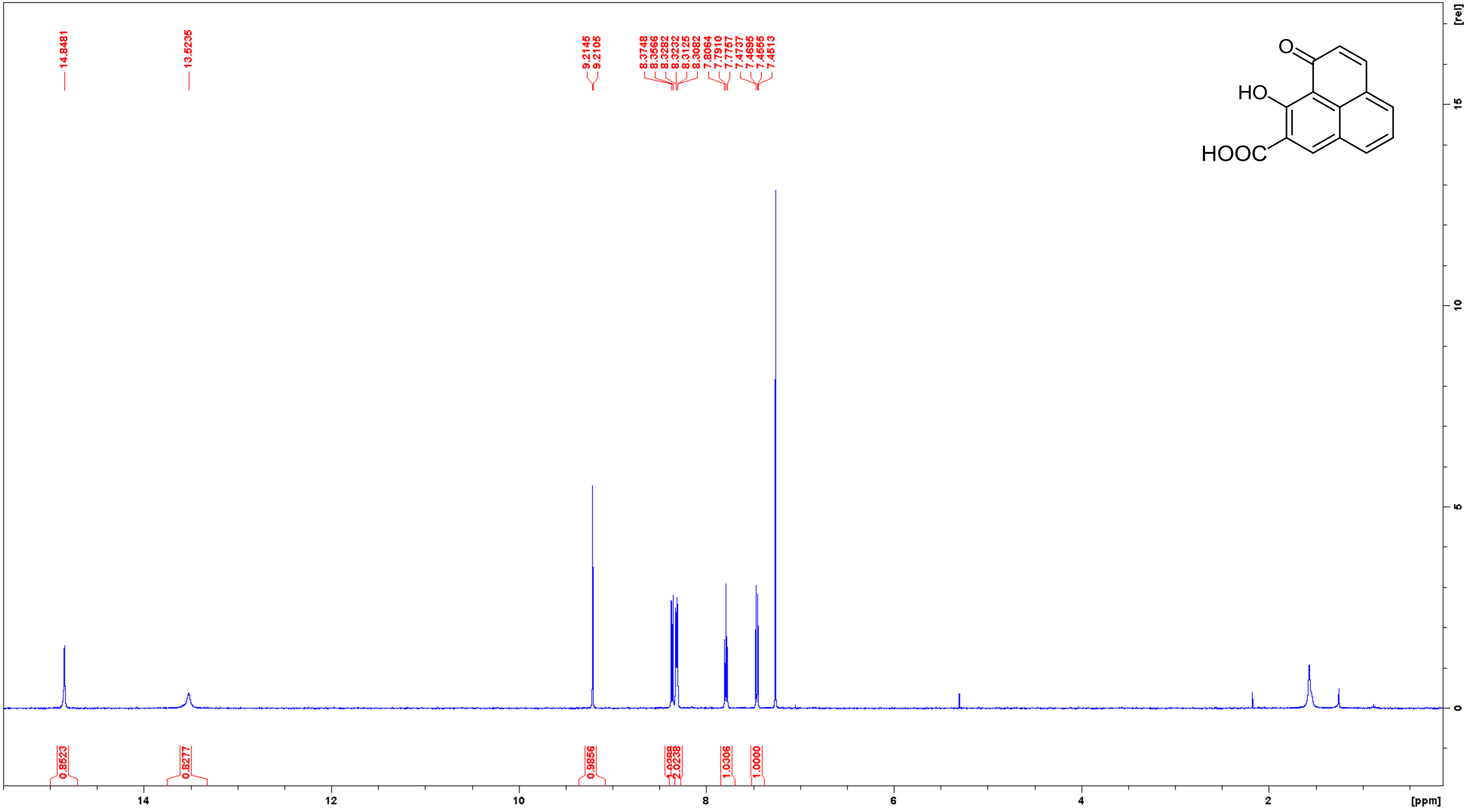

^{13}C NMR spectrum (125 MHz, $CDCl_3$) of compound **34**:

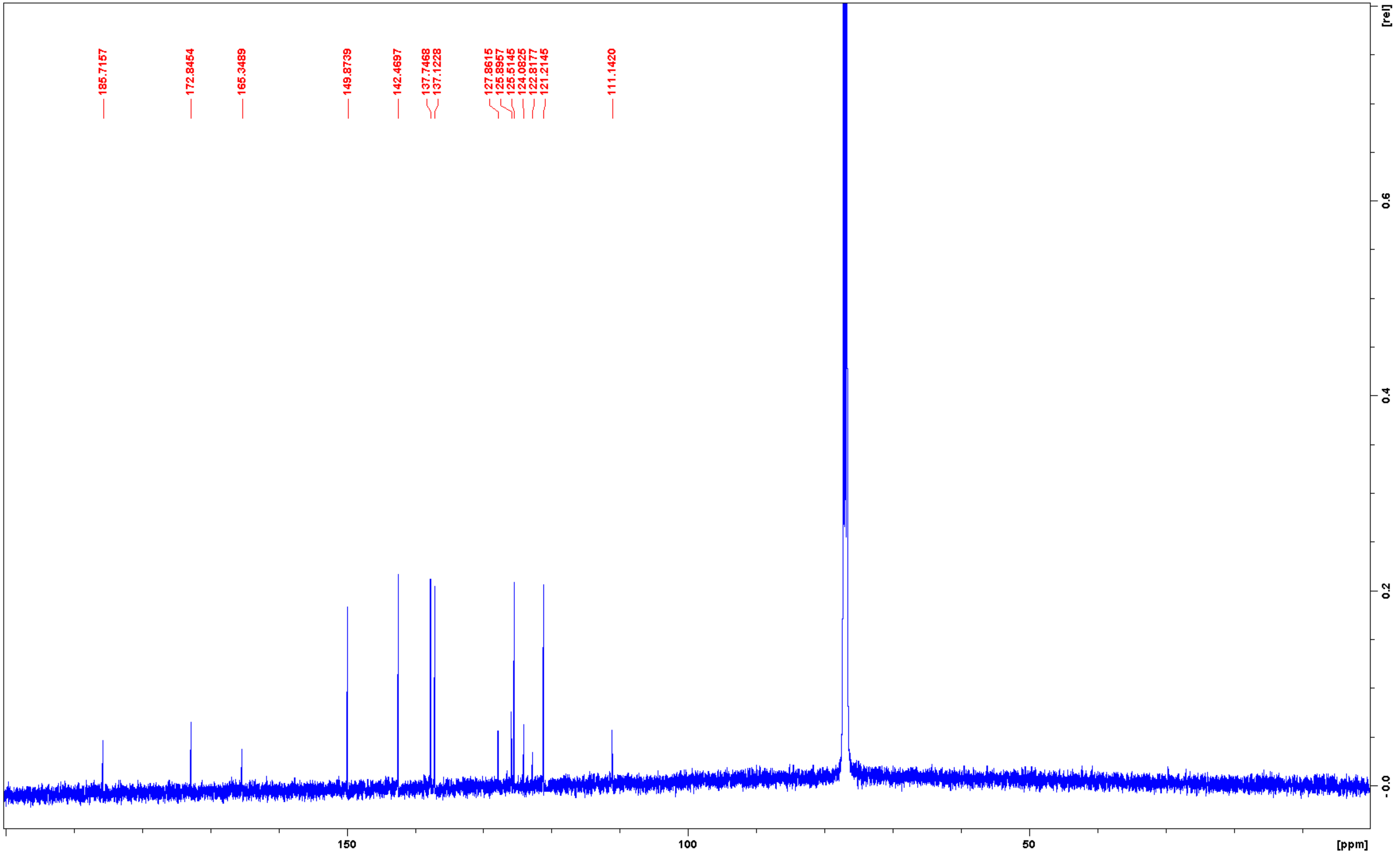

Expanded ^{13}C NMR spectrum (125 MHz, $CDCl_3$) of compound **34**:

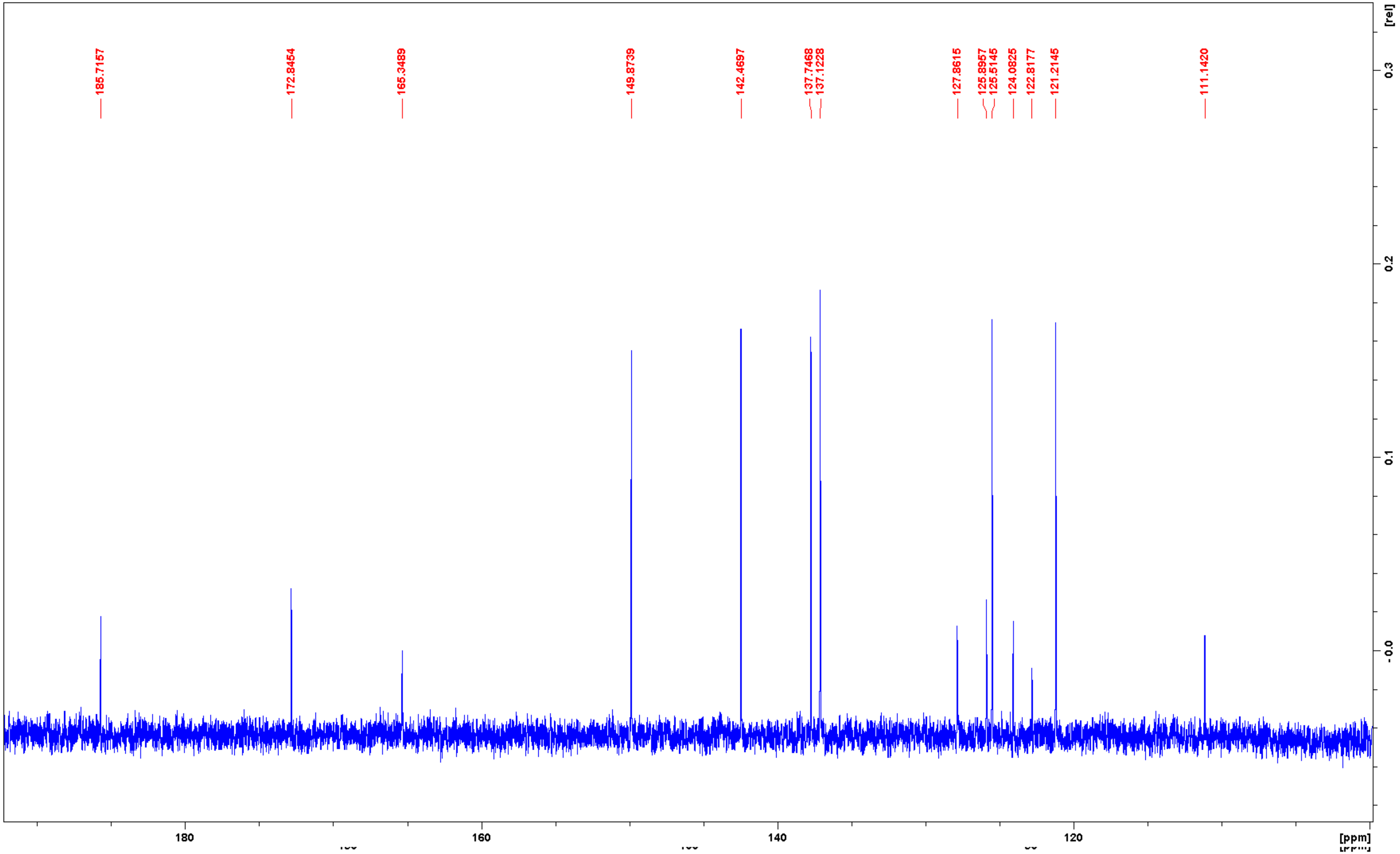

^{13}C NMR spectrum with **DEPT 135 and DEPT 90** (125 MHz, $CDCl_3$) of compound **34**:

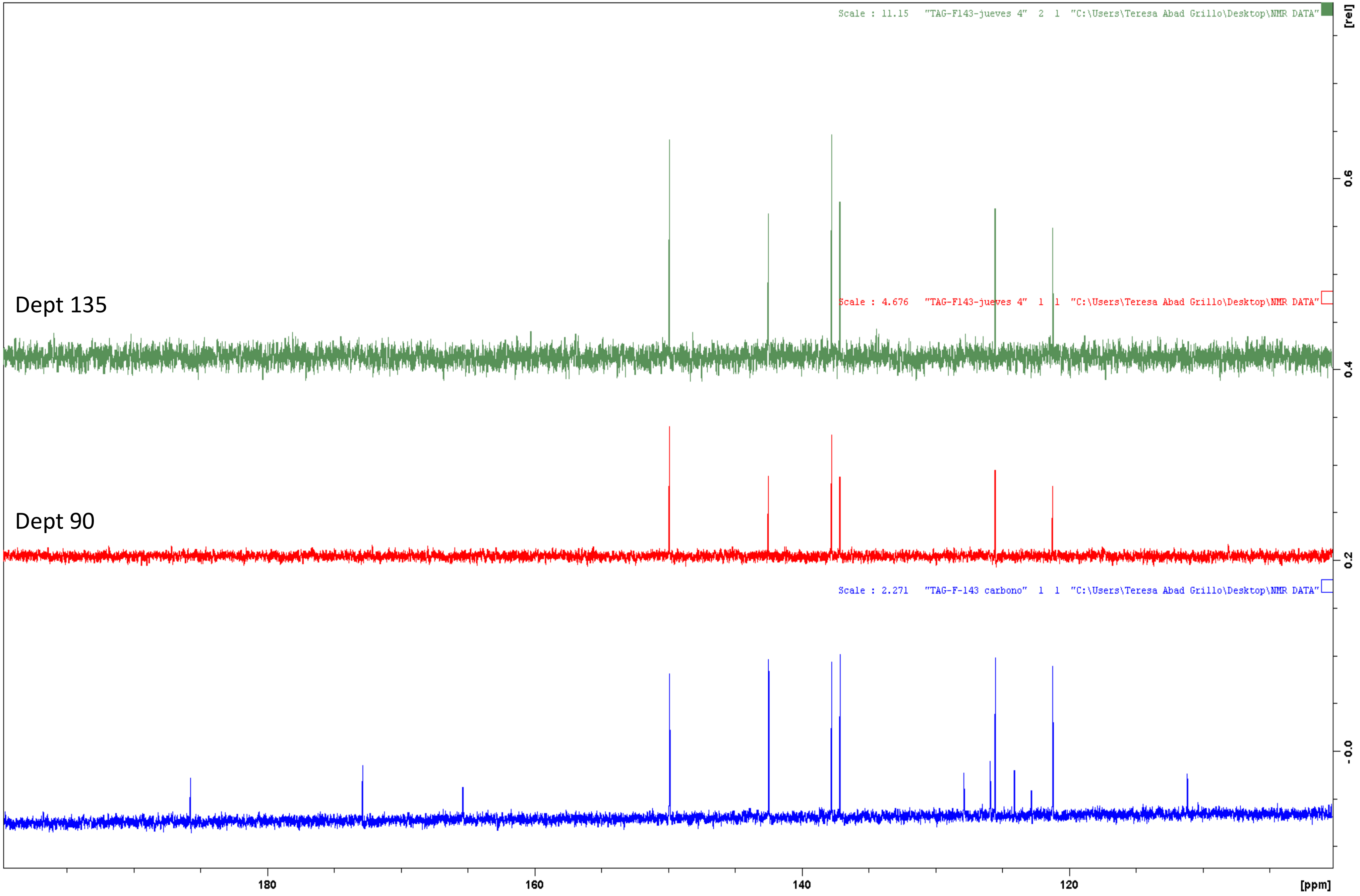

^1H NMR spectrum (600 MHz, $CDCl_3$) of compound **35**:

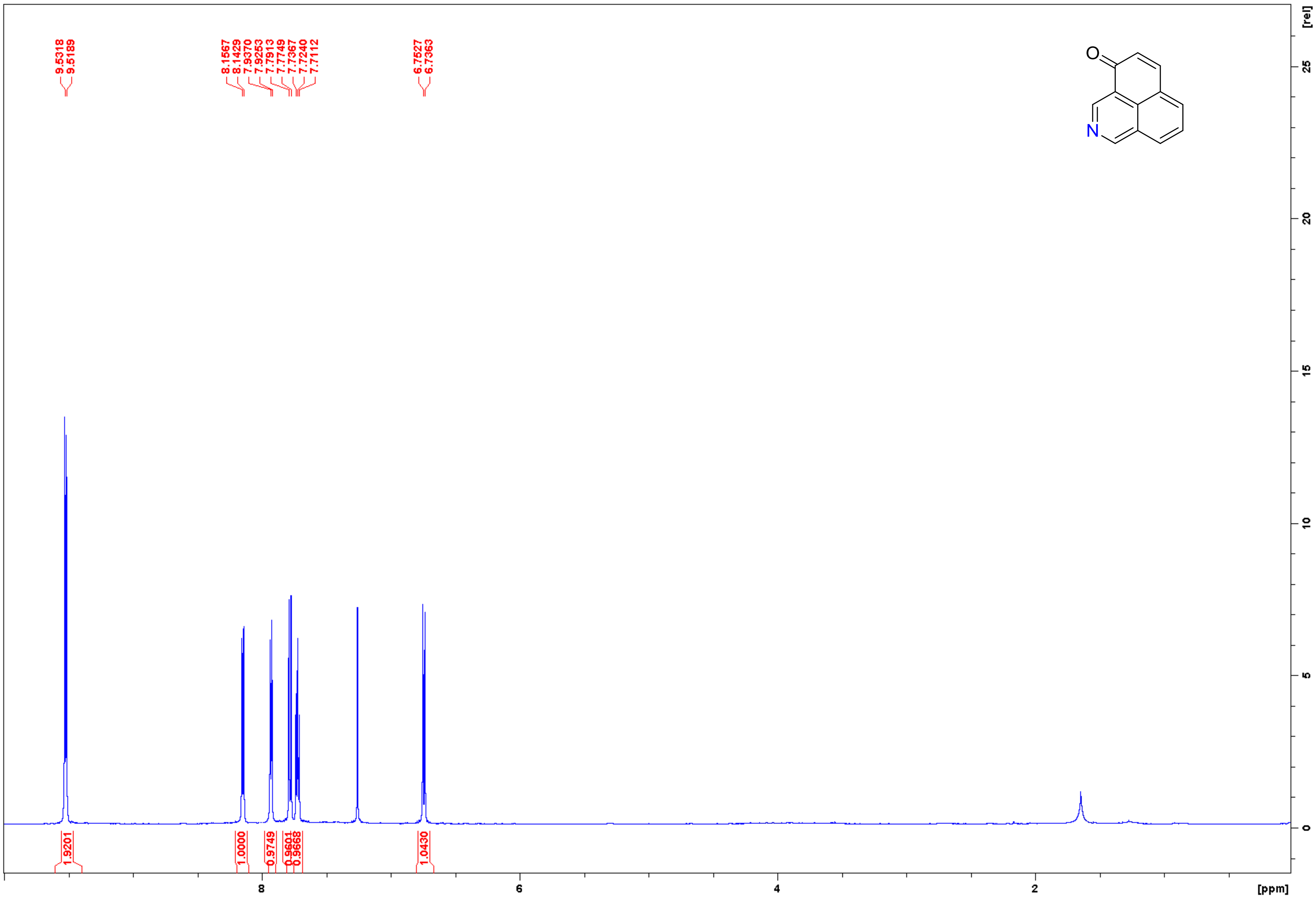

^{13}C NMR spectrum (150 MHz, $CDCl_3$) of compound **35**:

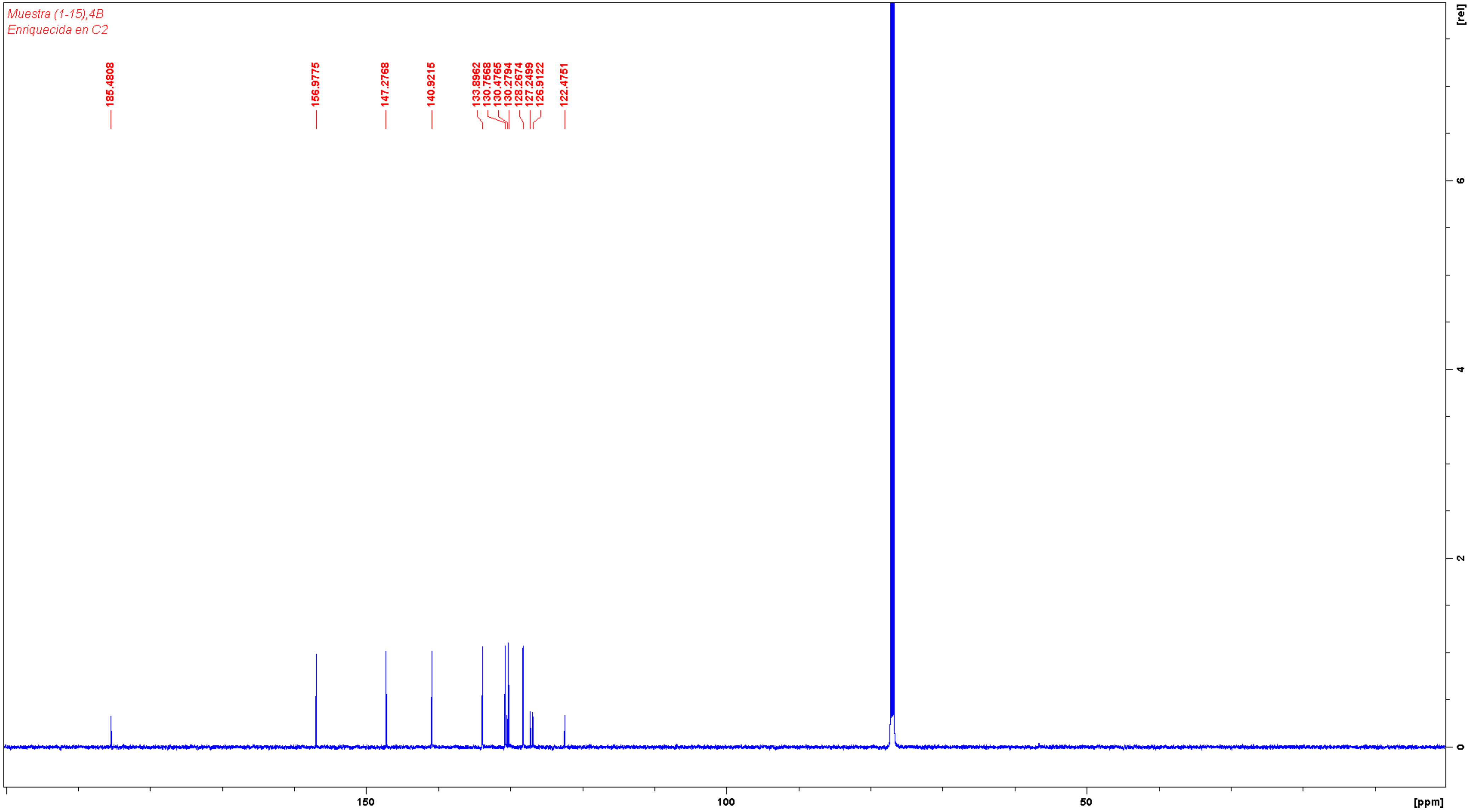

^{1}H NMR spectrum (400 MHz, $CDCl_3$) of 2-methoxynaphthalene-1,6-dicarbaldehyde:

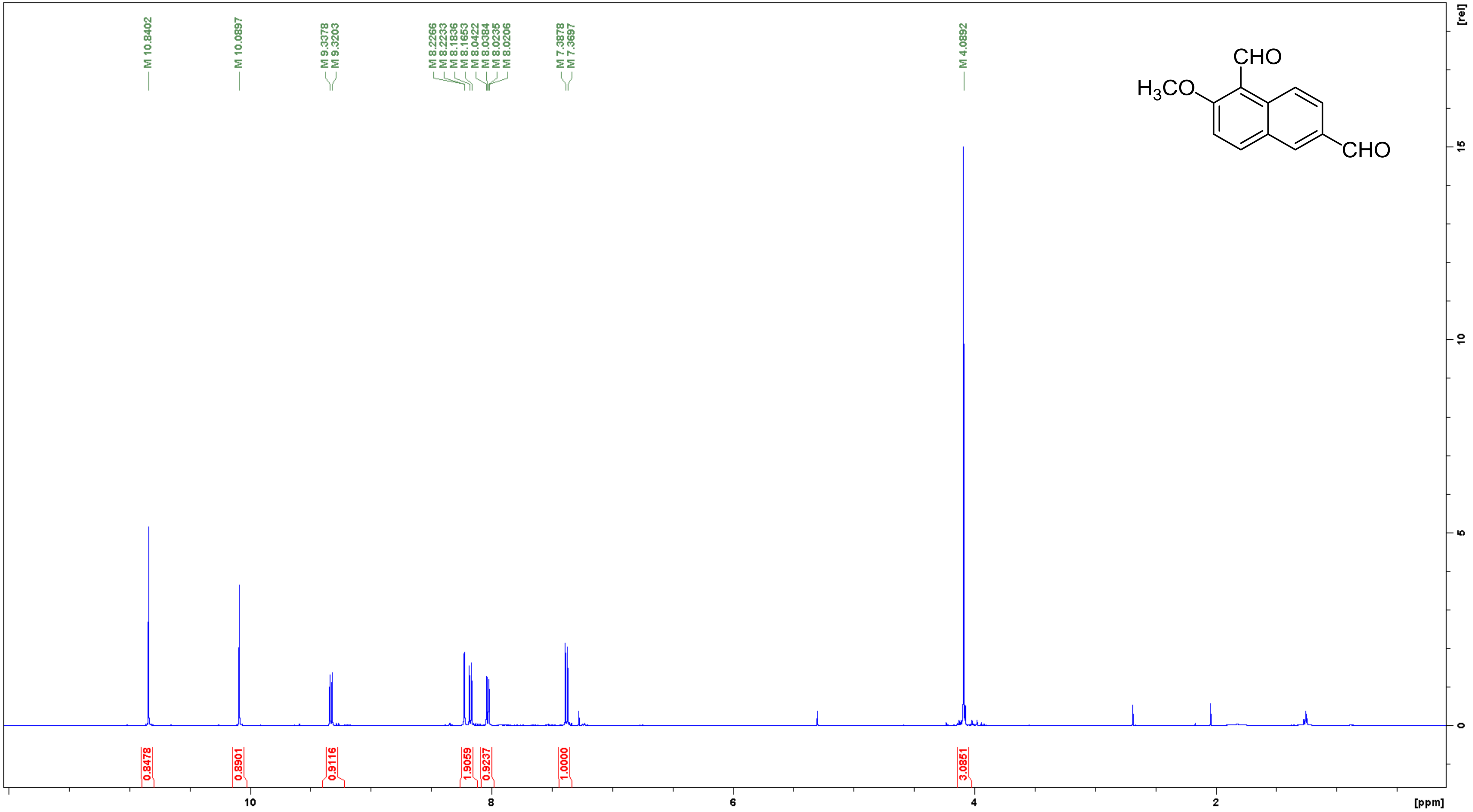

^{1}H NMR spectrum (500 MHz, $CDCl_3$) of compound **36**:

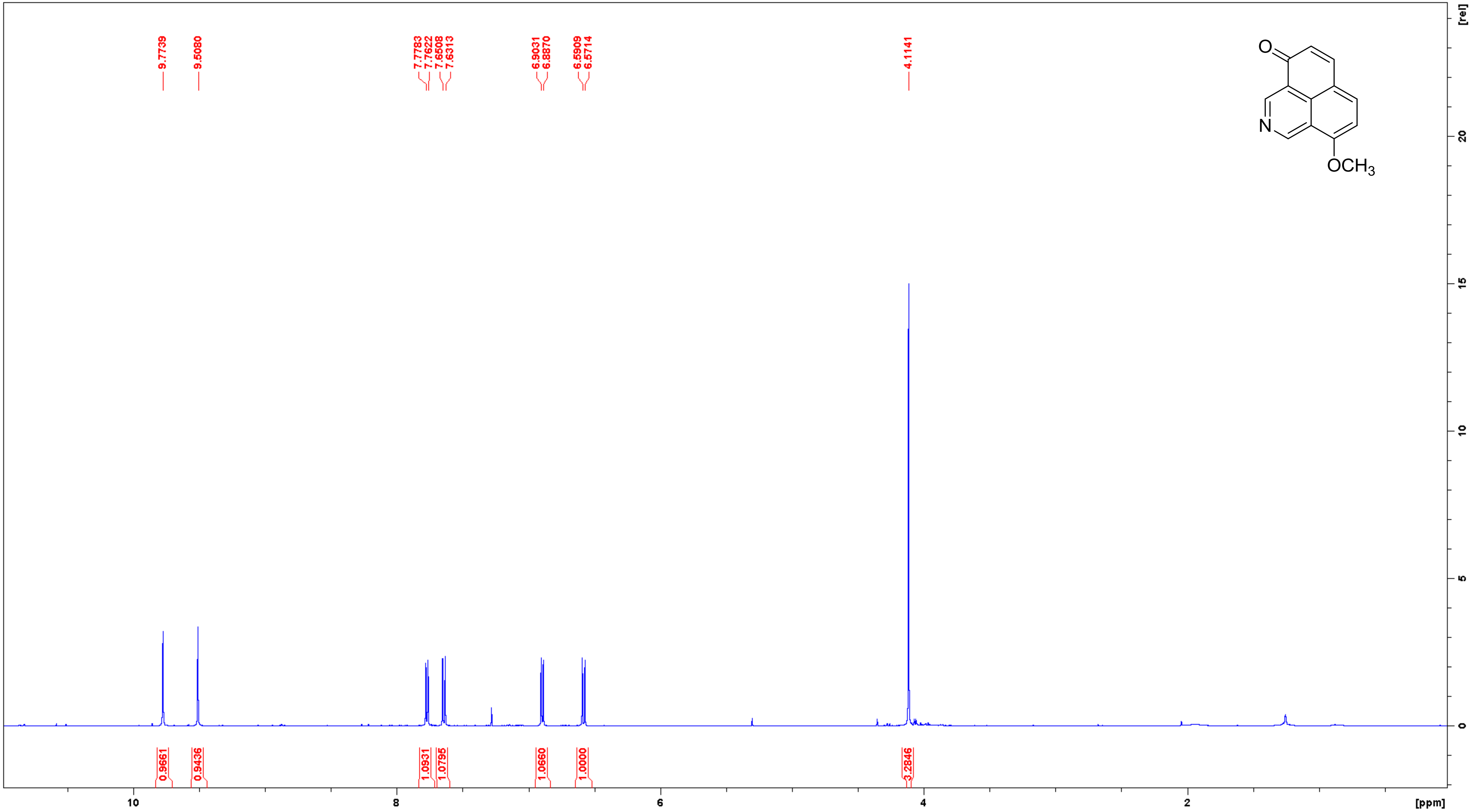

^{13}C NMR spectrum (125 MHz, $CDCl_3$) of compound **36**:

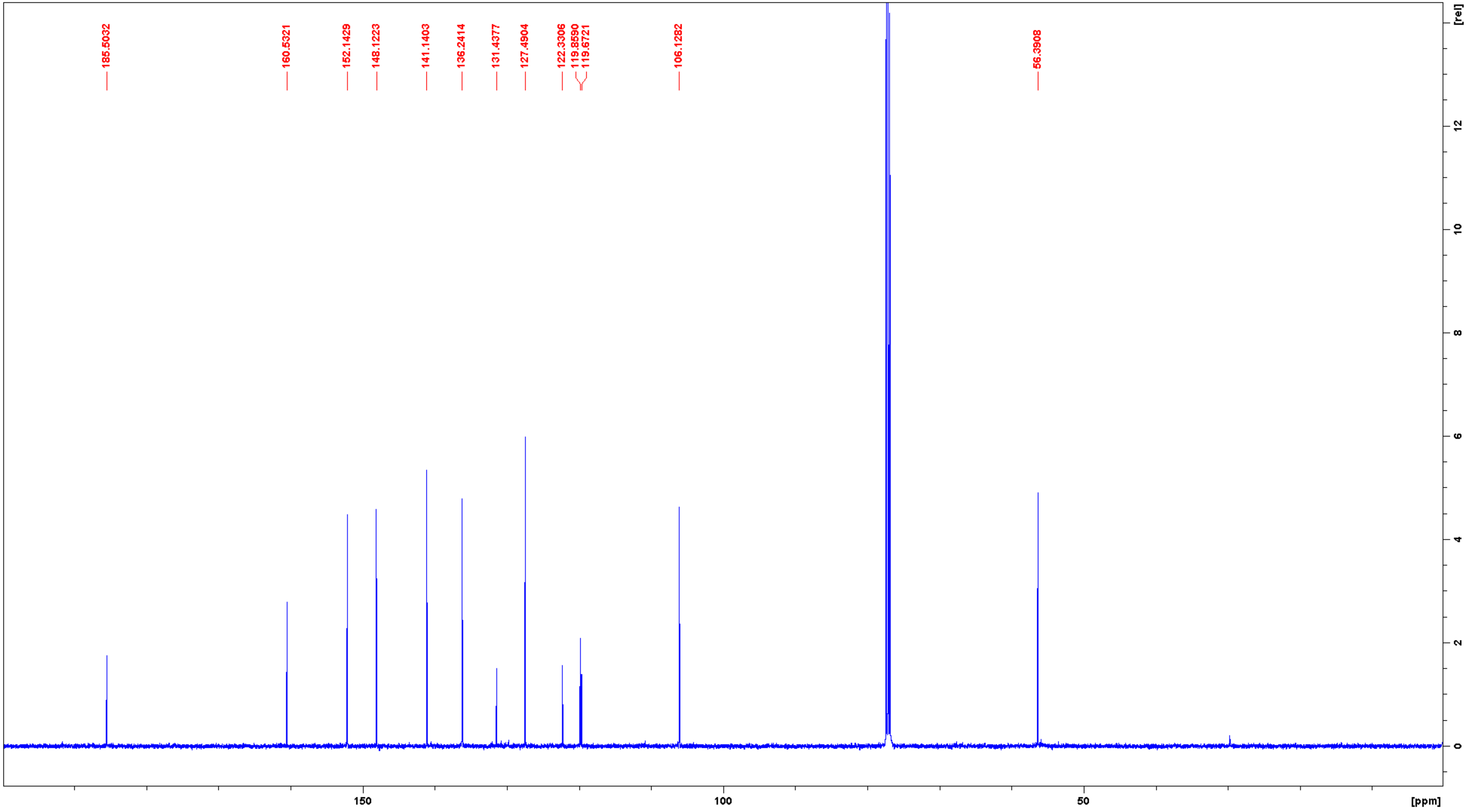

^{1}H NMR spectrum (500 MHz, $CDCl_3$) of compound **37**:

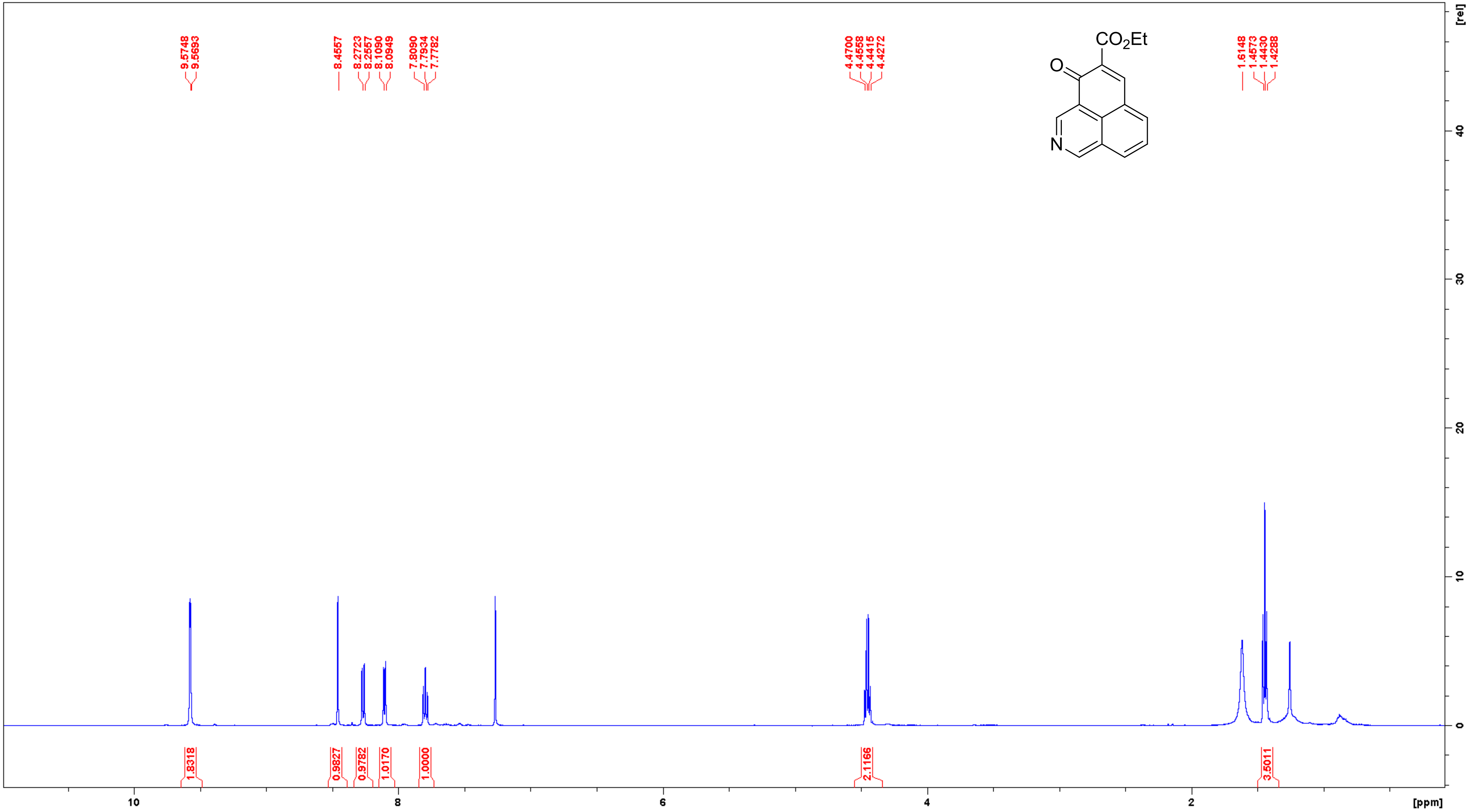

^{13}C NMR spectrum (125 MHz, $CDCl_3$) of compound **37**:

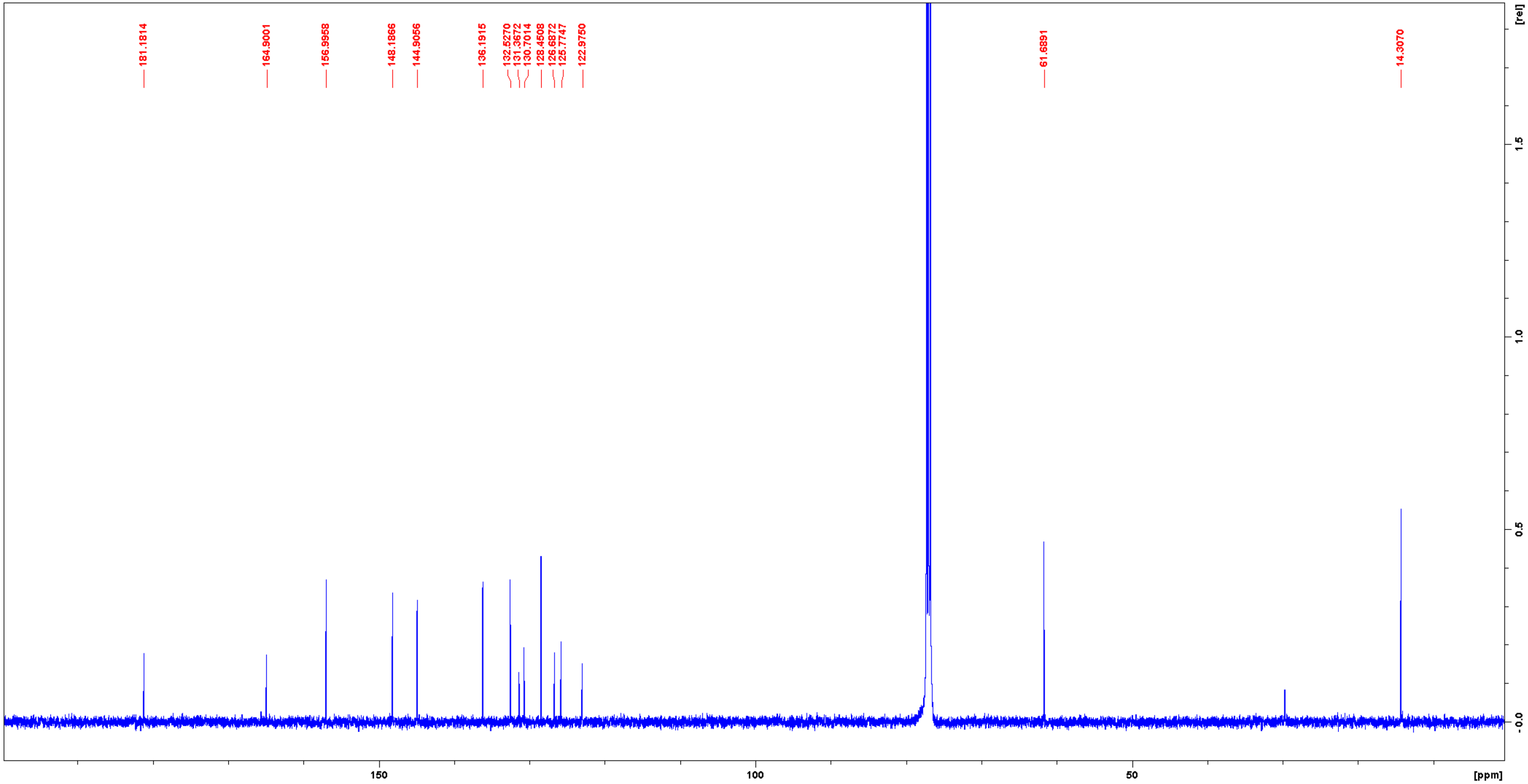

^{1}H NMR spectrum (500 MHz, $CDCl_3$) of compound **38**:

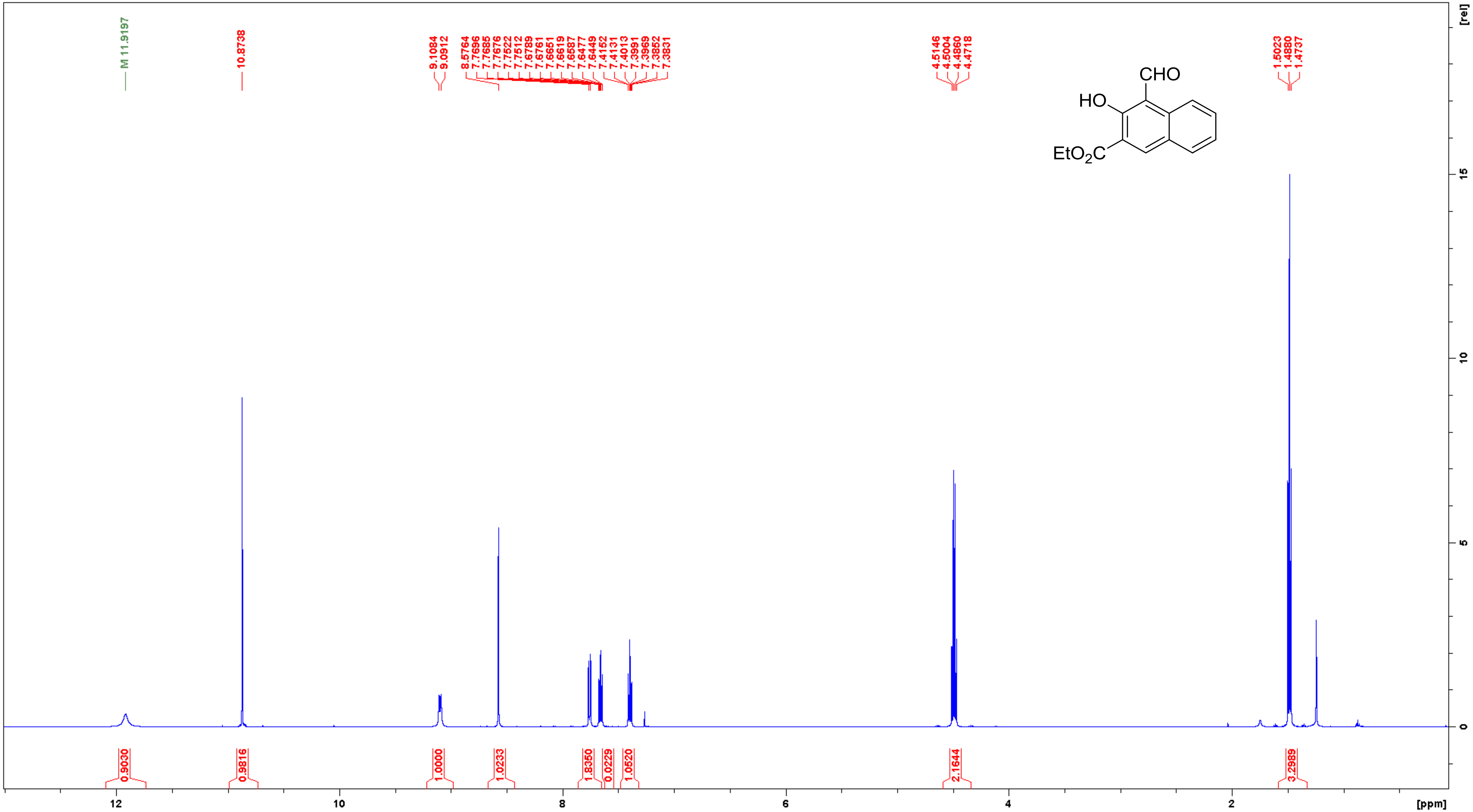

^{1}H NMR spectrum (500 MHz, $CDCl_3$) of compound **40**:

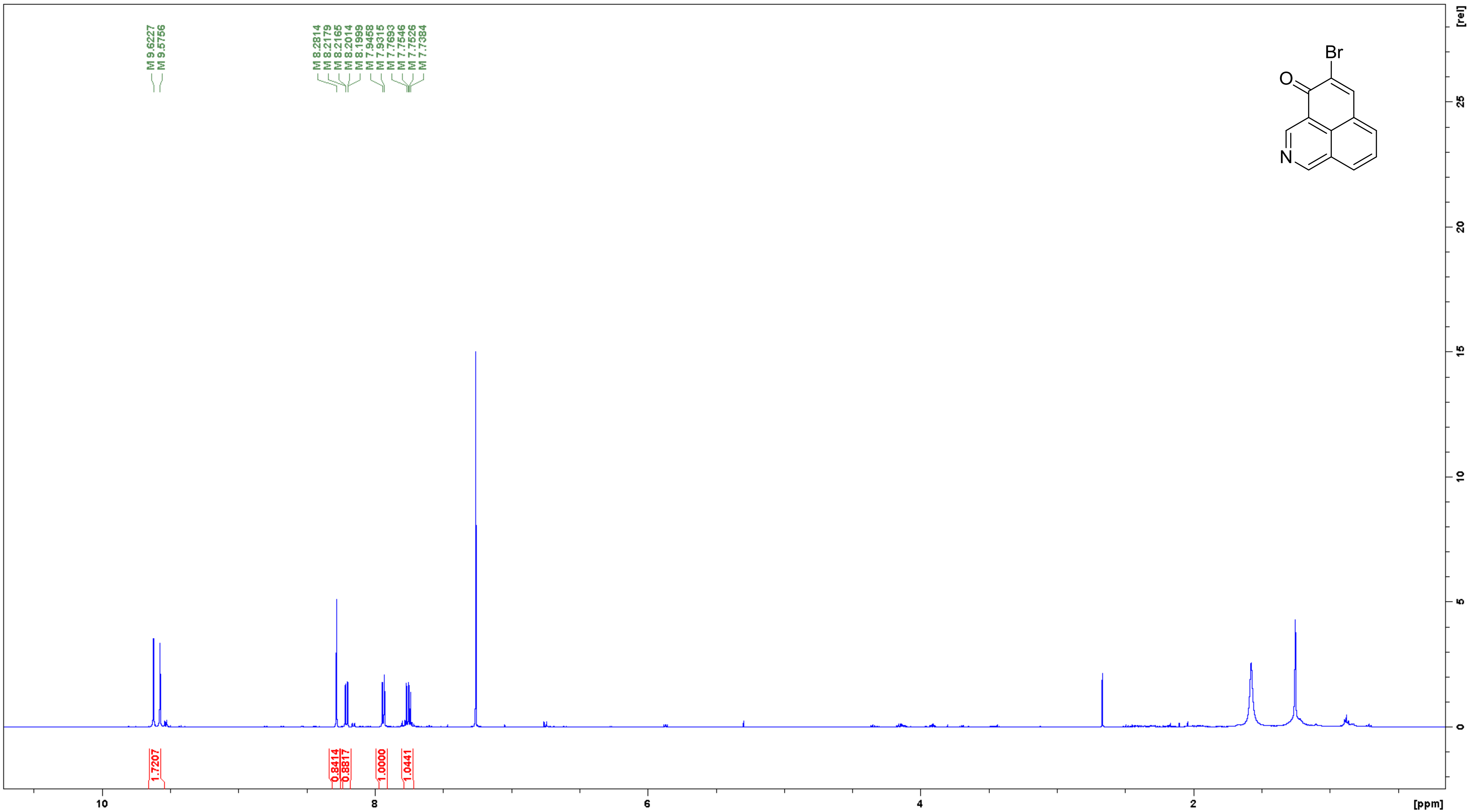

Expanded ^1H NMR spectrum (500 MHz, $CDCl_3$) of compound **40**:

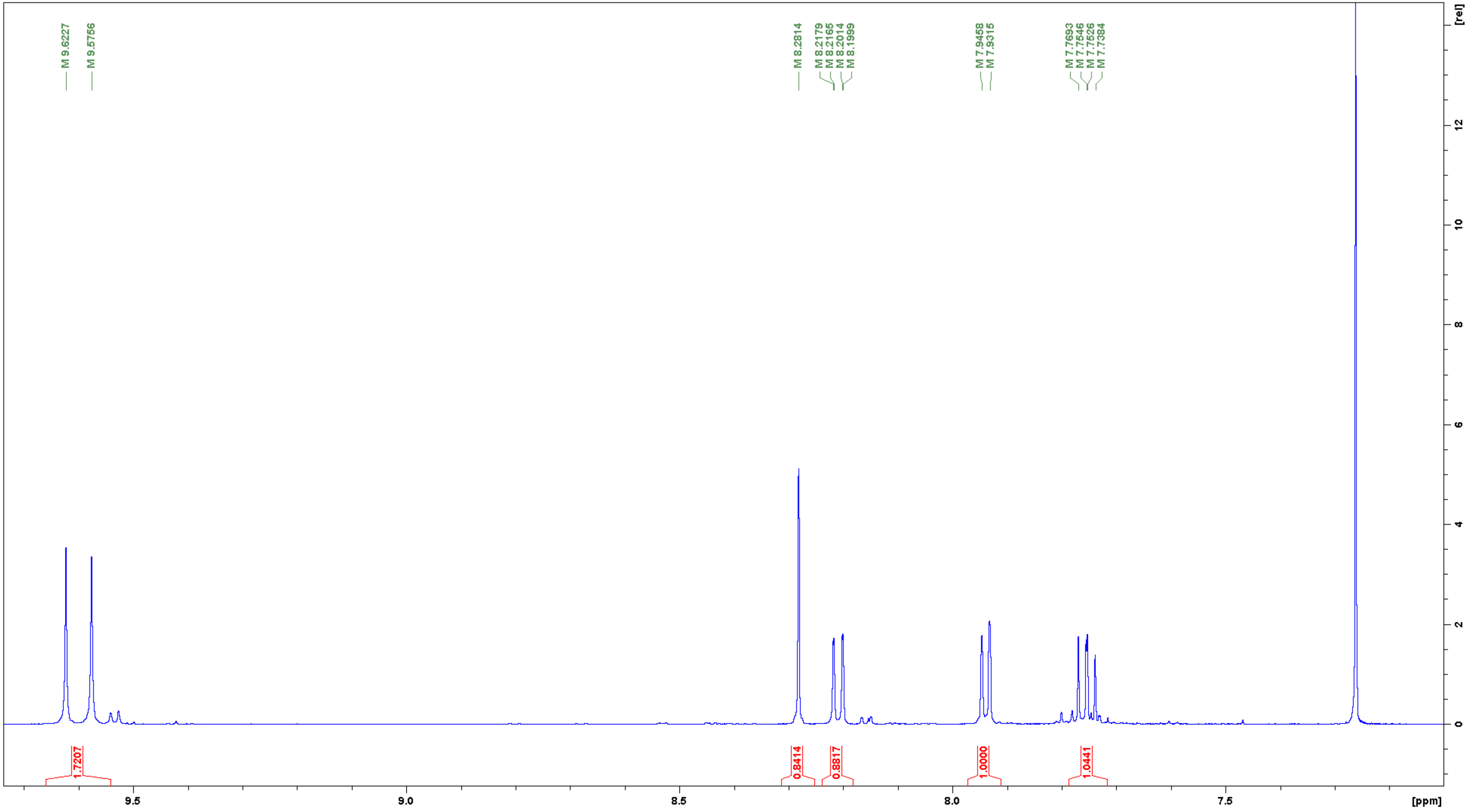

^{13}C NMR spectrum (125 MHz, $CDCl_3$) of compound **40**:

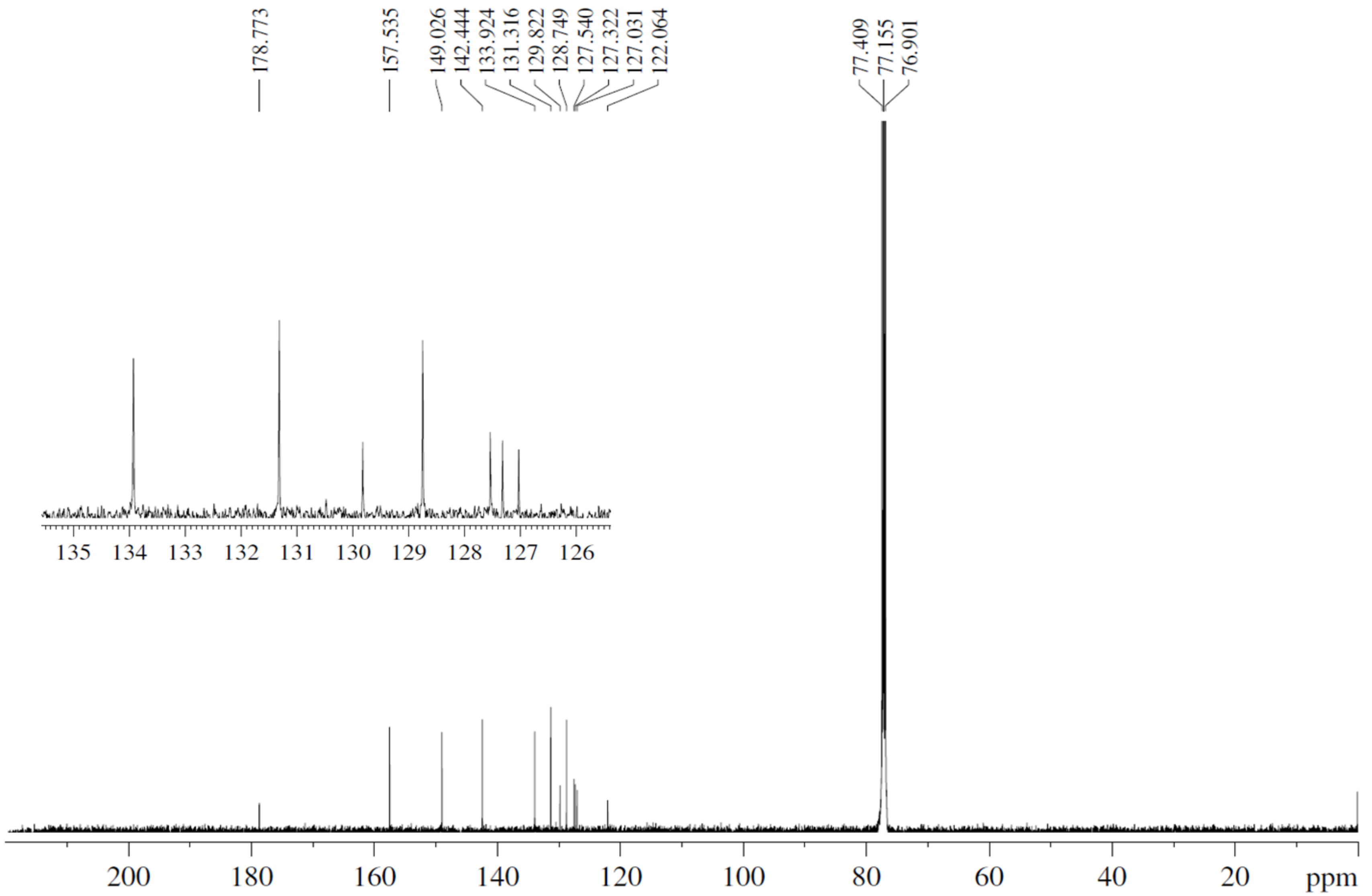

:

^{1}H NMR spectrum (500 MHz, $CDCl_3$) of compound **41**:

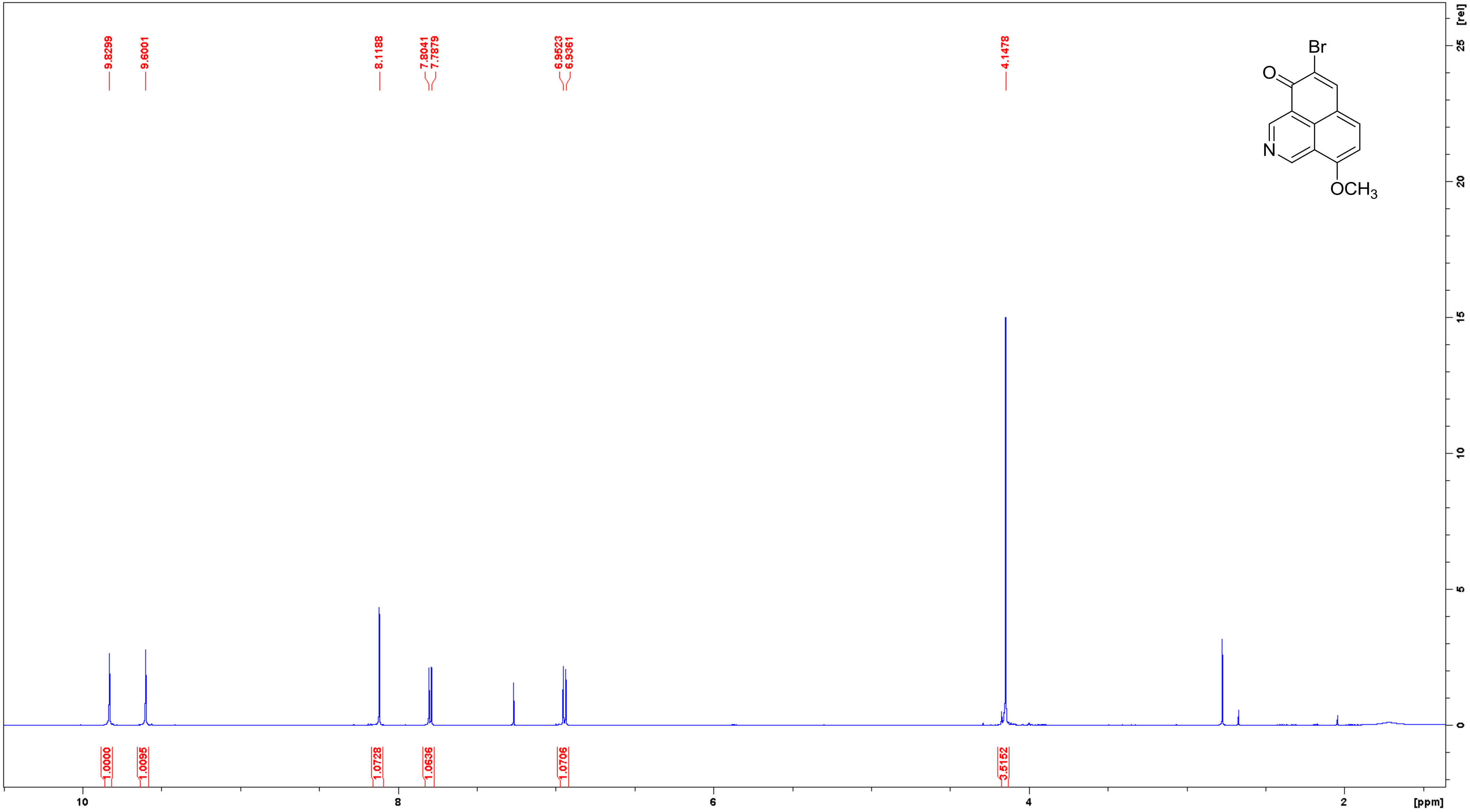

Expanded ^{1}H NMR spectrum (500 MHz, $CDCl_3$) of compound **41**:

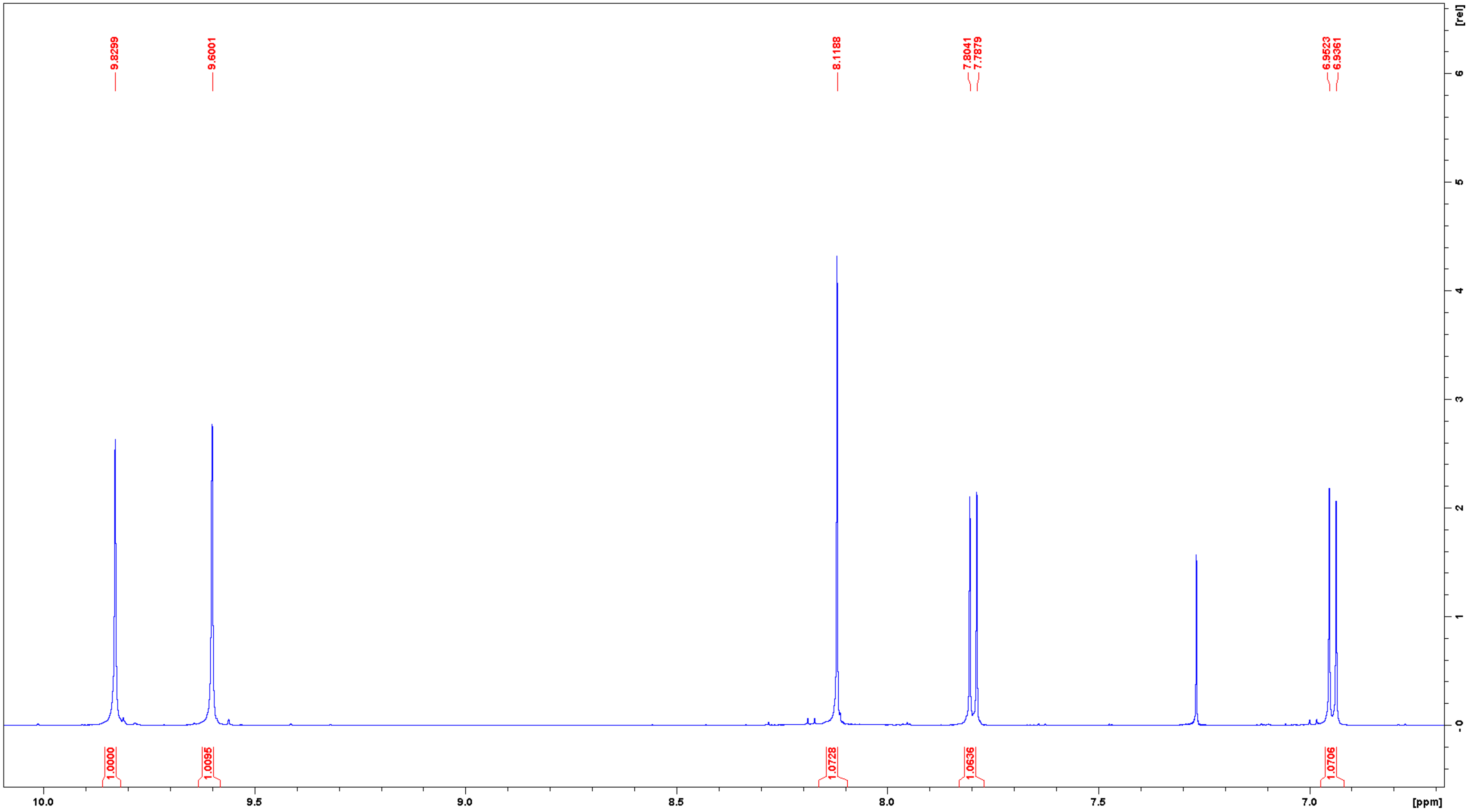

^{13}C NMR spectrum (125 MHz, $CDCl_3$) of compound **41**:

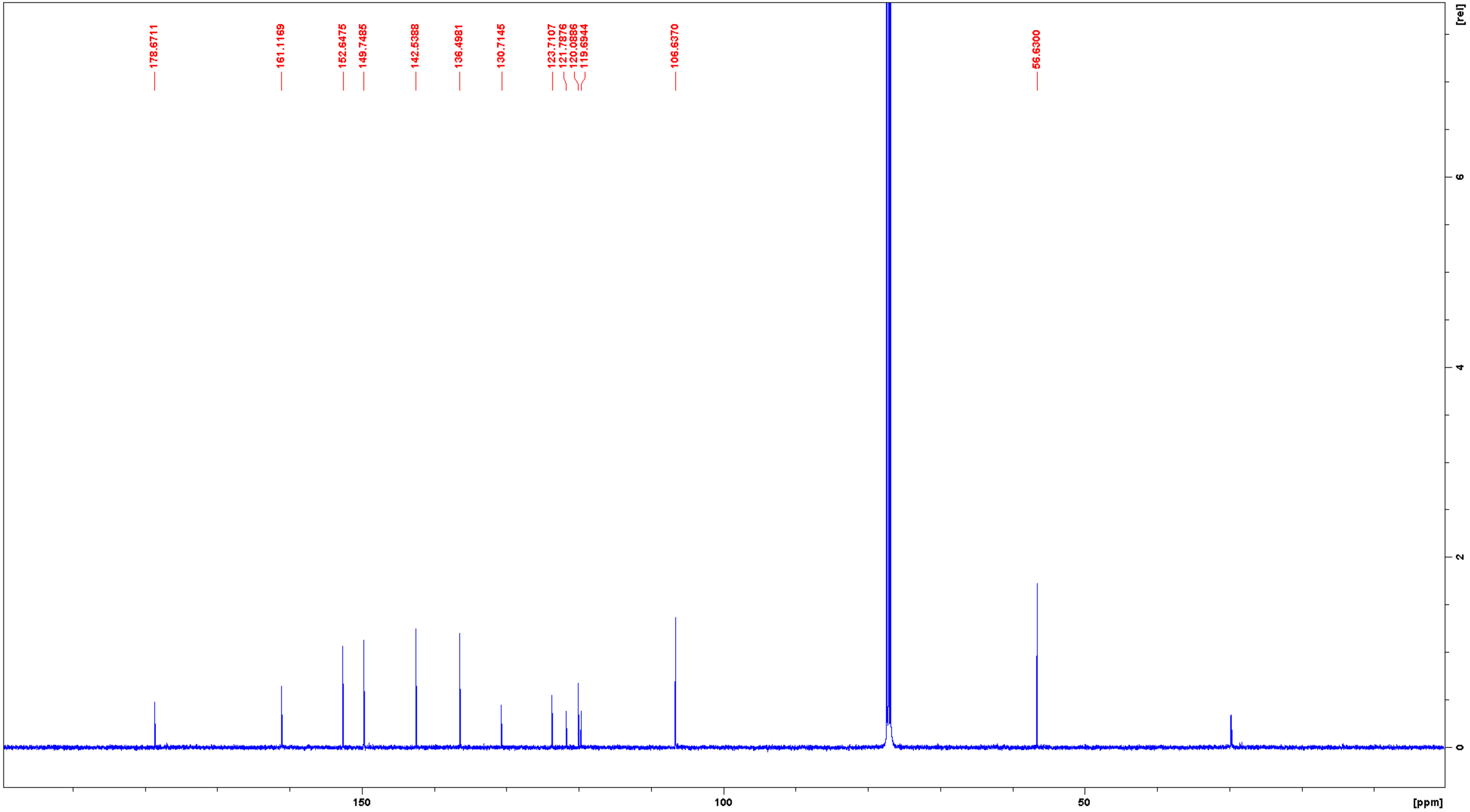

Expanded ^{13}C NMR spectrum (125 MHz, $CDCl_3$) of compound **41**:

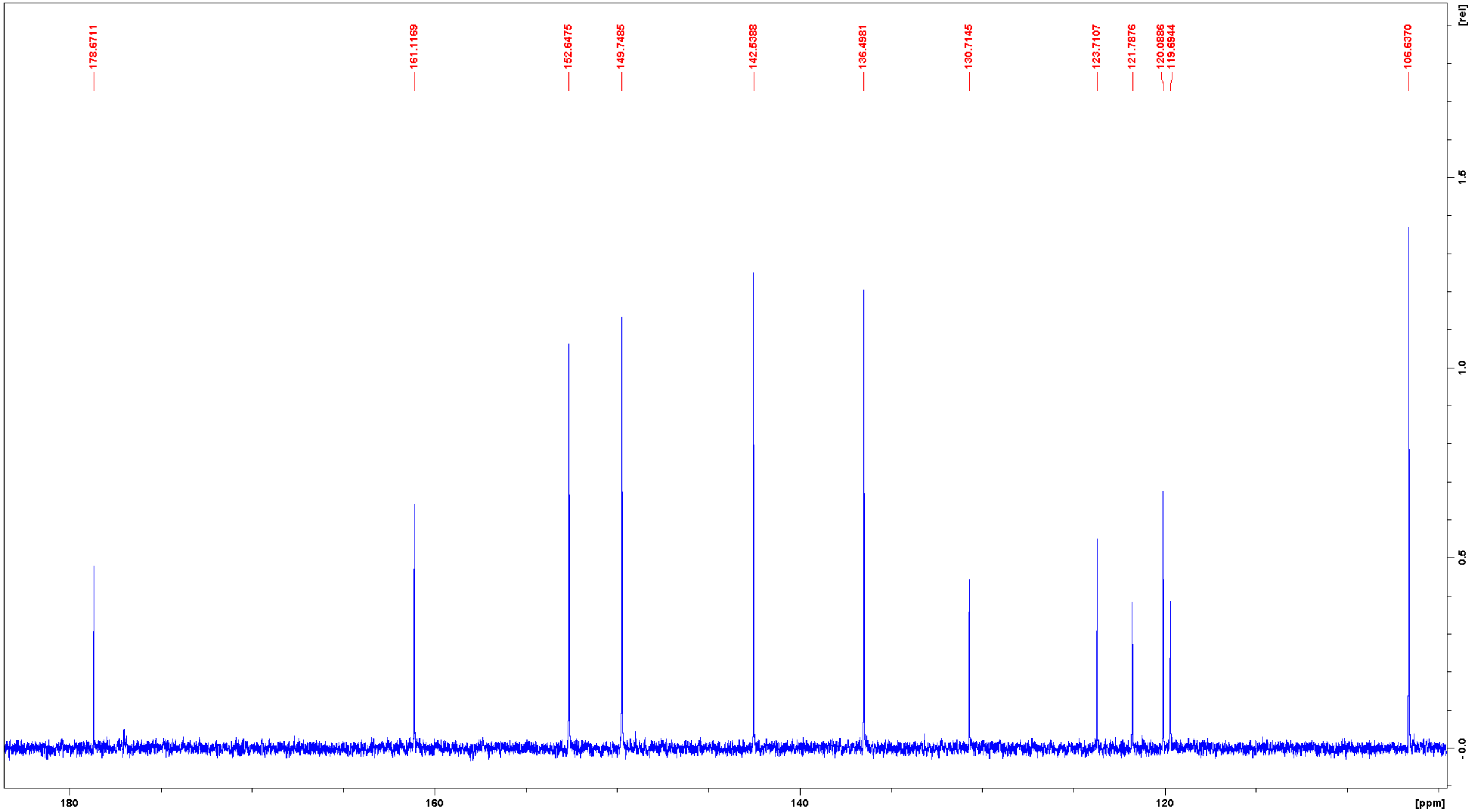

4. ¹H NMR, ¹³C NMR, COSY, HSQC and HMBC spectra of naphtho[1,8-*ab*]phenoxazine (26)

¹H NMR spectrum (500 MHz, $CDCl_3$) of compound **26**:

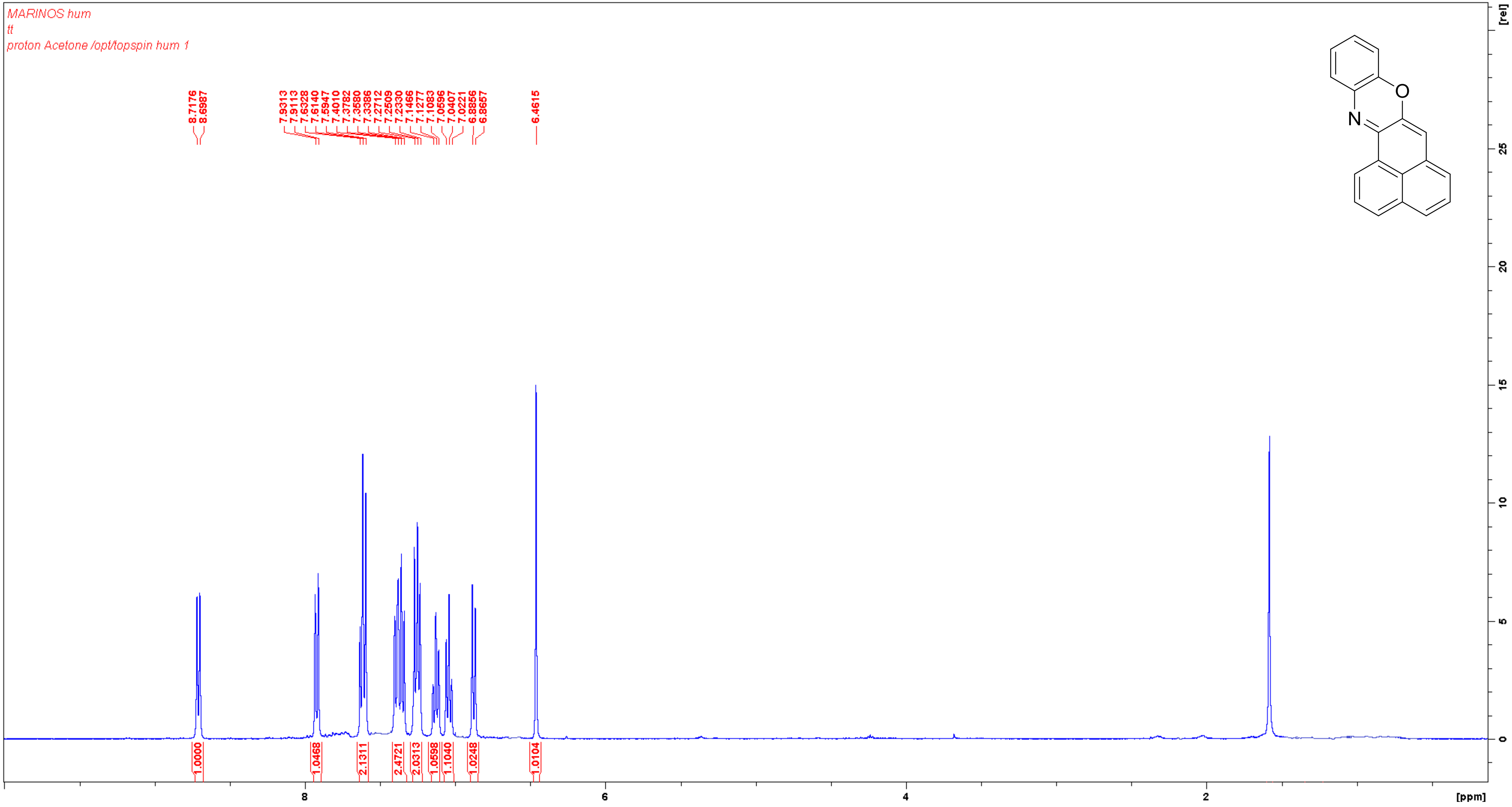

^{13}C NMR spectrum (125 MHz, $CDCl_3$) of compound **26**:

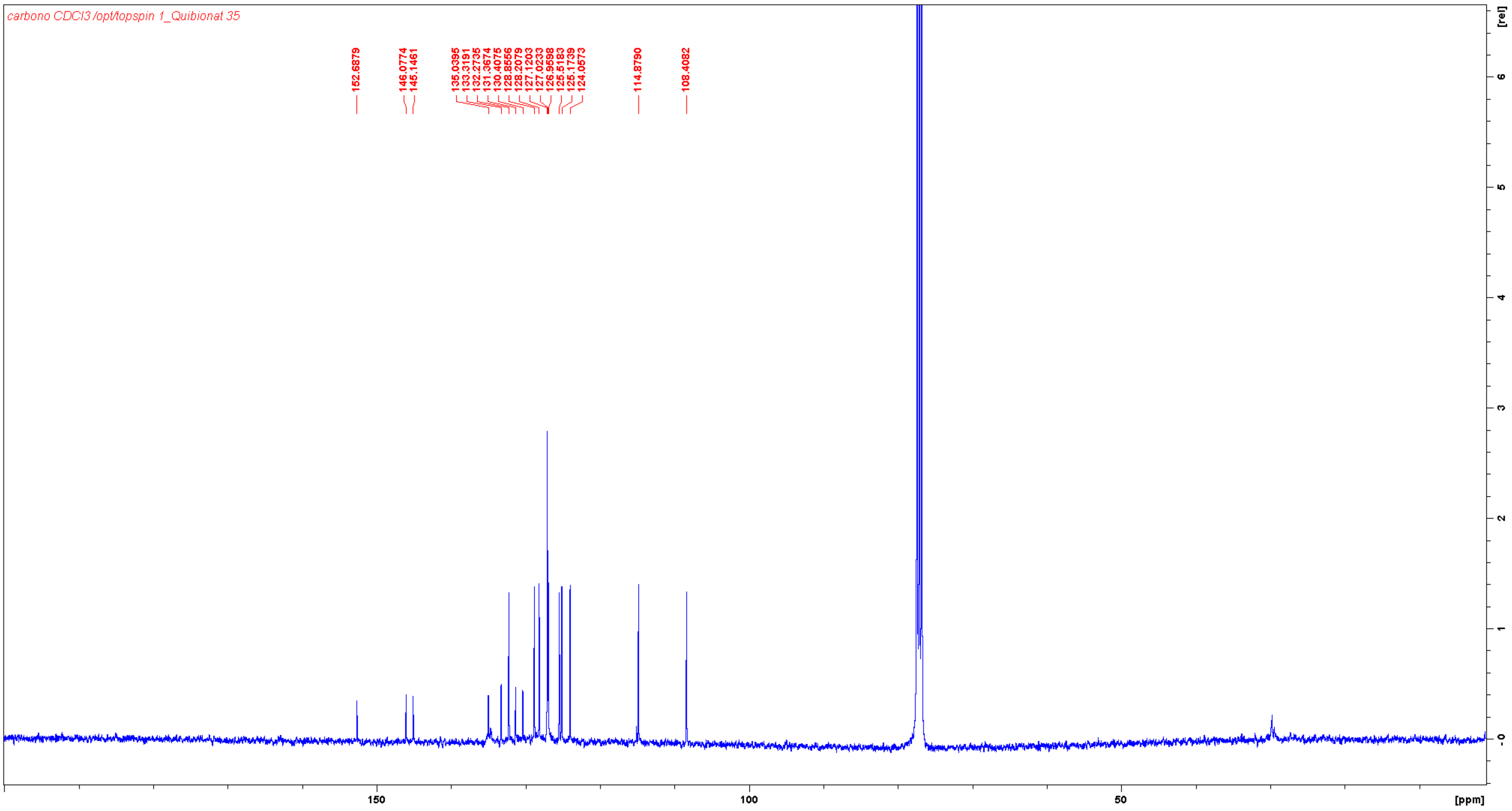

Expanded ^{13}C NMR spectrum (125 MHz, $CDCl_3$) of compound **26**:

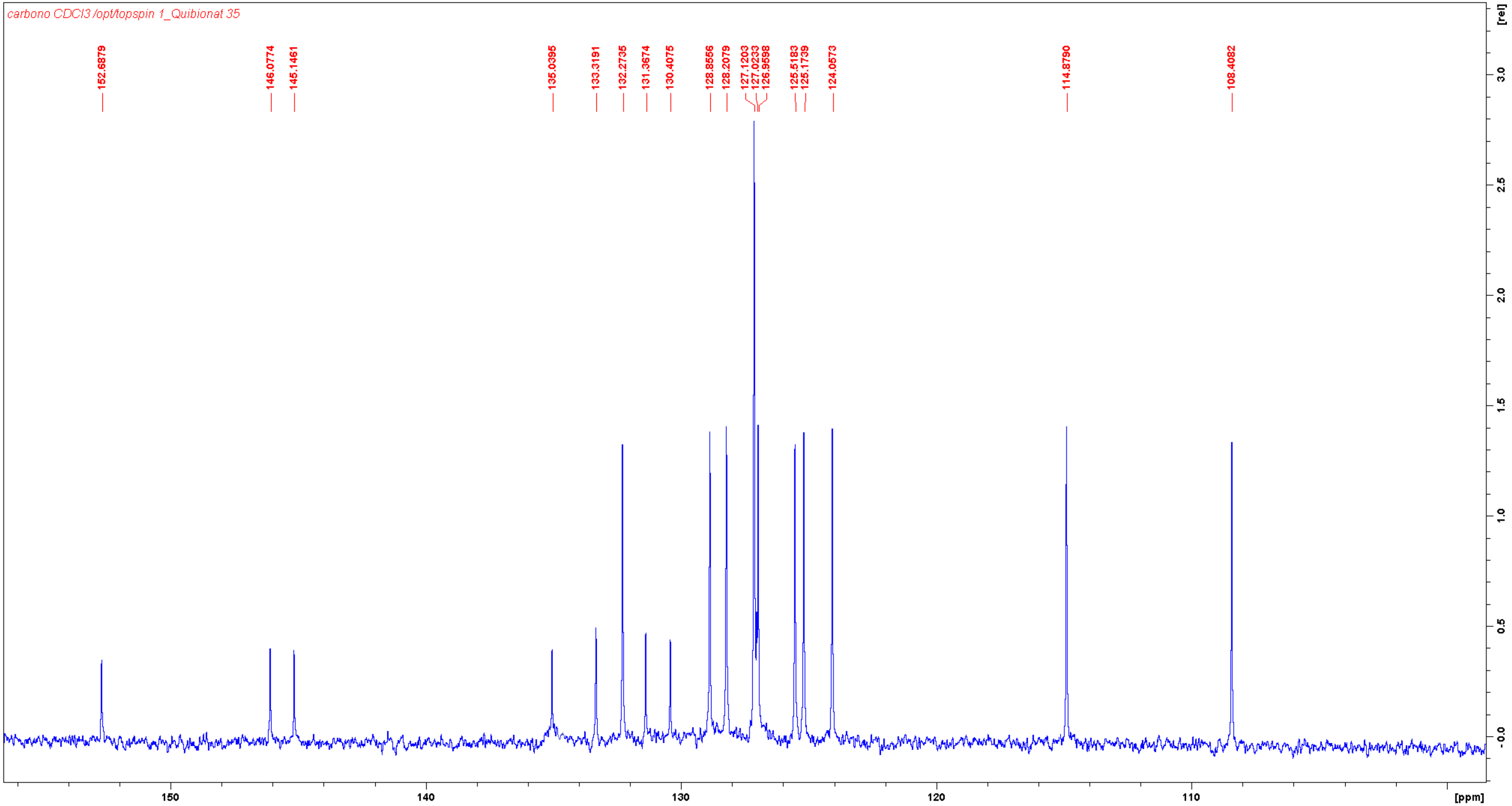

COSY spectrum (500 MHz, CDCl3,) of **26**:

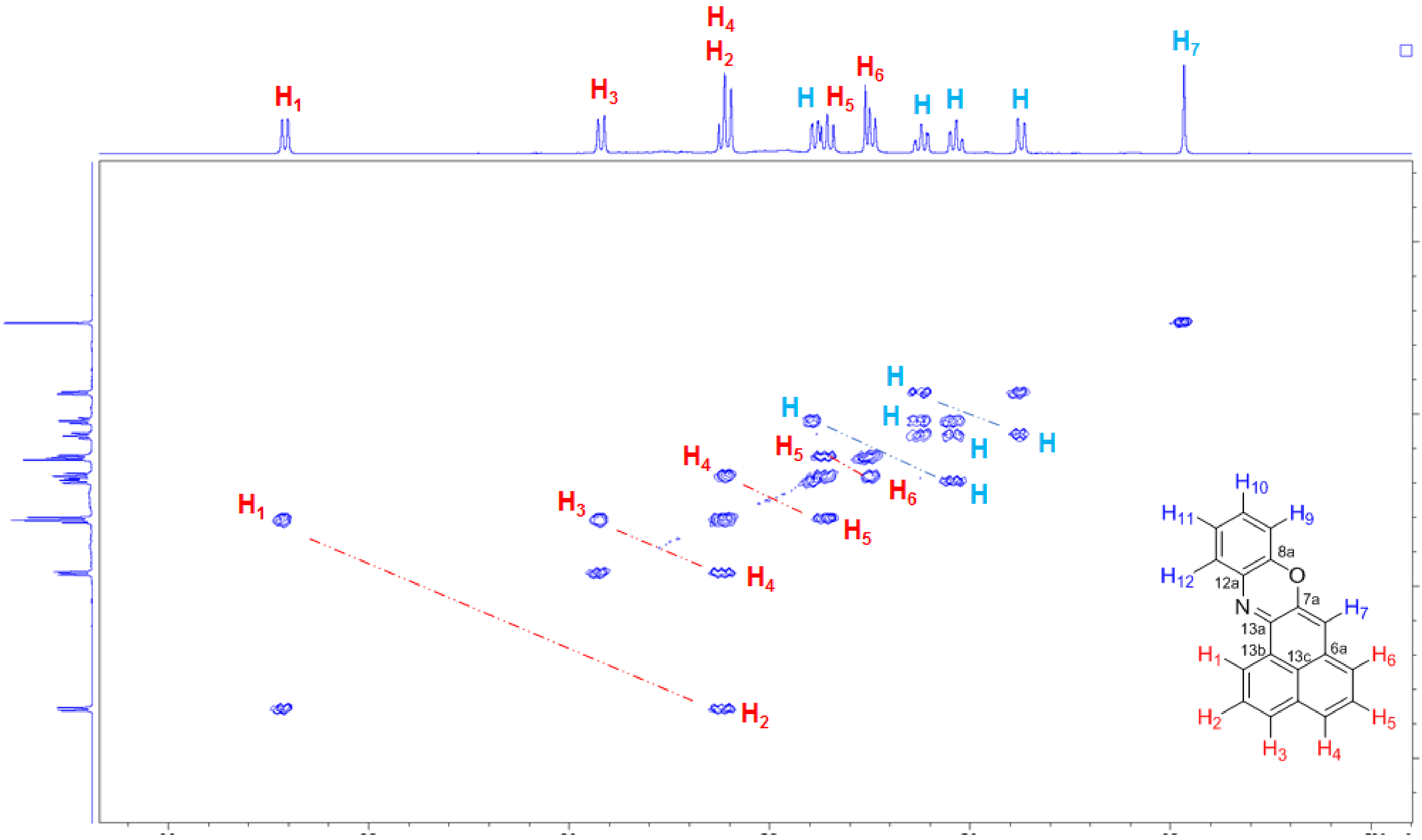

HSQC spectrum (500 MHz, $CDCl_3$) of **26**:

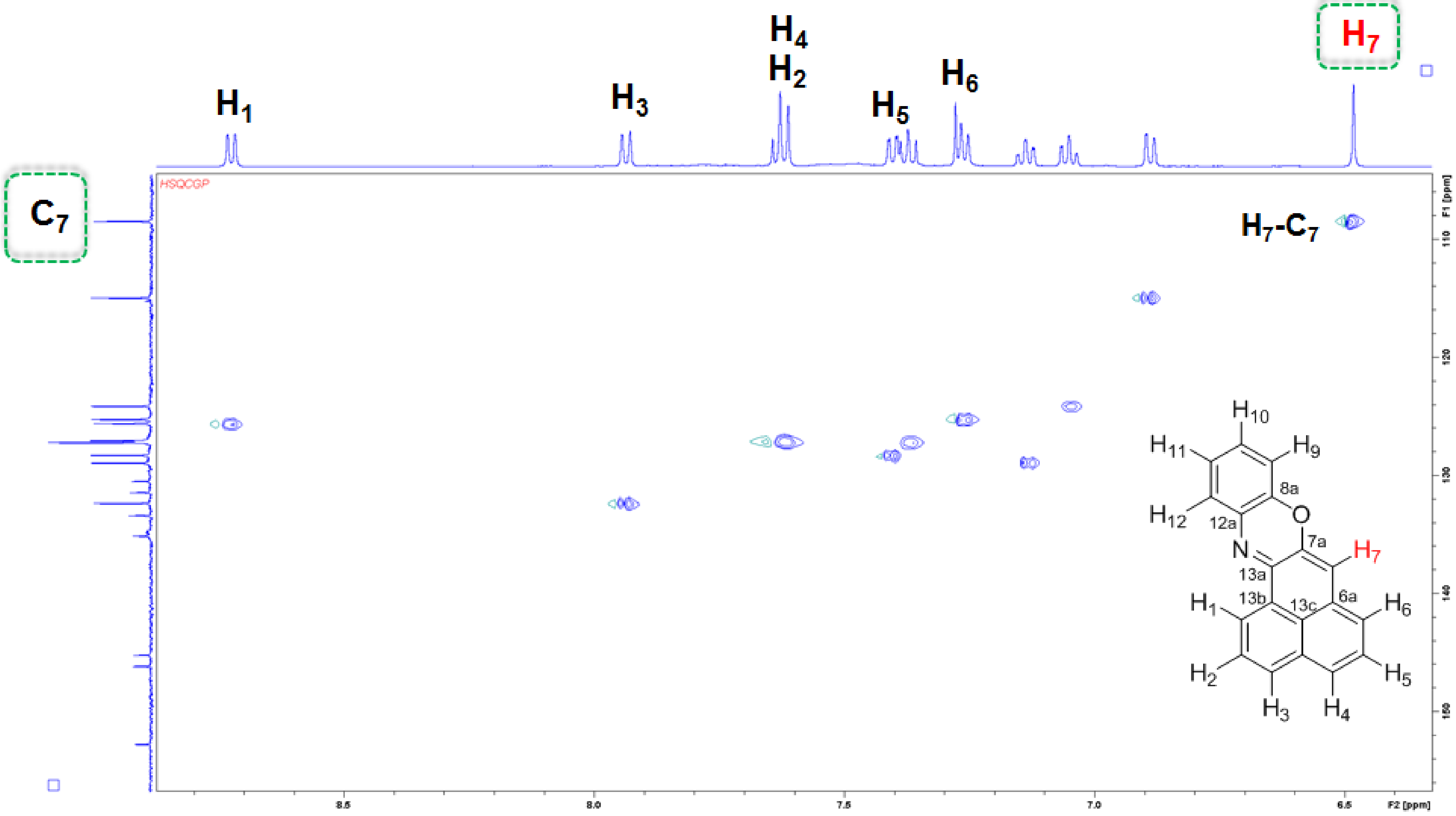

Expanded HSQC spectrum (500 MHz, $CDCl_3$) of **26**:

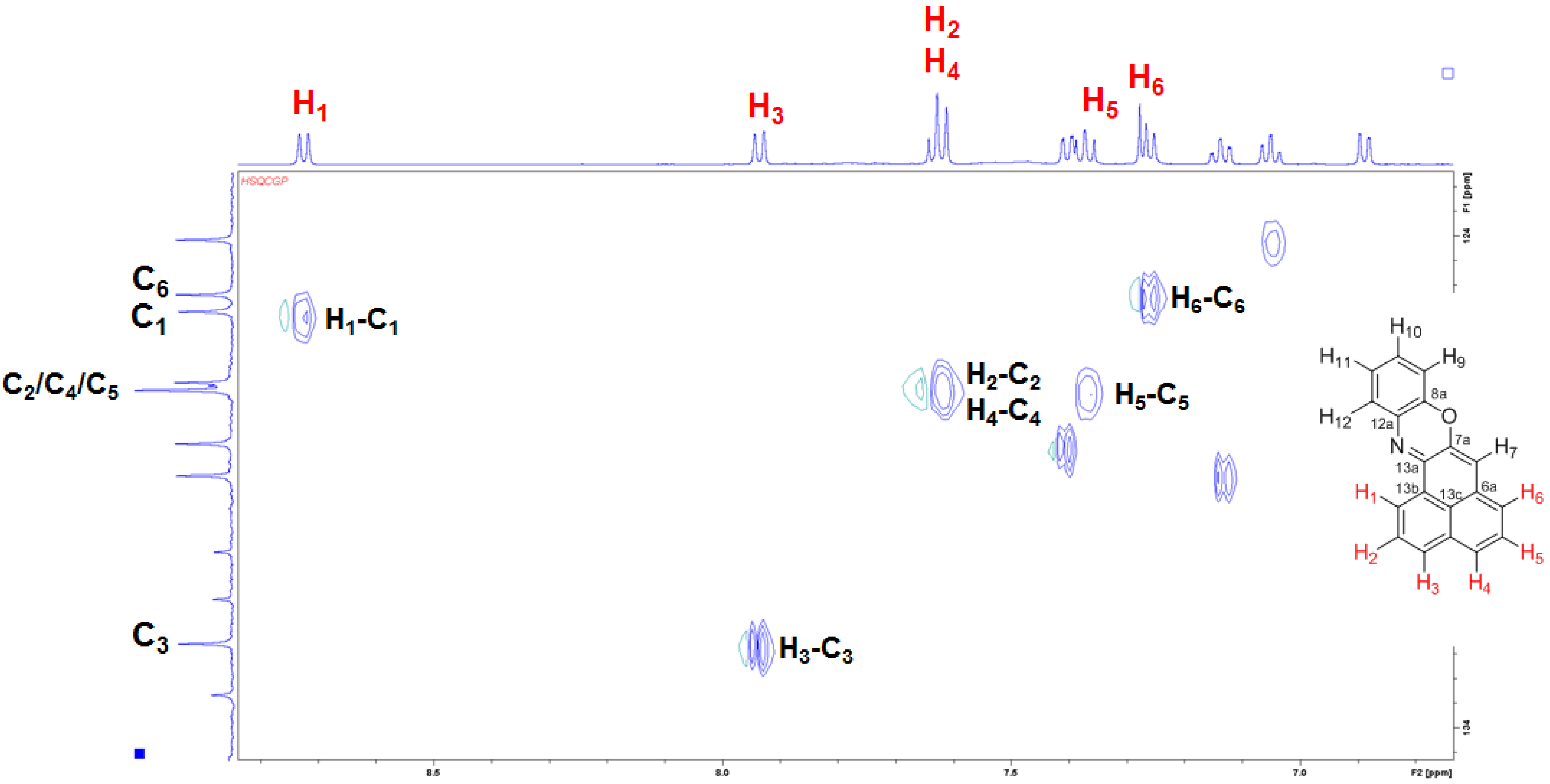

Table S1. ^1H NMR data and C-H single bond correlations (HSQC) of **26**

H	δ (ppm)	HSQC δ (ppm)
H-1	8.71	C-1 (125.5)
H-3	7.92	C-3 (132.3)
H-2	7.61	C-2 (127.2 or 126.9)
H-4	7.60	C-4 (127.2 or 126.9)
H-9 or H-12	7.38	C-9 or C-12
H-5	7.35	C-5 (127.2 or 126.9)
H-6	7.24	C-6 (125.2)
H-11 or H-10	7.12	C-11 or C-10
H-11 or H-10	7.03	C-11 or C-10
H-9 or H-12	6.87	C-9 or C-12
H-7	6.46	C-7 (108.5)

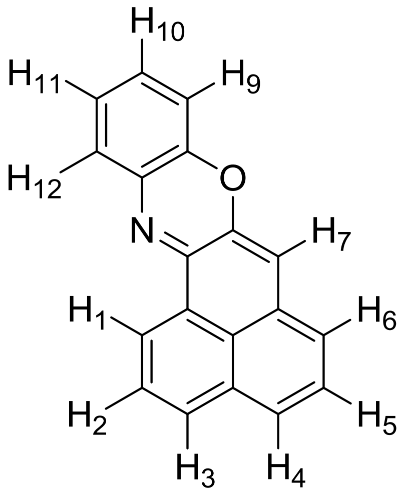

HMBC spectrum (500 MHz, $CDCl_3$) of **26**:

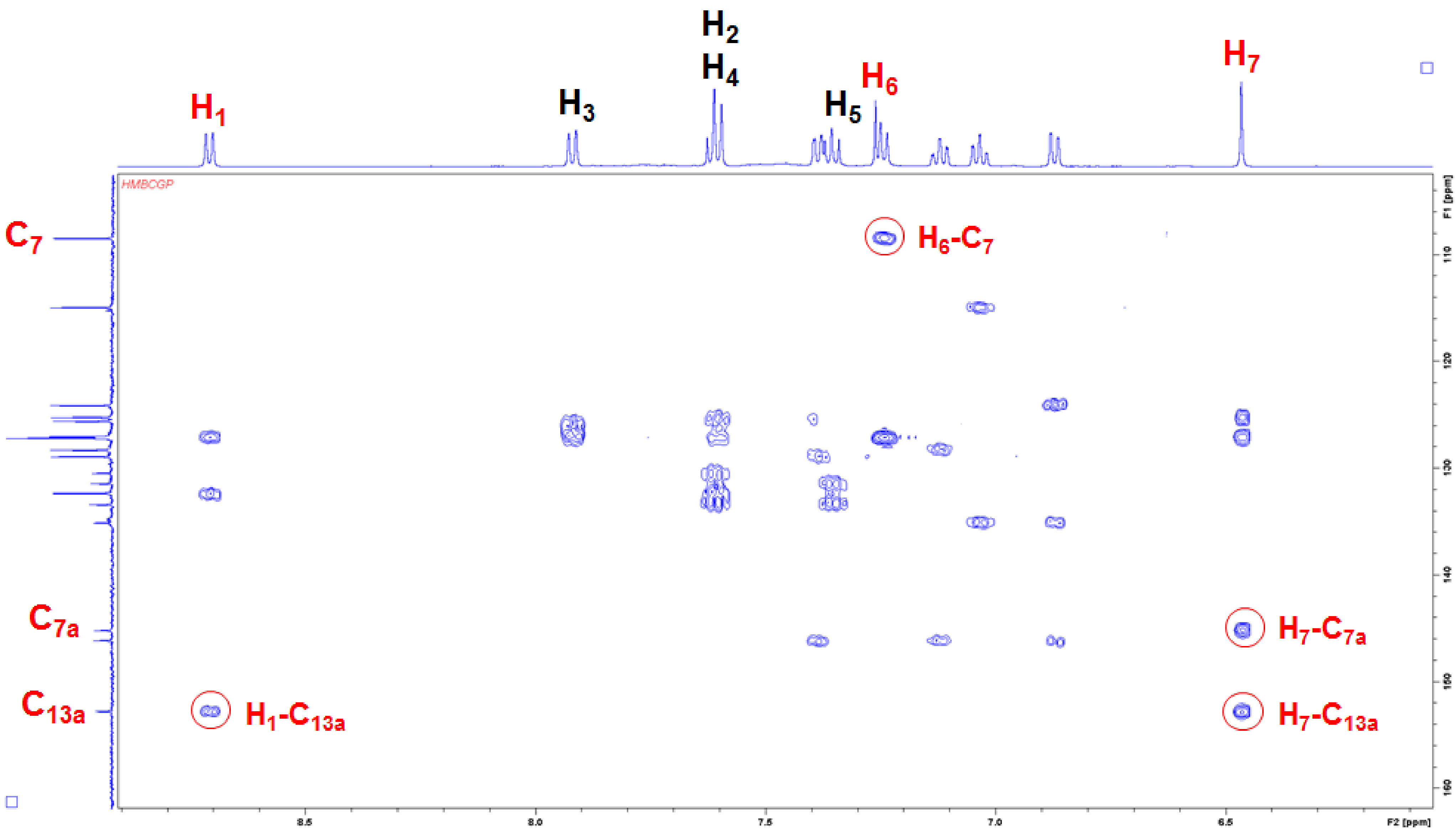

Expanded HMBC spectrum (500 MHz, $CDCl_3$) of **26**:

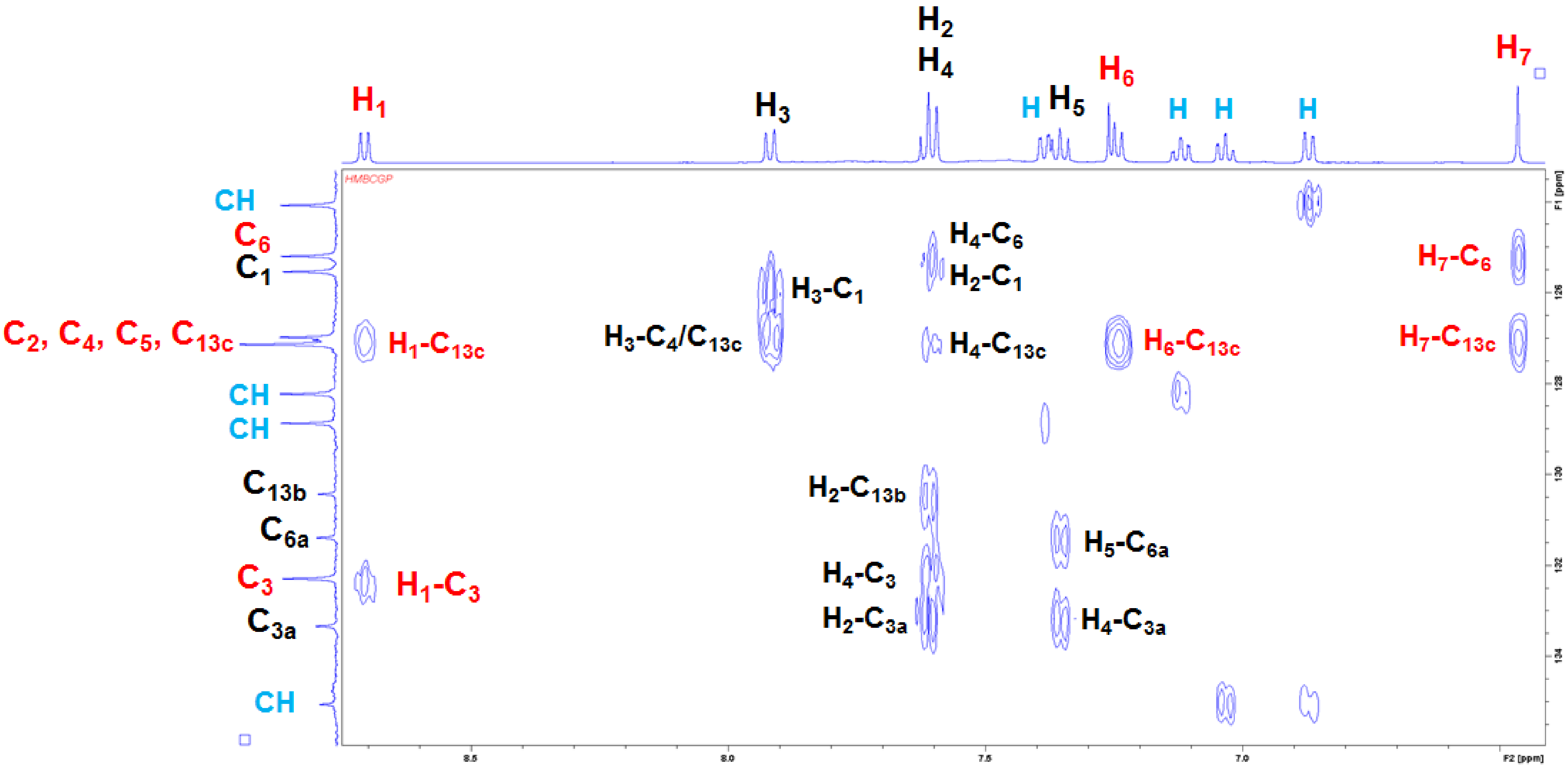

Table S2. ^{1}H NMR data and C-H correlations at three bonds of **26**

H	δ (ppm)	HMBC
H-1	8.71	C-13a, C-13c, C-3
H-3	7.92	C-1, C-13c, C-4
H-2	7.61	C-13b, C-3a, C-1*
H-4	7.60	C-13c, C-6, C-3
H-5	7.35	C-3a, C-6a
H-6	7.24	C-7, C-13c, C-4
H-7	6.46	**C-13a**, **C-13c**, **C-6**, **C-7a***

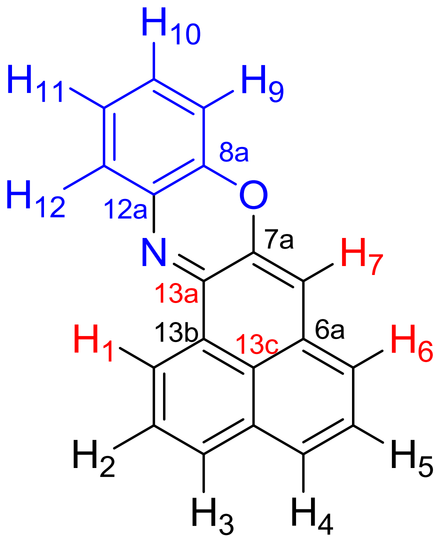

* Two bonds correlations

5. ^{1}H NMR, ^{13}C NMR, COSY, HSQC and HMBC spectra of 7*H*-naphtho[1,8-*bc*]acridin-7-one (27)

^{1}H NMR spectrum (500 MHz, $CDCl_3$) of compound **27**:

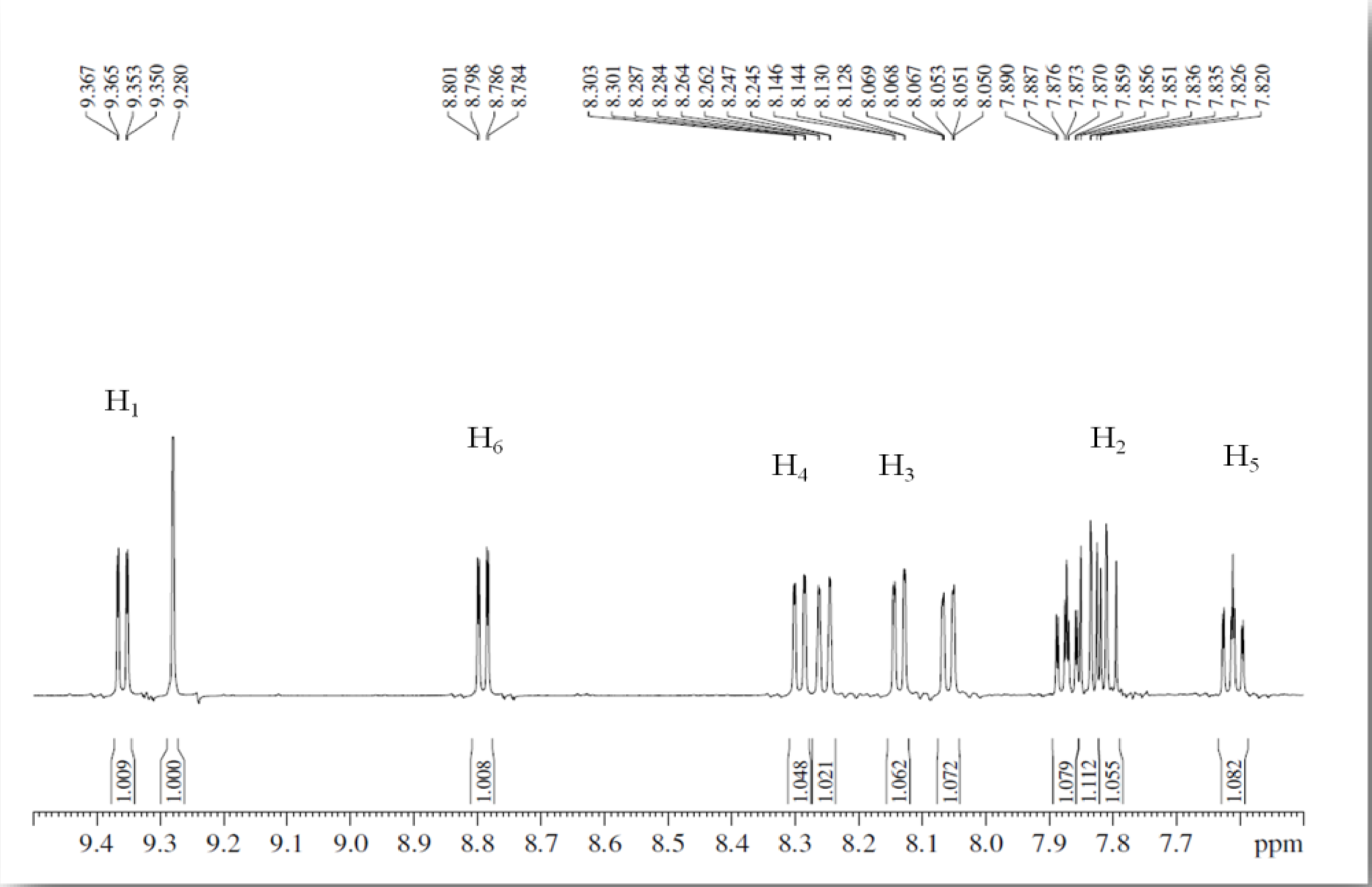

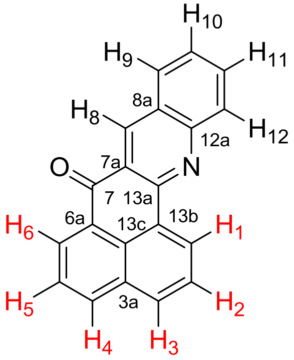

^{13}C NMR spectrum (125 MHz, CDCl3) of compound **27**:

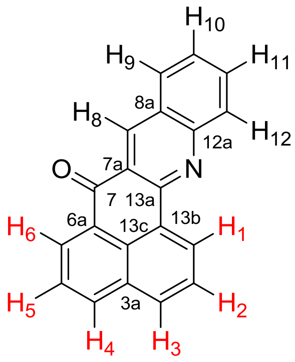

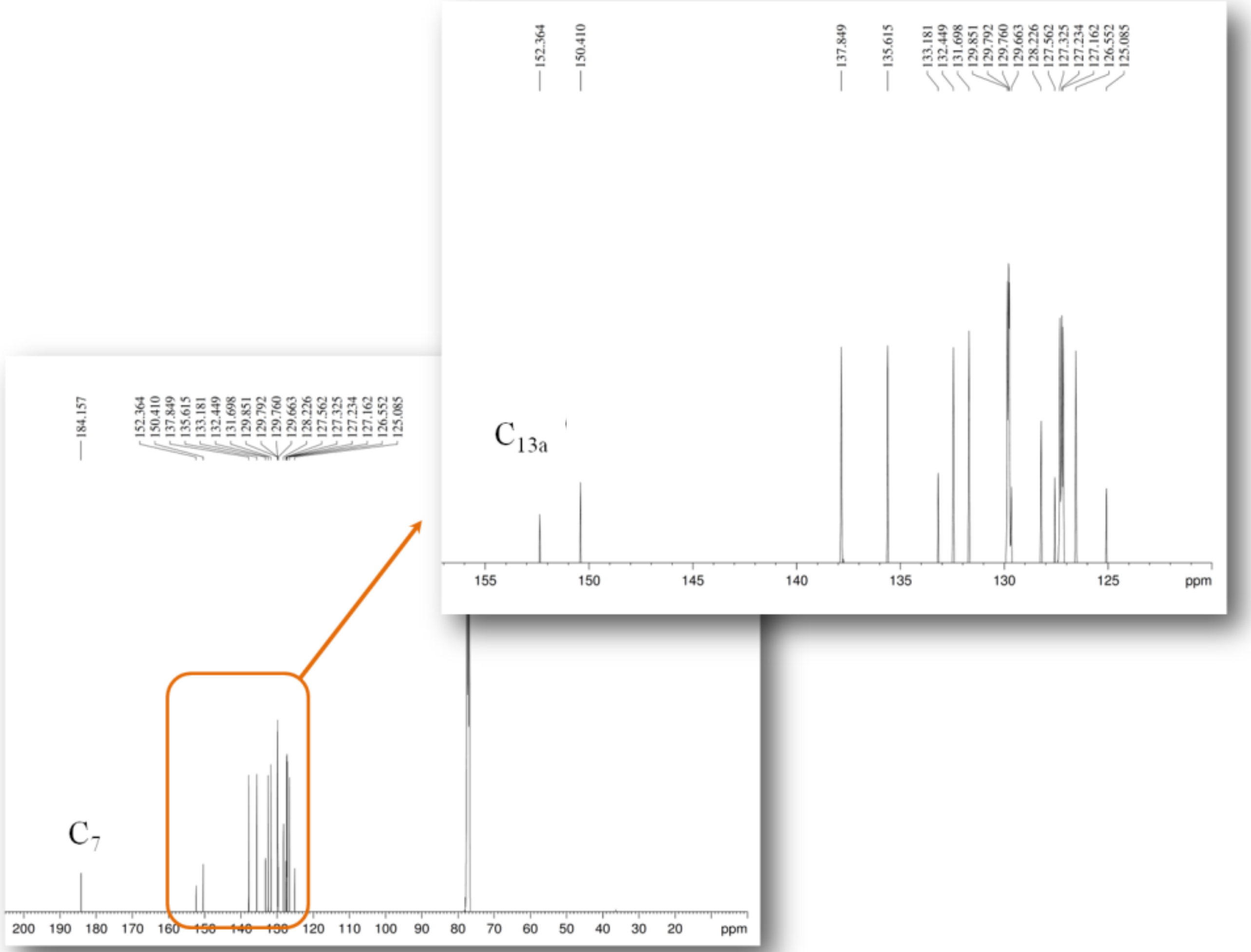

COSY spectrum (500 MHz, $CDCl_3$) of compound **27**:

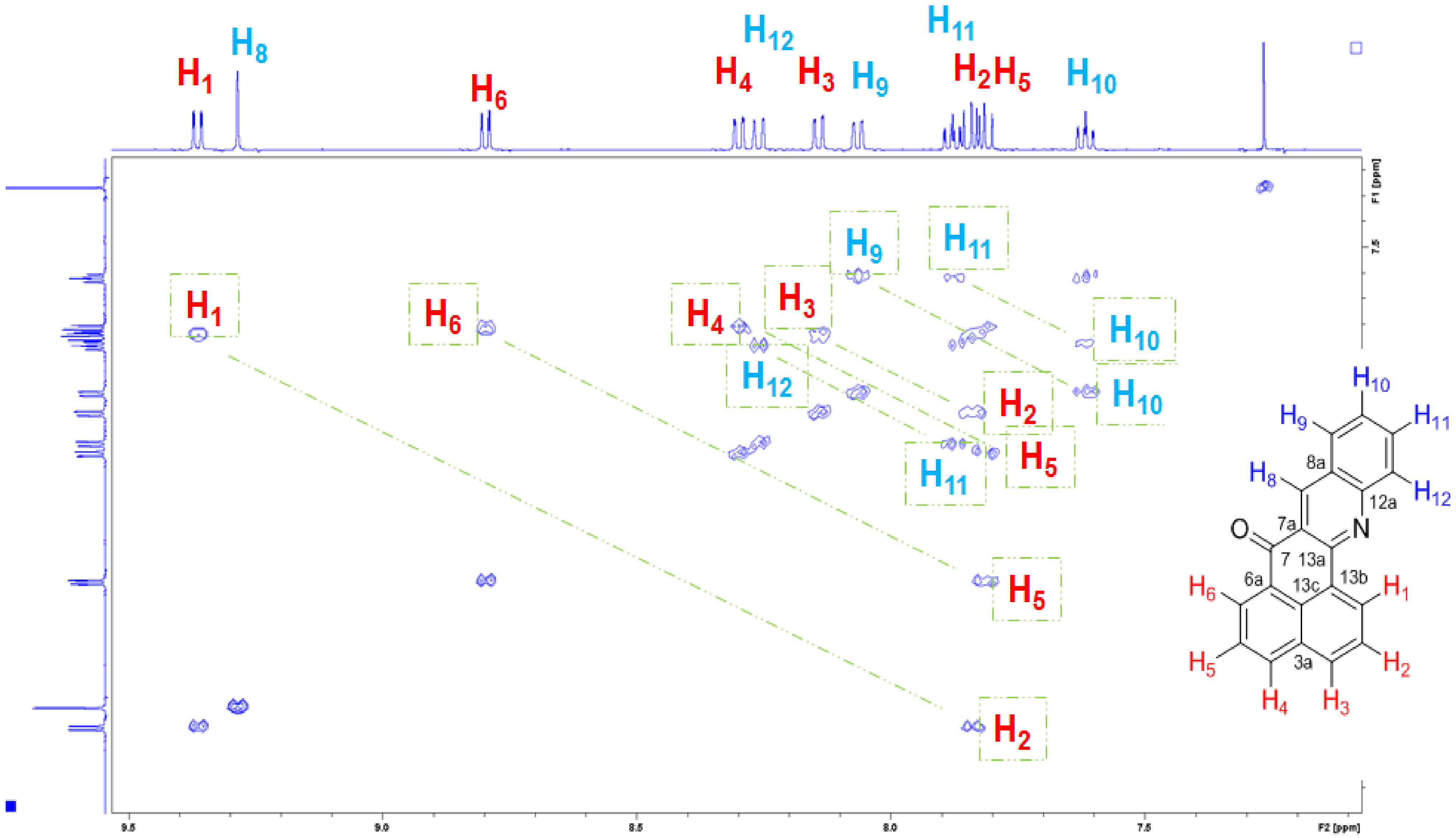

HSQC spectrum (500 MHz, $CDCl_3$) of compound **27**:

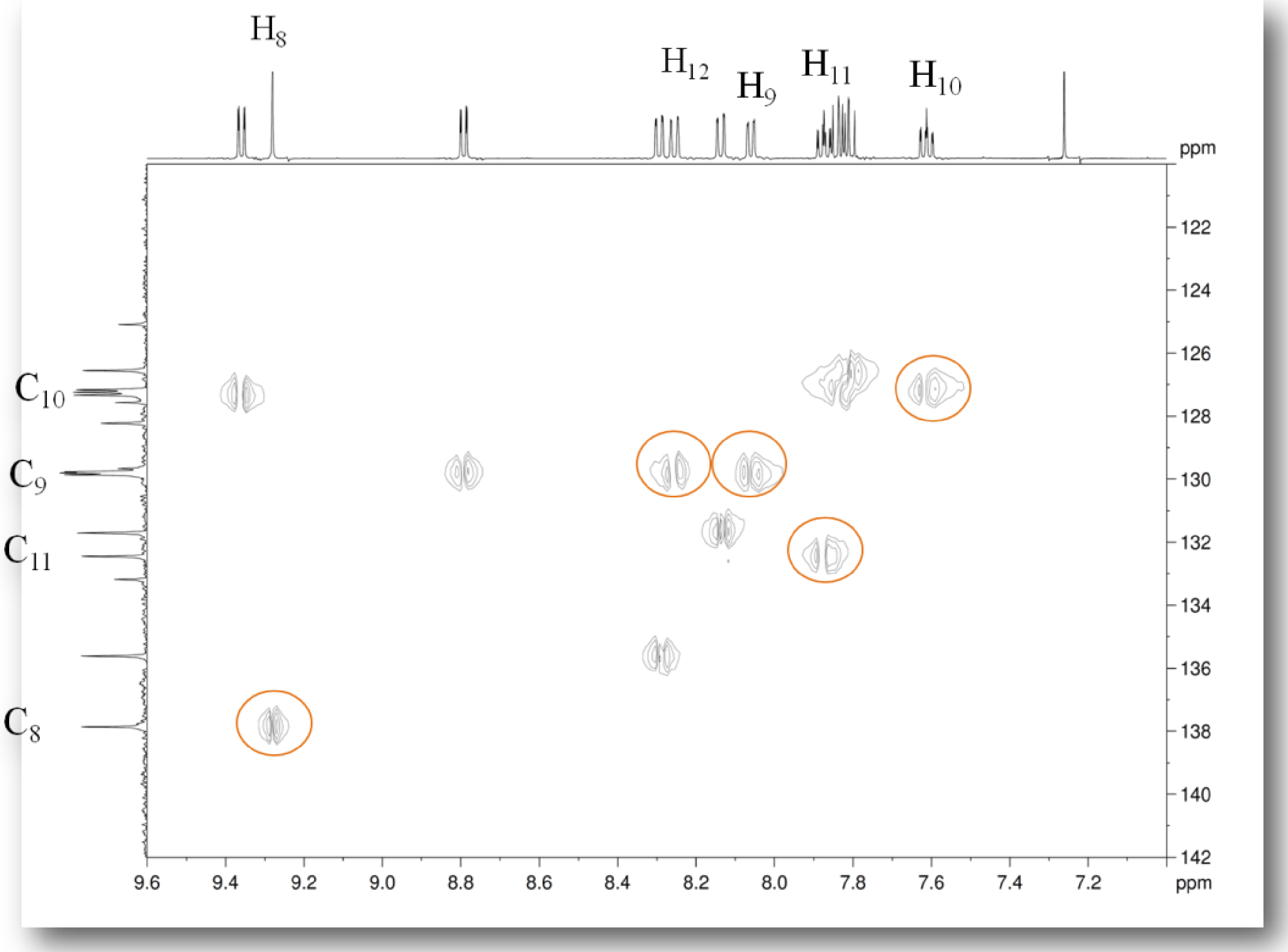

Table S3. ^{1}H NMR data and correlations C-H single bond correlations (HSQC) of **27**

H	δ (ppm)	HSQC δ (ppm)
H-1	9.36	C-1 (127.3)
H-8	9.28	C-8 (137.8)
H-6	8.79	C-6 (129.9 or 129.8)
H-4	8.29	C-4 (135.6)
H-12	8.25	C-12 (129.9 or 129.8)
H-3	8.14	C-3 (131.7)
H-9	8.06	C-9 (129.6)
H-11	7.88-7.85	C-11 (132.4)
H-2	7.83	C-2 (127.2)
H-5	7.81	C-5 (126.5)
H-10	7.62-7.59	C-10 (127.2)

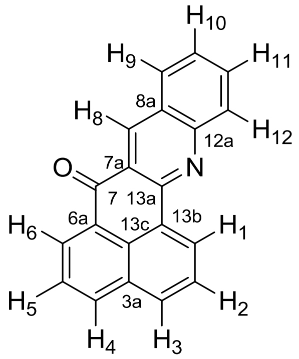

HMBC spectrum (500 MHz, CDCl3) of compound **27**

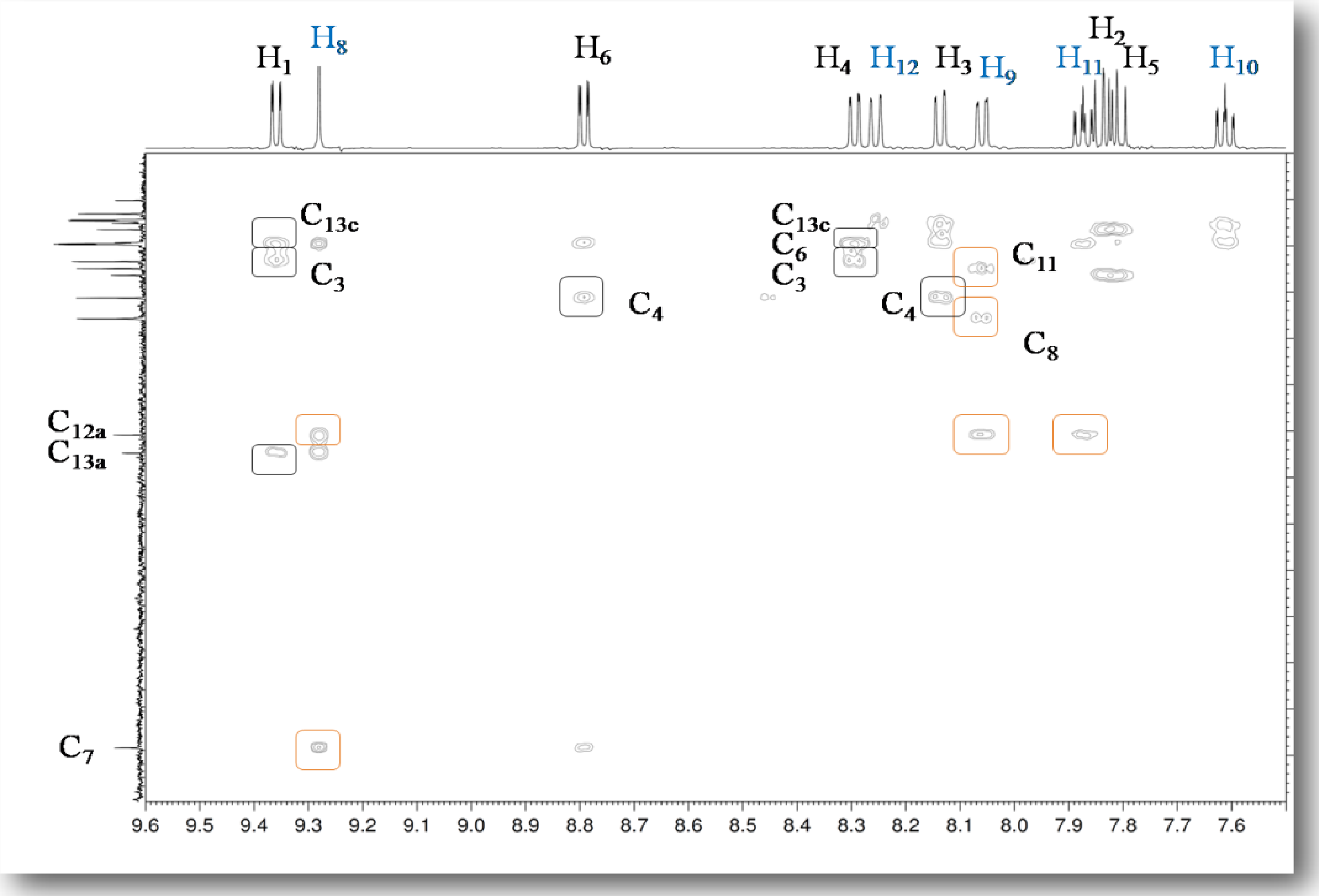

Table S4. ^{1}H NMR data and C-H three bond correlations (HMBC) of **27**

H	δ (ppm)	HMBC
H-1	9.36	C-13a, C-3,C-13c
H-8	9.28	C-9, C-12a, C-13a, C-7
H-6	8.79	C-7, C-4, C-13c
H-4	8.29	C-6,C-13c,C-3
H-12	8.25	C-10,C-8a
H-3	8.14	C-13c,C-10,C-4
H-9	8.06	C-8,C-13a,C-12a
H-11	7.88-7.85	C-9,C-12a
H-2	7.83	C-13b,C-3a
H-5	7.81	C-13c,C-3a
H-10	7.62-7.59	C-8a,C-12

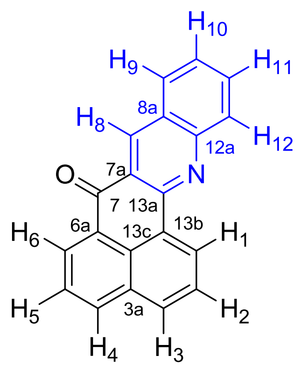

6. **^{1}H NMR, ^{13}C NMR, COSY, HSQC and HMBC spectra of compound 39**

^{1}H NMR spectrum (500 MHz, $CDCl_3$) of compound **39**:

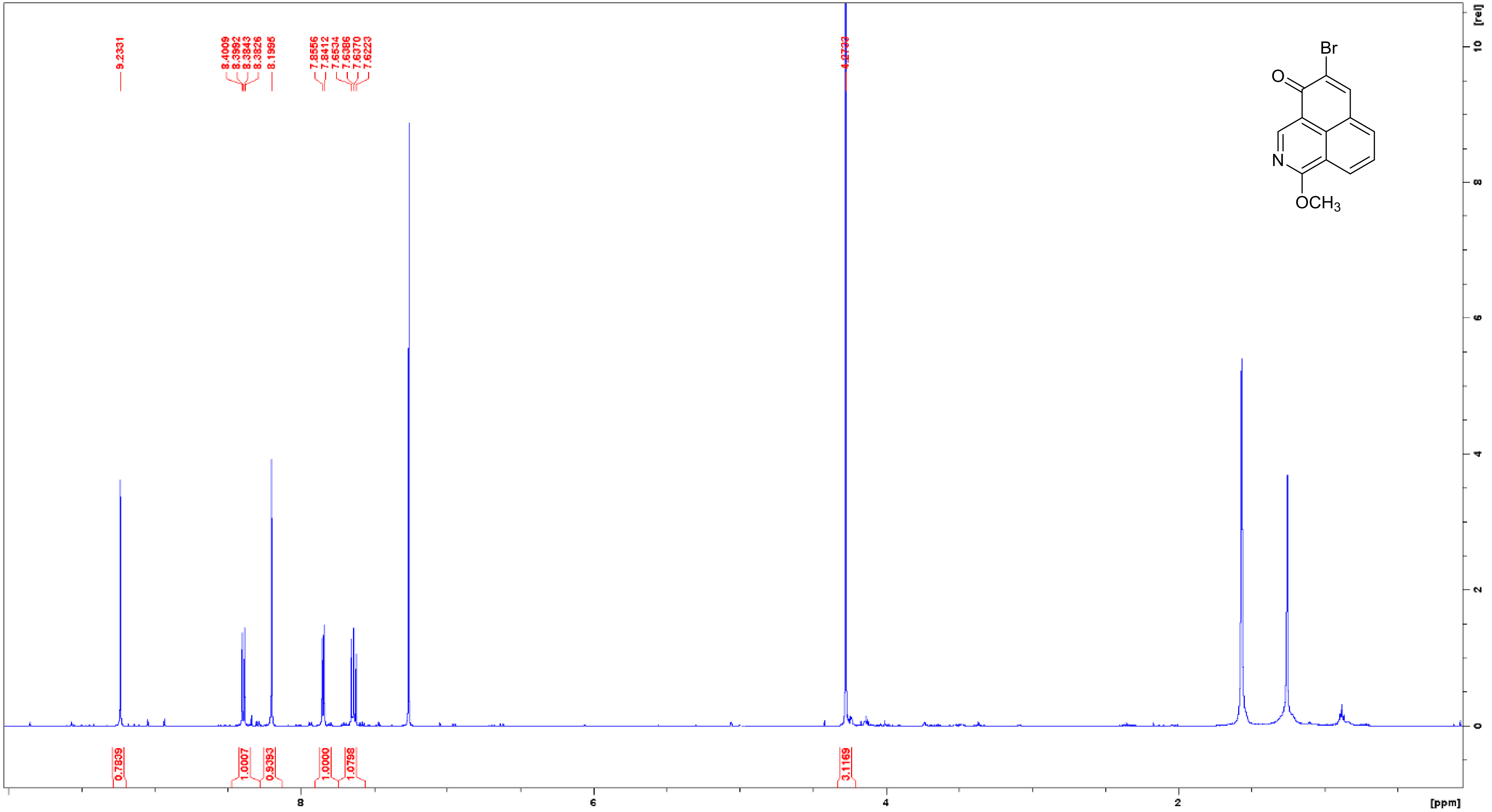

Expanded ^{1}H NMR spectrum (500 MHz, $CDCl_3$) of compound **39**:

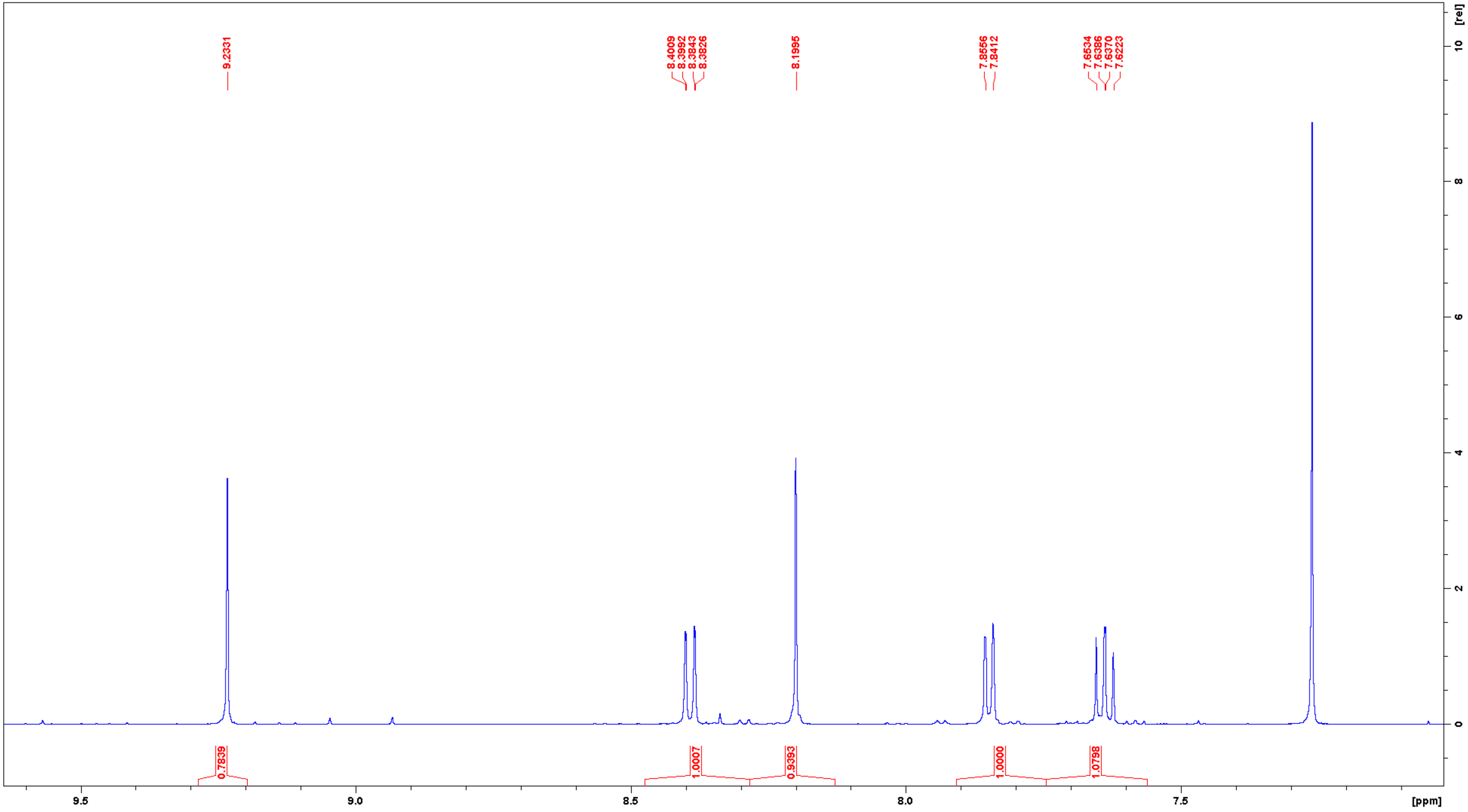

^{13}C NMR spectrum (125 MHz, $CDCl_3$) of compound **39**:

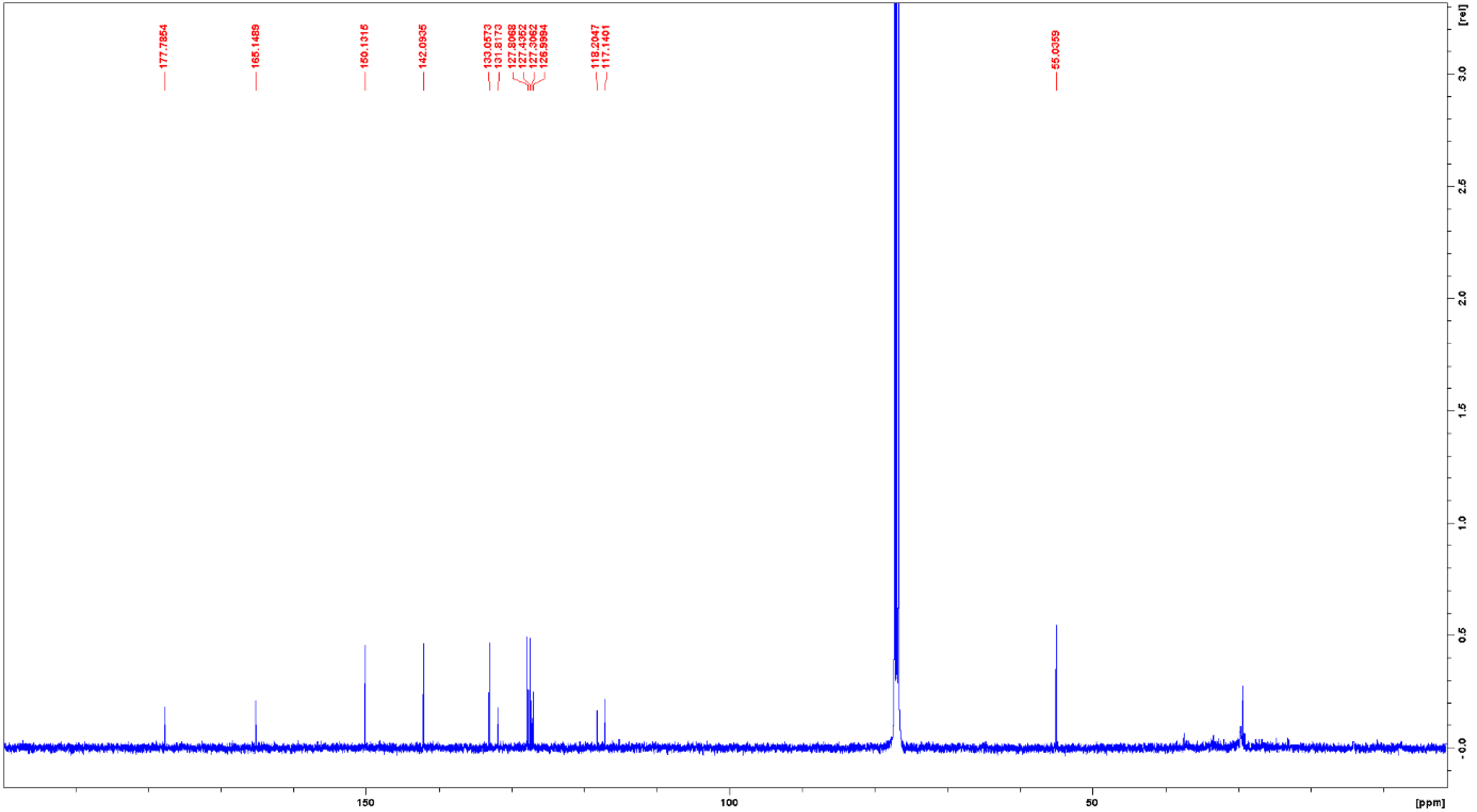

Expanded ^{13}C NMR spectrum (125 MHz, $CDCl_3$) of compound **39**:

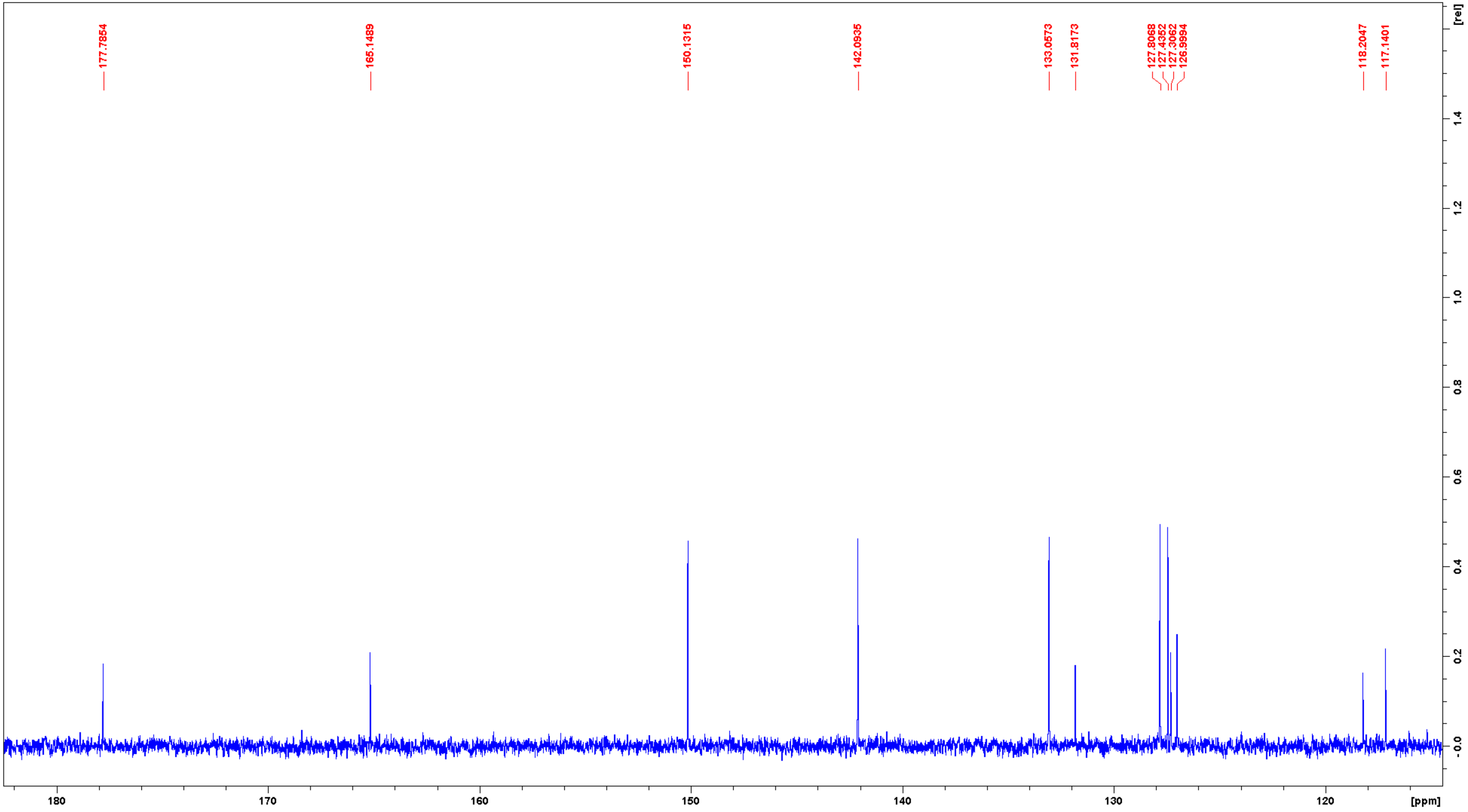

COSY spectrum (500 MHz, $CDCl_3$) of compound **39**:

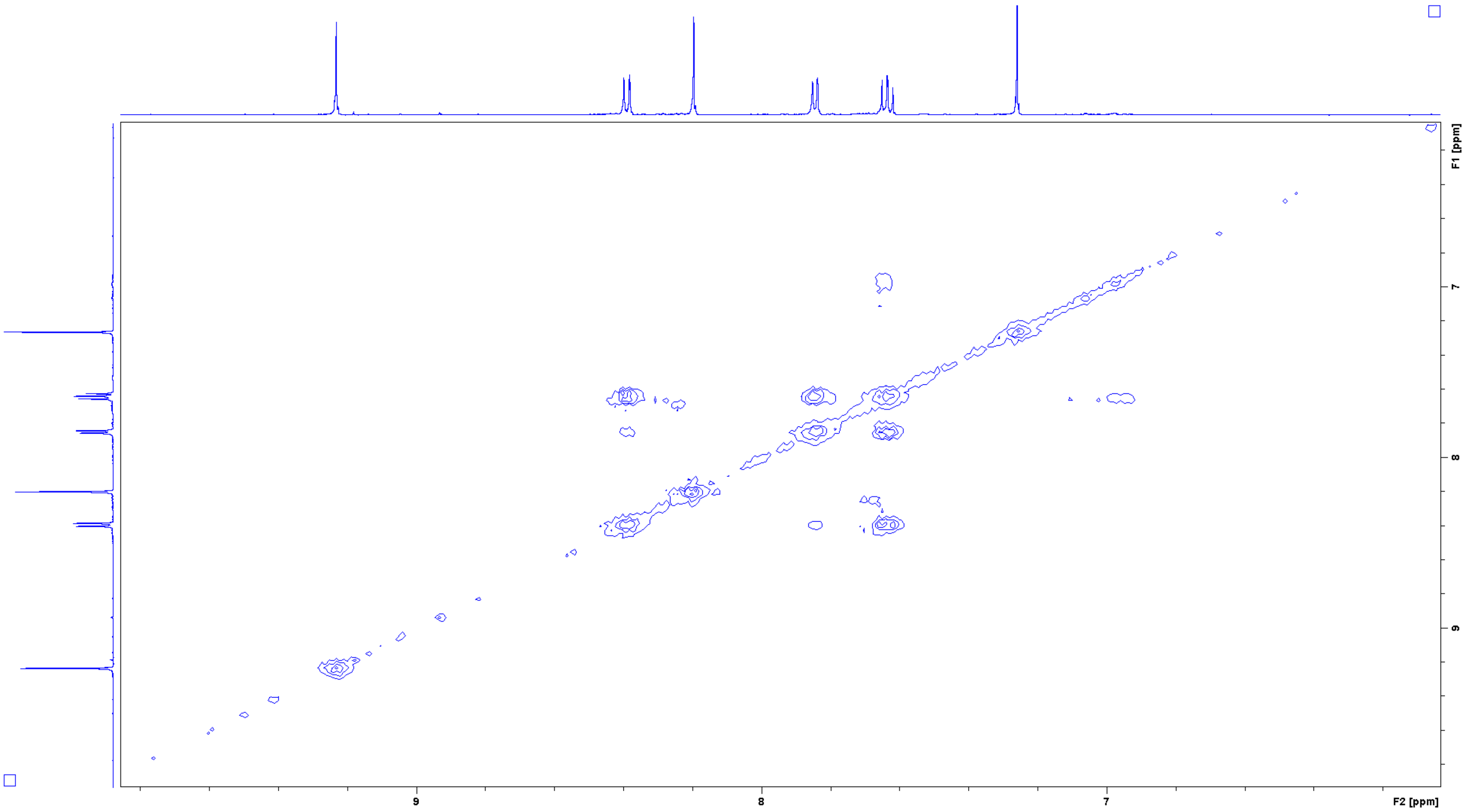

Expanded COSY spectrum (500 MHz, $CDCl_3$) of compound **39**:

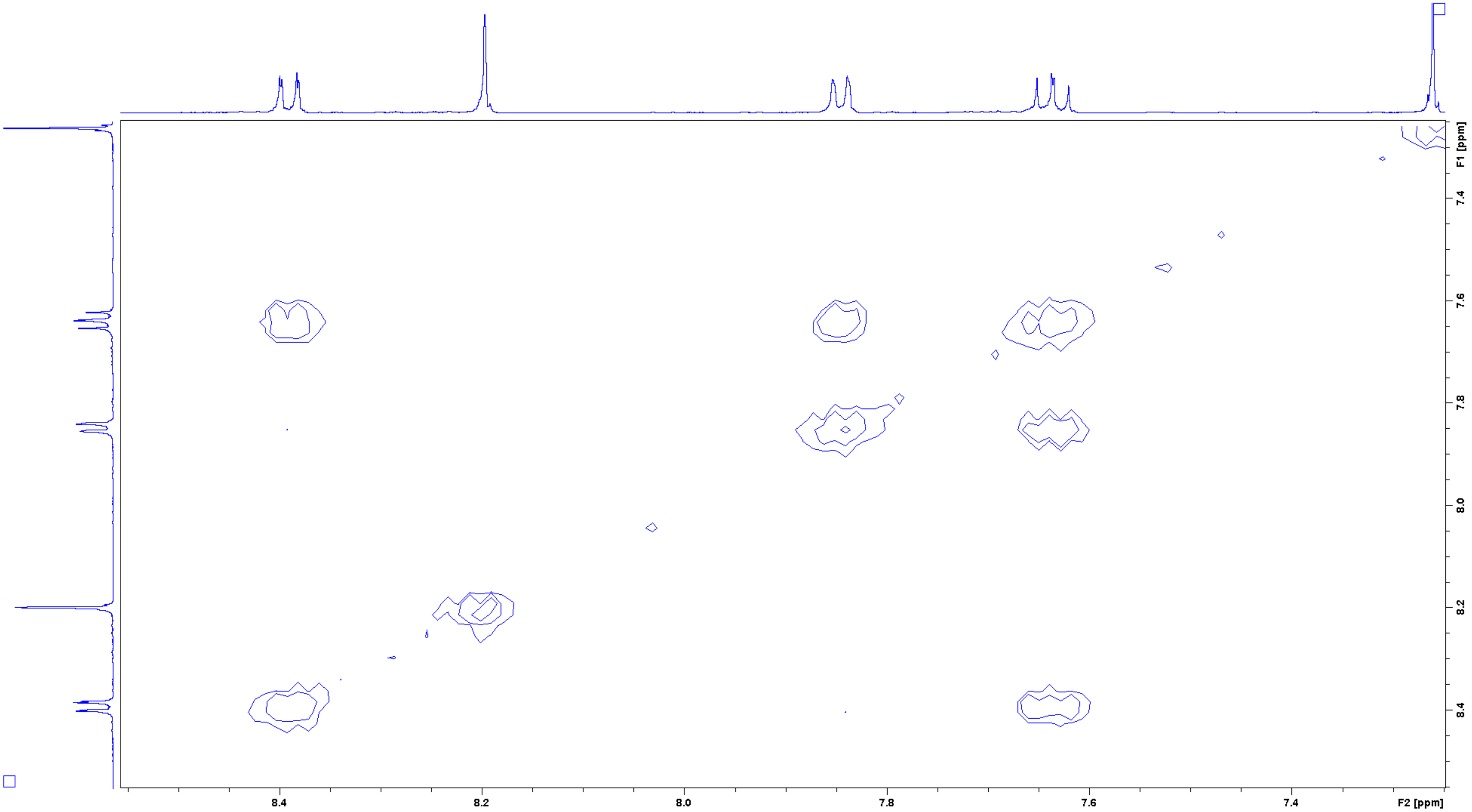

HSQCed spectrum (500 MHz, $CDCl_3$) of compound **39**:

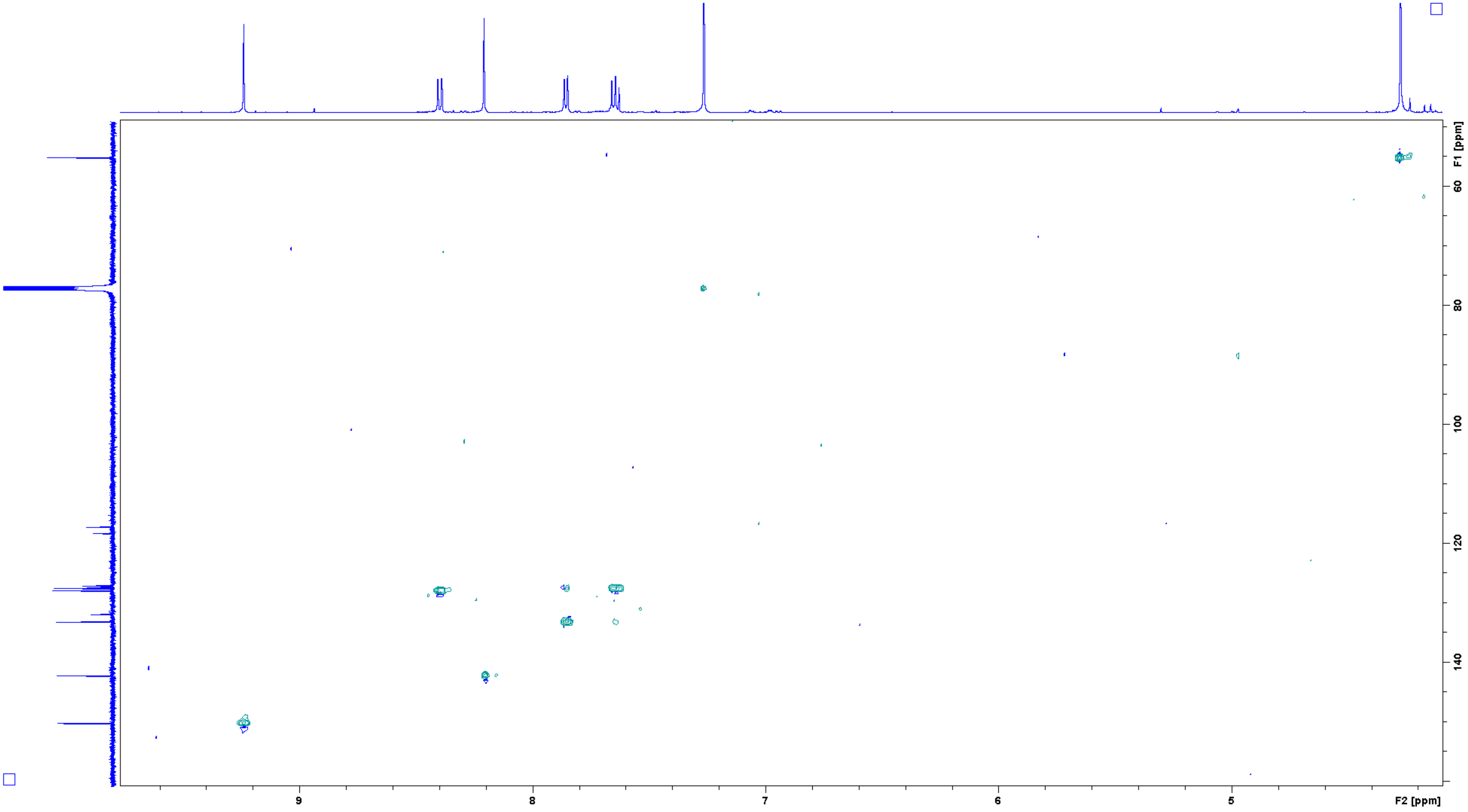

Expanded HSQCed spectrum (500 MHz, $CDCl_3$) of compound **39**:

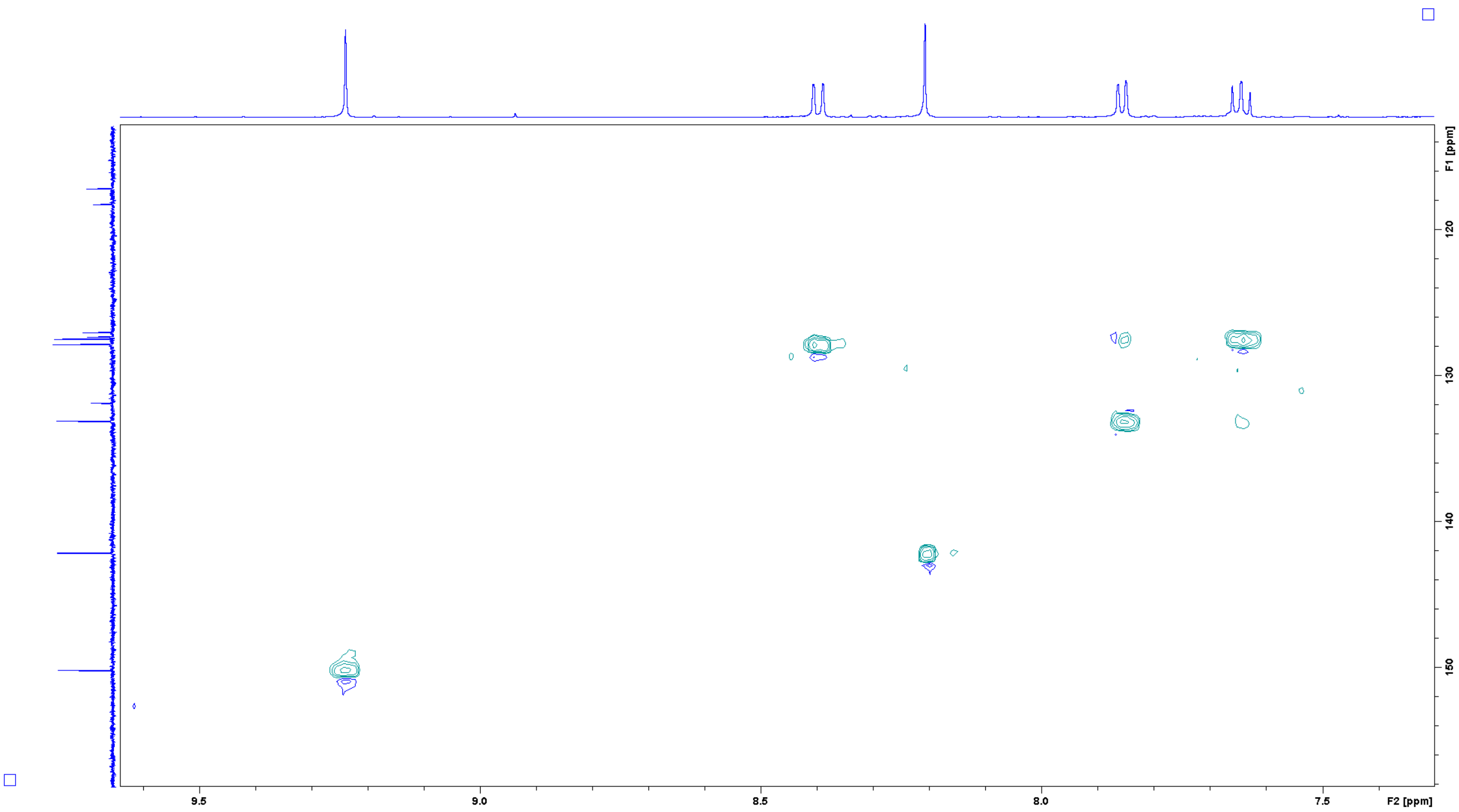

Expanded HSQCed spectrum (500 MHz, $CDCl_3$) of compound **39**:

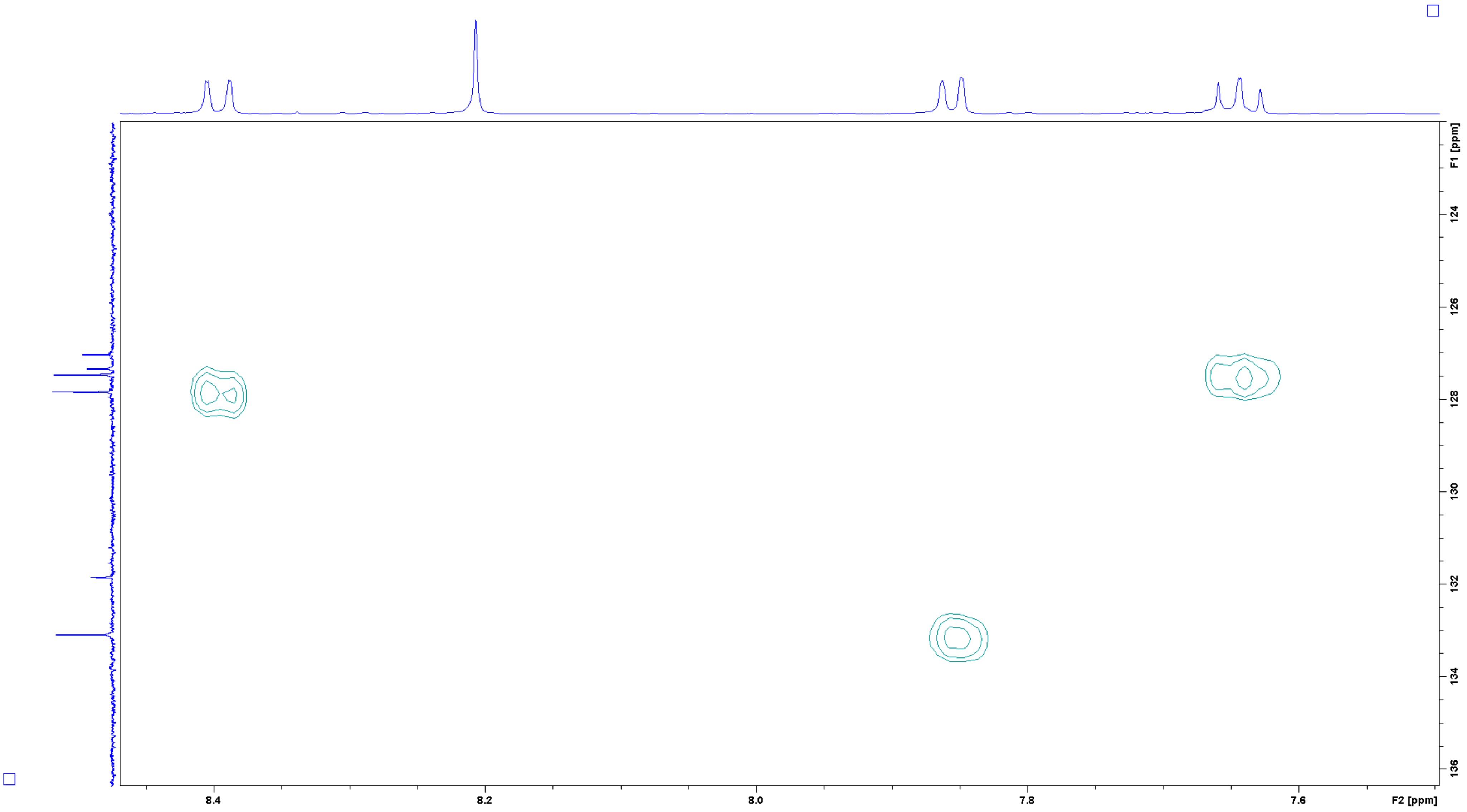

HMBC spectrum (500 MHz, $CDCl_3$) of compound **39**:

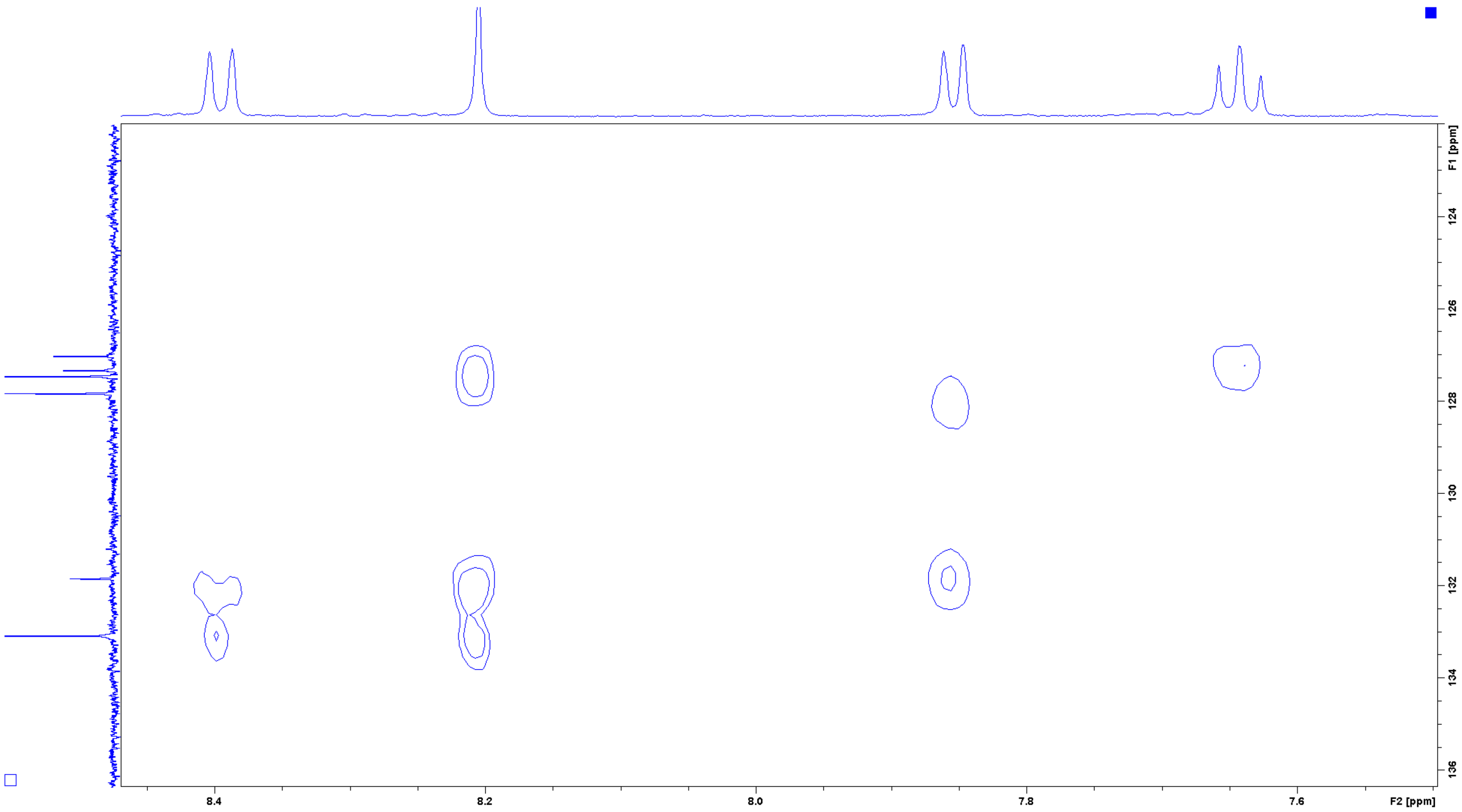

In the reaction of 1,3,5-triazines with naphthalene bis-substituted derivatives, an intermediate (**29a**/**32a)** is formed, which undergoes ring opening in a manner similar to azanaphthalenes [29]. Subsequent transformations generate the corresponding cation (**29b**/**32c)**, and final hydrolysis affords the 4*H*-benzo[*de*]isoquinolin-4-one (**36**/**37**) (Scheme S1).

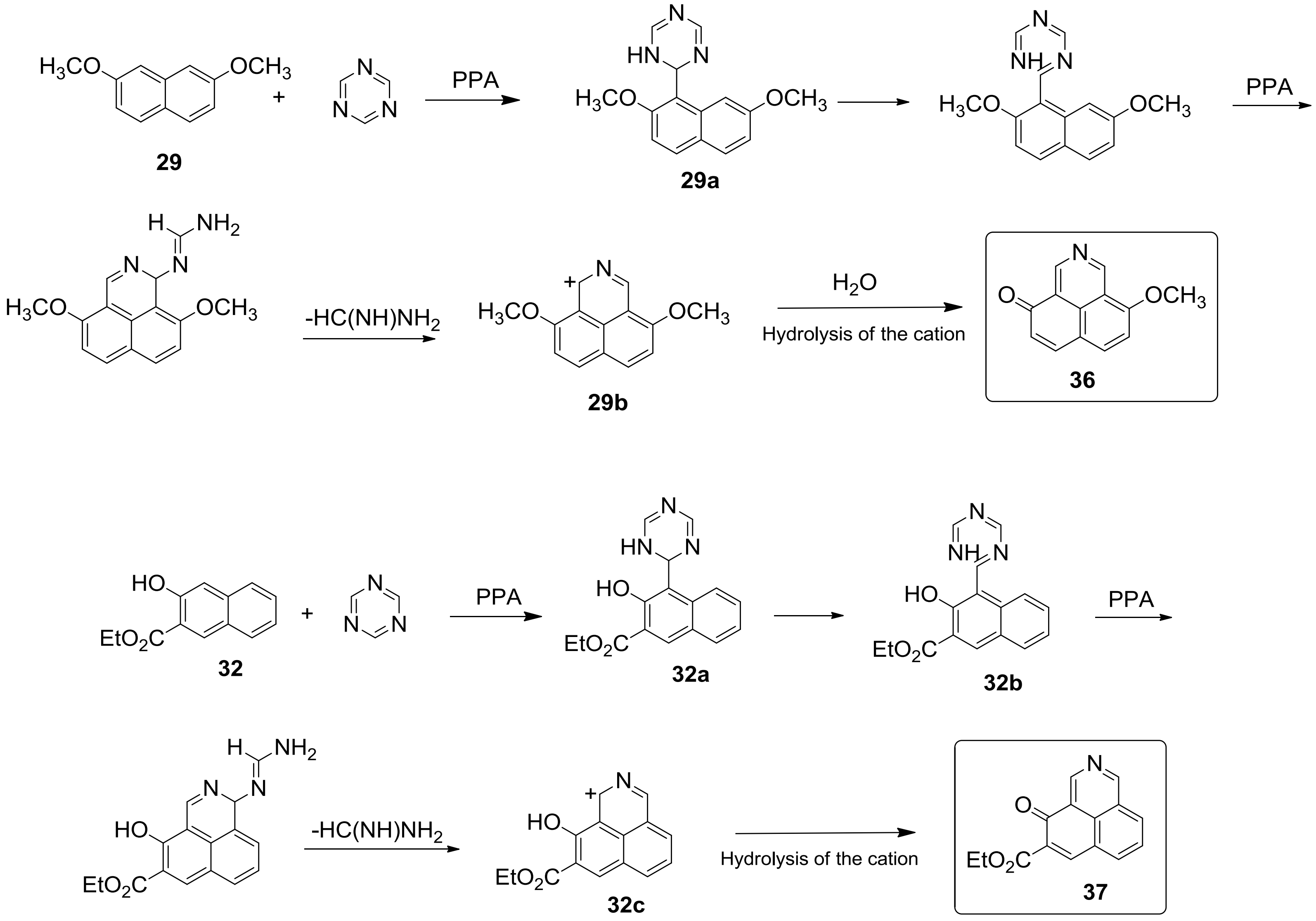

Scheme S1. Proposed mechanism for the formation of compounds **36** and **37**

29. Aksenov, V.A.; Aksenov, N.A.; Lyakhovnenko, A.S.; Aksenova, I.V. Regioselectivity Change in the Reaction of Naphthalene and 2-Naphthyl Ethers with 1,3,5-Triazines Depending on Reagent Quantities. *Synthesis* **2009**, *20*, 3439–3442. https://doi.org/10.1055/s-0029-1216973.
