# Supplementary material for: Synthesis of Substituted 1H-Phenalen-1-ones and Nitrogen-Containing Heterocyclic Analogues as Potential Anti-Plasmodial Agents
Source: Molecules. 2025 Dec 5;30(24):4667. doi: 10.3390/molecules30244667 (PMC12735704; doi:10.3390/molecules30244667)
Supplement: Supplementary file 1 [file molecules-30-04667-s001.zip › Supplementary Material-S2.pdf]

Supplementary Materials

Synthesis of Substituted 1*H*-Phenalen-1-ones and Nitrogen-Containing Heterocyclic Analogues as Potential Anti-plasmodial Agents

Teresa Abad-Grillo, [1,*] Grant McNaughton-Smith, [2,*] Mónica Blanco Freijó, [1] David Gutiérrez[3] and Ninoska Flores[3]

[1] Departamento de Química Orgánica, Universidad de La Laguna, Avenida Astrofísico Francisco Sánchez, 2, 38206 La Laguna, Tenerife, Spain; tereabad@ull.edu.es

[2] Centro Atlántico del Medicamento S.A (CEAMED S.A.), PCTT, 38200 La Laguna, Tenerife, Spain; gmcsmith@ceamedsa.com

[3] Instituto de Investigaciones Fármaco Bioquímicas, Facultad de Ciencias Farmacéuticas y Bioquímicas, Universidad Mayor de San Andrés, Avenida Saavedra 2224, Miraflores, La Paz, Bolivia

* Correspondence: tereabad@ull.edu.es; gmcsmith@ceamedsa.com

Contents:
High Resolution Mass Spectra

HRMS of compound **4**:

Multiple Mass Analysis: 3 mass(es) processed

Tolerance = 5.0 PPM / DBE: min = -20.0, max = 1000.0

Element prediction: Off

Number of isotope peaks used for i-FIT = 2

Monoisotopic Mass, Even Electron Ions

2154 formula(e) evaluated with 5 results within limits (up to 50 closest results for each mass)

Elements Used:

C: 0-52 H: 0-120 N: 0-4 O: 0-12 Na: 0-1

H-2123-Monica (B2 13) 4 (0.156)

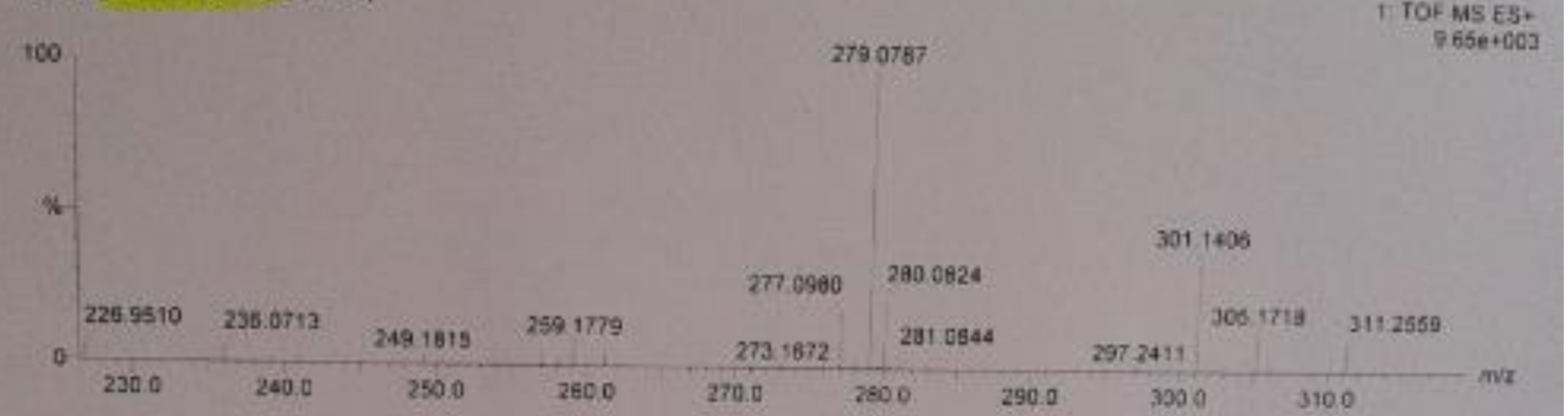

Minimum:	20.00				-20.0		
Maximum:	100.00		100.0	5.0	1000.0		
Mass	RA	Calc. Mass	mDa	PPM	DBE	i-FIT	Formula
279.0787	100.00	279.0786	0.1	0.4	13.5	7.2	C19 H12 O Na
		279.0788	-0.1	-0.4	-0.5	576.0	C4 H15 N4 O10
280.0824	23.38	280.0821	0.3	1.1	7.5	17.1	C12 H14 N O6
301.1406	40.68	301.1400	0.6	2.0	4.5	4.5	C13 H21 N2 O6
		301.1416	-1.0	-3.3	5.5	0.3	C16 H22 O4 Na

HRMS of compound **5**:

Elemental Composition Report

Page 1

Multiple Mass Analysis: 48 mass(es) processed - displaying only valid results
Tolerance = 50.0 PPM / DBE: min = -1.5, max = 50.0
Selected filters: None

Monoisotopic Mass, Odd and Even Electron Ions
146 formula(e) evaluated with 29 results within limits (all results (up to 1000) for each mass)
Elements Used:
C: 1-20 H: 2-11 N: 0-1 O: 0-1
Monica
05-May-14-BAFAMMA 190 (4.516)

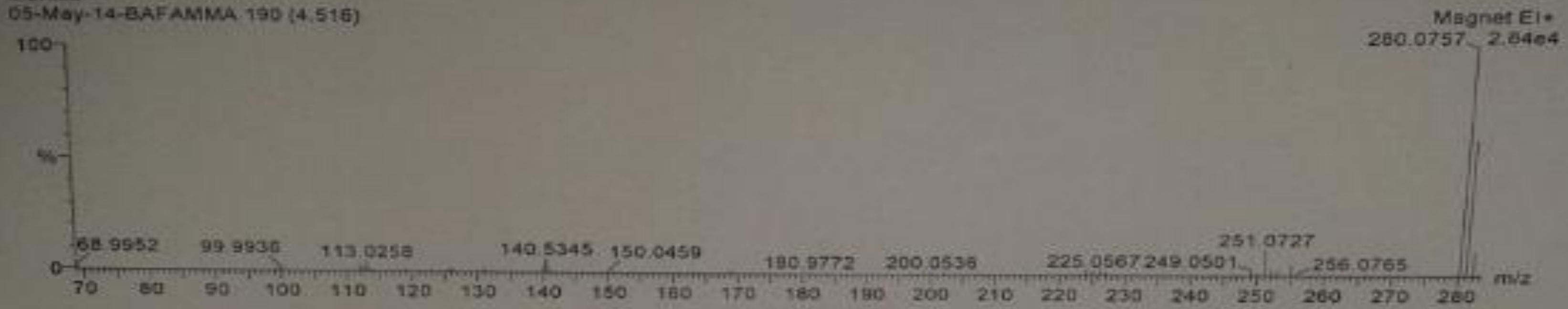

Minimum:	0.10				-1.5		
Maximum:	100.00		5.0	50.0	50.0		
Mass	RA	Calc. Mass	mDa	PPM	DBE	i-FIT	Formula
281.0834	59.95	281.0841	-0.7	-2.5	16.0	128.4	C20 H11 N O
280.0757	100.00	280.0762	-0.5	-1.8	16.5	3792.8	C20 H10 N O
256.0765	1.01	256.0762	0.3	1.2	14.5	5546153.0	C18 H10 N O
255.0805	4.89	255.0810	-0.5	-2.0	14.5	2773318.8	C19 H11 O
		255.0684	12.1	47.4	15.0	2773302.8	C18 H9 N O
253.0838	2.16	253.0891	-5.3	-20.9	15.0	2774013.5	C19 H11 N
252.0763	3.07	252.0813	-5.0	-19.8	15.5	2773310.5	C19 H10 N
251.0727	11.69	251.0735	-0.8	-3.2	16.0	246.8	C19 H9 N
250.0594	1.27	250.0657	-6.3	-25.2	16.5	2007.4	C19 H8 N
249.0501	2.15	249.0578	-7.7	-30.9	17.0	1726.0	C19 H7 N
227.0563	1.29	227.0497	6.6	29.1	14.5	5546191.5	C17 H7 O
226.0761	1.43	226.0783	-2.2	-9.7	14.0	2773158.8	C18 H10
		226.0657	10.4	46.0	14.5	2773146.8	C17 H8 N
226.0615	1.96	226.0657	-4.2	-18.6	14.5	2773146.8	C17 H8 N
225.0567	2.17	225.0578	-1.1	-4.9	15.0	337.4	C17 H7 N
224.0480	2.13	224.0500	-2.0	-8.9	15.5	469.0	C17 H6 N
223.0377	0.59	223.0422	-4.5	-20.2	16.0	577.2	C17 H5 N
202.0770	0.52	202.0783	-1.3	-6.4	12.0	5546082.5	C16 H10
202.0639	0.68	202.0657	-1.8	-8.9	12.5	5546103.0	C15 H8 N
200.0536	1.09	200.0500	3.6	18.0	13.5	2773245.0	C15 H6 N
		200.0626	-9.0	-45.0	13.0	2773247.5	C16 H8
199.0376	0.63	199.0422	-4.6	-23.1	14.0	2773140.0	C15 H5 N
163.0551	0.52	163.0548	0.3	1.8	10.5	5546076.0	C13 H7
151.0529	0.68	151.0548	-1.9	-12.6	9.5	5546095.0	C12 H7
150.0459	0.85	150.0470	-1.1	-7.3	10.0	2773079.5	C12 H6
126.0482	0.88	126.0470	1.2	9.5	8.0	5546113.0	C10 H6
126.0390	1.28	126.0344	4.6	36.5	8.5	5546164.0	C9 H4 N
113.0258	4.31	113.0265	-0.7	-6.2	8.0	5546575.0	C8 H3 N
112.0194	2.63	112.0187	0.7	6.2	8.5	2773561.0	C8 H2 N

HRMS of compound **6**:

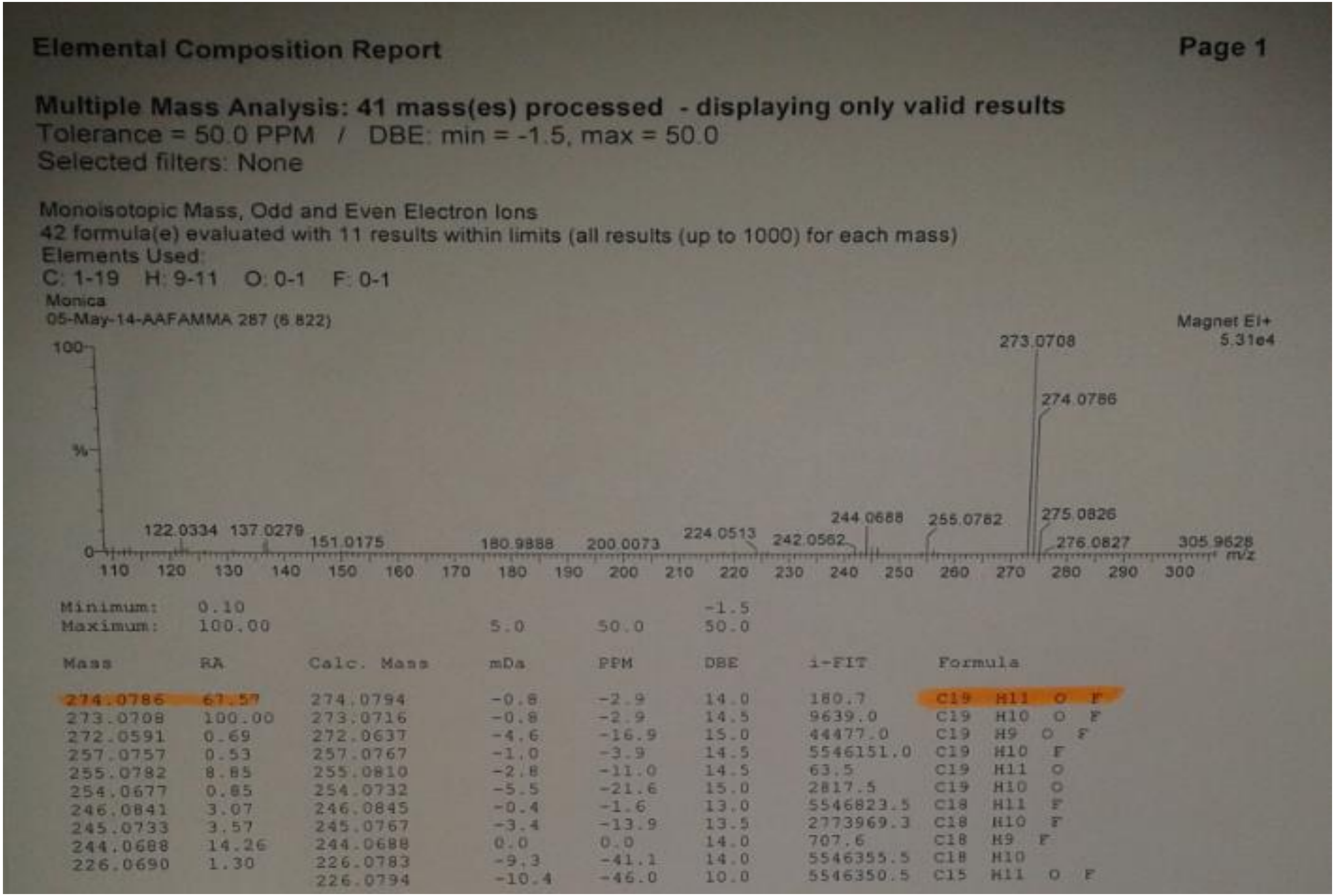

Elemental Composition Report Page 1

Multiple Mass Analysis: 41 mass(es) processed - displaying only valid results
Tolerance = 50.0 PPM / DBE: min = -1.5, max = 50.0
Selected filters: None

Monoisotopic Mass, Odd and Even Electron Ions
42 formula(e) evaluated with 11 results within limits (all results (up to 1000) for each mass)
Elements Used:
C: 1-19 H: 9-11 O: 0-1 F: 0-1

Minimum:	0.10				-1.5		
Maximum:	100.00		5.0	50.0	50.0		

Mass	RA	Calc. Mass	mDa	PPM	DBE	i-FIT	Formula
274.0786	67.57	274.0794	-0.8	-2.9	14.0	180.7	C19 H11 O F
273.0708	100.00	273.0716	-0.8	-2.9	14.5	9639.0	C19 H10 O F
272.0591	0.69	272.0637	-4.6	-16.9	15.0	44477.0	C19 H9 O F
257.0757	0.53	257.0767	-1.0	-3.9	14.5	5546151.0	C19 H10 F
255.0782	8.85	255.0810	-2.8	-11.0	14.5	63.5	C19 H11 O
254.0677	0.85	254.0732	-5.5	-21.6	15.0	2817.5	C19 H10 O
246.0841	3.07	246.0845	-0.4	-1.6	13.0	5546823.5	C18 H11 F
245.0733	3.57	245.0767	-3.4	-13.9	13.5	2773969.3	C18 H10 F
244.0688	14.26	244.0688	0.0	0.0	14.0	707.6	C18 H9 F
226.0690	1.30	226.0783	-9.3	-41.1	14.0	5546355.5	C18 H10
		226.0794	-10.4	-46.0	10.0	5546350.5	C15 H11 O F

HRMS of compound **7**:

Elemental Composition Report

Electrospray

Page 1

Multiple Mass Analysis: 4 mass(es) processed
Tolerance = 5.0 PPM / DBE: min = -20.0, max = 1000.0
Element prediction: Off
Number of isotope peaks used for i-FIT = 2

Monoisotopic Mass, Even Electron Ions
2194 formula(e) evaluated with 4 results within limits (up to 50 closest results for each mass)
Elements Used:
C: 0-52 H: 0-120 N: 0-1 O: 0-12 Na: 0-1 S: 0-1
H-2116- Monica (B2-14) 3 (0.121)

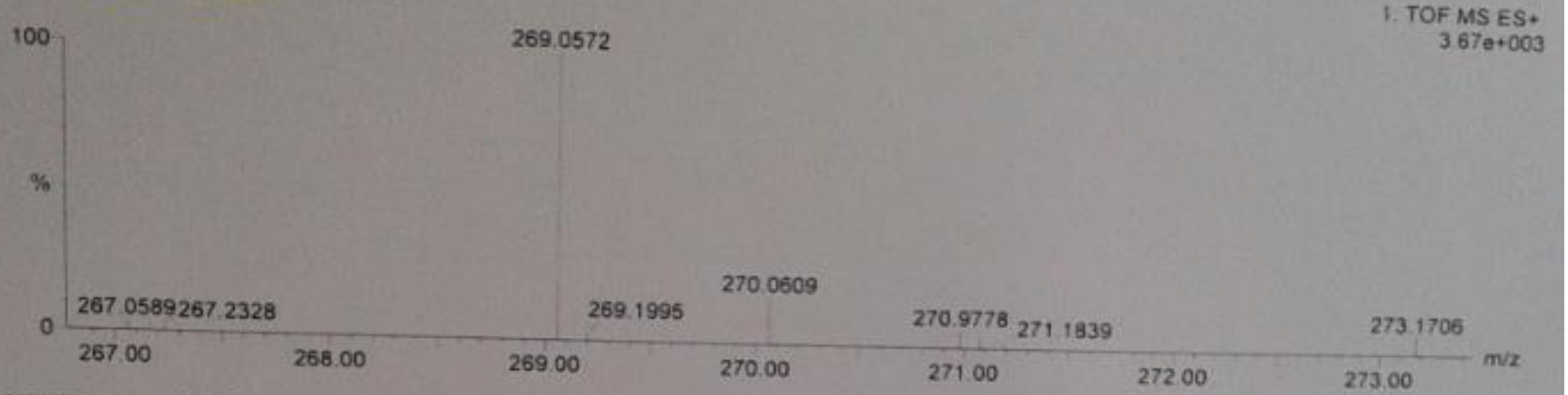

Minimum:	5.00				-20.0		
Maximum:	100.00		100.0	5.0	1000.0		
Mass	RA	Calc. Mass	mDa	PPM	DBE	i-FIT	Formula
269.0572	100.00	269.0578	-0.6	-2.2	12.5	4.8	C17 H10 O2 Na
270.0609	16.85	270.0614	-0.5	-1.9	6.5	11.5	C11 H12 N O7
270.9778	6.07	---					
273.1706	6.65	273.1702	0.4	1.5	2.5	n/a	C14 H25 O5
		273.1712	-0.6	-2.2	-5.5	n/a	C9 H30 O5 Na S

HRMS of compound **8**:

Elemental Composition Report

Page 1

Multiple Mass Analysis: 86 mass(es) processed - displaying only valid results
Tolerance = 50.0 PPM / DBE: min = -1.5, max = 50.0
Selected filters: None

Monoisotopic Mass, Odd and Even Electron Ions
3268 formula(e) evaluated with 11 results within limits (all results (up to 1000) for each mass)
Elements Used:
C: 1-14 H: 1-7 O: 0-4 F: 0-3 S: 0-1
26Abr13-B 396 (9.413) Sm (Mn, 50x6.00)
Magnet EI+

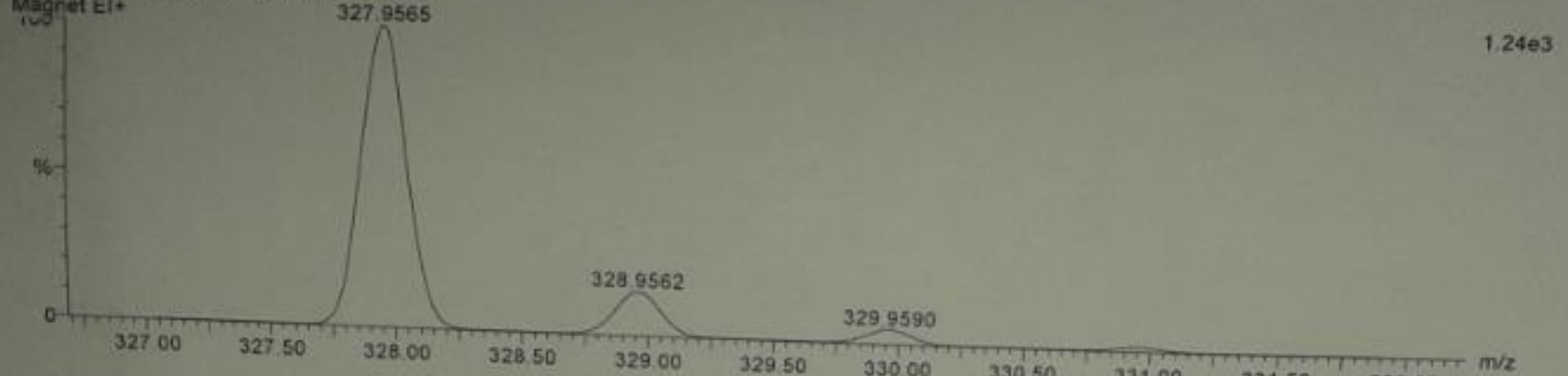

Mass	RA	Calc. Mass	mDa	PPM	DBE	Formula
Minimum:	30.00				-1.5	
Maximum:	100.00		5.0	50.0	50.0	
328.0170	77.59	328.0017	15.3	46.6	10.0	C14 H7 O4 F3 S
328.0139	79.57	328.0017	12.2	37.2	10.0	C14 H7 O4 F3 S
328.0109	81.51	328.0017	9.2	28.0	10.0	C14 H7 O4 F3 S
328.0079	83.36	328.0017	6.2	18.9	10.0	C14 H7 O4 F3 S
328.0049	85.13	328.0017	3.2	9.8	10.0	C14 H7 O4 F3 S
328.0019	86.82	328.0017	0.2	0.6	10.0	C14 H7 O4 F3 S
327.9988	88.42	328.0017	-2.9	-8.8	10.0	C14 H7 O4 F3 S
327.9958	89.95	328.0017	-5.9	-18.0	10.0	C14 H7 O4 F3 S
327.9928	91.40	328.0017	-8.9	-27.1	10.0	C14 H7 O4 F3 S
327.9897	92.77	328.0017	-12.0	-36.6	10.0	C14 H7 O4 F3 S
327.9868	94.05	328.0017	-14.9	-45.4	10.0	C14 H7 O4 F3 S

Compound **10**:

Monoisotopic Mass, Odd and Even Electron Ions
129 formula(e) evaluated with 27 results within limits (all results (up to 1000) for each mass)
Elements Used:
C: 1-20 H: 2-11 N: 0-1 O: 0-1

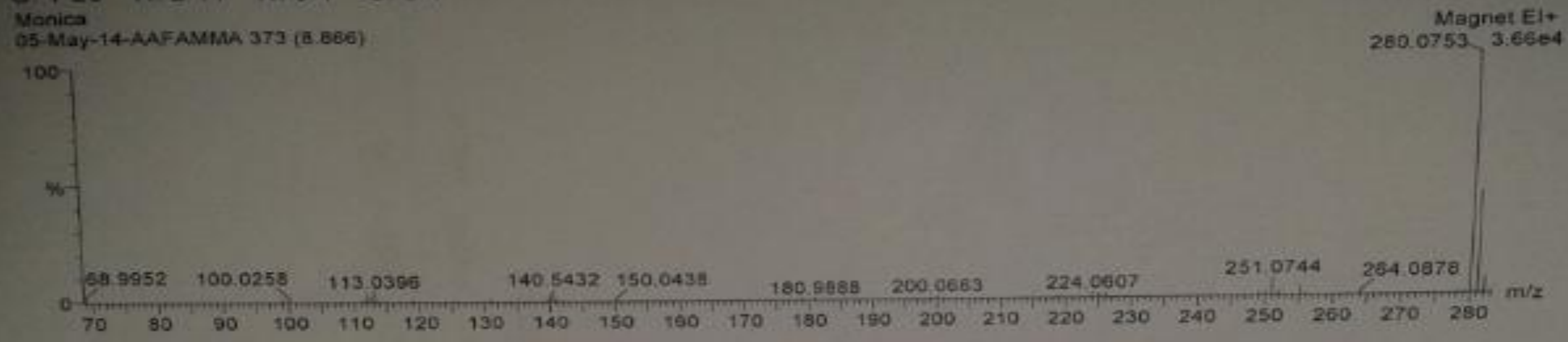

Mass	RA	Calc. Mass	mDa	PPM	DBE	i-FIT	Formula
Minimum:	0.10				-1.5		
Maximum:	100.00		5.0	50.0	50.0		
281.0833	42.85	281.0841	-0.8	-2.8	16.0	174.3	C20 H11 N O
280.0753	100.00	280.0762	-0.9	-3.2	16.5	1945.2	C20 H10 N O
279.0606	0.83	279.0684	-7.8	-28.0	17.0	26090.7	C20 H9 N O
278.0624	0.84	278.0606	1.8	6.5	17.5	18449.4	C20 H8 N O
278.0471	0.62	278.0606	-13.5	-48.6	17.5	18449.4	C20 H8 N O
264.0878	1.26	264.0813	6.5	24.6	16.5	5546243.0	C20 H10 N
264.0732	1.24	264.0813	-8.1	-30.7	16.5	5546243.0	C20 H10 N
262.0575	0.52	262.0657	-8.2	-31.3	17.5	2773320.8	C20 H8 N
256.0786	0.60	256.0762	2.4	9.4	14.5	5546119.0	C18 H10 N O
255.0783	4.08	255.0810	-2.7	-10.6	14.5	2773339.0	C19 H11 O
		255.0684	9.9	38.8	15.0	2773316.5	C18 H9 N O
252.0776	1.45	252.0813	-3.7	-14.7	15.5	5546275.5	C19 H10 N
251.0744	7.42	251.0735	0.9	3.6	16.0	2773827.5	C19 H9 N
		251.0861	-11.7	-46.6	15.5	2773900.0	C20 H11
250.0637	1.15	250.0657	-2.0	-8.0	16.5	1516.3	C19 H8 N
249.0548	1.65	249.0578	-3.0	-12.0	17.0	1446.7	C19 H7 N
226.0778	1.37	226.0783	-0.5	-2.2	14.0	5546259.0	C18 H10
225.0587	1.62	225.0578	0.9	4.0	15.0	2773213.5	C17 H7 N
224.0607	2.05	224.0626	-1.9	-8.5	15.0	393.7	C18 H8
		224.0500	10.7	47.8	15.5	413.9	C17 H6 N
201.0708	0.52	201.0704	0.4	2.0	12.5	5546101.5	C16 H9
200.0663	1.16	200.0626	3.7	18.5	13.0	2773061.5	C16 H8
199.0521	0.51	199.0548	-2.7	-13.6	13.5	272.3	C16 H7
		199.0422	9.9	49.7	14.0	291.4	C15 H5 N
150.0438	0.62	150.0470	-3.2	-21.3	10.0	5546111.0	C12 H6
113.0396	4.47	113.0391	0.5	4.4	7.5	5546787.0	C9 H5
112.0325	3.75	112.0313	1.2	10.7	8.0	2773717.0	C9 H4

Compound **11**:

Elemental Composition Report

Multiple Mass Analysis: 64 mass(es) processed - displaying only valid results
Tolerance = 50.0 PPM / DBE: min = -1.5, max = 50.0
Selected filters: None

Monoisotopic Mass, Odd and Even Electron Ions
183 formula(e) evaluated with 27 results within limits (all results (up to 1000) for each mass)
Elements Used:
C: 1-19 H: 2-11 O: 0-1 F: 0-1

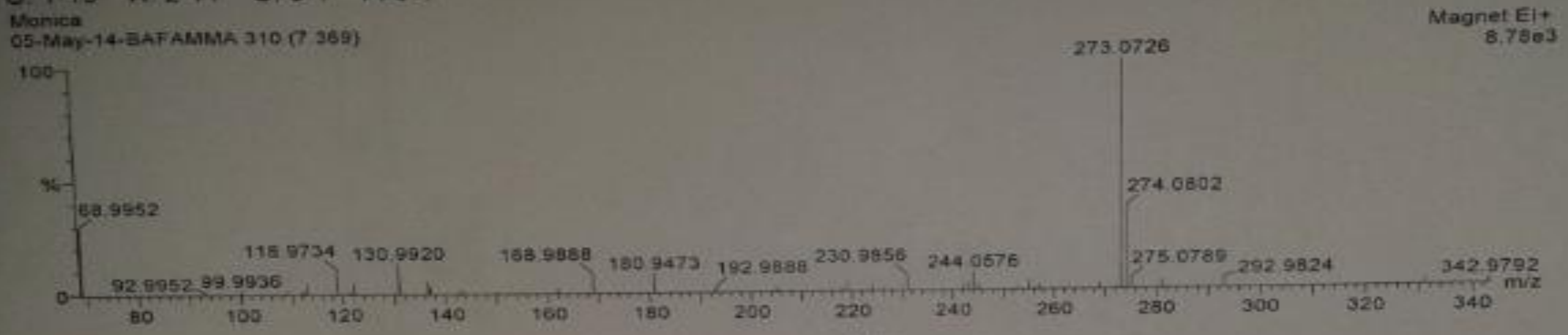

Mass	RA	Calc. Mass	mDa	PPM	DBE	i-FIT	Formula
Minimum:	0.10				-1.5		
Maximum:	100.00		5.0	50.0	50.0		
274.0802	36.24	274.0794	0.8	2.9	14.0	2774027.5	C19 H11 O F
273.0726	100.00	273.0716	1.0	3.7	14.5	282.3	C19 H10 O F
257.0807	1.71	257.0767	4.0	15.6	14.5	5546086.5	C19 H10 F
256.0688	0.72	256.0688	0.0	0.0	15.0	2773074.0	C19 H9 F
255.0679	3.78	255.0610	6.9	27.1	15.5	70.2	C19 H8 F
253.0602	0.67	253.0653	-5.1	-20.2	15.5	2773196.5	C19 H9 O
245.0690	2.22	245.0767	-7.7	-31.4	13.5	5546108.0	C18 H10 F
245.0464	0.53	245.0403	6.1	24.9	14.5	5546036.5	C17 H6 O F
244.0576	8.28	244.0688	-11.2	-45.9	14.0	2773096.5	C18 H9 F
243.0595	1.32	243.0610	-1.5	-6.2	14.5	434.9	C18 H8 F
242.0526	2.65	242.0532	-0.6	-2.5	15.0	377.7	C18 H7 F
226.0853	1.01	226.0794	5.9	26.1	10.0	5546053.0	C15 H11 O F
		226.0783	7.0	31.0	14.0	5546056.0	C18 H10
224.0677	2.14	224.0637	4.0	17.9	11.0	45.4	C15 H9 O F
		224.0626	5.1	22.8	15.0	43.7	C18 H8
218.0637	1.21	218.0732	-9.5	-43.6	12.0	2773362.8	C16 H10 O
		218.0532	10.5	48.2	13.0	2773291.8	C16 H7 F
150.0519	0.51	150.0481	3.8	25.3	6.0	5546029.5	C9 H7 O F
		150.0470	4.9	32.7	10.0	5546032.0	C12 H6
137.0455	3.26	137.0403	5.2	37.9	5.5	5546118.5	C8 H6 O F
		137.0391	6.4	46.7	9.5	5546136.5	C11 H5
127.0243	0.98	127.0184	5.9	46.4	8.5	5546041.5	C9 H3 O
[illegible]	4.71	122.0157	-1.7	-13.9	10.0	5546190.5	C10 H2
[illegible]	[illegible]	[illegible]	[illegible]	[illegible]	[illegible]	[illegible]	[illegible]

HRMS of compound **13**:

Multiple Mass Analysis: 104 mass(es) processed - displaying only valid results
Tolerance = 50.0 PPM / DBE: min = -1.5, max = 50.0
Selected filters: None

Monoisotopic Mass, Odd and Even Electron Ions
285 formula(e) evaluated with 34 results within limits (all results (up to 1000) for each mass)
Elements Used:
C: 1-20 H: 2-14 O: 0-2
Monica
05-May-14-BAFAMMA (9.366) None

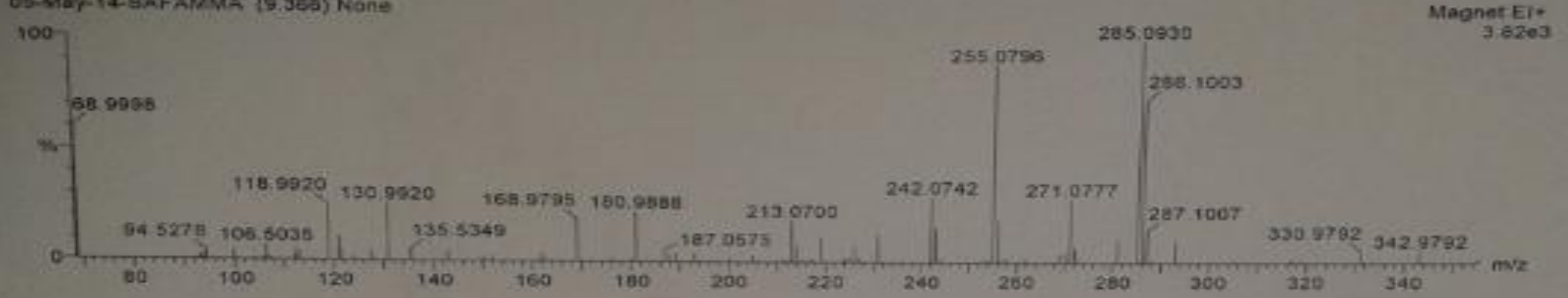

Minimum:	0.10				-1.5		
Maximum:	100.00		5.0	50.0	50.0		

Mass	RA	Calc. Mass	mDa	PPM	DBE	i-FIT	Formula
286.1003	73.59	286.0994	0.9	3.1	14.0	27.5	C20 H14 O2
285.0930	100.00	285.0916	1.4	4.9	14.5	797.9	C20 H13 O2
273.0862	0.91	273.0916	-5.4	-19.8	13.5	5546033.0	C19 H13 O2
272.0738	6.42	272.0837	-9.9	-36.4	14.0	2773027.0	C19 H12 O2
271.0777	28.90	271.0759	1.8	6.6	14.5	1.1	C19 H11 O2
270.0604	4.14	270.0681	-7.7	-28.5	15.0	634.4	C19 H10 O2
256.0824	19.33	256.0888	-6.4	-25.0	14.0	74.8	C19 H12 O
255.0796	89.68	255.0810	-1.4	-5.5	14.5	7.4	C19 H11 O
253.0659	0.71	253.0653	0.6	2.4	15.5	1726.5	C19 H9 O
244.0885	2.12	244.0888	-0.3	-1.2	13.0	5546052.5	C18 H12 O
243.0840	15.80	243.0810	3.0	12.3	13.5	2773069.5	C18 H11 O
242.0742	29.89	242.0732	1.0	4.1	14.0	126.3	C18 H10 O
227.0870	1.21	227.0861	0.9	4.0	13.5	5546037.5	C18 H11
226.0765	6.84	226.0783	-1.4	-6.2	14.0	2773023.0	C18 H10
225.0713	1.07	225.0704	0.9	4.0	14.5	156.2	C18 H9
224.0681	1.47	224.0626	5.5	24.5	15.0	162.0	C18 H8
215.0832	1.70	215.0861	-2.9	-13.5	12.5	2773106.5	C17 H11
214.0742	7.28	214.0783	-4.1	-19.2	13.0	2773020.8	C17 H10
213.0700	18.78	213.0704	-0.4	-1.9	13.5	53.5	C17 H9
212.0626	0.74	212.0626	0.0	0.0	14.0	492.0	C17 H8
211.0562	1.62	211.0548	1.4	6.6	14.5	364.9	C17 H7
211.0445	0.77	211.0548	-10.3	-48.8	14.5	364.9	C17 H7
200.0674	1.78	200.0626	4.8	24.0	13.0	5546045.0	C16 H8
189.0651	3.38	189.0704	-5.3	-28.0	11.5	5546071.5	C15 H9
188.0586	3.19	188.0626	-4.0	-21.3	12.0	2773057.5	C15 H8
187.0575	5.71	187.0548	2.7	14.4	12.5	90.0	C15 H7
187.0455	3.47	187.0548	-9.3	-49.7	12.5	90.0	C15 H7
180.0613	1.51	180.0575	3.8	21.1	10.0	2773813.5	C13 H8 O
180.0613	1.51	180.0575	2.5	14.3	11.5	2773044.8	C14 H7
175.0573	0.70	175.0548	[illegible]	[illegible]	10.5	5546031.0	C13 H7

HRMS of compound **16**:

Elemental Composition Report

Multiple Mass Analysis: 56 mass(es) processed - displaying only valid results

Tolerance = 50.0 PPM / DBE: min = -1.5, max = 50.0

Selected filters: None

Monoisotopic Mass, Odd and Even Electron Ions

245 formula(e) evaluated with 38 results within limits (all results (up to 1000) for each mass)

Elements Used:

C: 1-21 H: 2-17 N: 0-1 O: 0-1

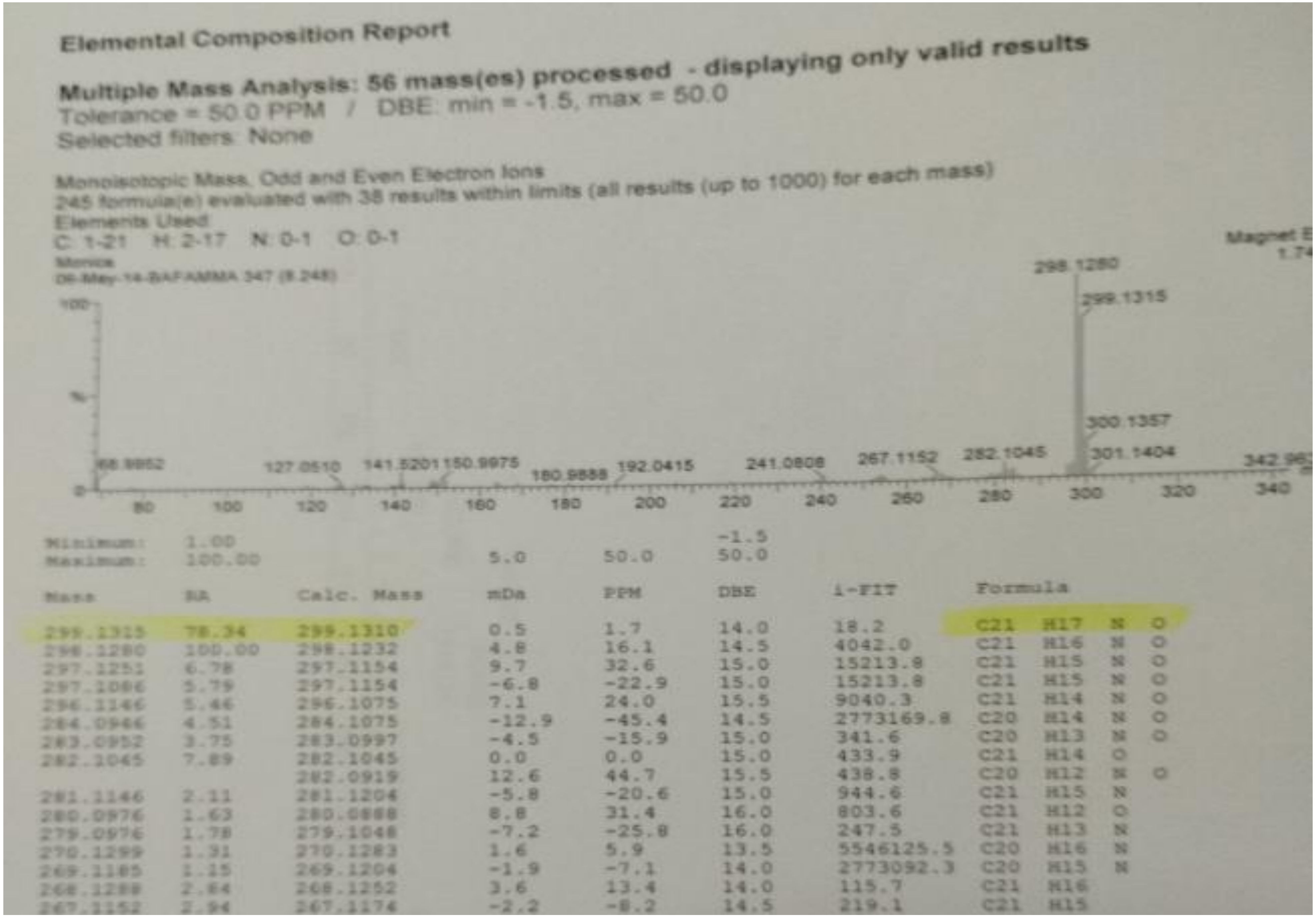

Minimum:	1.00				-1.5					
Maximum:	100.00		5.0	50.0	50.0					
Mass	RA	Calc. Mass	mDa	PPM	DBE	i-FIT	Formula			
299.1315	78.34	299.1310	0.5	1.7	14.0	18.2	C21	H17	N	O
298.1280	100.00	298.1232	4.8	16.1	14.5	4042.0	C21	H16	N	O
297.1251	6.78	297.1154	9.7	32.6	15.0	15213.8	C21	H15	N	O
297.1086	5.79	297.1154	-6.8	-22.9	15.0	15213.8	C21	H15	N	O
296.1146	5.46	296.1075	7.1	24.0	15.5	9040.3	C21	H14	N	O
284.0946	4.51	284.1075	-12.9	-45.4	14.5	2773169.8	C20	H14	N	O
283.0952	3.75	283.0997	-4.5	-15.9	15.0	341.6	C20	H13	N	O
282.1045	7.89	282.1045	0.0	0.0	15.0	433.9	C21	H14	O	
		282.0919	12.6	44.7	15.5	438.8	C20	H12	N	O
281.1146	2.11	281.1204	-5.8	-20.6	15.0	944.6	C21	H15	N	
280.0976	1.63	280.0888	8.8	31.4	16.0	803.6	C21	H12	O	
279.0976	1.78	279.1048	-7.2	-25.8	16.0	247.5	C21	H13	N	
270.1299	1.31	270.1283	1.6	5.9	13.5	5546125.5	C20	H16	N	
269.1185	1.15	269.1204	-1.9	-7.1	14.0	2773092.3	C20	H15	N	
268.1288	2.64	268.1252	3.6	13.4	14.0	115.7	C21	H16		
267.1152	2.94	267.1174	-2.2	-8.2	14.5	219.1	C21	H15		

HRMS of compound **17**:

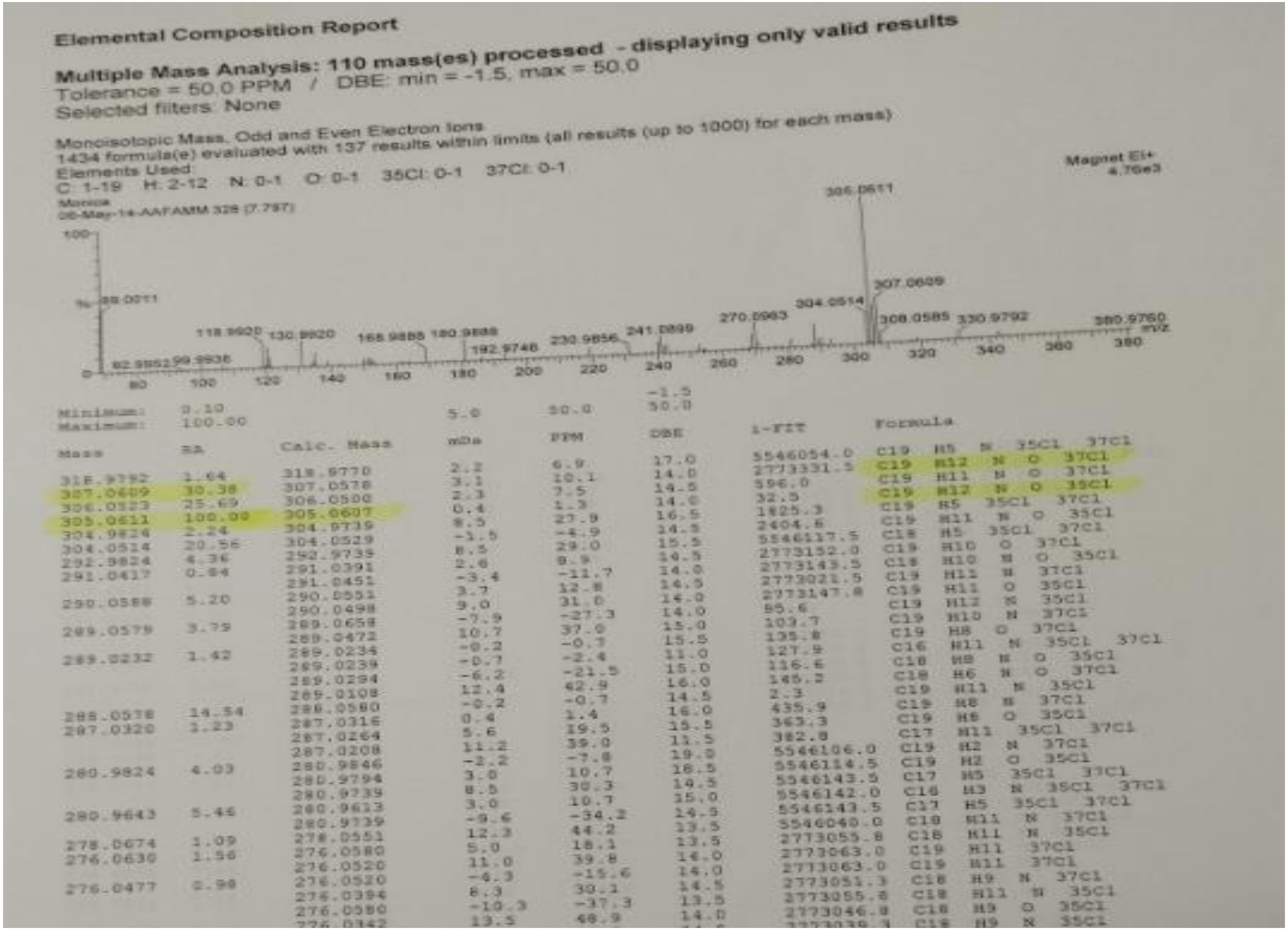

Elemental Composition Report

Multiple Mass Analysis: 110 mass(es) processed - displaying only valid results

Tolerance = 50.0 PPM / DBE: min = -1.5, max = 50.0

Selected filters: None

Monoisotopic Mass, Odd and Even Electron Ions

1434 formula(e) evaluated with 137 results within limits (all results (up to 1000) for each mass)

Elements Used:

C: 1-19 H: 2-12 N: 0-1 O: 0-1 35Cl: 0-1 37Cl: 0-1

Monica

06-May-14-AAFAMM 328 (7.797)

Minimum:	0.10				-1.5		
Maximum:	100.00		5.0	50.0	50.0		
Mass	RA	Calc. Mass	mDa	PPM	DBE	i-FIT	Formula
318.9792	1.64	318.9770	2.2	6.9	17.0	5546054.0	C19 H5 N 35Cl 37Cl
307.0609	30.38	307.0578	3.1	10.1	14.0	2773331.5	C19 H12 N O 37Cl
306.0523	25.69	306.0500	2.3	7.5	14.5	596.0	C19 H11 N O 37Cl
305.0611	100.00	305.0607	0.4	1.3	14.0	32.5	C19 H12 N O 35Cl
304.9824	2.24	304.9739	8.5	27.9	16.5	1825.3	C19 H5 35Cl 37Cl
304.0514	20.56	304.0529	-1.5	-4.9	14.5	2404.6	C19 H11 N O 35Cl
292.9824	4.36	292.9739	8.5	29.0	15.5	5546117.5	C18 H5 35Cl 37Cl
291.0417	0.64	291.0391	2.6	8.9	14.5	2773152.0	C19 H10 O 37Cl
		291.0451	-3.4	-11.7	14.0	2773143.5	C18 H10 N O 35Cl
290.0588	5.20	290.0551	3.7	12.8	14.5	2773021.5	C19 H11 N 37Cl
		290.0498	9.0	31.0	14.0	2773147.8	C19 H11 O 35Cl
289.0579	3.75	289.0658	-7.9	-27.3	14.0	95.6	C19 H12 N 35Cl
		289.0472	10.7	37.0	15.0	103.7	C19 H10 N 37Cl
289.0232	1.42	289.0234	-0.2	-0.7	15.5	135.8	C19 H8 O 37Cl
		289.0239	-0.7	-2.4	11.0	127.9	C16 H11 N 35Cl 37Cl
		289.0294	-6.2	-21.5	15.0	116.6	C18 H8 N O 35Cl
		289.0108	12.4	42.9	16.0	145.2	C18 H6 N O 37Cl
288.0578	14.54	288.0580	-0.2	-0.7	14.5	2.3	C19 H11 N 35Cl
287.0320	1.23	287.0316	0.4	1.4	16.0	435.9	C19 H8 N 37Cl
		287.0264	5.6	19.5	15.5	363.3	C19 H6 O 35Cl
		287.0208	11.2	39.0	11.5	382.8	C17 H11 35Cl 37Cl
280.9824	4.03	280.9846	-2.2	-7.8	19.0	5546106.0	C19 H2 N 37Cl
		280.9794	3.0	10.7	18.5	5546114.5	C19 H2 O 35Cl
		280.9739	8.5	30.3	14.5	5546143.5	C17 H5 35Cl 37Cl
280.9643	5.46	280.9613	3.0	10.7	15.0	5546142.0	C16 H3 N 35Cl 37Cl
		280.9739	-9.6	-34.2	14.5	5546143.5	C17 H5 35Cl 37Cl
278.0674	1.09	278.0551	12.3	44.2	13.5	5546040.0	C18 H11 N 37Cl
276.0630	1.56	276.0580	5.0	18.1	13.5	2773055.8	C18 H11 N 35Cl
		276.0520	11.0	39.8	14.0	2773063.0	C19 H11 37Cl
276.0477	0.98	276.0520	-4.3	-15.6	14.0	2773063.0	C19 H11 37Cl
		276.0394	8.3	30.1	14.5	2773051.3	C18 H9 N 37Cl
		276.0580	-10.3	-37.3	13.5	2773055.8	C18 H11 N 35Cl
		276.0342	13.5	48.9	14.0	2773046.8	C18 H9 O 35Cl
		[illegible]	[illegible]	[illegible]	[illegible]	2773039.3	C18 H9 N 35Cl

HRMS of compound **19**:

Elemental Composition Report

Multiple Mass Analysis: 50 mass(es) processed - displaying only valid results

Tolerance = 10.0 PPM / DBE: min = -1.5, max = 50.0

Selected filters: None

Monoisotopic Mass, Odd and Even Electron Ions

795 formula(e) evaluated with 13 results within limits (all results (up to 1000) for each mass)

Elements Used:

C: 5-14 H: 2-9 N: 0-1 O: 0-12

Monica

07-May-14-AAFAMM 254 (6.038)

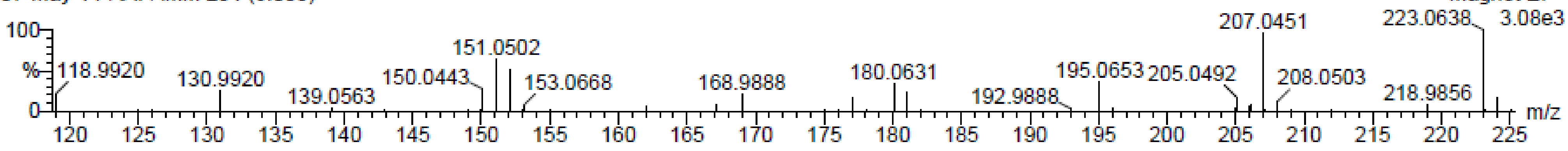

Minimum:	0.10				-1.5		
Maximum:	100.00		5.0	10.0	50.0		

Mass	RA	Calc. Mass	mDa	PPM	DBE	i-FIT	Formula
223.0638	100.00	223.0633	0.5	2.2	11.0	0.9	C14 H9 N O2
211.9837	0.73	211.9831	0.6	2.8	7.5	5546025.5	C7 H2 N O7
207.0451	95.56	207.0446	0.5	2.4	11.5	11.1	C14 H7 O2
206.0618	7.26	206.0606	1.2	5.8	11.5	1690.8	C14 H8 N O
205.0492	17.18	205.0501	-0.9	-4.4	7.5	1539.7	C11 H9 O4
181.0671	3.75	181.0653	1.8	9.9	9.5	5546061.5	C13 H9 O
181.0488	2.17	181.0501	-1.3	-7.2	5.5	5546035.0	C9 H9 O4
178.0651	2.47	178.0657	-0.6	-3.4	10.5	2773549.8	C13 H8 N
177.0565	16.61	177.0552	1.3	7.3	6.5	2773025.0	C10 H9 O3
		177.0578	-1.3	-7.3	11.0	2773021.5	C13 H7 N
175.0436	1.25	175.0422	1.4	8.0	12.0	283.8	C13 H5 N
167.0728	8.16	167.0735	-0.7	-4.2	9.0	5546123.5	C12 H9 N
149.0256	2.29	149.0265	-0.9	-6.0	11.0	1495.4	C11 H3 N

HRMS of compound **20**:

Elemental Composition Report

Multiple Mass Analysis: 45 mass(es) processed - displaying only valid results

Tolerance = 10.0 PPM / DBE: min = -1.5, max = 50.0
Selected filters: None

Monoisotopic Mass, Odd and Even Electron Ions
199 formula(e) evaluated with 6 results within limits (all results (up to 1000) for each mass)
Elements Used:
C: 5-14 H: 2-17 N: 0-1 O: 0-1

Monica
06-May-14-EAFAMM 331 (7.868)

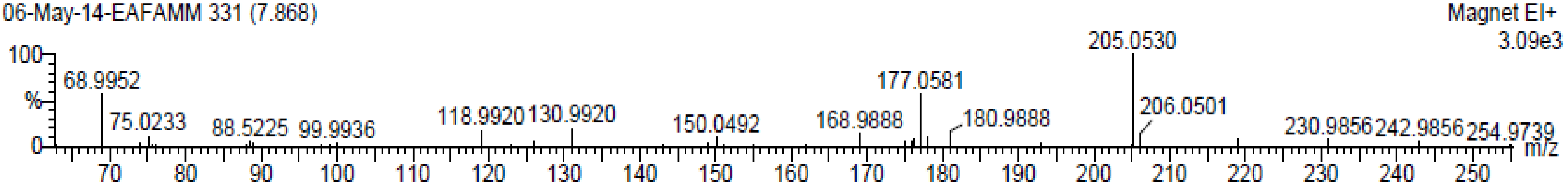

Minimum:	0.10				-1.5		
Maximum:	100.00		5.0	10.0	50.0		

Mass	RA	Calc. Mass	mDa	PPM	DBE	i-FIT	Formula
205.0530	100.00	205.0528	0.2	1.0	12.0	2773584.3	C14 H7 N O
177.0581	57.99	177.0578	0.3	1.7	11.0	2773153.5	C13 H7 N
175.0436	5.84	175.0422	1.4	8.0	12.0	993.6	C13 H5 N
126.0468	5.45	126.0470	-0.2	-1.6	8.0	5546076.5	C10 H6
88.0180	0.93	88.0187	-0.7	-8.0	6.5	2773069.3	C6 H2 N
75.0233	11.02	75.0235	-0.2	-2.7	5.5	2773013.3	C6 H3

HRMS of compound **21**:

Elemental Composition Report

Multiple Mass Analysis: 62 mass(es) processed - displaying only valid results

Tolerance = 10.0 PPM / DBE: min = -1.5, max = 50.0

Selected filters: None

Monoisotopic Mass, Odd and Even Electron Ions

810 formula(e) evaluated with 15 results within limits (all results (up to 1000) for each mass)

Elements Used:

C: 5-14 H: 2-9 N: 0-1 O: 0-12

Monica

06-May-14-EAFAMM 119 (2.829)

Magnet EI+

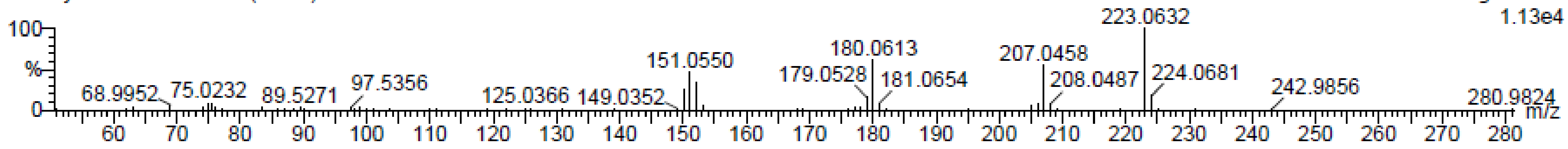

Minimum:	0.10				-1.5		
Maximum:	100.00		5.0	10.0	50.0		

Mass	RA	Calc. Mass	mDa	PPM	DBE	i-FIT	Formula
280.9824	0.68	280.9808	1.6	5.7	10.0	5546040.5	C10 H3 N O9
223.0632	100.00	223.0633	-0.1	-0.4	11.0	17.6	C14 H9 N O2
207.0458	55.52	207.0446	1.2	5.8	11.5	24.0	C14 H7 O2
206.0610	7.75	206.0606	0.4	1.9	11.5	3542.8	C14 H8 N O
181.0654	8.63	181.0653	0.1	0.6	9.5	2773062.5	C13 H9 O
168.0582	0.87	168.0575	0.7	4.2	9.0	5546052.0	C12 H8 O
151.0550	45.66	151.0548	0.2	1.3	9.5	1555.0	C12 H7
150.0479	24.18	150.0470	0.9	6.0	10.0	4179.3	C12 H6
99.0238	2.70	99.0235	0.3	3.0	7.5	100.2	C8 H3
98.0156	2.47	98.0157	-0.1	-1.0	8.0	197.5	C8 H2
87.0227	1.89	87.0235	-0.8	-9.2	6.5	5546078.5	C7 H3
86.0160	0.90	86.0157	0.3	3.5	7.0	2773112.3	C7 H2
76.0313	2.68	76.0313	0.0	0.0	5.0	2773084.8	C6 H4
75.0232	9.12	75.0235	-0.3	-4.0	5.5	185.0	C6 H3
74.0156	3.55	74.0157	-0.1	-1.4	6.0	659.3	C6 H2

HRMS of compound **26**:

Elemental Composition Report

Page 1

Multiple Mass Analysis: 16 mass(es) processed - displaying only valid results
Tolerance = 19.0 PPM / DBE: min = -1.5, max = 50.0
Selected filters: None

Monoisotopic Mass, Odd and Even Electron Ions
388 formula(e) evaluated with 8 results within limits (all results (up to 1000) for each mass)
Elements Used:
C: 5-19 H: 0-13 N: 0-1 O: 0-12

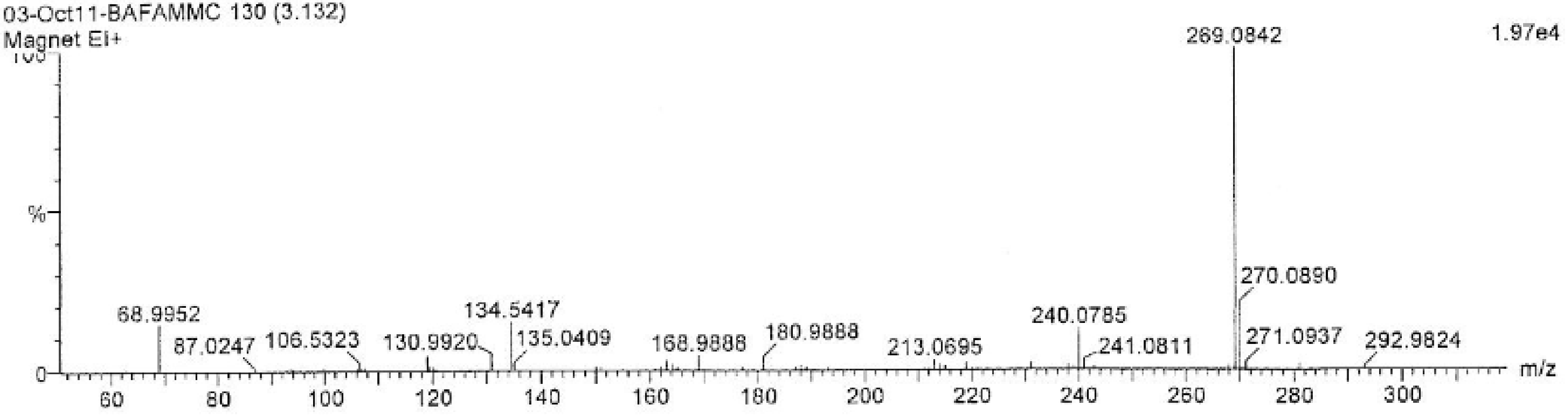

Minimum:	2.00				-1.5		
Maximum:	100.00		5.0	19.0	50.0		

Mass	RA	Calc. Mass	mDa	PPM	DBE	i-FIT	Formula
270.0890	21.10	270.0919	-2.9	-10.7	14.5	2774491.3	C19 H12 N O
269.0842	100.00	269.0841	0.1	0.4	15.0	6.3	C19 H11 N O
		269.0814	2.8	10.4	10.5	48.1	C16 H13 O4
240.0785	12.50	240.0786	-0.1	-0.4	10.0	29.1	C15 H12 O3
		240.0813	-2.8	-11.7	14.5	12.3	C18 H10 N
213.0695	2.90	213.0704	-0.9	-4.2	13.5	144.4	C17 H9
163.0552	2.91	163.0548	0.4	2.5	10.5	119.0	C13 H7

HRMS of compound **27**:

Elemental Composition Report

Electrospray

Page 1

Multiple Mass Analysis: 3 mass(es) processed
Tolerance = 5.0 PPM / DBE: min = -20.0, max = 1000.0
Element prediction: Off
Number of isotope peaks used for i-FIT = 2

Monoisotopic Mass, Even Electron Ions
8121 formula(e) evaluated with 27 results within limits (up to 50 closest results for each mass)
Elements Used:
C: 0-52 H: 0-120 N: 0-6 O: 0-12 Na: 0-1 79Br: 0-1 81Br: 0-1

H-2129- Monica (82-4 (1)) 15 (0.521)

2: TOF MS ES+
1.43e+003

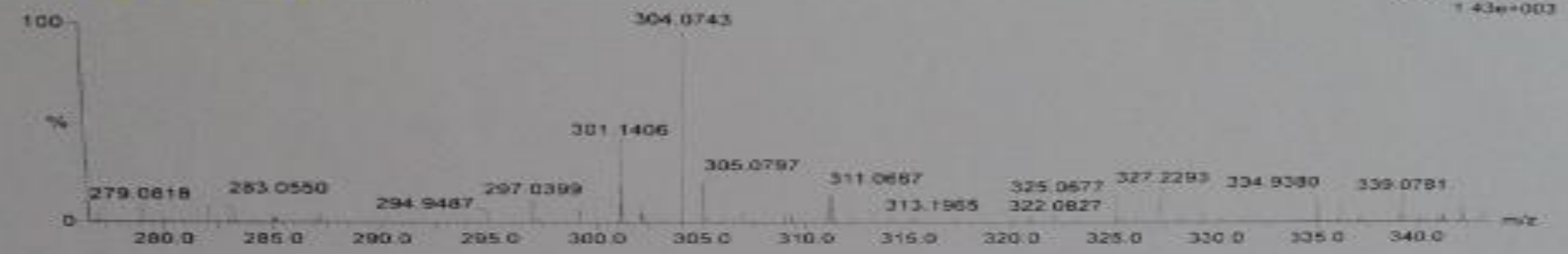

Mass	RA	Calc. Mass	mDa	PPM	DBE	i-FIT	Formula
Minimum:	20.00				-20.0		
Maximum:	100.00		100.0	5.0	1000.0		
301.1406	43.64	301.1410	-0.4	-1.3	-10.5	34.7	C H30 N6 O6 79Br
		301.1402	0.4	1.3	-4.5	9.0	C8 H29 N4 O Na 81Br
		301.1400	0.6	2.0	4.5	1.0	C13 H21 N2 O6
		301.1413	-0.7	-2.3	-14.5	33.8	C3 H35 O8 Na 79Br
		301.1413	-0.7	-2.3	-6.5	9.4	C9 H32 O5 81Br
		301.1413	-0.7	-2.3	9.5	0.0	C14 H17 N6 O2
		301.1397	0.9	3.0	-15.5	47.4	H34 N2 O10 79Br
		301.1416	-1.0	-3.3	5.5	0.0	C16 H22 O4 Na
304.0743	100.00	304.0741	0.2	0.7	1.5	56.3	C5 H14 N5 O10
		304.0746	-0.3	-1.0	-11.5	104.4	C H30 N5 O2 79Br 81Br
		304.0738	0.5	1.6	15.5	0.9	C20 H11 N O Na
		304.0749	-0.6	-2.0	0.5	32.0	C8 H20 N5 O Na 79Br
		304.0736	0.7	2.3	-4.5	50.6	C7 H24 N O5 Na 79Br
		304.0735	0.8	2.6	3.5	10.5	C13 H21 N O2 81Br
		304.0732	1.1	3.6	-16.5	135.0	H34 N O6 79Br 81Br
		304.0754	-1.1	-3.6	-9.5	111.0	C H25 N3 O5 81Br
		304.0757	-1.4	-4.6	2.5	35.8	C8 H15 N3 O8 Na
305.0797	21.06	305.0800	-0.3	-1.0	3.5	0.9	C11 H20 N4 O 81Br
		305.0800	-0.3	-1.0	-4.5	1.8	C5 H23 N4 O4 Na 79Br
		305.0803	-0.6	-2.0	15.5	10.1	C18 H10 N4 Na
		305.0790	0.7	2.3	10.5	6.4	C17 H14 O4 Na
		305.0787	1.0	3.3	-9.5	4.9	C4 H27 O8 Na 79Br
		305.0787	1.0	3.3	14.5	8.2	C15 H9 N6 O2
		305.0787	1.0	3.3	-1.5	0.8	C10 H24 O5 81Br
		305.0808	-1.1	-3.6	-2.5	2.4	C5 H18 N2 O11 Na
		305.0784	1.3	4.3	-5.5	5.2	C2 H22 N6 O6 79Br
		305.0811	-1.4	-4.6	-6.5	2.0	C6 H28 O8 79Br

HRMS of compound **29**:

Elemental Composition Report

Multiple Mass Analysis: 74 mass(es) processed - displaying only valid results

Tolerance = 10.0 PPM / DBE: min = -1.5, max = 50.0

Selected filters: None

Monoisotopic Mass, Odd and Even Electron Ions

188 formula(e) evaluated with 3 results within limits (all results (up to 1000) for each mass)

Elements Used:

C: 5-12 H: 2-12 O: 0-2

Monica Blaco

21Ene-13-BAFAMMA 140 (3.328)

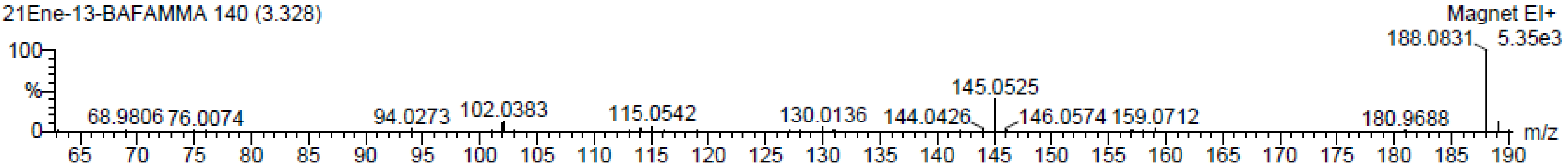

Minimum:	0.10				-1.5		
Maximum:	100.00		5.0	10.0	50.0		

Mass	RA	Calc. Mass	mDa	PPM	DBE	i-FIT	Formula
188.0831	100.00	188.0837	-0.6	-3.2	7.0	2.1	C12 H12 O2
142.0046	1.22	142.0055	-0.9	-6.3	9.0	2773064.3	C9 H2 O2
115.0542	6.63	115.0548	-0.6	-5.2	6.5	2773015.5	C9 H7

HRMS of compound **30**:

Elemental Composition Report

Page .

Multiple Mass Analysis: 70 mass(es) processed - displaying only valid results
Tolerance = 5.0 PPM / DBE: min = 0.0, max = 50.0
Isotope matching not enabled

Monoisotopic Mass, Odd and Even Electron Ions
3239 formula(e) evaluated with 26 results within limits (all results (up to 1000) for each mass)

Trujillo (MBF-32-44)E/I 70 ev Temp F 250 C
H14043-Trujillo (MBF-B2-44) E I 70 ev Temp F 250 C 70 (3.417)

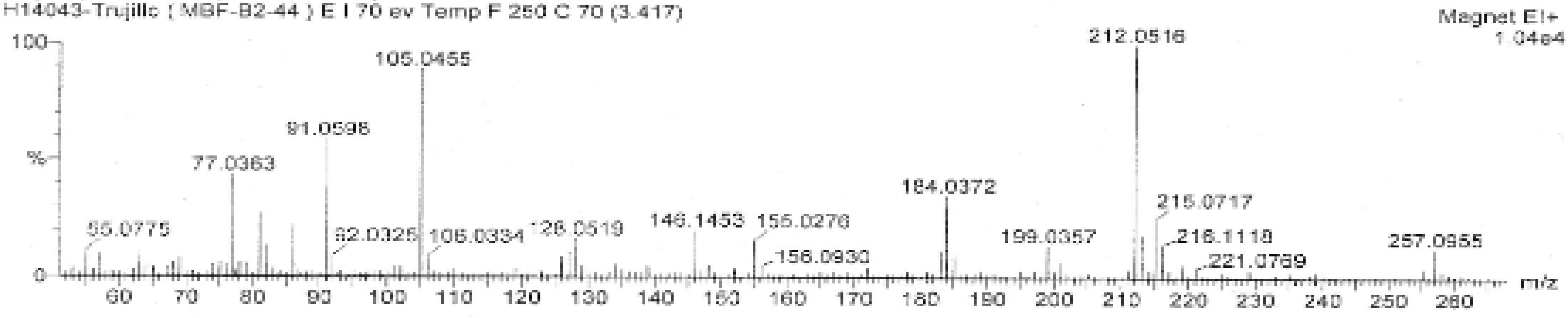

Minimum:	30.00				0.0	
Maximum:	100.00		200.0	5.0	50.0	

Mass	RA	Calc. Mass	mDa	PPM	DBE	Formula
212.0711	51.83	212.0712	-0.1	-0.3	9.5	C13 H10 N O2
212.0686	62.55	212.0685	0.1	0.6	5.0	C10 H12 O5
212.0662	71.59	212.0671	-0.9	-4.4	5.5	C8 H10 N3 O4
212.0589	91.88	212.0586	0.3	1.5	10.0	C12 H8 N2 O2
212.0565	95.67	212.0559	0.6	2.8	5.5	C9 H10 N O5
212.0492	98.66	212.0500	-0.8	-3.9	14.5	C16 H6 N
212.0468	94.92	212.0473	-0.5	-2.6	10.0	C13 H8 O3
		212.0460	0.8	3.8	10.5	C11 H6 N3 O2
212.0443	69.27	212.0433	1.0	4.6	6.0	C8 H8 N2 O5
212.0370	66.70	212.0374	-0.4	-2.1	15.0	C15 H4 N2
212.0346	57.76	212.0348	-0.2	-0.8	10.5	C12 H6 N O3
212.0322	49.70	212.0321	0.1	0.5	6.0	C9 H8 O6
212.0298	41.10	212.0307	-0.9	-4.3	6.5	C7 H6 N3 O5
184.0393	34.39	184.0399	-0.6	-3.0	9.5	C11 H6 N O2
184.0372	34.82	184.0372	0.0	0.1	5.0	C8 H8 O5
184.0351	34.24	184.0358	-0.7	-4.0	5.5	C6 H6 N3 O4
184.0308	32.12	184.0313	-0.5	-2.7	14.0	C15 H4
184.0266	30.18	184.0273	-0.7	-3.7	10.0	C10 H4 N2 O2
105.0551	43.09	105.0552	-0.1	-0.7	0.5	C4 H9 O3
105.0539	51.86	105.0538	0.1	0.7	1.0	C2 H7 N3 O2
105.0455	89.07	105.0453	0.2	2.2	5.5	C6 H5 N2
105.0431	87.73	105.0426	0.5	4.8	1.0	C3 H7 N O3
105.0323	33.66	105.0327	-0.4	-3.8	6.0	C5 H3 N3
91.0629	38.75	91.0633	-0.4	-4.7	0.0	C3 H9 N O2
91.0546	34.77	91.0548	-0.2	-1.9	4.5	C7 H7
77.0390	31.70	77.0391	-0.1	-1.6	4.5	C6 H5

HRMS of compound **32**:

Elemental Composition Report

Page 1

Multiple Mass Analysis: 53 mass(es) processed - displaying only valid results
Tolerance = 10.0 PPM / DBE: min = -1.5, max = 50.0
Selected filters: None

Monoisotopic Mass, Odd and Even Electron Ions
168 formula(e) evaluated with 12 results within limits (all results (up to 1000) for each mass)
Elements Used:
C: 5-13 H: 2-12 O: 0-3
Monica
18-Nov13-AAFAMMA 345 (8.201)

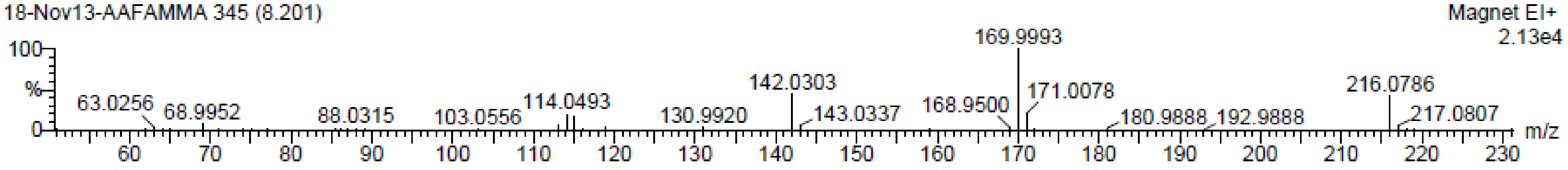

Minimum:	0.10				-1.5		
Maximum:	100.00		5.0	10.0	50.0		

Mass	RA	Calc. Mass	mDa	PPM	DBE	i-FIT	Formula
216.0786	41.60	216.0786	0.0	0.0	8.0	6.9	C13 H12 O3
172.0145	2.19	172.0160	-1.5	-8.7	9.0	5546217.5	C10 H4 O3
171.0078	19.95	171.0082	-0.4	-2.3	9.5	2773797.0	C10 H3 O3
169.9993	100.00	170.0004	-1.1	-6.5	10.0	436.4	C10 H2 O3
131.0486	0.55	131.0497	-1.1	-8.4	6.5	5546052.0	C9 H7 O
115.0552	16.34	115.0548	0.4	3.5	6.5	2773120.3	C9 H7
113.0398	4.93	113.0391	0.7	6.2	7.5	3826.9	C9 H5
103.0556	1.56	103.0548	0.8	7.8	5.5	5546138.5	C8 H7
89.0392	2.09	89.0391	0.1	1.1	5.5	5546179.5	C7 H5
88.0315	2.28	88.0313	0.2	2.3	6.0	2773200.8	C7 H4
86.0154	0.67	86.0157	-0.3	-3.5	7.0	355.9	C7 H2
65.0389	1.10	65.0391	-0.2	-3.1	3.5	5546068.5	C5 H5

HRMS of compound **33**:

Elemental Composition Report

Page 1

Multiple Mass Analysis: 536 mass(es) processed - displaying only valid results
Tolerance = 10.0 PPM / DBE: min = -1.5, max = 50.0
Selected filters: None

Monoisotopic Mass, Odd and Even Electron Ions
1658 formula(e) evaluated with 21 results within limits (all results (up to 1000) for each mass)
Elements Used:
C: 5-16 H: 2-12 O: 0-4

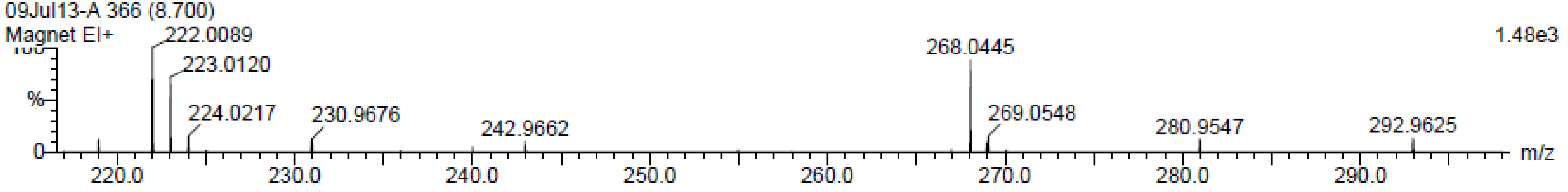

Minimum:	0.10				-1.5	
Maximum:	100.00		5.0	10.0	50.0	

Mass	RA	Calc. Mass	mDa	PPM	DBE	Formula
268.0742	31.24	268.0736	0.6	2.2	11.0	C16 H12 O4
268.0717	39.35	268.0736	-1.9	-7.1	11.0	C16 H12 O4
240.0426	0.14	240.0423	0.3	1.2	11.0	C14 H8 O4
240.0404	0.74	240.0423	-1.9	-7.9	11.0	C14 H8 O4
225.0194	0.88	225.0188	0.6	2.7	11.5	C13 H5 O4
225.0173	0.27	225.0188	-1.5	-6.7	11.5	C13 H5 O4
224.0486	3.79	224.0473	1.3	5.8	11.0	C14 H8 O3
224.0465	5.07	224.0473	-0.8	-3.6	11.0	C14 H8 O3
224.0114	13.05	224.0110	0.4	1.8	12.0	C13 H4 O4
224.0093	12.98	224.0110	-1.7	-7.6	12.0	C13 H4 O4
223.0408	20.82	223.0395	1.3	5.8	11.5	C14 H7 O3
223.0387	27.38	223.0395	-0.8	-3.6	11.5	C14 H7 O3
223.0038	59.30	223.0031	0.7	3.1	12.5	C13 H3 O4

HRMS of compound **34**:

Elemental Composition Report

Multiple Mass Analysis: 116 mass(es) processed - displaying only valid results

Tolerance = 10.0 PPM / DBE: min = -1.5, max = 50.0

Selected filters: None

Monoisotopic Mass, Odd and Even Electron Ions

619 formula(e) evaluated with 20 results within limits (all results (up to 1000) for each mass)

Elements Used:

C: 5-14 H: 2-18 O: 0-4

Monica

18-Nov13-AAFAMMA 278 (6.608)

Magnet EI+

4.18e3

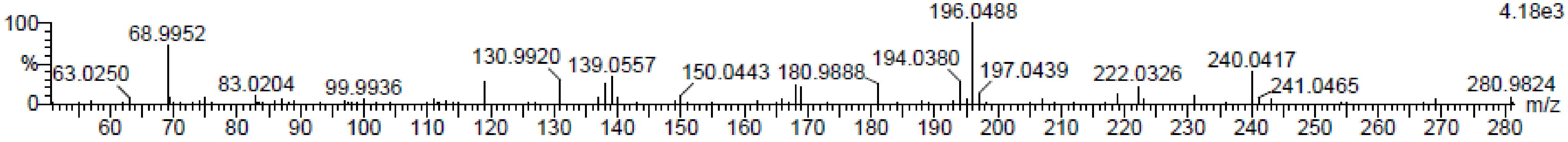

Minimum:	0.10				-1.5		
Maximum:	100.00		5.0	10.0	50.0		

Mass	RA	Calc. Mass	mDa	PPM	DBE	i-FIT	Formula
240.0417	40.58	240.0423	-0.6	-2.5	11.0	15.9	C14 H8 O4
223.0392	5.87	223.0395	-0.3	-1.3	11.5	5546125.0	C14 H7 O3
222.0326	21.58	222.0317	0.9	4.1	12.0	2773132.5	C14 H6 O3
194.0380	27.76	194.0368	1.2	6.2	11.0	2090.1	C13 H6 O2
188.0835	3.09	188.0837	-0.2	-1.1	7.0	2773014.8	C12 H12 O2
168.0569	23.69	168.0575	-0.6	-3.6	9.0	2773059.5	C12 H8 O
151.0043	0.51	151.0031	1.2	7.9	6.5	5546025.5	C7 H3 O4
140.0623	7.22	140.0626	-0.3	-2.1	8.0	5546142.5	C11 H8
139.0557	33.48	139.0548	0.9	6.5	8.5	2773079.0	C11 H7
137.0400	8.12	137.0391	0.9	6.6	9.5	1184.4	C11 H5
127.0554	1.63	127.0548	0.6	4.7	7.5	5546037.0	C10 H7
101.0386	0.22	101.0391	-0.5	-4.9	6.5	2773032.3	C8 H5
[illegible]	[illegible]	[illegible]	[illegible]	[illegible]	[illegible]	[illegible]	[illegible]

HRMS of compound **35**:

Elemental Composition Report

Multiple Mass Analysis: 112 mass(es) processed - displaying only valid results

Tolerance = 10.0 PPM / DBE: min = -1.5, max = 50.0

Selected filters: None

Monoisotopic Mass, Odd and Even Electron Ions

345 formula(e) evaluated with 7 results within limits (all results (up to 1000) for each mass)

Elements Used:

C: 2-12 H: 0-7 N: 0-1 O: 0-1

Monica

29-Sep-15-AAFAMM 326 (8.298)

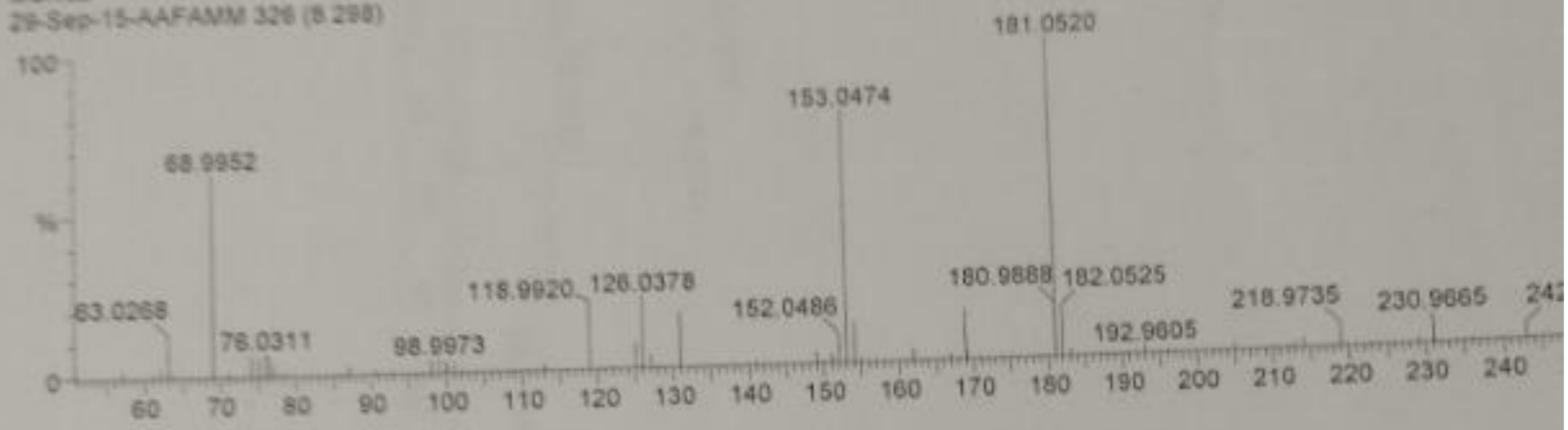

Minimum:	0.10				-1.5				
Maximum:	100.00		5.0	10.0	50.0				
Mass	RA	Calc. Mass	mDa	PPM	DBE	i-FIT	Formula		
181.0520	100.00	181.0528	-0.8	-4.4	10.0	9.4	C12 H7	N	O
169.0542	1.45	169.0528	1.4	8.3	9.0	5546037.0	C11 H7	N	O
157.0533	0.45	157.0528	0.5	3.2	8.0	5546026.0	C10 H7	N	O
152.0486	8.87	152.0500	-1.4	-9.2	9.5	1893.7	C11 H6	N	
125.0381	5.98	125.0391	-1.0	-8.0	8.5	521.9	C10 H5		
87.0241	2.18	87.0235	0.6	6.9	6.5	5546038.0	C7 H3		
76.0311	6.85	76.0313	-0.2	-2.6	5.0	2773033.5	C6 H4		

HRMS of compound 2-methoxy-1,6-naphthalenedicarbaldehyde:

Elemental Composition Report

Multiple Mass Analysis: 153 mass(es) processed - displaying only valid results
Tolerance = 10.0 PPM / DBE: min = -1.5, max = 50.0
Selected filters: None

Monoisotopic Mass, Odd and Even Electron Ions
543 formula(e) evaluated with 17 results within limits (all results (up to 1000) for each mass)
Elements Used:
C: 2-13 H: 0-10 O: 0-3
Monica
29-Sep-15-AAFAMM (10.742) None

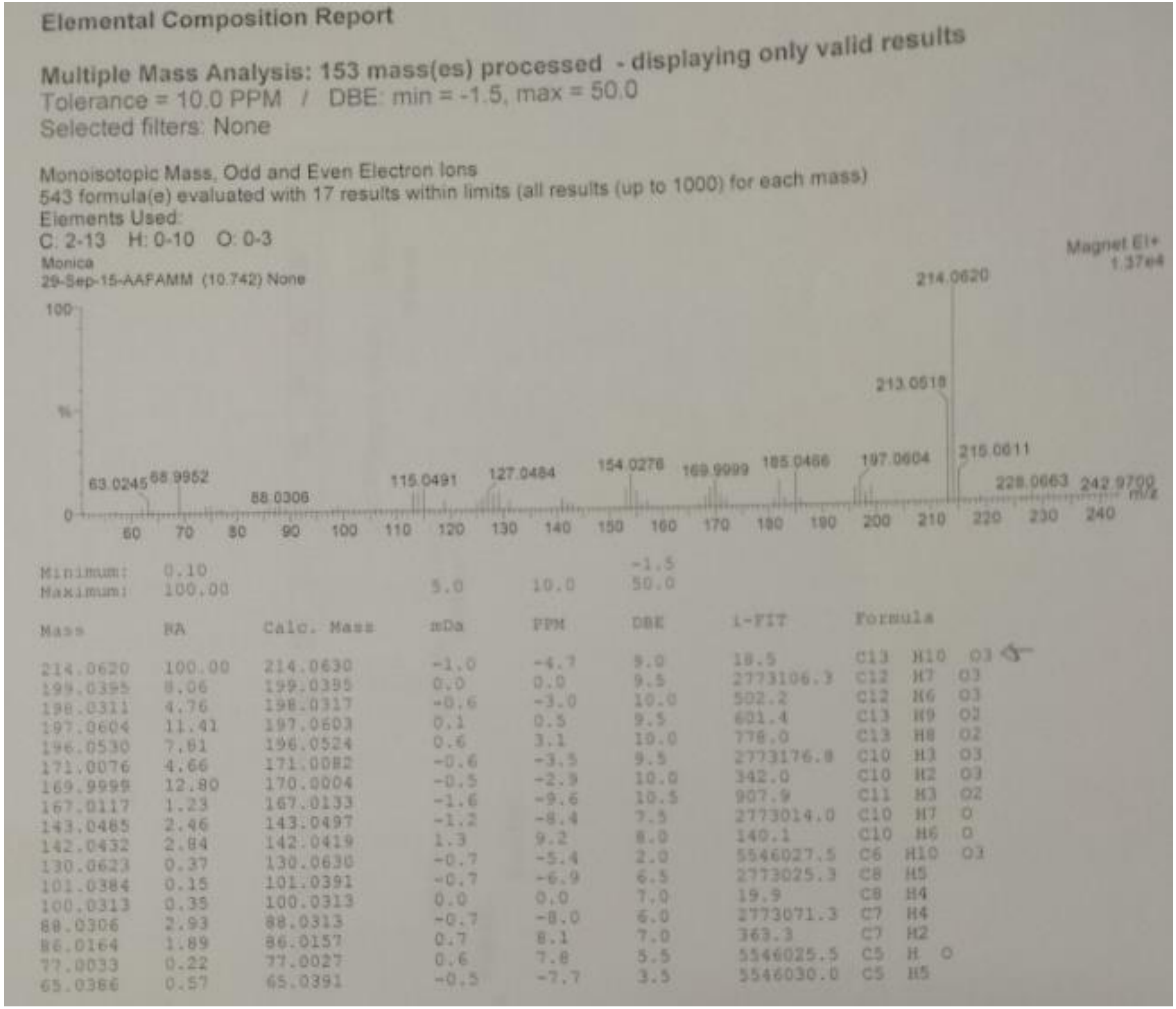

Minimum:	0.10				-1.5		
Maximum:	100.00		5.0	10.0	50.0		

Mass	RA	Calc. Mass	mDa	PPM	DBE	i-FIT	Formula
214.0620	100.00	214.0630	-1.0	-4.7	9.0	18.5	C13 H10 O3
199.0395	8.06	199.0395	0.0	0.0	9.5	2773106.3	C12 H7 O3
198.0311	4.76	198.0317	-0.6	-3.0	10.0	502.2	C12 H6 O3
197.0604	11.41	197.0603	0.1	0.5	9.5	601.4	C13 H9 O2
196.0530	7.81	196.0524	0.6	3.1	10.0	778.0	C13 H8 O2
171.0076	4.66	171.0082	-0.6	-3.5	9.5	2773176.8	C10 H3 O3
169.9999	12.80	170.0004	-0.5	-2.9	10.0	342.0	C10 H2 O3
167.0117	1.23	167.0133	-1.6	-9.6	10.5	907.9	C11 H3 O2
143.0485	2.46	143.0497	-1.2	-8.4	7.5	2773014.0	C10 H7 O
142.0432	2.84	142.0419	1.3	9.2	8.0	140.1	C10 H6 O
130.0623	0.37	130.0630	-0.7	-5.4	2.0	5546027.5	C6 H10 O3
101.0384	0.15	101.0391	-0.7	-6.9	6.5	2773025.3	C8 H5
100.0313	0.35	100.0313	0.0	0.0	7.0	19.9	C8 H4
88.0306	2.93	88.0313	-0.7	-8.0	6.0	2773071.3	C7 H4
86.0164	1.89	86.0157	0.7	8.1	7.0	363.3	C7 H2
77.0033	0.22	77.0027	0.6	7.8	5.5	5546025.5	C5 H O
65.0366	0.57	65.0391	-0.5	-7.7	3.5	5546030.0	C5 H5

HRMS of compound **36**:

Elemental Composition Report

Multiple Mass Analysis: 1569 mass(es) processed - displaying only valid results

Tolerance = 10.0 PPM / DBE: min = -1.5, max = 50.0

Selected filters: None

Monoisotopic Mass, Odd and Even Electron Ions

6788 formula(e) evaluated with 15 results within limits (all results (up to 1000) for each mass)

Elements Used:

C: 13-13 H: 2-9 N: 0-1 O: 0-2

Monica

29-Sep-15-A 518 (13.185)

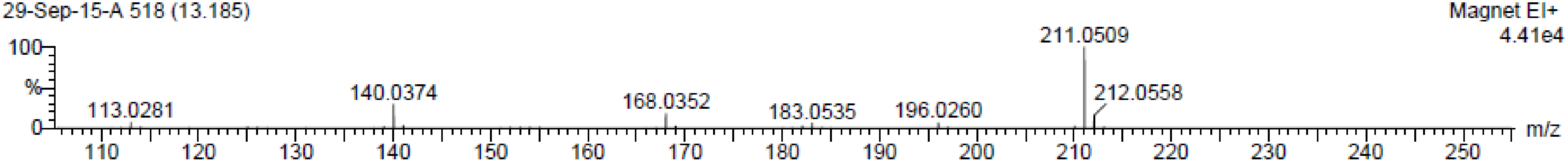

Minimum:	0.10				-1.5	
Maximum:	100.00		5.0	10.0	50.0	
Mass	RA	Calc. Mass	mDa	PPM	DBE	Formula
211.0645	72.24	211.0633	1.2	5.7	10.0	C13 H9 N O2
211.0626	76.50	211.0633	-0.7	-3.3	10.0	C13 H9 N O2
210.0566	1.48	210.0555	1.1	5.2	10.5	C13 H8 N O2
210.0547	1.47	210.0555	-0.8	-3.8	10.5	C13 H8 N O2
197.0608	0.42	197.0603	0.5	2.5	9.5	C13 H9 O2
197.0589	0.49	197.0603	-1.4	-7.1	9.5	C13 H9 O2
196.0530	2.59	196.0524	0.6	3.1	10.0	C13 H8 O2
196.0512	3.00	196.0524	-1.2	-6.1	10.0	C13 H8 O2
195.0433	0.15	195.0446	-1.3	-6.7	10.5	C13 H7 O2
192.0451	0.13	192.0449	0.2	1.0	11.5	C13 H6 N O
192.0434	0.12	192.0449	-1.5	-7.8	11.5	C13 H6 N O
181.0646	0.14	181.0653	-0.7	-3.9	9.5	C13 H9 O

HRMS of compound **37**:

Elemental Composition Report

Multiple Mass Analysis: 148 mass(es) processed - displaying only valid results

Tolerance = 10.0 PPM / DBE: min = -1.5, max = 50.0

Selected filters: None

Monoisotopic Mass, Odd and Even Electron Ions

1128 formula(e) evaluated with 25 results within limits (all results (up to 1000) for each mass)

Elements Used:

C: 5-15 H: 2-14 N: 0-1 O: 0-3

Monica

28-Sep-15-CAFAMMA 125 (3.182)

Magnet EI+

9.21e3

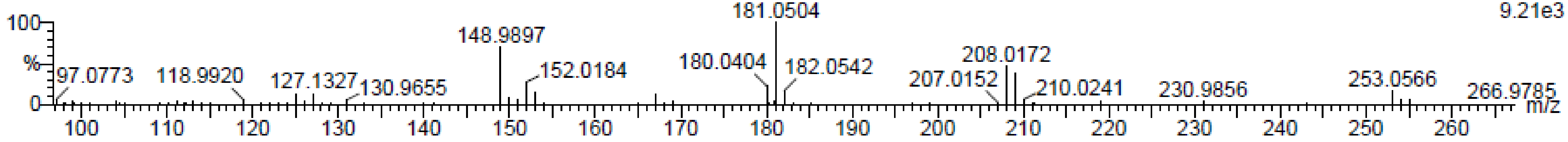

Mass	RA	Calc. Mass	mDa	PPM	DBE	i-FIT	Formula
Minimum:	0.10				-1.5		
Maximum:	100.00		5.0	10.0	50.0		
256.0963	0.35	256.0974	-1.1	-4.3	9.5	5546030.5	C15 H14 N O3
253.0729	13.12	253.0739	-1.0	-4.0	11.0	295.7	C15 H11 N O3
209.0220	37.68	209.0239	-1.9	-9.1	11.5	4.5	C13 H5 O3
208.0172	46.43	208.0160	1.2	5.8	12.0	1351.1	C13 H4 O3
199.0250	0.82	199.0269	-1.9	-9.5	10.0	5546041.5	C11 H5 N O3
183.0821	0.23	183.0810	1.1	6.0	8.5	5546027.0	C13 H11 O
181.1004	0.74	181.1017	-1.3	-7.2	8.5	2773041.8	C14 H13
179.0376	0.25	179.0371	0.5	2.8	11.0	5668.7	C12 H5 N O
178.0797	0.23	178.0783	1.4	7.9	10.0	5546027.0	C14 H10
178.0634	0.29	178.0630	0.4	2.2	6.0	1184.5	C10 H10 O3
176.0514	0.20	176.0500	1.4	8.0	11.5	2773026.8	C13 H6 N
173.0976	0.17	173.0966	1.0	5.8	6.5	5546026.0	C12 H13 O
169.0666	0.22	169.0653	1.3	7.7	8.5	2773026.8	C12 H9 O
164.0474	0.45	164.0473	0.1	0.6	6.0	2773034.5	C9 H8 O3
[illegible]	[illegible]	[illegible]	[illegible]	[illegible]	[illegible]	[illegible]	[illegible]

HRMS of compound **38**:

Elemental Composition Report

Multiple Mass Analysis: 54 mass(es) processed - displaying only valid results
Tolerance = 10.0 PPM / DBE: min = -1.5, max = 50.0
Selected filters: None

Monoisotopic Mass, Odd and Even Electron Ions
214 formula(e) evaluated with 8 results within limits (all results (up to 1000) for each mass)
Elements Used:
C: 5-14 H: 2-13 O: 0-4
Tere
02-Jul-14-AAFAMM 47 (1.196)

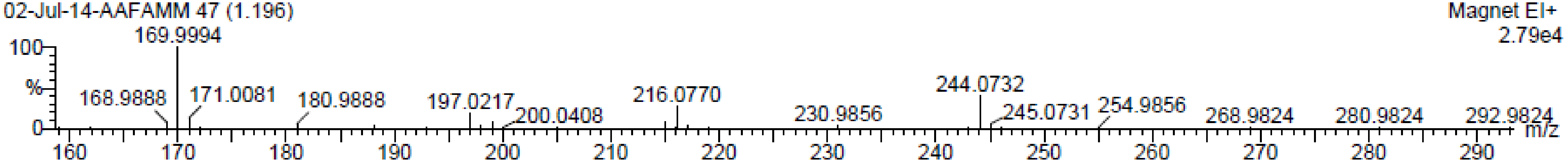

Minimum:	0.10				-1.5		
Maximum:	100.00		5.0	10.0	50.0		

Mass	RA	Calc. Mass	mDa	PPM	DBE	i-FIT	Formula
244.0732	40.86	244.0736	-0.4	-1.6	9.0	9.5	C14 H12 O4
243.0639	0.24	243.0657	-1.8	-7.4	9.5	6518.0	C14 H11 O4
216.0770	27.06	216.0786	-1.6	-7.4	8.0	19.9	C13 H12 O3
186.0317	0.25	186.0317	0.0	0.0	9.0	2773526.8	C11 H6 O3
172.0149	2.05	172.0160	-1.1	-6.4	9.0	2773038.8	C10 H4 O3
171.0081	12.87	171.0082	-0.1	-0.6	9.5	26.1	C10 H3 O3
170.0355	0.10	170.0368	-1.3	-7.6	9.0	2773342.3	C11 H6 O2
169.9994	100.00	170.0004	-1.0	-5.9	10.0	89.3	C10 H2 O3

HRMS of compound **40**:

Page 1

Elemental Composition Report

Multiple Mass Analysis: 209 mass(es) processed - displaying only valid results
Tolerance = 10.0 PPM / DBE: min = -1.5, max = 50.0
Selected filters: None

Monoisotopic Mass, Odd and Even Electron Ions
1757 formula(e) evaluated with 8 results within limits (all results (up to 1000) for each mass)
Elements Used:
C: 5-12 H: 0-6 N: 0-1 O: 0-1 79Br: 0-1 81Br: 0-1

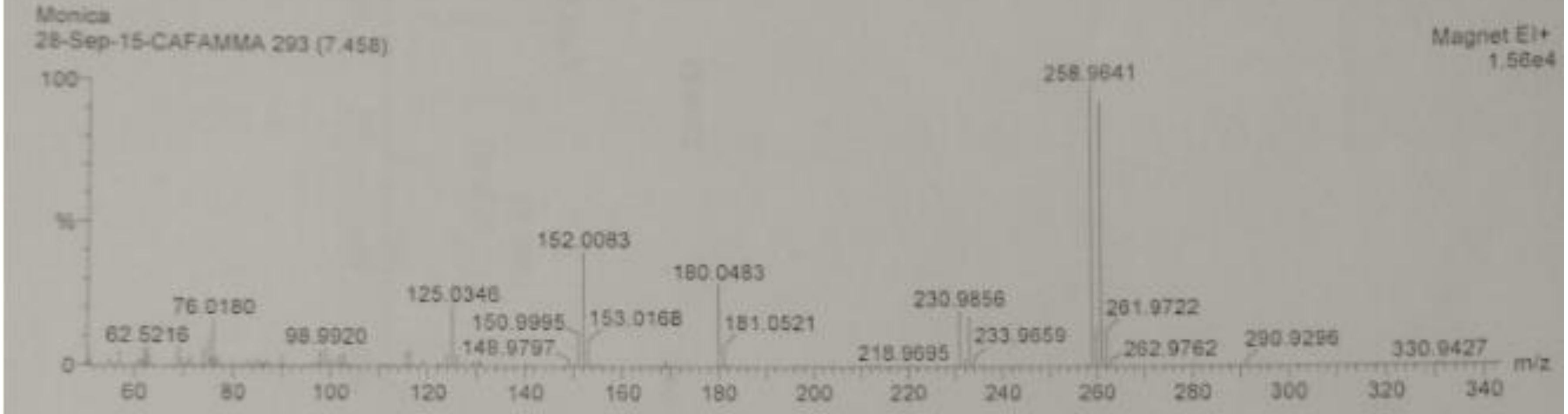

Minimum:	0.10				-1.5		
Maximum:	100.00		5.0	10.0	50.0		

Mass	RA	Calc. Mass	mDa	PPM	DBE	i-FIT	Formula
260.9625	93.97	260.9612	1.3	5.0	10.0	48.4	C12 H6 N O 81Br —
258.9641	100.00	258.9633	0.8	3.1	10.0	16.6	C12 H6 N O 79Br —
218.9695	0.72	218.9684	1.1	5.0	8.0	5546079.5	C10 H6 N 79Br
167.0361	0.41	167.0371	-1.0	-6.0	10.0	2773043.3	C11 H5 N O
123.0107	0.90	123.0109	-0.2	-1.6	10.0	2167.9	C9 H N
122.0161	1.14	122.0157	0.4	3.3	10.0	384.4	C10 H2
113.0255	0.19	113.0265	-1.0	-8.8	8.0	5546026.5	C8 H3 N
76.0180	16.22	76.0187	-0.7	-9.2	5.5	2773149.0	C5 H2 N
