# Supplementary material for: Synthesis of Substituted 1H-Phenalen-1-ones and Nitrogen-Containing Heterocyclic Analogues as Potential Anti-Plasmodial Agents
Source: Molecules. 2025 Dec 5;30(24):4667. doi: 10.3390/molecules30244667 (PMC12735704; doi:10.3390/molecules30244667)
Supplement: Supplementary file 1 [file molecules-30-04667-s001.zip › Supplementary Material-S3.pdf]

Supplementary Materials

Synthesis of Substituted 1*H*-Phenalen-1-ones and Nitrogen-Containing Heterocyclic Analogues as Potential Anti-plasmodial Agents

Teresa Abad-Grillo, [1,*] Grant McNaughton-Smith, [2,*] Mónica Blanco Freijó, [1] David Gutiérrez[3] and Ninoska Flores[3]

[1] Departamento de Química Orgánica, Universidad de La Laguna, Avenida Astrofísico Francisco Sánchez, 2, 38206 La Laguna, Tenerife, Spain; tereabad@ull.edu.es

[2] Centro Atlántico del Medicamento S.A (CEAMED S.A.), PCTT, 38200 La Laguna, Tenerife, Spain; gmcsmith@ceamedsa.com

[3] Instituto de Investigaciones Fármaco Bioquímicas, Facultad de Ciencias Farmacéuticas y Bioquímicas, Universidad Mayor de San Andrés, Avenida Saavedra 2224, Miraflores, La Paz, Bolivia

* Correspondence: tereabad@ull.edu.es; gmcsmith@ceamedsa.com

Contents:

UV-VIS spectra

Compound **5**:

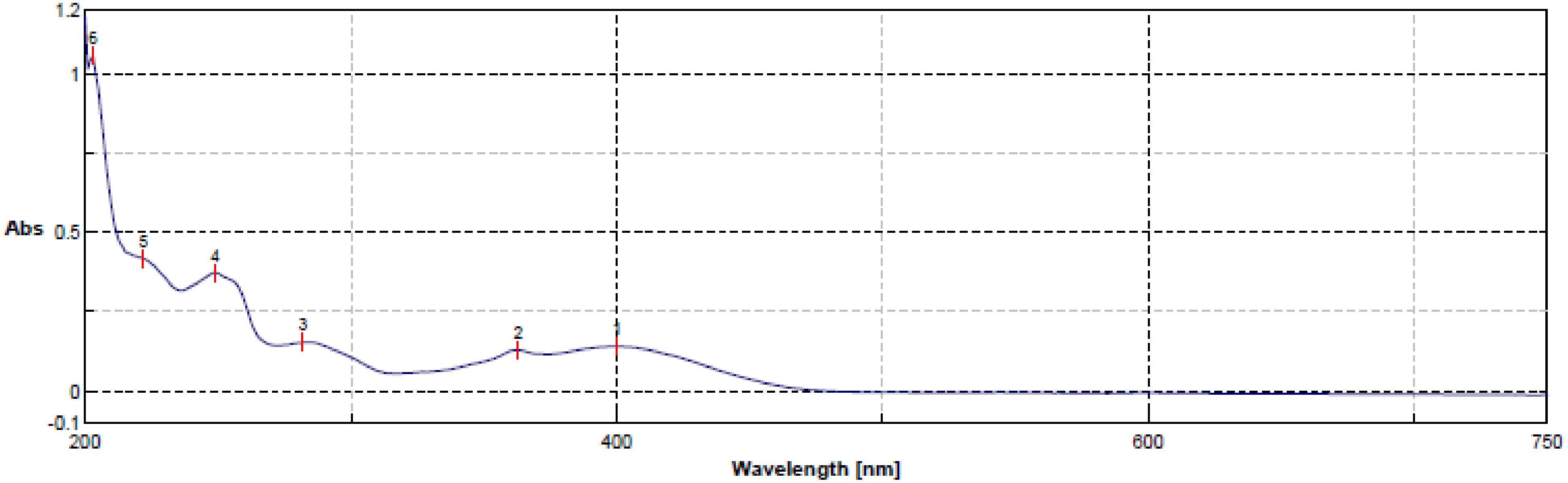

Date	05/06/2014 12:58	File name	F162_B4-21-3B-3
Model	V-560		
Serial No.	A029479		
Band width	1.0 nm		
Response	Fast		
Measurement range	750 - 200 nm		
Data pitch	1nm		
Scanning speed	400nm/min		
Sample ID	491		
No. of cycle	1		
Sample name			
Operator	Jesús Trujillo Vázquez		
Comment			

No.	nm	Abs	No.	nm	Abs	No.	nm	Abs	No.	nm	Abs	No.	nm	Abs
1	400	0.13952	2	363	0.12807	3	282	0.1519	4	249	0.37241	5	222	0.41659
6	203	1.05637												

Compound **6**:

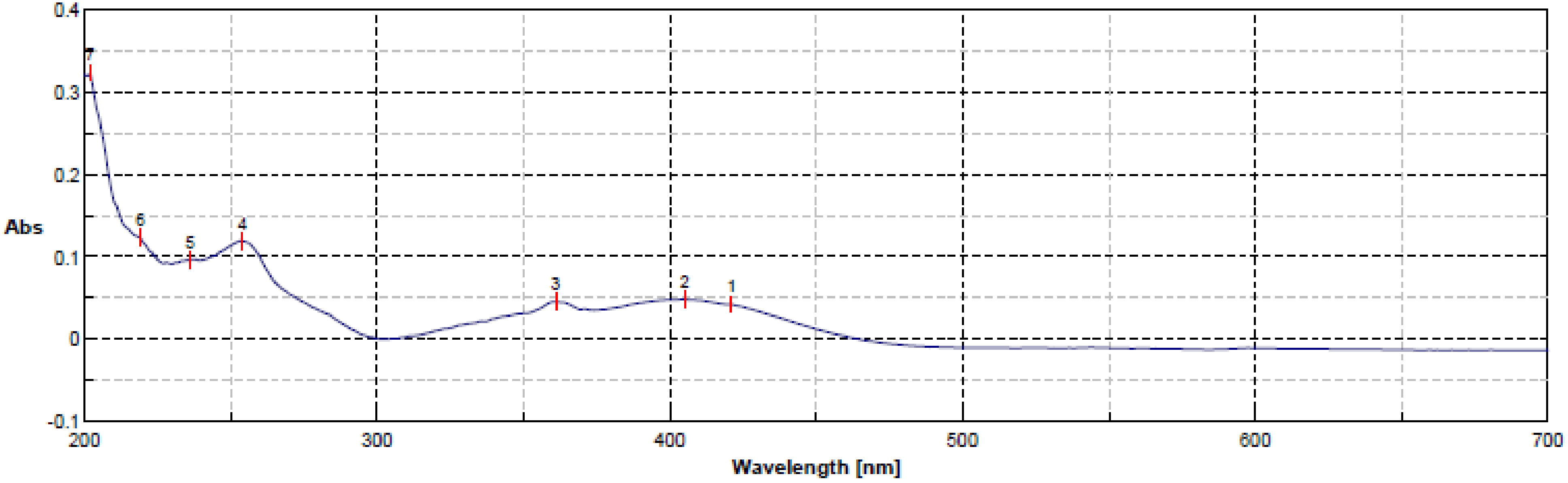

Date	06/06/2014 10:32	File name	F163_B4-19-2A
Model	V-560		
Serial No.	A029479		
Band width	1.0 nm		
Response	Fast		
Measurement range	700 - 200 nm		
Data pitch	1nm		
Scanning speed	400nm/min		
Sample ID	503		
No. of cycle	1		
Sample name			
Operator	Jesús Trujillo Vázquez		
Comment			

No.	nm	Abs	No.	nm	Abs	No.	nm	Abs	No.	nm	Abs	No.	nm	Abs
1	421	0.04196	2	405	0.04804	3	361	0.0452	4	254	0.11924	5	236	0.09636
6	219	0.12292	7	202	0.32404									

:Compound 7:

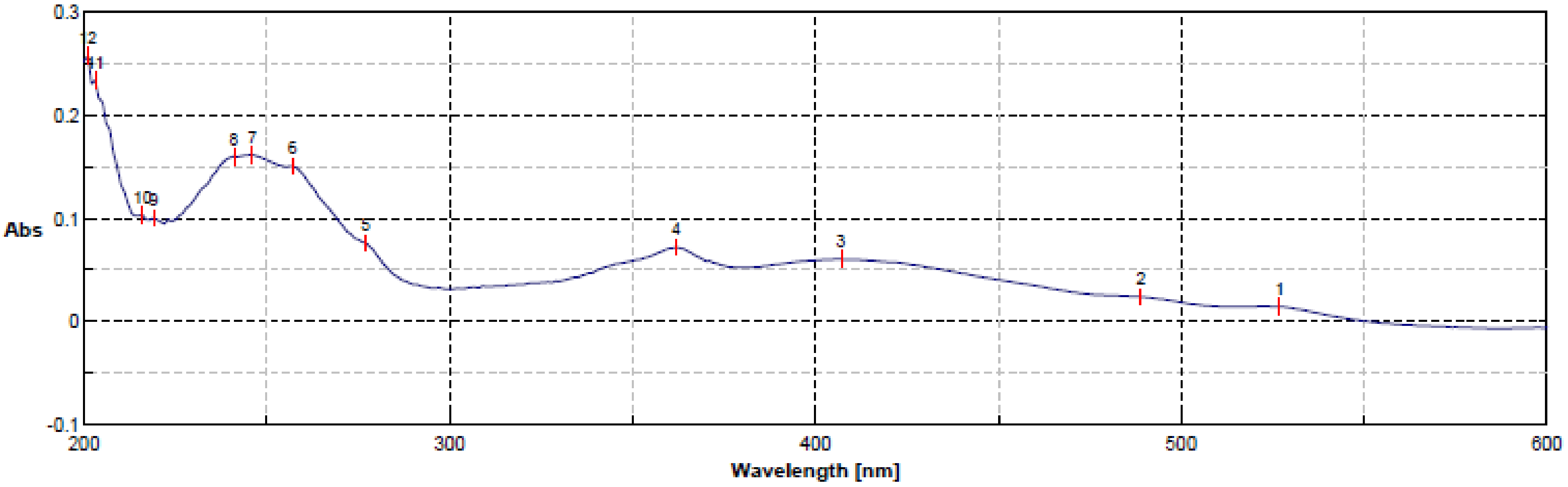

Date	01/08/2013 16:16
Model	V-560
Serial No.	A029479
Band width	1.0 nm
Response	Fast
Measurement range	600 - 200 nm
Data pitch	1nm
Scanning speed	100nm/min
Sample ID	248
No. of cycle	1
Sample name	
Operator	Jesús Trujillo Vázquez
Comment	

File name B2-14_2

No.	nm	Abs	No.	nm	Abs	No.	nm	Abs	No.	nm	Abs	No.	nm	Abs
1	527	0.01373	2	489	0.02362	3	407	0.06006	4	362	0.07196	5	277	0.07598
6	257	0.15053	7	246	0.16157	8	241	0.16023	9	219	0.10013	10	216	0.10323
11	203	0.23437	12	201	0.25816									

Compound **8**:

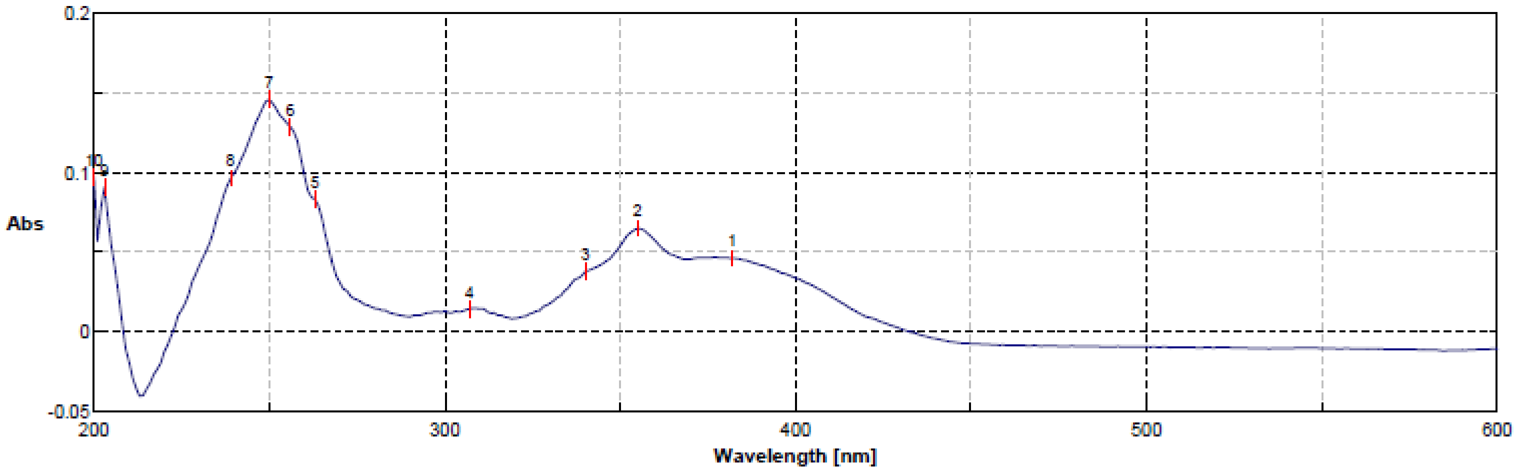

Date	01/08/2013 17:51	File name	B3-22_1
Model	V-560		
Serial No.	A029479		
Band width	1.0 nm		
Response	Fast		
Measurement range	600 - 200 nm		
Data pitch	1nm		
Scanning speed	100nm/min		
Sample ID	263		
No. of cycle	1		
Sample name			
Operator	Jesús Trujillo Vázquez		
Comment			

No.	nm	Abs	No.	nm	Abs	No.	nm	Abs	No.	nm	Abs	No.	nm	Abs
1	382	0.04618	2	355	0.06491	3	340	0.03786	4	307	0.01426	5	263	0.08337
6	256	0.129	7	250	0.14607	8	239	0.09651	9	203	0.0908	10	200	0.09712

Compound **10**:

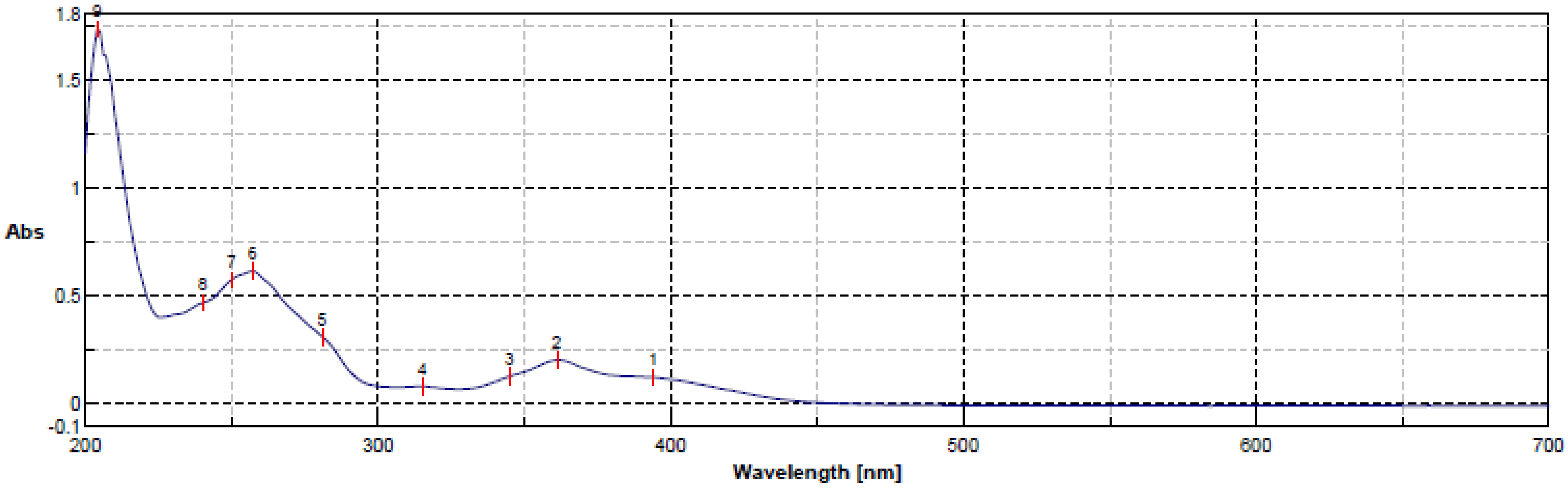

Date	06/06/2014 11:14	File name	F154_B4-03-3B-2
Model	V-560		
Serial No.	A029479		
Band width	1.0 nm		
Response	Fast		
Measurement range	700 - 200 nm		
Data pitch	1nm		
Scanning speed	400nm/min		
Sample ID	513		
No. of cycle	1		
Sample name			
Operator	Jesús Trujillo Vázquez		
Comment			

No.	nm	Abs	No.	nm	Abs	No.	nm	Abs	No.	nm	Abs	No.	nm	Abs
1	394	0.12203	2	361	0.20373	3	345	0.12668	4	315	0.0815	5	281	0.31154
6	257	0.61501	7	250	0.57348	8	240	0.46744	9	204	1.73212			

Compound **11**:

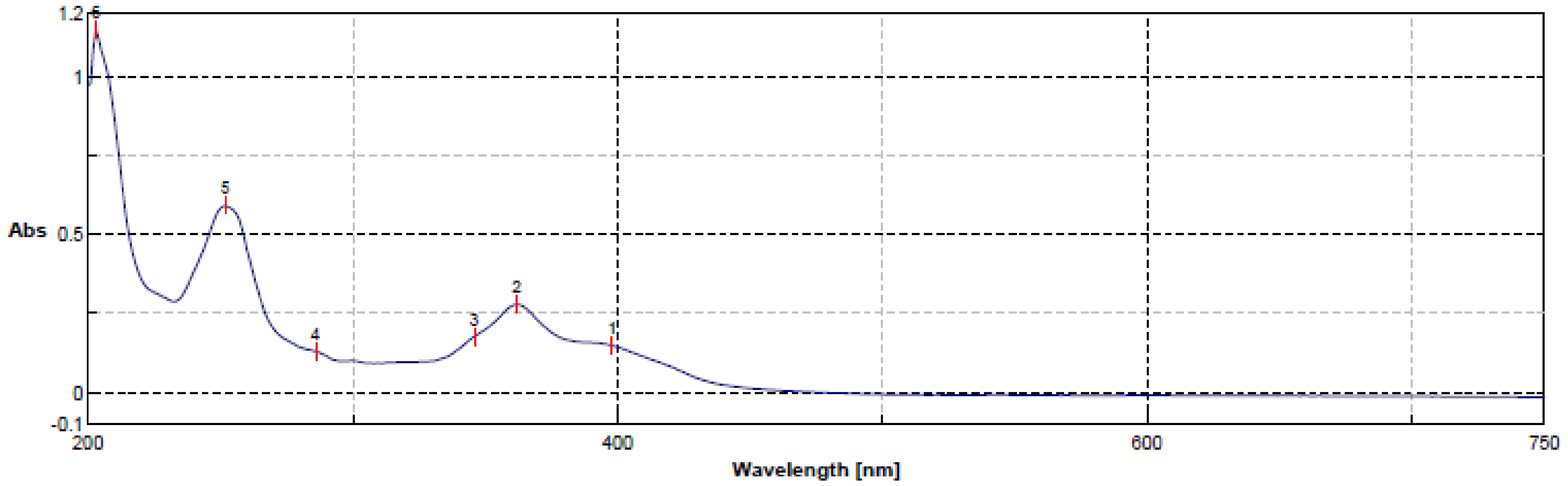

Date	05/06/2014 13:02	File name	F155_B4-04C
Model	V-560		
Serial No.	A029479		
Band width	1.0 nm		
Response	Fast		
Measurement range	750 - 200 nm		
Data pitch	1nm		
Scanning speed	400nm/min		
Sample ID	492		
No. of cycle	1		
Sample name			
Operator	Jesús Trujillo Vázquez		
Comment			

No.	nm	Abs	No.	nm	Abs	No.	nm	Abs	No.	nm	Abs	No.	nm	Abs
1	398	0.14748	2	362	0.27711	3	346	0.17581	4	286	0.12914	5	252	0.59069
6	203	1.14989												

Compound **13**:

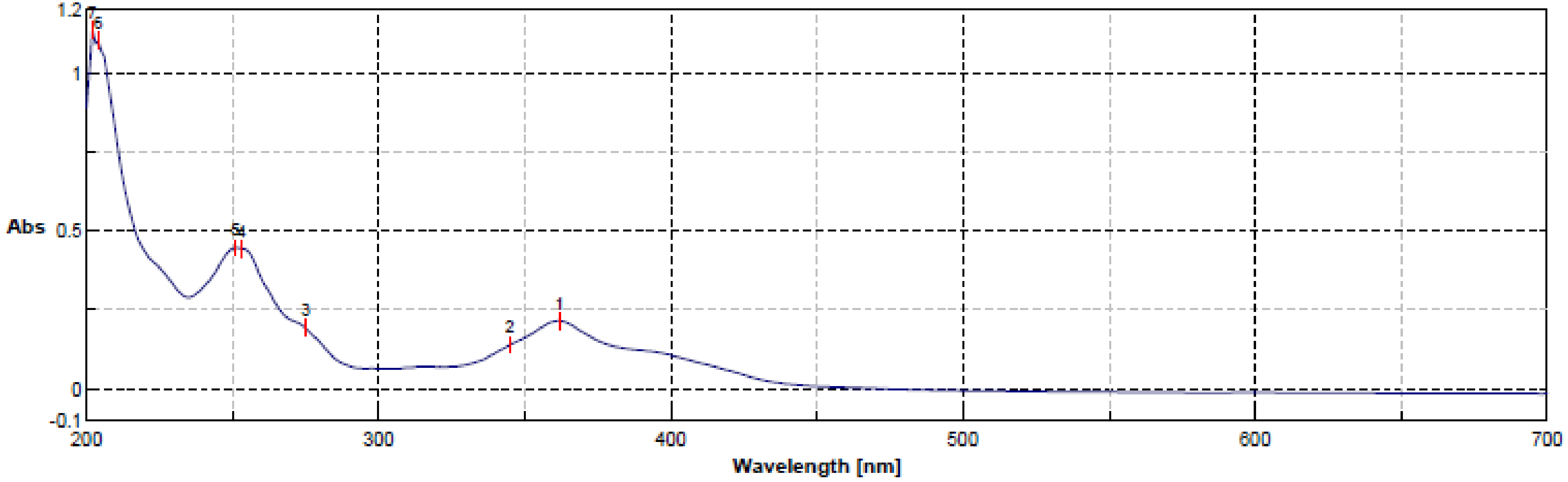

Date	06/06/2014 10:07	File name	F157_B4-26-2B
Model	V-560		
Serial No.	A029479		
Band width	1.0 nm		
Response	Fast		
Measurement range	700 - 200 nm		
Data pitch	1nm		
Scanning speed	400nm/min		
Sample ID	498		
No. of cycle	1		
Sample name			
Operator	Jesús Trujillo Vázquez		
Comment			

No.	nm	Abs	No.	nm	Abs	No.	nm	Abs	No.	nm	Abs	No.	nm	Abs
1	362	0.21457	2	345	0.13824	3	275	0.19101	4	253	0.44353	5	251	0.44598
6	204	1.10257	7	202	1.13926									

Compound **16**:

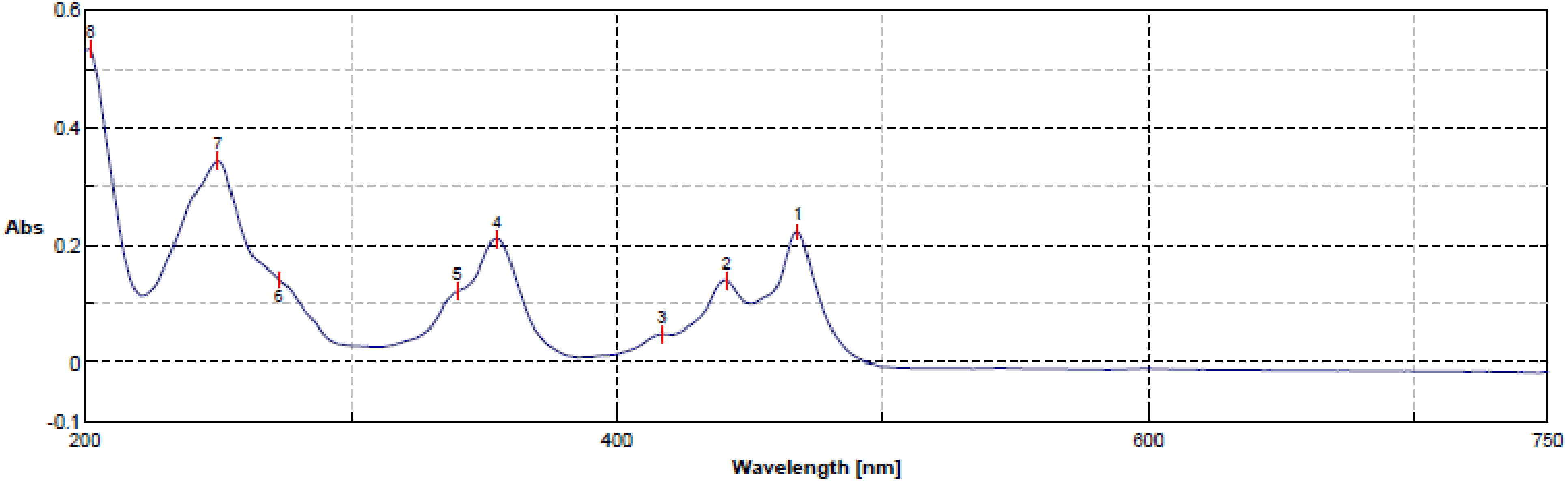

Date	05/06/2014 10:23	File name	F145_B3-94-2B
Model	V-560		
Serial No.	A029479		
Band width	1.0 nm		
Response	Fast		
Measurement range	750 - 200 nm		
Data pitch	1nm		
Scanning speed	400nm/min		
Sample ID	469		
No. of cycle	1		
Sample name			
Operator	Jesús Trujillo Vázquez		
Comment			

No.	nm	Abs	No.	nm	Abs	No.	nm	Abs	No.	nm	Abs	No.	nm	Abs
1	468	0.22252	2	441	0.14027	3	417	0.04852	4	355	0.21078	5	340	0.12111
6	273	0.14135	7	250	0.34416	8	202	0.53432						

Compound **17**:

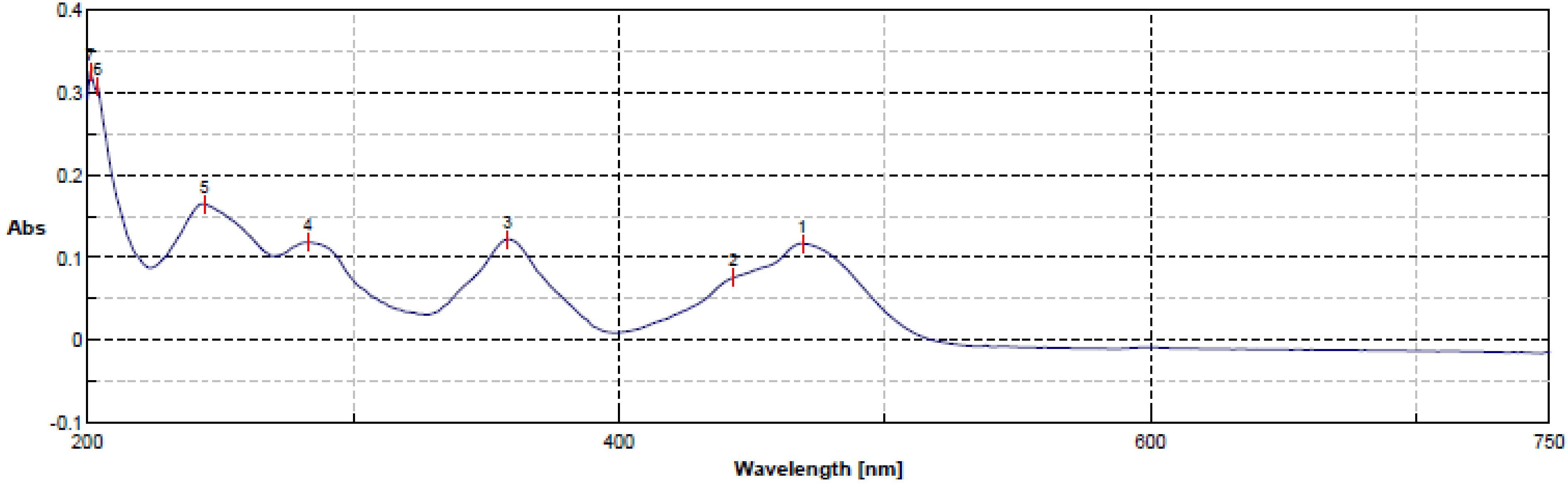

Date	05/06/2014 13:06	File name	F149_B3-98-2B
Model	V-560		
Serial No.	A029479		
Band width	1.0 nm		
Response	Fast		
Measurement range	750 - 200 nm		
Data pitch	1nm		
Scanning speed	400nm/min		
Sample ID	493		
No. of cycle	1		
Sample name			
Operator	Jesús Trujillo Vázquez		
Comment			

No.	nm	Abs	No.	nm	Abs	No.	nm	Abs	No.	nm	Abs	No.	nm	Abs
1	469	0.11686	2	443	0.07562	3	358	0.12195	4	283	0.11841	5	244	0.16502
6	204	0.3077	7	201	0.32585									

Compound **19**:

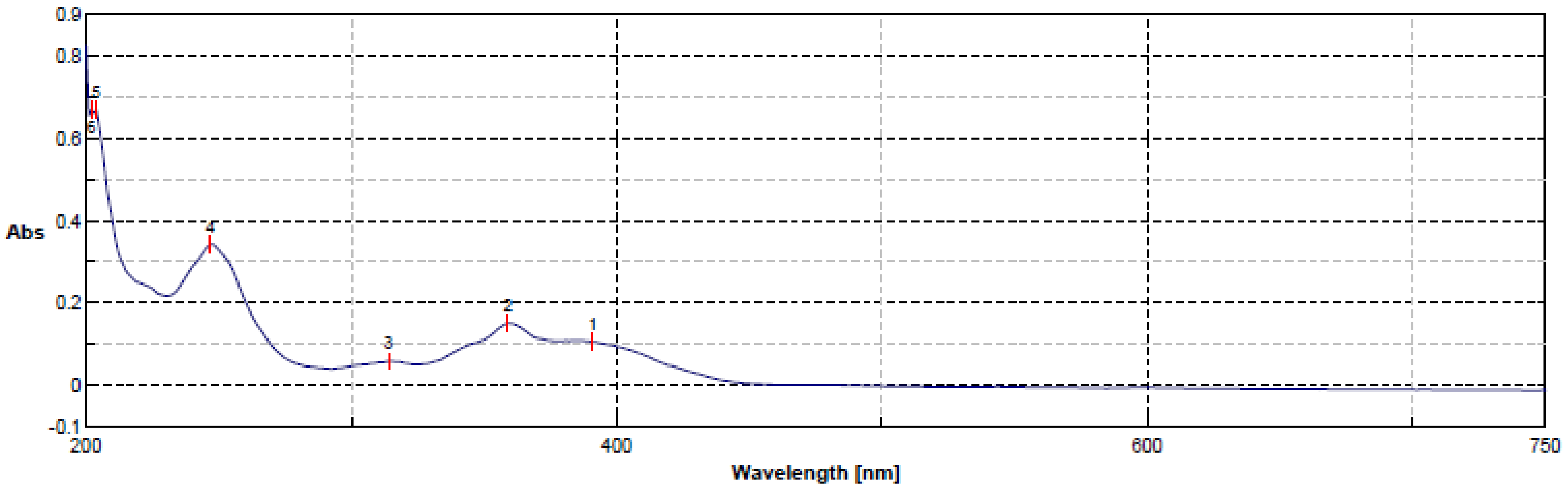

Date	05/06/2014 12:34	File name	F179_B4-42-NR
Model	V-560		
Serial No.	A029479		
Band width	1.0 nm		
Response	Fast		
Measurement range	750 - 200 nm		
Data pitch	1nm		
Scanning speed	400nm/min		
Sample ID	486		
No. of cycle	1		
Sample name			
Operator	Jesús Trujillo Vázquez		
Comment			

No.	nm	Abs	No.	nm	Abs	No.	nm	Abs	No.	nm	Abs	No.	nm	Abs
1	391	0.10501	2	359	0.14954	3	314	0.05771	4	247	0.34143	5	204	0.66952
6	202	0.66986												

Compound **20**:

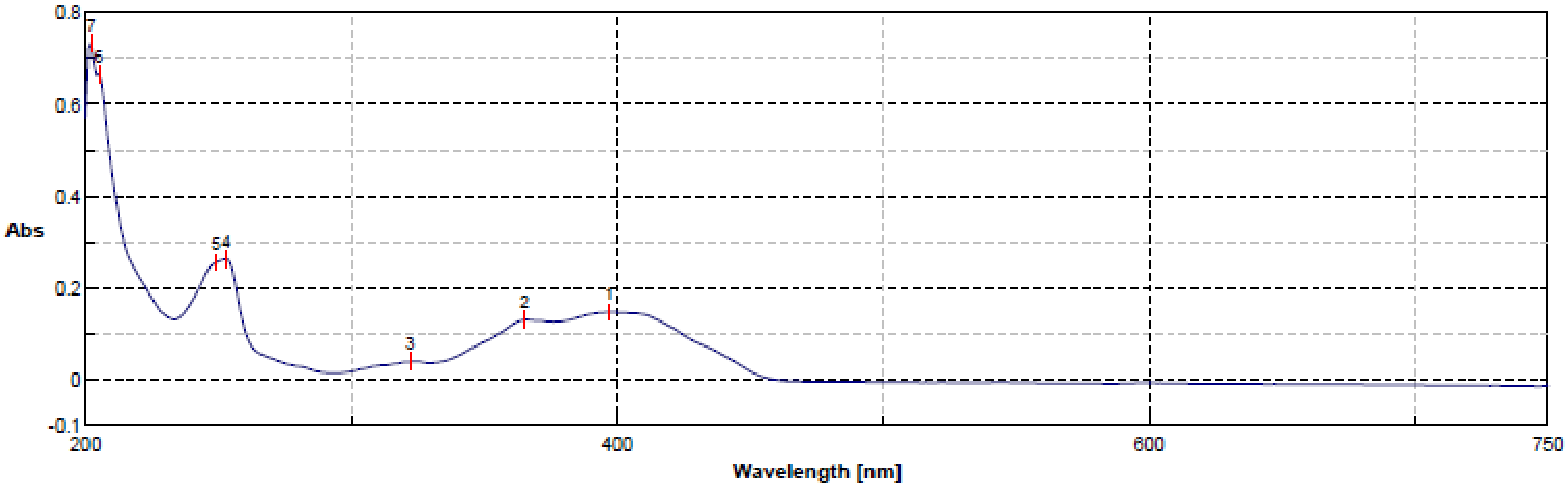

Date	05/06/2014 12:40	File name	F181_B4-47-B
Model	V-560		
Serial No.	A029479		
Band width	1.0 nm		
Response	Fast		
Measurement range	750 - 200 nm		
Data pitch	1nm		
Scanning speed	400nm/min		
Sample ID	487		
No. of cycle	1		
Sample name			
Operator	Jesús Trujillo Vázquez		
Comment			

No.	nm	Abs	No.	nm	Abs	No.	nm	Abs	No.	nm	Abs	No.	nm	Abs
1	397	0.14607	2	365	0.12961	3	322	0.03771	4	253	0.26322	5	249	0.25479
6	205	0.66682	7	202	0.73318									

Compound **21**:

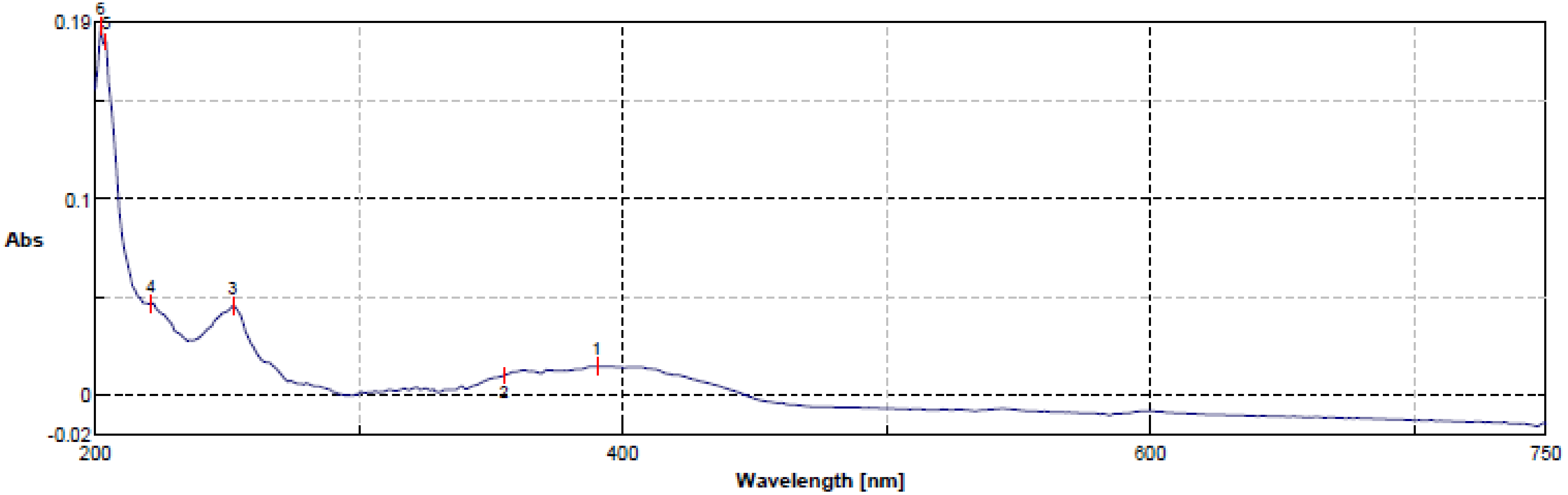

Date	05/06/2014 11:46	File name	F182_B4-54-FO-2
Model	V-560		
Serial No.	A029479		
Band width	1.0 nm		
Response	Fast		
Measurement range	750 - 200 nm		
Data pitch	1nm		
Scanning speed	400nm/min		
Sample ID	483		
No. of cycle	1		
Sample name			
Operator	Jesús Trujillo Vázquez		
Comment			

No.	nm	Abs	No.	nm	Abs	No.	nm	Abs	No.	nm	Abs	No.	nm	Abs
1	390	0.01455	2	355	0.01002	3	252	0.04558	4	221	0.04636	5	204	0.18042
6	202	0.18846												

Compound **25**:

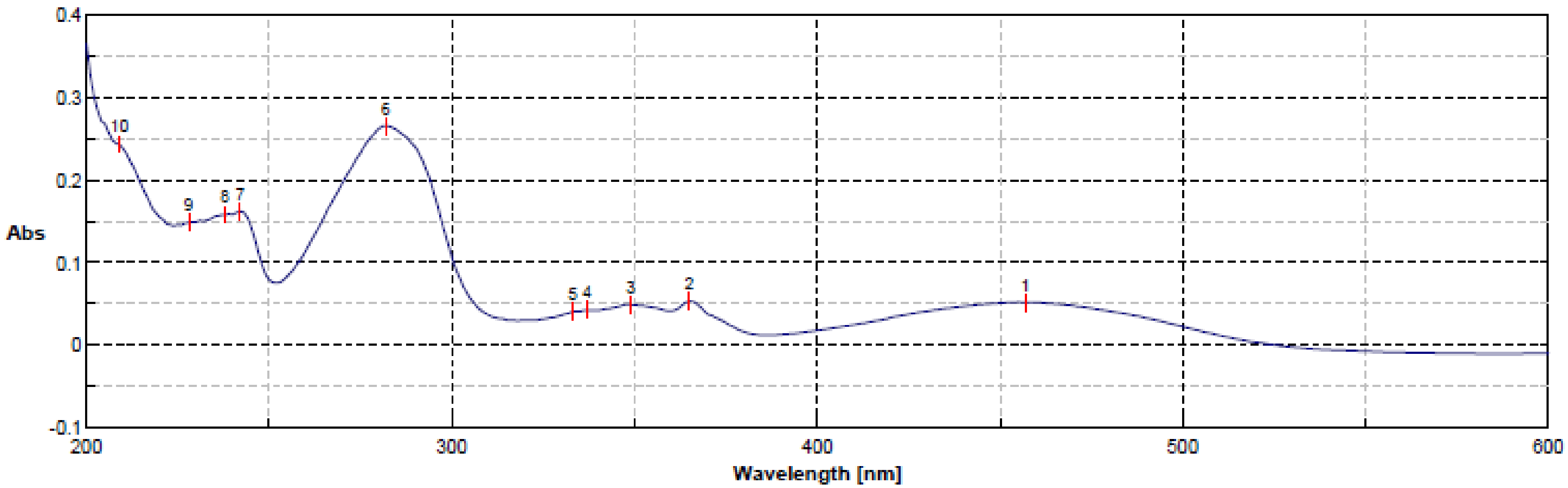

Date	29/07/2013 18:06	File name	B1-83_1
Model	V-560		
Serial No.	A029479		
Band width	1.0 nm		
Response	Fast		
Measurement range	600 - 200 nm		
Data pitch	1nm		
Scanning speed	100nm/min		
Sample ID	228		
No. of cycle	1		
Sample name			
Operator	Jesús Trujillo Vázquez		
Comment			

No.	nm	Abs	No.	nm	Abs	No.	nm	Abs	No.	nm	Abs	No.	nm	Abs
1	457	0.05143	2	365	0.05312	3	349	0.04875	4	337	0.04228	5	333	0.0406
6	282	0.26573	7	242	0.1621	8	238	0.15795	9	228	0.14842	10	209	0.2437

Compound **26**:

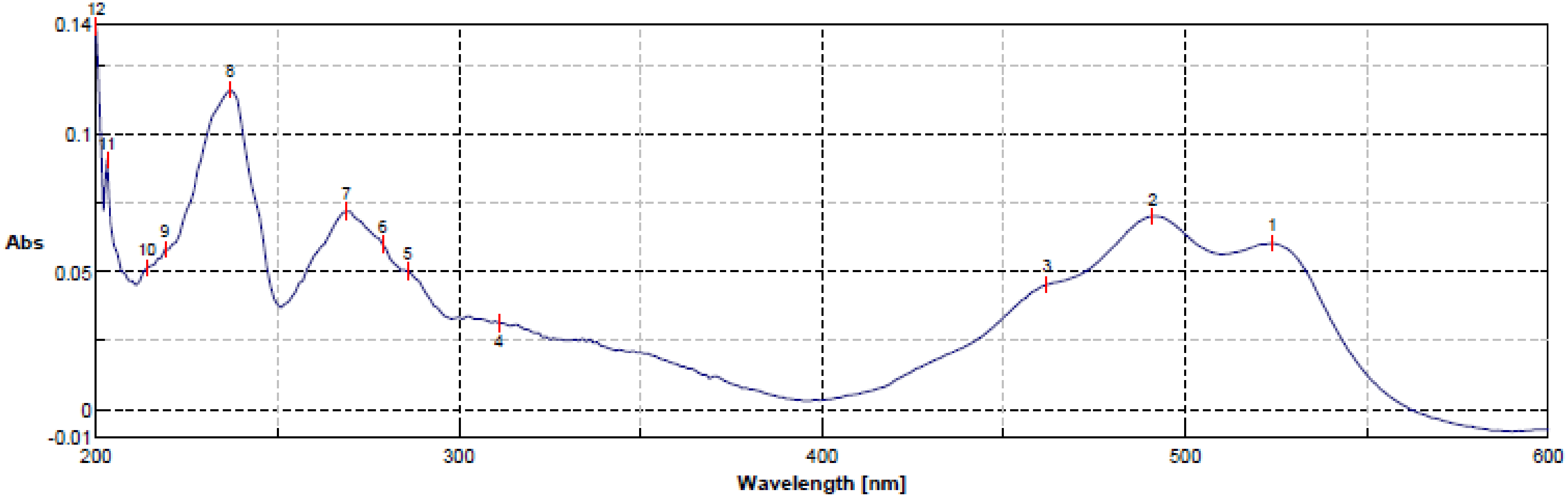

Date	30/07/2013 17:36	File name	B2-02_1
Model	V-560		
Serial No.	A029479		
Band width	1.0 nm		
Response	Fast		
Measurement range	600 - 200 nm		
Data pitch	1nm		
Scanning speed	100nm/min		
Sample ID	245		
No. of cycle	1		
Sample name			
Operator	Jesús Trujillo Vázquez		
Comment			

No.	nm	Abs	No.	nm	Abs	No.	nm	Abs	No.	nm	Abs	No.	nm	Abs
1	524	0.06055	2	491	0.07033	3	462	0.04536	4	311	0.03143	5	286	0.05015
6	279	0.06006	7	269	0.07203	8	237	0.11645	9	219	0.0583	10	214	0.05141
11	203	0.09067	12	200	0.13934									

Compound **33**:

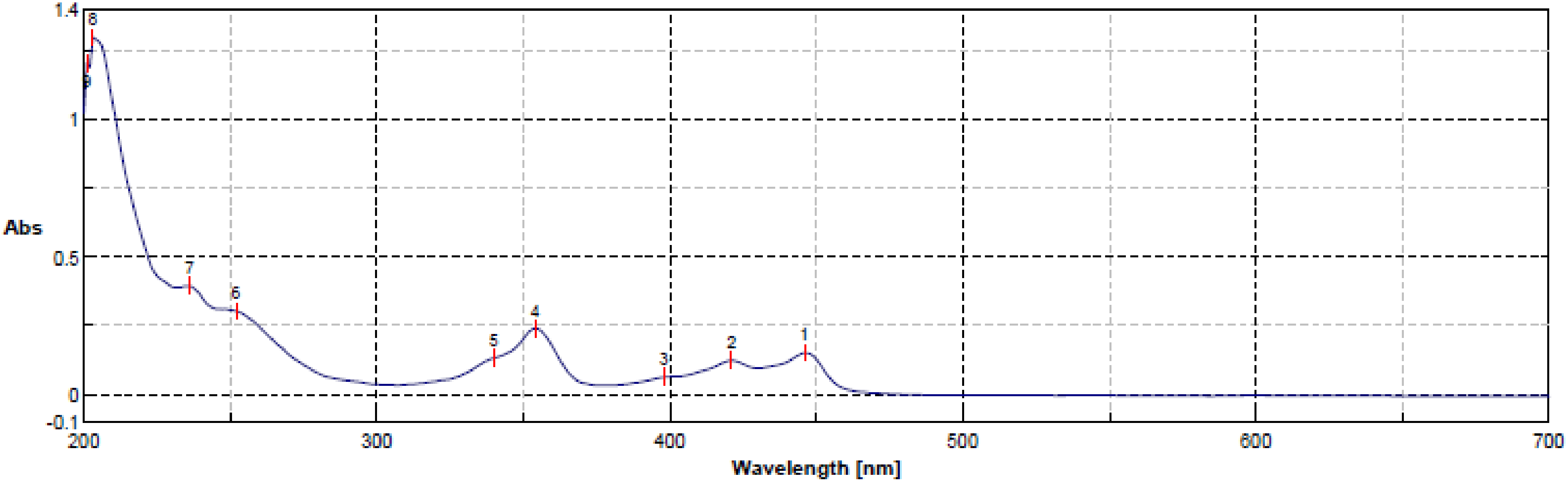

Date	06/06/2014 10:45	File name	F119_B3-46F-3
Model	V-560		
Serial No.	A029479		
Band width	1.0 nm		
Response	Fast		
Measurement range	700 - 200 nm		
Data pitch	1nm		
Scanning speed	400nm/min		
Sample ID	506		
No. of cycle	1		
Sample name			
Operator	Jesús Trujillo Vázquez		
Comment			

No.	nm	Abs	No.	nm	Abs	No.	nm	Abs	No.	nm	Abs	No.	nm	Abs
1	446	0.15074	2	421	0.12305	3	398	0.06203	4	354	0.24047	5	340	0.13165
6	252	0.30317	7	236	0.39466	8	203	1.3013	9	201	1.20796			

Compound **34**:

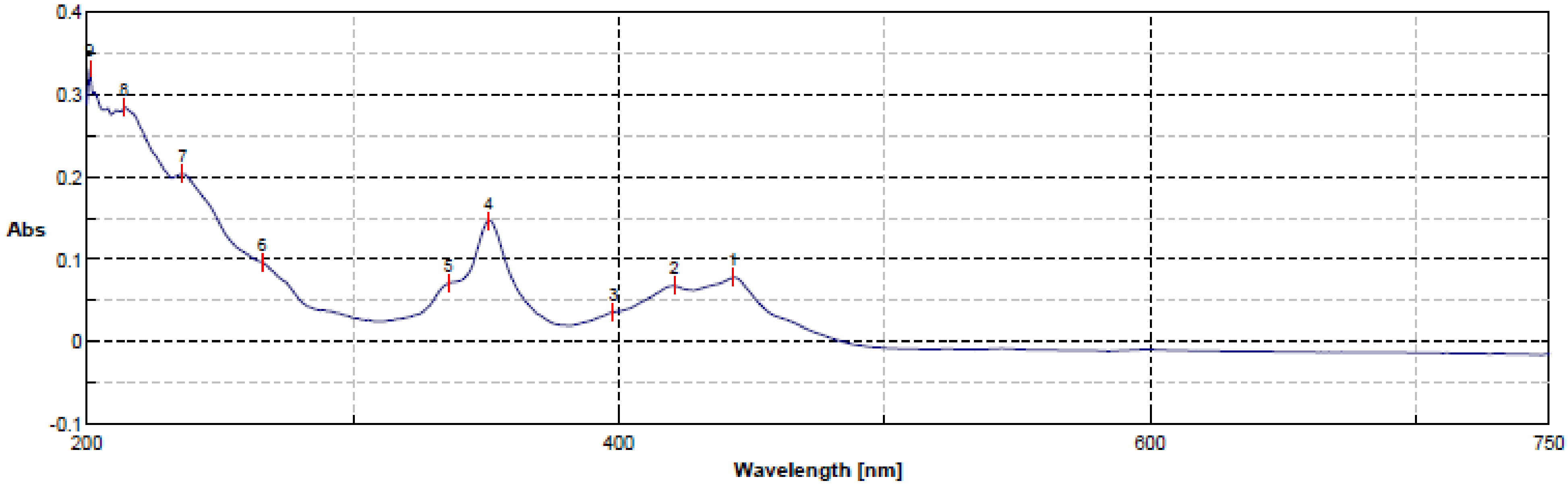

Date	05/06/2014 11:32
Model	V-560
Serial No.	A029479
Band width	1.0 nm
Response	Fast
Measurement range	750 - 200 nm
Data pitch	1nm
Scanning speed	400nm/min
Sample ID	480
No. of cycle	1
Sample name	
Operator	Jesús Trujillo Vázquez
Comment	

File name F143_Grant

No.	nm	Abs	No.	nm	Abs	No.	nm	Abs	No.	nm	Abs	No.	nm	Abs
1	443	0.07763	2	421	0.06767	3	398	0.03551	4	351	0.14743	5	336	0.0707
6	266	0.09647	7	236	0.20362	8	214	0.2849	9	201	0.33206			
